# Gearing Effects on *N*-9-Anth-PyBidine-Cu(OAc)_2_-Catalyzed Asymmetric Direct Haloimidation Reactions of Alkylidenemalononitriles

**DOI:** 10.1021/acs.orglett.4c03405

**Published:** 2024-12-04

**Authors:** Yuri Takagi, Takaaki Saito, Natsuki Mizuno, Takayoshi Arai

**Affiliations:** Soft Molecular Activation Research Center (SMARC), Chiba Iodine Resource Innovation Center (CIRIC), Synthetic Organic Chemistry, Department of Chemistry, Graduate School of Science, Chiba University, 1-33 Yayoi, Inage, Chiba 263-8522, Japan

## Abstract

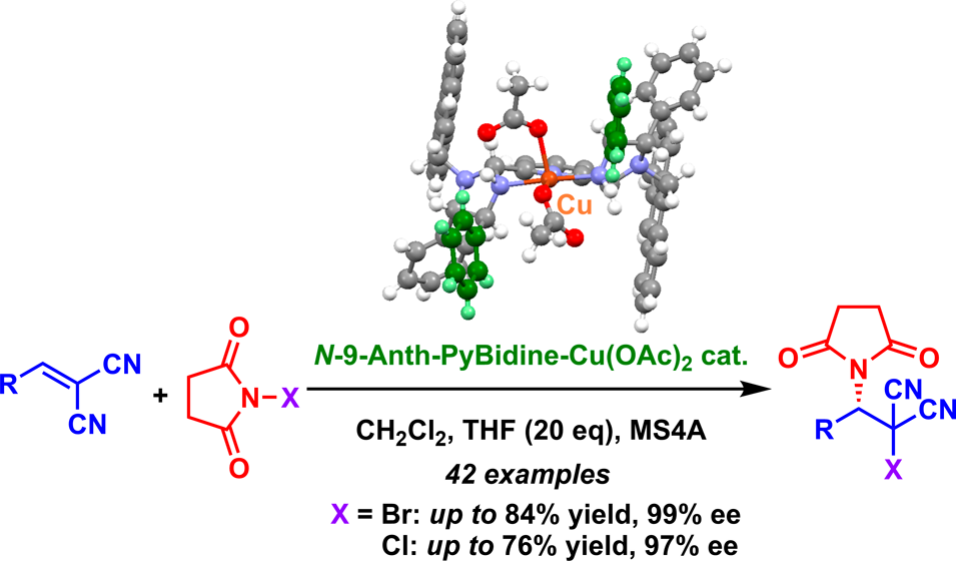

A newly developed *N*-9-anthranylmethyl bis(imidazolidine)pyridine (*N*-9-Anth-PyBidine)-Cu(OAc)_2_ complex catalyzed asymmetric haloimidation reactions of alkylidenemalononitriles with *N*-bromosuccinimide and *N*-chlorosuccinimide, employing the succinimide moiety directly as a copper-bound nucleophile. The anthranyl substituent showed a gearing effect that produced a well-organized asymmetric sphere involving the *N*-H proton of the imidazolidine ring in the ligand. The gearing effect afforded hydrogen bonding-assisted copper-catalyzed haloimidation reactions with high enantioselectivity.

Haloamination of an alkene is a useful reaction for introducing a nitrogen functional group at the vicinal position into a newly formed halogen–carbon bond.^1,2^ Because the reaction can also generate sp^3^ stereogenic centers, a wide range of catalytic asymmetric haloamination reactions have been studied on both intramolecular^3−5^ and intermolecular^6^ processes. Conventional research has utilized cationic halogenating reagents (e.g., imide-derived halogenating compounds) to construct halonium intermediates that subsequently accept nitrogen nucleophiles (Scheme 1, part 1). One drawback of the haloamination using halogenating reagents is the generation of stoichiometric waste for introducing a single halogen atom. As an example, in the case of reactions using *N*-bromosuccinimide (NBS), which is commonly employed for bromination, succinimide is generated as the waste. If the succinimide itself acts as the nitrogen nucleophile, efficient haloimidation is rationally designed without employing an external nitrogen nucleophile (i.e., direct haloimidation), as shown in section 2 of Scheme 1.^7^ To date, only a limited number of waste-free catalytic asymmetric haloamination reactions have been achieved. Masson et al. reported the first catalytic asymmetric bromoimidation of enamide substrates serving as electron-enriched alkenes.^8^ The reaction of enamides with NBS was catalyzed by a chiral phosphoric acid to give the bromoaminal products with high diastereo- and enantioselectivity. The synthesis of diastereomeric bromoaminals was also achieved by using the calcium salt of the chiral phosphoric acid catalyst. Zhou et al. demonstrated the asymmetric bromosulfonamidation of allylic alcohols with *N*,*N*-dibromo-4-nitrobenzenesulfonamide, catalyzed by a cinchona-derived thiourea.^9^

**Scheme 1:**
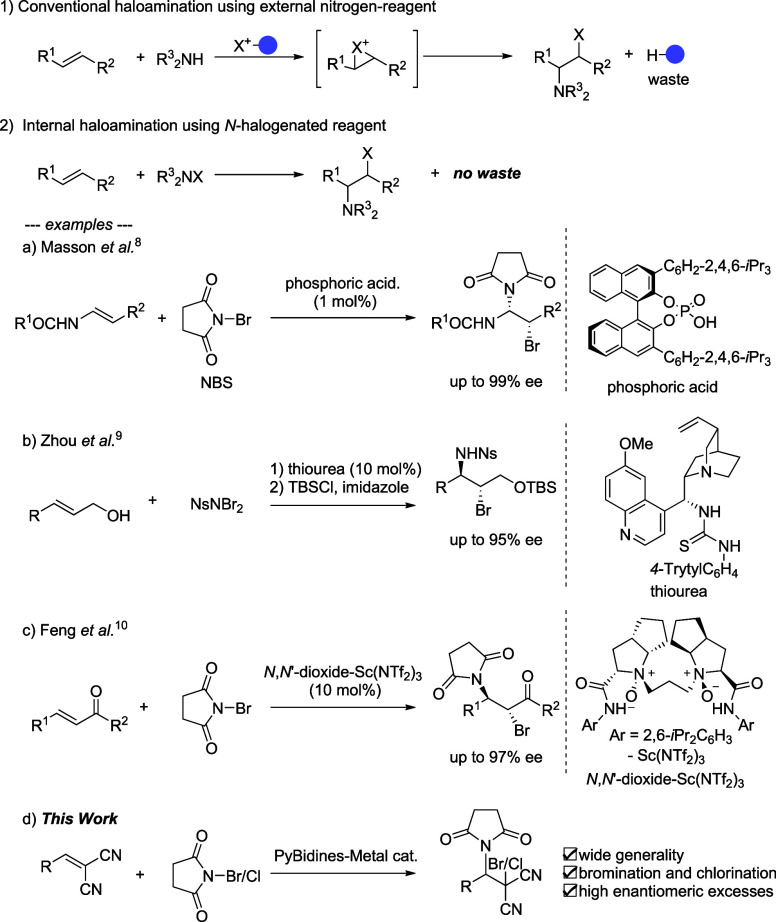
Classification of Haloamination Reactions

For the electron-deficient alkenes, Feng’s group used chiral compound *N*,*N*′-dioxide-Sc(NTf_2_)_3_ as a catalyst to promote the asymmetric bromoimidation of chalcones.^10^ The work presented here demonstrates a new direct catalytic asymmetric halosuccinimidation of alkylidenemalononitriles using an *N*-9-anthranylmethyl-PyBidine-Cu(OAc)_2_ complex. The bis(imidazolidine)pyridine, “PyBidine”, ligand was originally developed for the highly *endo*-selective copper-catalyzed [3+2]-cycloaddition of iminoesters with nitrostyrenes.^11^ Among the applications of the PyBidine-metal catalysts, asymmetric iodolactonization using NIS was also realized using PyBidine-Ni(OAc)_2_.^12^

The development of a direct asymmetric haloimidation catalyzed by a PyBidine-metal complex in this work began with a search for appropriate alkene substrates capable of undergoing bromosuccinimidation by NBS. A survey of various alkenes identified benzylidenemalononitrile (**1a**) as a potential substrate (unsuccessful alkenes are provided in the Supporting Information).

In the absence of an external nucleophile, PyBidine-Ni(OAc)_2_ was found to promote the reaction of **1a** with NBS in CH_2_Cl_2_ to give bromosuccinimidation product **2a** in 18% yield with 46% ee (Table 1, entry 3). Interestingly, PyBidine-Cu(OAc)_2_ exhibited a higher catalytic activity and provided **2a** in 70% yield with 24% ee (entry 4). The *N*-2,4,5-Me_3_C_6_H_4_CH_2_PyBidine (**L2**)-Cu(OAc)_2_ catalyst gave an 80% yield of **2a** with 44% ee, which was superior to the results using **L2**-Ni(OAc)_2_ (entry 9). Using the **L2**-Cu(OAc)_2_ catalyst in THF, the asymmetric induction was improved to 56% ee, although the yield was decreased to 18% (entry 15). The effects of THF as an additive (rather than as the sole solvent) were also carefully examined, and the results are listed in Table 2. A reaction in CH_2_Cl_2_ incorporating 20 equiv of THF relative to the amount of **2a** afforded 54% ee while maintaining the original catalytic activity (entry 3). The positive effects of THF became clearer by the coexistence of MS4A to afford 78% yield with 63% ee (entry 5). Both pybim (**L8**)^13^ and phbox (**L9**)^14^ ligands were ineffective when applied to Cu(OAc)_2_-catalyzed bromoimidation.

**Table 1:**
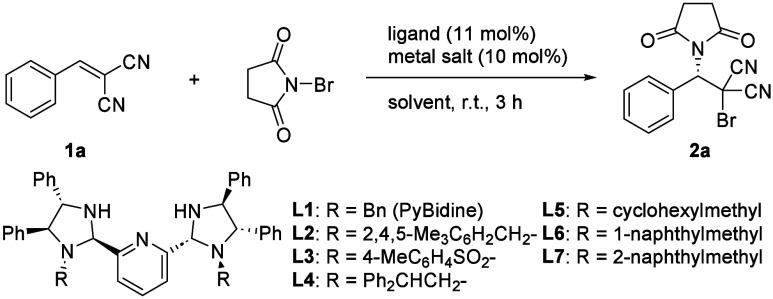
Survey of PyBidine-Metal Catalysts for Bromoimidation of Benzylidenemalononitrile

entry	ligand	metal salt	solvent	yield (%)	ee (%)
1	**L1**	none	CH_2_Cl_2_	nr	–
2	**L1**	Co(OAc)_2_^a^	CH_2_Cl_2_	4	4
3	**L1**	Ni(OAc)_2_^a^	CH_2_Cl_2_	18	46
4	**L1**	Cu(OAc)_2_^b^	CH_2_Cl_2_	70	24
5	**L1**	CuOAc	CH_2_Cl_2_	22	20
6	**L1**	Cu(OTf)_2_	CH_2_Cl_2_	nr	–
7	**L1**	Zn(OAc)_2_	CH_2_Cl_2_	cm^c^	–
8	**L2**	Ni(OAc)_2_^a^	CH_2_Cl_2_	44	38
9	**L2**	Cu(OAc)_2_^b^	CH_2_Cl_2_	80	44
10	**L3**	Cu(OAc)_2_^b^	CH_2_Cl_2_	trace	
11	**L4**	Cu(OAc)_2_^b^	CH_2_Cl_2_	71	15
12	**L5**	Cu(OAc)_2_^b^	CH_2_Cl_2_	73	2
13	**L6**	Cu(OAc)_2_^b^	CH_2_Cl_2_	79	45
14	**L7**	Cu(OAc)_2_^b^	CH_2_Cl_2_	64	46
15	**L2**	Cu(OAc)_2_^b^	THF	18	56
16	**L2**	Cu(OAc)_2_^b^	MeCN	54	rac
17	**L2**	Cu(OAc)_2_^b^	toluene	33	26

a With tetrahydrate.

b With monohydrate.

c Complex mixture.

**Table 2:**
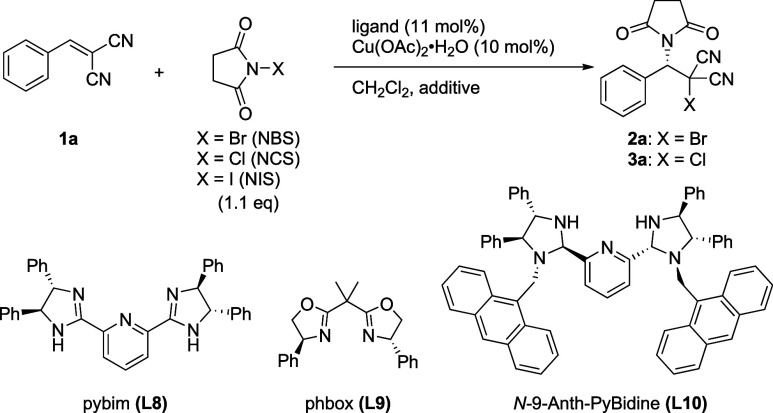
Optimization of the PyBidine-Cu(OAc)_2_-Catalyzed Haloimidation of Benzylidenemalononitrile

entry	X	ligand	additive	temp (°C)	yield (%)	ee (%)
1	Br	**L2**	–	r.t.	80	44
2	Br	**L2**	THF^a^	r.t.	50	42
3	Br	**L2**	THF^b^	r.t.	76	54
4	Br	**L2**	THF^c^	r.t.	49	42
5	Br	**L2**	THF,^b^ MS4A	r.t.	78	63
6	Br	**L8**	THF,^b^ MS4A	r.t.	11	5
7	Br	**L9**	THF,^b^ MS4A	r.t.	48	5
8	Br	**L10**	–	r.t.	61	70
9	Br	**L10**	THF,^b^ MS4A	r.t.	57	74
10	Br	**L10**	THF,^b^ MS4A	–20	68	88
11	Br	**L10**	THF,^b^ MS4A	–40	50	92
12	Br	**L10**	THF,^b^ MS4A	–78	83	87
13^d^	Br	**L10**	THF,^b^ MS4A	–78	82	99
14	Cl	**L10**	THF,^b^ MS4A	–78	83	94
15^d^	Cl	**L10**	THF,^b^ MS4A	–78	78	95
16^d^	I	**L10**	THF,^b^ MS4A	–78	no^e^	–

a With 10 equiv of THF to afford **1a**.

b With 20 equiv of THF to afford **1a**.

c With 50 equiv of THF to afford **1a**.

d In the dark.

e Not obtained.

With the optimization study, *N*-9-anthranylmethylPyBidine (*N*-9-Anth-PyBidine, **L10**) was discovered as a more efficient chiral ligand. Under conditions similar to those in entry 5, **L10**-Cu(OAc)_2_ catalyst gave **2a** with 74% ee (entry 9). For **L8**-Cu(OAc)_2_ catalysis, the asymmetric induction was improved by reducing the reaction temperature. Furthermore, the reaction under shading conditions at −78 °C furnished **2a** in 82% yield with 99% ee (entry 13).

The **L10**-Cu(OAc)_2_ catalyst was also effective in conjunction with chlorosuccinimidation using NCS to produce **3a**. The optimal conditions for chlorosuccinimidation were established on the basis of those employed for the bromosuccinimidation. The shading did not affect the chlorosuccinimidation reaction with regard to either catalytic activity or asymmetric induction (entries 14 and 15).

The generality of **L10**-Cu(OAc)_2_-catalyzed halosuccinimidation is summarized in Scheme 2. Variously substituted benzylidenemalononitriles were successfully employed in both catalytic asymmetric bromoimidation to give **2a**–**r** and chloroimidation to give **3a**–**r** in a highly enantioselective manner. As a heteroaromatic substrate, from 2-(thiophen-2-ylmethylene)malononitrile, **3s** was obtained in 61% yield with 72% ee by carrying out the reaction at −40 °C. For the aliphatic examples, cyclohexyl-substituted **2t** was produced with 97% ee and **3t** with 96% ee.

**Scheme 2:**
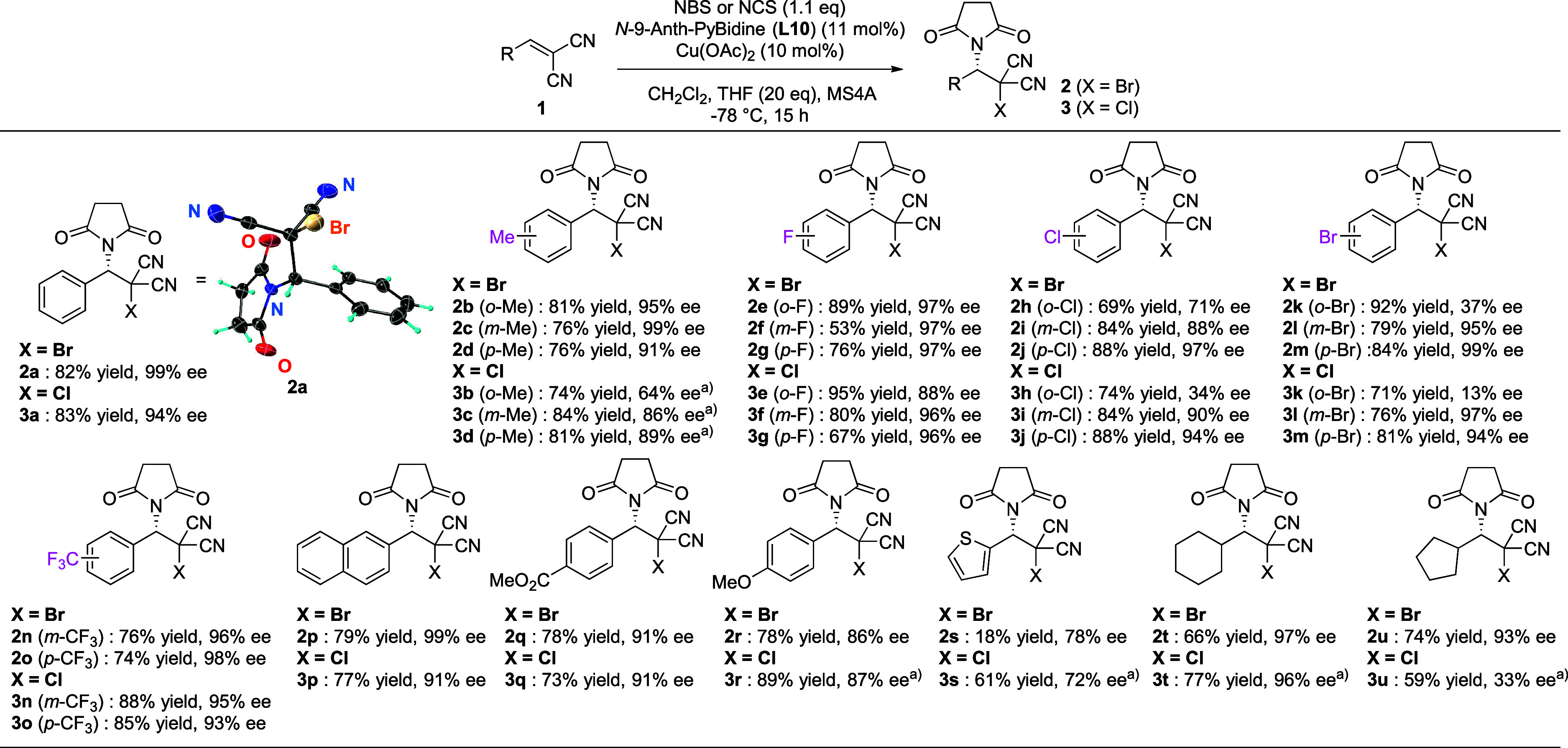
Substrate Scope for the **L10**-Cu(OAc)_2_-Catalyzed Halosuccinimidation Reaction Reaction carried out at −40 °C. The X-ray structure of **2a** is shown with 50% probability ellipsoids.

The synthetic utility of the haloimidation products is demonstrated in Scheme 3. The photoinduced reaction of bromoimidation product **2a** (having 96% ee) with phenyl acetylene gave cross coupling adduct **4a** in 66% yield with 96% ee.^15^ In addition, one or both cyano groups of **3a** were successfully converted into an amide functionality.^16^

**Scheme 3:**
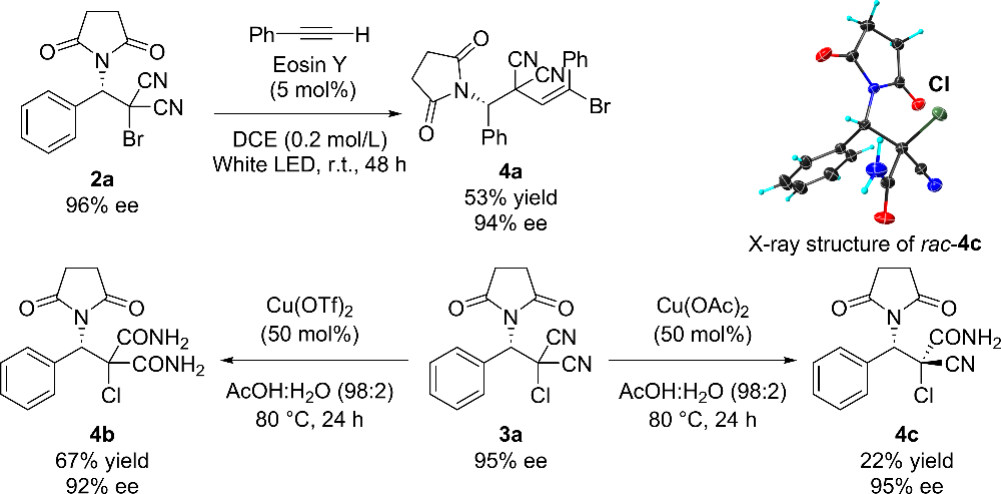
Synthetic Transformation of **2a** and **3a** The X-ray structure of *rac*-**4c** is shown with 50% probability ellipsoids.

The control experiments performed to elucidate the reaction mechanism are summarized in Scheme 4.

**Scheme 4:**
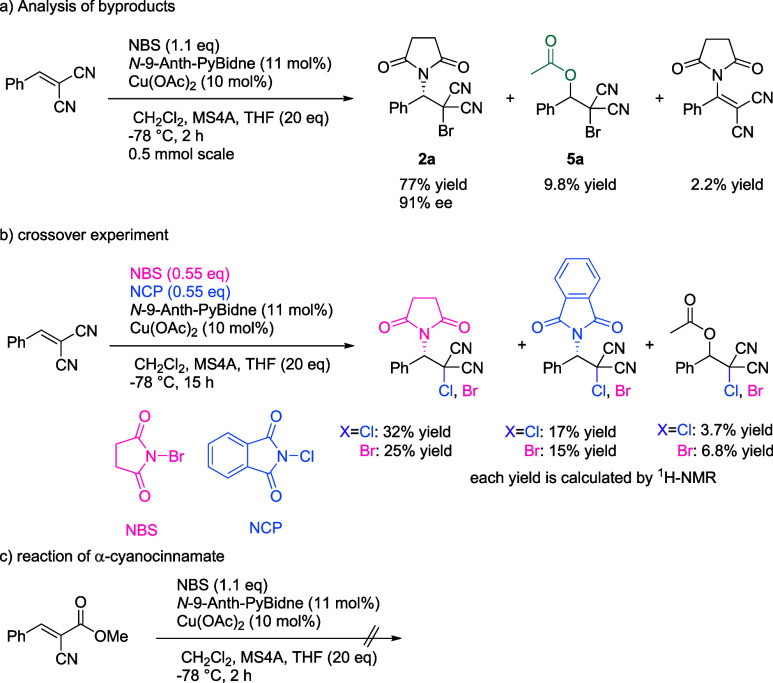
Control Experiments

Even with the optimized conditions shown in Scheme 2, several unidentified products were found in the reaction mixture. A reaction on the 0.5 mmol scale using 10 mol % **L10**-Cu(OAc)_2_ as the catalyst allowed these byproducts to be isolated and analyzed (Scheme 4a). Importantly, a β-acetoxy-brominated product (**5a**) was found to be produced in 9.8% yield. When the reaction was carried out using NBS (0.55 equiv) and NCP (0.55 equiv), chlorosuccinimidation and bromophthalimidation products were obtained along with comparable yields of the bromosuccimidation and chlorophthalimidation products, respectively (Scheme 4b). This crossover suggests that the haloimidation reaction proceeds in a stepwise rather than concerted manner. The reaction of α-cyanocinnamate was found not to proceed well with this catalyst (Scheme 4c).

The structures of *N*-Bn-PyBidine-Cu(OAc)_2_ and *N*-9-Anth-PyBidine-Cu(OAc)_2_ were evaluated and compared on the basis of X-ray crystallographic analyses (Figure 1). The square pyramidal copper complexes were found to both have similar geometries and coordination. The distance between the Cu center and oxygen atom of the apical acetoxy functional group was determined to be longer than that to the equatorial acetoxy functional group, indicating that the former would act as a stronger base.

**Figure 1:**
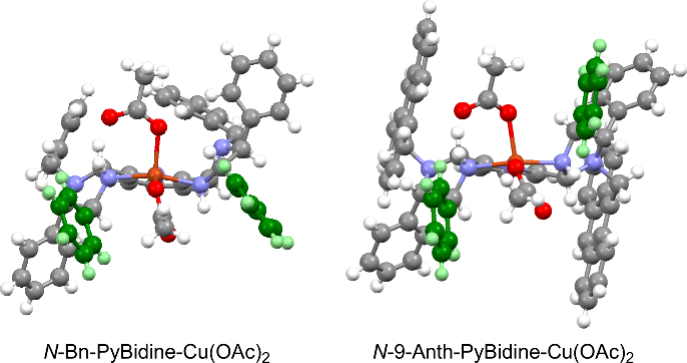
Structures of PyBidine-Cu(OAc)_2_ complexes as determined by X-ray diffraction, shown with 50% probability ellipsoids.

The green phenyl groups exhibit different orientations in the two complexes. In *N*-Bn-PyBidine-Cu(OAc)_2_, the two phenyl rings are located in the third and fourth quadrants. In contrast, in the case of *N*-9-Anth-PyBidine-Cu(OAc)_2_, these two moieties are situated in the first and third quadrants due to the remote effect (i.e., the gearing effect) of the *N*-9-Anth substituent, which contributed to the production of the efficient asymmetric reaction sphere.

On the basis of the control experiments shown in Scheme 4 and the structure ascertained for *N*-9-Anth-PyBidine-Cu(OAc)_2_, a proposed catalytic cycle is provided in Scheme 5.

**Scheme 5:**
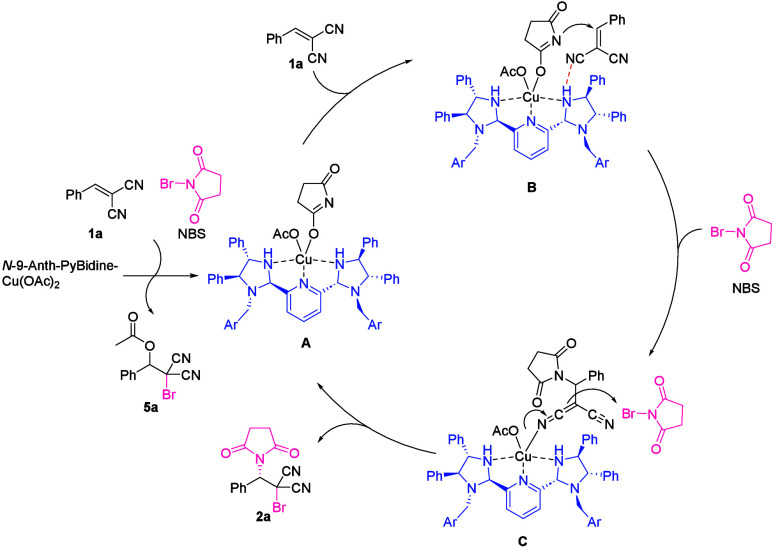
Proposed Catalytic Cycle

The reaction starts with the formation of a nucleophilic imide species on the copper catalyst. The acetoxy anion at an apical position on *N*-9-Anth-PyBidine-Cu(OAc)_2_ attacks the benzylidenemalononitrile, and the bromination at the α-position of the malononitrile would give β-acetoxy-brominated product **5a** with the formation of imide species on the copper catalyst (**A**). Note also that the direct nucleophilic attack of the acetoxy anion on the NBS represents an alternative pathway to generate the copper-bound imide species, although AcOBr was not detected during analyses using electrospray ionization mass spectrometry (ESI-MS). The formation of imide species on the copper catalyst (**A**) was suggested by the detection of a fragment at *m*/*z* 1064.3824 corresponding to [*N*-9-AnthPyBidine-Cu(C_4_H_8_NO_2_)]^+^ calculated for *m*/*z* 1064.3834. Following the nucleophilic addition of the imide anion to benzylidenemalononitrile (**B**), a copper-bound enolate is obtained from β-imidated malononitrile (**C**). The bromination of the enolate by another NBS molecule gives bromoimidation product **2a** with regeneration of the imidated copper catalyst (**A**). The fact that the reaction of electron-enriched **3b**–**d** and **3r** did not procced at −78 °C suggested the imide addition was the rate-determining and stereodetermining step, not via formation of the halonium intermediate.

The enantioselective imidation of the benzylidenemalononitrile at stage **B** in Scheme 5 was examined via density functional theory (DFT) calculations (Figure 2). An O-bound imide anion is generated on the square pyramidal copper center of the *N*-9-Anth-PyBidine-Cu complex (Figure 2a). When the benzylidenemalononitrile approaches the imide anion from the front side of the complex, the NH proton of the imidazolidine ligand and three aromatic CH protons (two anthracenyl CHs and one green-colored phenyl CH) would form hydrogen bonds with the *trans*-nitrile functional group to the benzene ring of benzylidenemalononitrile (Figure 2b). With the assistance of a hydrogen bonding network, the O-bound copper-imide anion would react with the *si* face of the benzylidenemalononitrile. When the *cis*-nitrile functional group of benzylidenemalononitrile forms hydrogen bonds to promote the *re* face reaction, the benzene ring of benzylidenemalononitrile shows steric repulsion with the substituents constructing the square pyramidal copper complex. The fact that the reaction of α-cyanocinnamate did not occur to any appreciable extent, as examined in Scheme 4c, also suggests the importance of the *trans*-nitrile functional group as a means of activation via the hydrogen bonding network. The TS model of Figure 2 explains the formation of (*S*)-enriched **2a** using the (*S*,*S*)-diphenylethrenediamine-derived *N*-9-Anth-PyBidine-Cu(OAc)_2_ catalyst.

**Figure 2:**
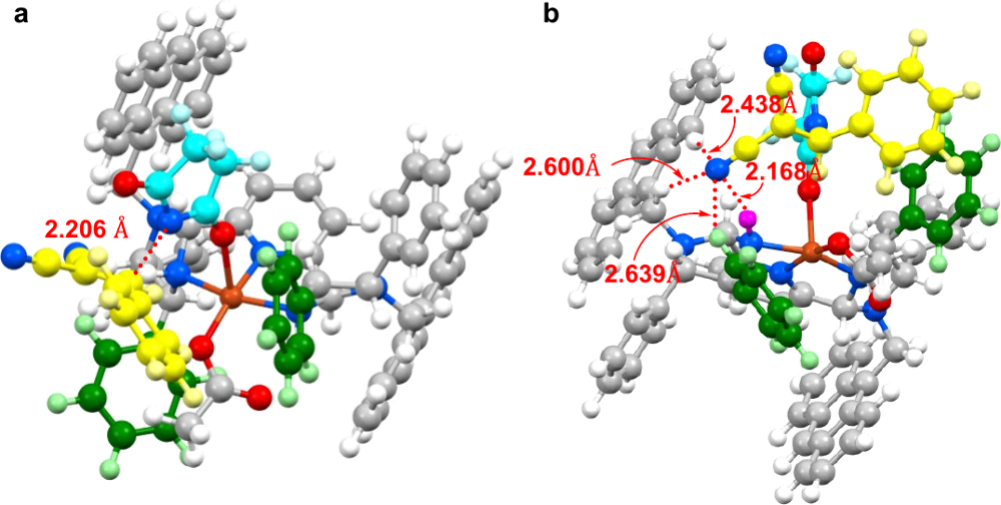
TS model for the enantioselective imidation of benzylidenemalononitrile (**1a**) using the *N*-9-Anth-PyBidine-Cu(OAc)_2_ catalyst (yellow for CHs of **1a**, light blue foor CHs of succinimide, and purple for the NH proton forming a hydrogen bond): (a) direction to see TS for forming a carbon–nitrogen bond and (b) direction to see the hydrogen bonds with the nitrile group of **1a**.

In conclusion, the first general catalytic asymmetric haloimidation of alkylidenemalononitriles was achieved using a newly developed *N*-9-Anth-PyBidine-Cu(OAc)_2_ catalyst. The anthranyl substituent provided a gearing effect for the construction of an efficient asymmetric reaction sphere for conducting a hydrogen bonding-assisted metal-catalyzed reaction.

## Data Availability

The data underlying this study are available in the published article and its Supporting Information.

## Supporting Information Available

Experimental procedures, compound characterization, crystal data, and DFT calculations (PDF)
