# Supplementary material for: Gearing Effects on N-9-Anth-PyBidine-Cu(OAc)2-Catalyzed Asymmetric Direct Haloimidation Reactions of Alkylidenemalononitriles
Source: Org Lett. 2024 Dec 4;26(50):10678–83. doi: 10.1021/acs.orglett.4c03405 (PMC11667719; doi:10.1021/acs.orglett.4c03405)
Supplement: Supplementary file 1 — ol4c03405_si_001.pdf [file ol4c03405_si_001.pdf]

# Supporting Information

# Gearing Effects on *N*-9-Anth-PyBidine-Cu(OAc)$_2$-Catalyzed Asymmetric Direct Haloimidation Reactions of Alkylidenemalononitriles

Yuri Takagi, Takaaki Saito, Natsuki Mizuno, Takayoshi Arai*

Soft Molecular Activation Research Center (SMARC)
Chiba Iodine Resource Innovation Center (CIRIC)
Synthetic Organic Chemistry, Department of Chemistry, Graduate School of Science, Chiba University
1-33 Yayoi, Inage, Chiba, Japan

tarai@faculty.chiba-u.jp

## Contents

1. General S3

2. General procedure of asymmetric haloimidation catalyzed *N*-9-Anth-PyBidine complex S3

3. Screening of other alkenes S3

4. Optimization of chloroimidation S4

5. ESI-Mass analysis of metal complex S4

6. NMR analysis of crossover experiment S5

7. Transformation of haloimidation products S5

8. 2 mmol scale experiment S6

9. Analytical data for products S7

10. X-ray crystallographic analysis S18

11. DFT calculations S27

12. NMR spectra S32

13. HPLC chart S77

## 1. General

Dry solvents were purchased from commercial suppliers and used without further purification. Analytical thin-layer chromatography (TLC) was performed on glass plates coated with 0.25 mm 230-400 mesh silica gel containing a fluorescent indicator (Merck, #1.05715.0009). Silica-gel column chromatography was performed on Kanto silica gel 60 N (spherical, neutral 40-50 μm). High-resolution mass spectra were recorded on a Thermo Fisher Scientific Exactive Orbitrap mass spectrometer (ESI, APCI). $^{1}$H-NMR spectra were recorded on JEOL ECS-400 (400MHz), ECA-500 (500MHz) spectrometers. Chemical shifts of $^{1}$H-NMR spectra were reported relative to tetramethyl silane (δ 0). $^{13}$C-NMR spectra were recorded on ECA-500 (125MHz) spectrometers. Chemical shifts of $^{13}$C-NMR spectra were reported relative to $CDCl_3$ (δ 77.0). Splitting patterns were reported as s, singlet; d, doublet; t, triplet; m, multiplet; br, broad.

General experimental details for synthesis PyBidine derivative have been described.[1,2] Benzylidenemalononitrile derivatives were synthesized according to known procedure.[3]

1) T. Arai, A. Mishiro, N. Yokoyama, K. Suzuki, H. Sato, *J. Am. Chem. Soc.* **2010**, *132*, 5338.

2) T. Arai, T. Suzuki, T. Inoue, S. Kuwano, *Synlett* **2017**, *28*, 122.

3) E. Ogino, A. Nakamura, S. Kuwano, T. Arai, *Org. Lett.* **2021**, *23*, 1980.

## 2. General procedure of asymmetric haloimidation catalyzed *N*-9-Anth-PyBidine complex

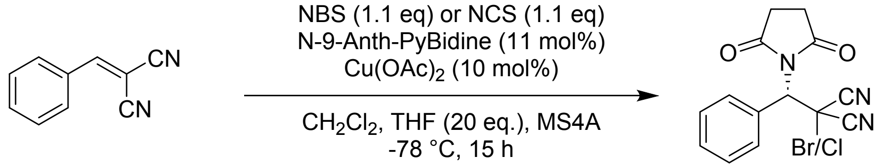


*N*-9-Anth-PyBidine (0.011 mmol), $Cu(OAc)_2$ (0.010 mmol) were added to a test tube equipped with a stir bar and a three-way cock under Ar. Dichloromethane (1.00 ml) was added to the test tube and the mixture was stirred over three hours. After removal the solvent under reduced pressure, alkylidenemalononitrile (0.10 mmol) and activated 4Å MS (100 mg) was added, and the test tube was covered with aluminum foil. Dichloromethane (1.00 ml) and THF (0.16 ml) was added and stirred under Ar at -78 °C. After keeping over 30 minutes at -78 °C, NBS or NCS (0.11 mmol) was added. The reaction mixture was stirred for 15 h and removed solvent under reduced pressure, the residue was purified silica-gel column chromatography used Isolera™ Prime. The enantiomeric excess of products was determined by chiral stationary phase HPLC using a Daicel Chiralpak IC-3,IBN-3, IA,AZ-3,OZ-3 and OD-H column. Especially, for the reaction using NBS, reaction was carried out under shading condition (i.e. The reaction vessel was covered with aluminum foil).

## 3. Screening of other alkenes (Table S1)

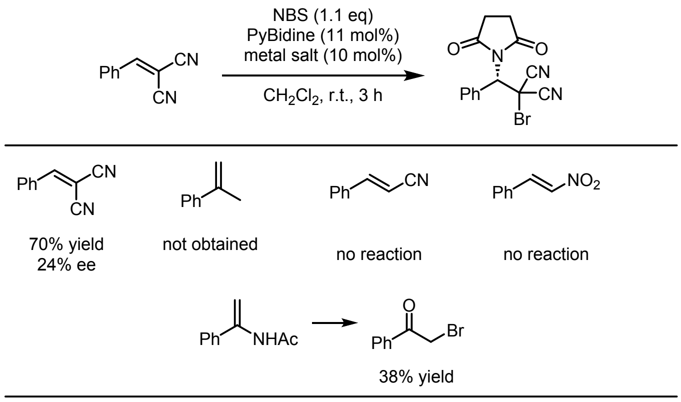

## 4. Optimization of chloroimidaiton

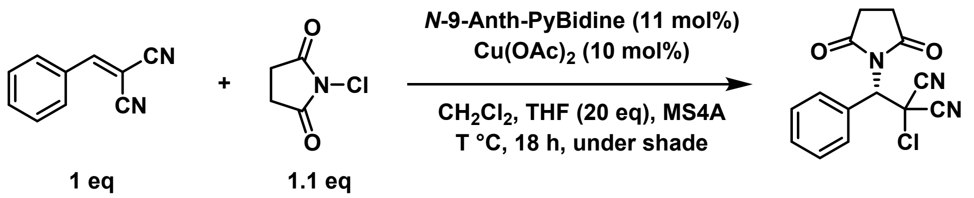


| entry | T (°C) | yield (%) | ee (%) |
|---|---|---|---|
| 1 | r.t. | 80 | 57 |
| 2 | 0 | 74 | 74 |
| 3 | -20 | 81 | 82 |
| 4 | -40 | 86 | 87 |
| 5 | -78 | 78 | 95 |
| 6* | -78 | 83 | 94 |

*under light

## 5. ESI-Mass analysis of metal complex (Figure.S1)

**L10**+Cu(OAc)$_2$+NBS

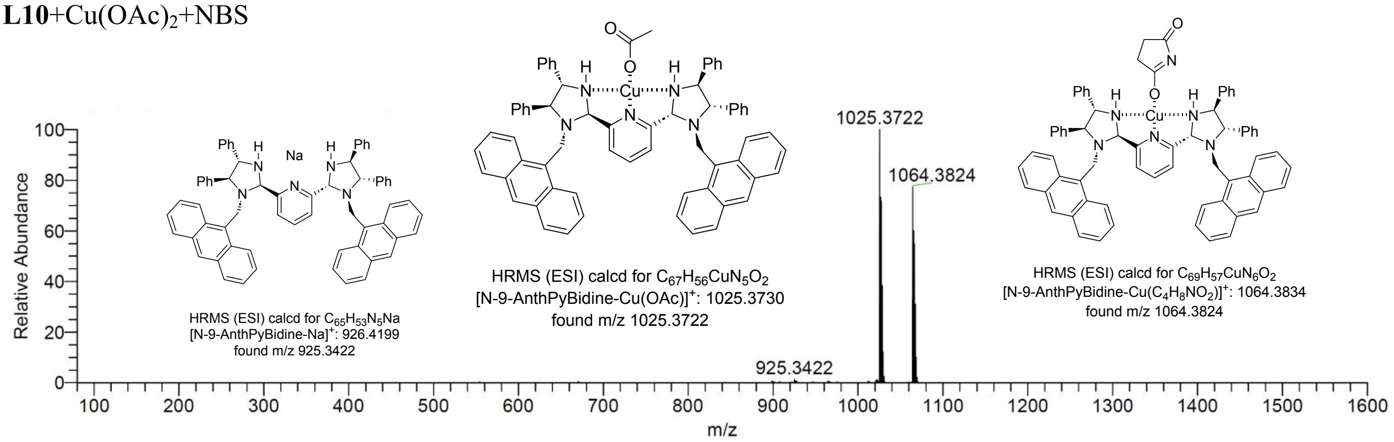


**L10**+Cu(OAc)$_2$+NCS

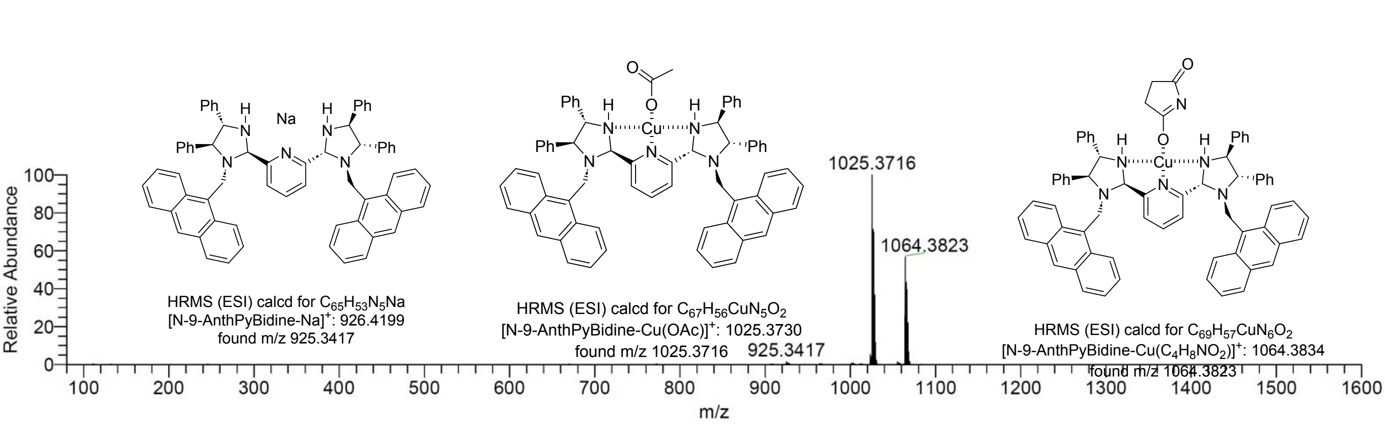

## 6. NMR analysis of crossover experiment (Figure.S2)

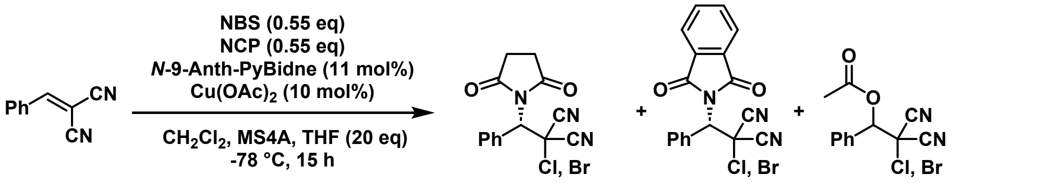


X=Cl: 32% yield (A) Br: 25% yield (B)
X=Cl: 17% yield (C) Br: 15% yield (D)
X=Cl: 3.7% yield (E) Br: 6.8% yield (F)

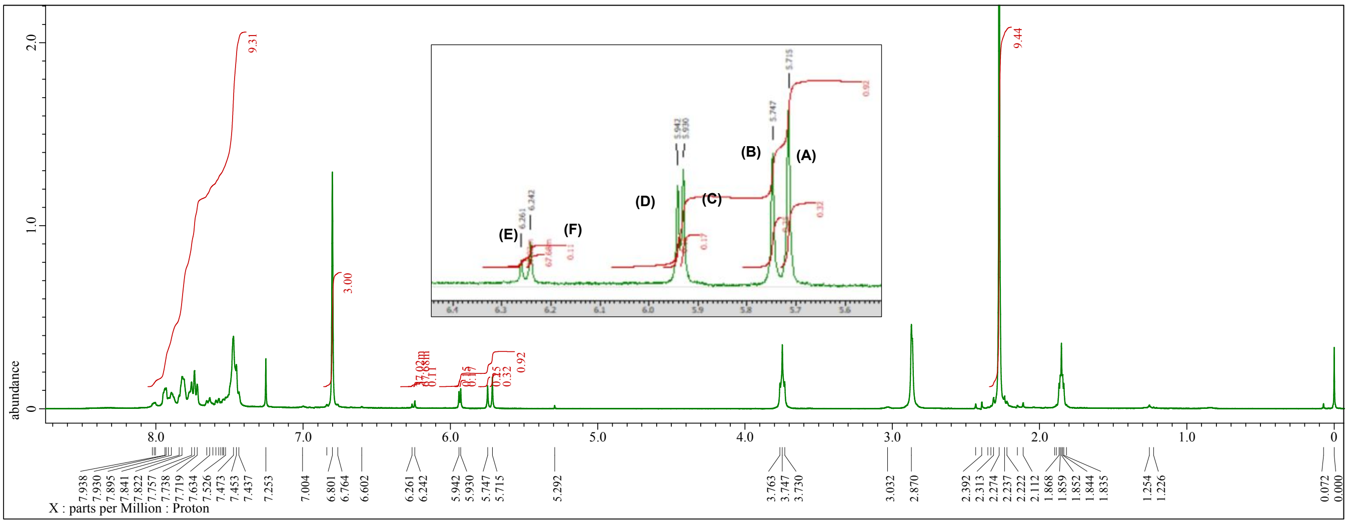


Used 1,3,5-trimethylbenzene as internal standard. Each NMR yield was calculated by benzyl position proton.

## 7. Transformation of haloimidation products

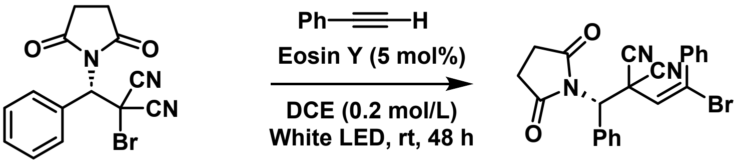


**2a**
**96% ee**

**4a**
**53% yield**
**94% ee**

To a stirred solution of **2a** (132.8 mg, 0.40 mmol) with ethynylbenzene (81.7 mg, 0.80 mmol) in 2.0 mL of DCE was added Na2-eosin Y (13.8 mg, 0.02 mmol). The stirred reaction mixture was irradiated by blue LED at room temperature for 48 h. Then the solvent was removed in vacuo, and the residue was purified by column chromatography on silica gel (hexane/ethyl acetate = 9/1 to 4/1) to give **4a** (93.8 mg, 53% yield). The enantiomeric excess of products was determined by chiral stationary phase HPLC using a Daicel Chiralcel AZ-3 column (hexane:2-propanol = 90/10, 1.0 mL/min, 254 nm); major enantiomer tr = 24.4 min, minor enantiomer tr = 38.7 min, 94% ee

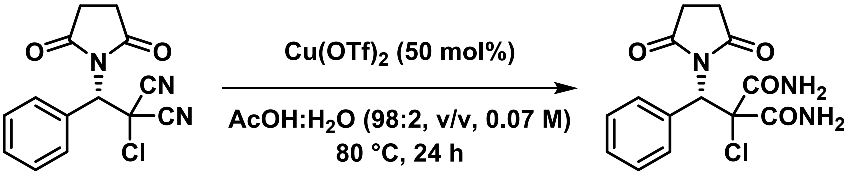


**3a**
**95% ee**

**4b**
**67% yield**
**92% ee**

A test tube, equipped with a stirring bar, was charged with **3a** (28.8 mg, 0.10 mmol), $Cu(OTf)_2$ (18.1 mg, 0.05 mmol), acetic acid (1.47 mL), and $H_2O$ (20 μL). The reaction mixture was heated to 80 °C and allowed to react for 24 h. The crude mixture

was poured into brine (3 mL), then extracted with EtOAc (3x3mL). The combined organic phase was washed with water (3x3 mL) and brine (3 mL), then dried over $Na_2SO_4$ and concentrated under reduced pressure. The product was purified by column chromatography on silica gel (hexane:EtOAc = 2/3 to EtOAc) to afford **4b** (21.7 mg, 22% yield). The enantiomeric excess of products was determined by chiral stationary phase HPLC using a Daicel Chiralcel AZ-3 column (hexane:EtOH = 10/90, 0.5 mL/min, 215 nm); major enantiomer tr = 21.9 min, minor enantiomer tr = 9.04 min, 92% ee; $[\alpha]_D^{23.9}$= -6.12 (c= 1.0, acetone, 92% ee)

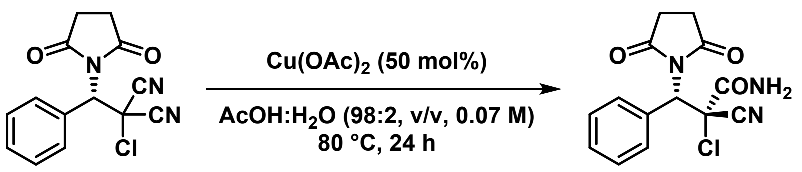


**3a**
**95% ee**

**4c**
**22% yield**
**95% ee**

A test tube, equipped with a stirring bar, was charged with **3a** (28.8 mg, 0.10 mmol), $Cu(OAc)_2$ (9.1 mg, 0.05 mmol), acetic acid (1.47 mL), and $H_2O$ (20 μL). The reaction mixture was heated to 80 °C and allowed to react for 24 h. The crude mixture was poured into brine (3 mL), then extracted with EtOAc (3x3mL). The combined organic phase was washed with water (3x3 mL) and brine (3 mL), then dried over $Na_2SO_4$ and concentrated under reduced pressure. The product was purified by column chromatography on silica gel (hexane:EtOAc = 3/2 to 2/3) to afford **4b** (6.77 mg, 22% yield). The enantiomeric excess of products was determined by chiral stationary phase HPLC using a Daicel Chiralcel IBN-3 column (hexane:2-propanol = 60/40, 0.5 mL/min, 254 nm); major enantiomer tr = 25.7 min, minor enantiomer tr = 28.2 min, 95% ee; $[\alpha]_D^{24.9}$= -81.2 (c= 0.5, acetone, 95% ee).

**8. 2 mmol scale experiment**

*N*-9-Anth-PyBidine (198.9 mg, 0.22 mmol), $Cu(OAc)_2$ (36.3 mg, 0.20 mmol) were added to a flask equipped with a stir bar and a three-way cock under Ar. Dichloromethane (20.0 ml) was added to the flask and the mixture was stirred over three hours. After removal the solvent under reduced pressure, benzylidenemalononitrile (308.3 mg, 2.0 mmol) and activated 4Å MS (2.0 g) was added. Then the flask and three-way cock was covered with aluminum foil. Dichloromethane (20.0 ml) and THF (3.2 ml) was added to the reaction vessel and stirred under Ar at -78 °C. After keeping 30 minutes -78 °C, NCS (293.8 mg, 2.2 mmol) was add. The reaction mixture was stirred 18 h, and solvent was removed under reduced pressure. The residue was purified silica-gel column chromatography to give **3a** (466.4 mg, 81% yield, 93% ee)
The enantiomeric excess of products was determined by chiral stationary phase HPLC using a Daicel Chiralpak AZ-3 column.

## 9. Analytical data for product

**(*S*)-2-bromo-2-((2,5-dioxopyrrolidin-1-yl)(phenyl)methyl)malononitrile (2a)**

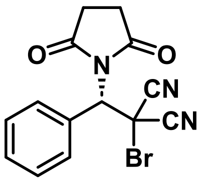

The reaction was performed following general procedure. The residue was purified by silica gel column chromatography (hexane/ethyl acetate = 9/1 to 2/1) to give a white amorphous solid (29.4 mg, 82% yield); $^1$H NMR (500 MHz, $CDCl_3$): δ 7.76-7.73 (m, 2H), 7.52-7.44 (m, 3H), 5.74 (s, 1H), 2.86 (s, 4H); $^{13}$C NMR(125 MHz, $CDCl_3$): δ 175.8, 131.0, 130.5, 130.0, 129.3, 111.2, 111.0, 62.7, 28.0, 27.5; HRMS calcd $C_{14}H_{11}O_2N_3Br$ $(M+H)^+$: 332.0029, found: *m/z* 332.0031; Enantiomeric excess was determined by HPLC with a Chiralcel IC-3 column (hexane:2-propanol = 90/10, 0.5 mL/min, 254 nm); major enantiomer tr = 44.1 min, minor enantiomer tr = 35.1 min, 99% ee; $[\alpha]_D^{24.5}$= -44.6 (c= 0.5, $CHCl_3$, 99% ee)

**(*S*)-2-bromo-2-((2,5-dioxopyrrolidin-1-yl)(o-tolyl)methyl)malononitrile (2b)**

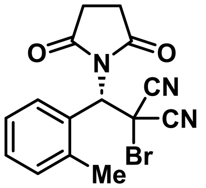

The reaction was performed following general procedure. The residue was purified by silica gel column chromatography (hexane/ethyl acetate = 9/1 to 2/1) to give a white amorphous solid (28.5 mg, 81% yield); $^1$H NMR (500 MHz, $CDCl_3$): δ 8.17 (dd, *J*=7.9 and 1.3 Hz, 1H), 7.37 (td, *J*=7.4, 1.4 Hz, 1H), 7.32 (td, *J*= 7.6 and 1.3 Hz, 1H), 7.28-7.26 (m, 1H), 6.13 (s, 1H), 2.84 (s, 4H), 2.61 (s, 3H); $^{13}$C NMR(125 MHz, $CDCl_3$): δ 176.1, 138.3, 131.6, 130.9, 129.9, 129.8, 127.4, 111.5, 111.4, 57.6, 28.1, 27.7, 20.2; HRMS calcd $C_{15}H_{13}O_2N_3Br$ $(M+H)^+$: 346.0186, found: *m/z* 346.0187; Enantiomeric excess was determined by HPLC with a Chiralcel IC-3 column (hexane:2-propanol = 90/10, 1.0 mL/min, 254 nm); major enantiomer tr = 17.5 min, minor enantiomer tr = 24.6 min 95% ee; $[\alpha]_D^{25.0}$= -19.5 (c= 0.5, $CHCl_3$, 95% ee)

**(*S*)-2-bromo-2-((2,5-dioxopyrrolidin-1-yl)(m-tolyl)methyl)malononitrile (2c)**

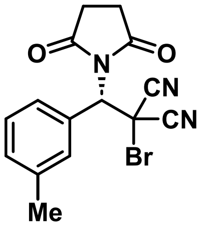

The reaction was performed following general procedure. The residue was purified by silica gel column chromatography (hexane/ethyl acetate = 9/1 to 2/1) to give a pale yellow amorphous solid (27.0 mg, 76% yield); $^1$H NMR (500 MHz, $CDCl_3$): δ 7.56 (d, *J*=7.7 Hz, 1H), 7.50 (s, 1H), 7.34 (t, *J*= 7.6 Hz, 1H), 7.30 (d, *J*=7.7 Hz, 1H), 5.70 (s, 1H), 2.86 (s, 4H), 2.39 (s, 3H); $^{13}$C NMR(125 MHz, $CDCl_3$): δ176.1, 138.3, 131.6, 130.9, 129.9, 129.8, 127.4, 111.5, 111.4, 57.6, 28.1, 27.7, 20.3; HRMS calcd $C_{15}H_{13}O_2N_3Br$ $(M+H)^+$: 346.0186, found: *m/z* 346.0186; Enantiomeric excess was determined by HPLC with a Chiralcel IC-3 column (hexane:2-propanol = 90/10, 1.0 mL/min, 254 nm); major enantiomer tr = 15.0 min, minor enantiomer tr = 18.9 min 99% ee; $[\alpha]_D^{24.7}$= -35.8 (c= 0.5, $CHCl_3$, 99% ee)

**(*S*)-2-bromo-2-((2,5-dioxopyrrolidin-1-yl)(p-tolyl)methyl)malononitrile (2d)**

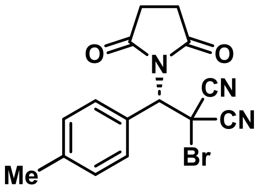

The reaction was performed following general procedure. The residue was purified by silica gel column chromatography (hexane/ethyl acetate = 9/1 to 2/1) to give a yellow amorphous solid (26.5 mg, 76% yield); $^1$H NMR (500 MHz, $CDCl_3$): δ 7.62 (d, *J*=8.3 Hz, 2H), 7.24 (d, *J*=7.7 Hz, 2H), 5.70 (s, 1H), 2.84 (s, 4H), 2.38 (s, 3H); $^{13}$C NMR(125 MHz, $CDCl_3$): δ175.9, 141.5, 130.1, 130.0, 127.6, 111.4, 111.2, 62.6, 28.1, 27.9, 21.4; HRMS calcd $C_{15}H_{13}O_2N_3Br$ $(M+H)^+$: 346.0186, found: *m/z* 346.0185; Enantiomeric excess was determined by HPLC with a Chiralcel OD-H column (hexane:2-propanol = 80/20, 1.0 mL/min, 254 nm); major enantiomer tr = 16.6 min, minor enantiomer tr = 25.6 min 91% ee; $[\alpha]_D^{25.4}$= -34.9 (c= 0.5, $CHCl_3$, 91% ee)

**(*S*)-2-bromo-2-((2,5-dioxopyrrolidin-1-yl)(2-fluorophenyl)methyl)malononitrile (2e)**

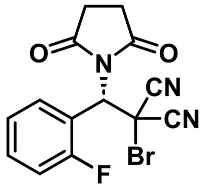

The reaction was performed following general procedure. The residue was purified by silica gel column chromatography (hexane/ethyl acetate = 9/1 to 2/1) to give a white amorphous solid (31.4 mg, 89% yield); $^1$H NMR (500 MHz, $CDCl_3$): δ 8.09 (td, *J*=7.6, 1.6 Hz, 1H), 7.50-7.46 (m, 1H), 7.33-7.25 (m, 1H), 7.21-7.15 (m, 1H), 6.27 (d, *J*=1.4 Hz, 1H), 2.84 (s, 4H); $^{13}$C NMR(125 MHz, $CDCl_3$): δ 175.6, 160.2, 132.9, 130.7, 125.1, 118.1, 116.1, 110.9, 110.8, 53.9, 27.9, 26.5; HRMS calcd $C_{14}H_{10}O_2N_3BrF$ $(M+H)^+$: 339.9935, found: *m/z* 339.9939; Enantiomeric excess was determined by HPLC with a Chiralcel IC-3 column (hexane:2-propanol = 90/10, 1.0 mL/min, 254 nm); major enantiomer tr = 23.1 min, minor enantiomer tr = 18.2 min 97% ee; $[\alpha]_D^{24.8}$= -22.8 (c= 0.5, $CHCl_3$, 97% ee)

**(*S*)-2-bromo-2-((2,5-dioxopyrrolidin-1-yl)(3-fluorophenyl)methyl)malononitrile (2f)**

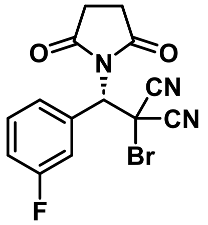

The reaction was performed following general procedure. The residue was purified by silica gel column chromatography (hexane/ethyl acetate = 9/1 to 2/1) to give a white amorphous solid (18.6 mg, 53% yield); 1H NMR (500 MHz, $CDCl_3$): δ 7.56-7.49 (m, 2H), 7.47-7.43 (m, 1H), 7.21 (tq, *J* = 8.3 and 1.1 Hz, 1H), 5.74 (s, 1H), 2.89 (s, 4H); $^{13}$C NMR(125 MHz, $CDCl_3$): δ 175.7, 162.6, 132.4, 131.0, 125.9, 118.2, 117.2, 111.0, 110.7, 62.0, 28.0, 27.1; HRMS calcd $C_{14}H_{10}O_2N_3BrF$ $(M+H)^+$: 349.9935, found: *m/z* 349.9943; Enantiomeric excess was determined by HPLC with a Chiralcel AZ-3 column (hexane:2-propanol = 90/10, 1.0 mL/min, 254 nm); major enantiomer tr = 25.2 min, minor enantiomer tr = 22.0 min 97% ee; $[\alpha]_D^{25.3}$= -38.6 (c= 0.5, $CHCl_3$, 97% ee)

**(*S*)-2-bromo-2-((2,5-dioxopyrrolidin-1-yl)(4-fluorophenyl)methyl)malononitrile (2g)**

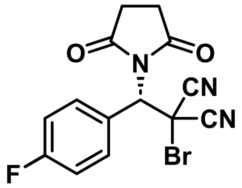

The reaction was performed following general procedure. The residue was purified by silica gel column chromatography (hexane/ethyl acetate = 9/1 to 2/1) to give a white amorphous solid (26.0 mg, 76% yield); $^1$H NMR (500 MHz, $CDCl_3$): δ 7.82-7.74 (m, 2H), 7.21-7.12 (m, 2H), 5.74 (s,1H), 2.89 (s, 4H); $^{13}$C NMR(125 MHz, $CDCl_3$): δ 175.7, 164.0, 132.3, 126.4, 116.5, 111.1, 110.1, 61.9, 28.0, 27.5; HRMS calcd $C_{14}H_{10}O_2N_3BrF$ $(M+H)^+$: 349.9935, found: *m/z* 349.9938; Enantiomeric excess was determined by HPLC with a Chiralcel AZ-3 column (hexane:2-propanol = 90/10, 1.0 mL/min, 254 nm); major enantiomer tr = 31.4 min, minor enantiomer tr = 27.1 min 97% ee; $[\alpha]_D^{25.5}$= -75.8 (c= 0.5, $CHCl_3$, 97% ee)

**(*S*)-2-bromo-2-((2-chlorophenyl)(2,5-dioxopyrrolidin-1-yl)methyl)malononitrile (2h)**

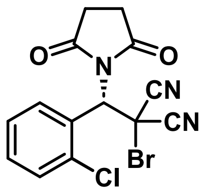

The reaction was performed following general procedure. The residue was purified by silica gel column chromatography (hexane/ethyl acetate = 9/1 to 2/1) to give a white amorphous solid (25.9 mg, 69% yield); $^1$H NMR (500 MHz, $CDCl_3$): δ 8.31-8.26 (m, 1H), 7.53-7.47 (m, 1H), 7.45-7.37 (m, 2H), 6.53 (s, 1H), 2.85 (s, 4H); $^{13}$C NMR (125 MHz, $CDCl_3$): δ 175.7, 135.2, 132.1, 131.4, 130.6, 128.7, 127.9, 111.2, 111.0, 57.0, 28.1, 26.5; HRMS calcd $C_{14}H_{10}O_2N_3BrCl$ $(M+H)^+$: 365.9639, found: *m/z* 365.9640; Enantiomeric excess was determined by HPLC with a Chiralcel IC-3 column (hexane:2-propanol = 90/10, 1.0 mL/min, 254 nm); major enantiomer tr = 22.5 min, minor enantiomer tr = 18.5 min 71% ee; $[\alpha]_D^{25.6}$= -9.3 (c= 0.5, $CHCl_3$, 71% ee)

**(*S*)-2-bromo-2-((3-chlorophenyl)(2,5-dioxopyrrolidin-1-yl)methyl)malononitrile (2i)**

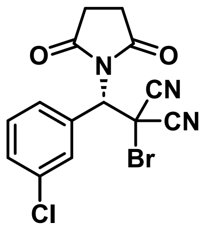

The reaction was performed following general procedure. The residue was purified by silica gel column chromatography (hexane/ethyl acetate = 9/1 to 2/1) to give a white amorphous solid (30.7 mg, 84% yield); $^1$H NMR (500 MHz, $CDCl_3$): δ 7.57-7.42 (m, 3H), 7.23-7.12 (m, 1H), 5.74 (s, 1H), 2.89 (s, 4H); $^{13}$C NMR(125 MHz, $CDCl_3$): δ 175.6, 135.2, 132.1, 131.3, 130.7, 130.2, 128.1, 110.1, 110.7, 62.0, 28.0, 27.1;

HRMS calcd $C_{14}H_{10}O_2N_3BrCl$ $(M+H)^+$: 365.9639, found: m/z 365.9640; Enantiomeric excess was determined by HPLC with a Chiralcel AZ-3 column (hexane:2-propanol = 90/10, 1.0 mL/min, 254 nm); major enantiomer tr = 21.9 min, minor enantiomer tr = 25.1 min 88% ee; $[\alpha]_D^{25.5}$= -40.4 (c= 0.5, $CHCl_3$, 88% ee)

**(*S*)-2-bromo-2-((4-chlorophenyl)(2,5-dioxopyrrolidin-1-yl)methyl)malononitrile (2j)**

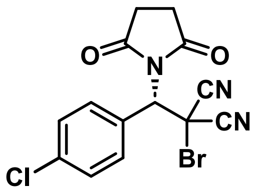

The reaction was performed following general procedure. The residue was purified by silica gel column chromatography (hexane/ethyl acetate = 9/1 to 2/1) to give a white amorphous solid (32.4 mg, 88% yield); $^1H$ NMR (400 MHz, $CDCl_3$): δ 7.69 (dt, *J*=9.0 and 2.4 Hz, 2H), 7.43 (dt, *J*=8.8 and 2.5 Hz, 2H), 5.72 (s, 1H), 2.87 (s, 4H); $^{13}C$ NMR (125 MHz, $CDCl_3$): δ 175.7, 137.3, 131.4, 129.5, 128.8, 111.0, 110.7, 62.0, 28.0, 27.2; HRMS calcd $C_{14}H_{11}O_2N_3Br$ $(M+H)^+$: 365.9639, found: *m/z* 365.9640; Enantiomeric excess was determined by HPLC with a Chiralcel IBN-3 column (hexane:2-propanol = 80/20, 1.0 mL/min, 254 nm); major enantiomer tr = 20.3 min, minor enantiomer tr = 25.9 min 97% ee; $[\alpha]_D^{25.7}$= -29.6 (c= 0.5, $CHCl_3$, 97% ee)

**(*S*)-2-bromo-2-((2-bromophenyl)(2,5-dioxopyrrolidin-1-yl)methyl)malononitrile (2k)**

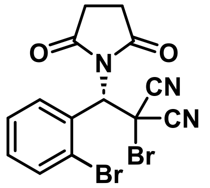

The reaction was performed following general procedure. The residue was purified by silica gel column chromatography (hexane/ethyl acetate = 9/1 to 2/1) to give a pale yellow amorphous solid (43.1 mg, 92% yield); $^1H$ NMR (500 MHz, $CDCl_3$): δ 8.32 (dd, *J*=8.0 and 1.7 Hz, 1H), 7.69 (dd, *J*=8.0, 1.4 Hz, 1H), 7.46 (td, *J*=7.7, 1.1 Hz, 1H), 7.36-7.32 (m, 1H), 6.53 (s, 1H), 2.85 (s, 4H); $^{13}C$ NMR(125 MHz, $CDCl_3$): δ 175.5, 134.0, 132.1, 131.5, 130.3, 128.3, 125.6, 111.0, 111.0, 59.4, 28.0, 26.5; HRMS calcd $C_{14}H_{10}O_2N_3Br_2$ $(M+H)^+$: 409.9134, found: *m/z* 409.9134; Enantiomeric excess was determined by HPLC with a Chiralcel IC-3 column (hexane:2-propanol = 90/10, 1.0 mL/min, 254 nm); major enantiomer tr = 22.5 min, minor enantiomer tr = 18.7 min 37% ee; $[\alpha]_D^{24.9}$= -7.2 (c= 0.5, $CHCl_3$, 37% ee)

**(*S*)-2-bromo-2-((3-bromophenyl)(2,5-dioxopyrrolidin-1-yl)methyl)malononitrile (2l)**

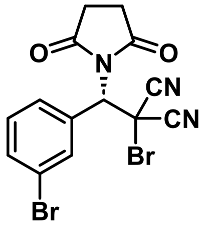

The reaction was performed following general procedure. The residue was purified by silica gel column chromatography (hexane/ethyl acetate = 9/1 to 2/1) to give a white amorphous solid (34.0 mg, 79% yield); $^1H$ NMR (400 MHz, $CDCl_3$): δ 7.88-7.82 (m, 1H), 7.79-7.69 (m, 1H), 7.65-7.60 (m, 1H), 7.35 (t, *J*=8.0 Hz, 1H), 5.69 (s, 1H), 2.88 (s, 4H); $^{13}C$ NMR (125 MHz, $CDCl_3$): δ 175.7, 134.1, 132.9, 132.3, 130.8, 128.5, 123.0, 110.8, 110.7, 61.8, 28.0, 27.0; HRMS calcd $C_{14}H_{10}O_2N_3Br_2$ $(M+H)^+$: 409.9134, found: *m/z* 409.9134; Enantiomeric excess was determined by HPLC with a Chiralcel IBN-3 column (hexane:2-propanol = 80/20, 0.5 mL/min, 254 nm); major enantiomer tr = 21.7 min, minor enantiomer tr = 30.0 min 95% ee; $[\alpha]_D^{25.6}$= -34.0 (c= 0.5, $CHCl_3$, 95% ee)

**(*S*)-2-bromo-2-((4-bromophenyl)(2,5-dioxopyrrolidin-1-yl)methyl)malononitrile (2m)**

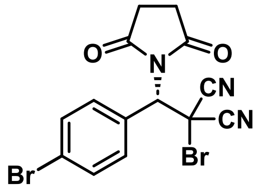

The reaction was performed following general procedure. The residue was purified by silica gel column chromatography (hexane/ethyl acetate = 9/1 to 2/1) to give a white amorphous solid (35.1 mg, 84% yield); $^1H$ NMR (400 MHz, $CDCl_3$): δ 7.69-7.53 (m, 4H), 5.70 (s, 1H), 2.87 (s, 4H); $^{13}C$ NMR(125 MHz, $CDCl_3$): δ 175.9, 132.7, 131.8, 129.5, 125.8, 111.2, 110.9, 62.2, 28.2, 27.2; HRMS calcd $C_{14}H_{10}O_2N_3Br_2$ $(M+H)^+$: 409.9134, found: *m/z* 409.9132; Enantiomeric excess was determined by HPLC with a Chiralcel OZ-3 column (hexane:2-propanol = 80/20, 1.0 mL/min, 254 nm); major enantiomer tr = 15.0 min, minor enantiomer tr = 23.3 min 99% ee; $[\alpha]_D^{25.0}$= -24.3 (c= 0.5, $CHCl_3$, 99% ee)

**(*S*)-2-bromo-2-((2,5-dioxopyrrolidin-1-yl)(3-(trifluoromethyl)phenyl)methyl)malononitrile (2n)**

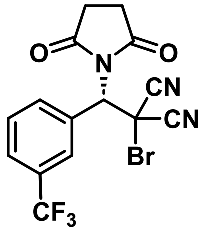


The reaction was performed following general procedure. The residue was purified by silica gel column chromatography (hexane/ethyl acetate = 9/1 to 2/1) to give a white amorphous solid (29.5 mg, 76% yield); $^1$H NMR (500 MHz, $CDCl_3$): δ 8.03 (d, *J*=8.0 Hz, 1H), 7.96 (s, 1H), 7.78 (d, *J*=7.7 Hz, 1H), 7.63 (t, *J*=7.9 Hz, 1H), 5.82 (s, 1H), 2.90 (s, 4H); $^{13}$C NMR(125 MHz, $CDCl_3$): δ 175.7, 133.3, 131.7, 131.4, 130.1, 127.9, 127.2, 123.3, 110.8, 110.6, 62.2, 28.0, 27.0; HRMS calcd $C_{15}H_{10}O_2N_3BrF_3$ $(M+H)^+$: 399.9903, found: *m/z* 399.9903; Enantiomeric excess was determined by HPLC with a Chiralcel IBN-3 column (hexane:2-propanol = 80/20, 1.0 mL/min, 254 nm); major enantiomer tr = 15.9 min, minor enantiomer tr = 23.8 min96% ee; $[\alpha]_D^{25.4}$= -30.1 (c= 0.5, $CHCl_3$, 96% ee)

**(*S*)-2-bromo-2-((2,5-dioxopyrrolidin-1-yl)(4-(trifluoromethyl)phenyl)methyl)malononitrile (2o)**

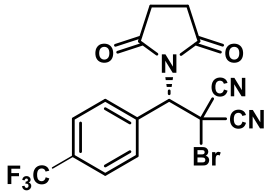


The reaction was performed following general procedure. The residue was purified by silica gel column chromatography (hexane/ethyl acetate = 9/1 to 2/1) to give a white amorphous solid (29.8 mg, 74% yield); $^1$H NMR (500 MHz, $CDCl_3$): δ 7.90 (d, *J*=8.3 Hz, 2H), 7.73 (d, *J*=8.3 Hz, 2H), 5.81 (s, 1H), 2.89 (s, 4H); $^{13}$C NMR (125 MHz, $CDCl_3$): δ 175.7, 134.2, 133.3, 130.6, 126.3, 123.3, 110.9, 110.6, 62.0, 28.0, 26.8; HRMS calcd $C_{15}H_{10}O_2N_3BrF_3$ $(M+H)^+$: 399.9906, found: *m/z* 399.9903; Enantiomeric excess was determined by HPLC with a Chiralcel OZ-3 column (hexane:2-propanol = 80/20, 1.0 mL/min, 254 nm); major enantiomer tr = 10.0 min, minor enantiomer tr = 22.3 min98% ee; $[\alpha]_D^{25.0}$= -39.1 (c= 0.1, $CHCl_3$, 98% ee)

**(*S*)-2-bromo-2-((2,5-dioxopyrrolidin-1-yl)(naphthalen-2-yl)methyl)malononitrile (2p)**

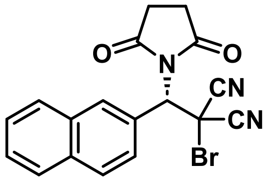


The reaction was performed following general procedure. The residue was purified by silica gel column chromatography (hexane/ethyl acetate = 9/1 to 2/1) to give a white amorphous solid (27.2 mg, 79% yield); $^1$H NMR (500 MHz, CDCl3): δ 8.22 (d, *J*=1.7 Hz, 1H), 7.96-7.82 (m, 4H), 7.60-7.54 (m, 2H), 5.91 (s, 1H), 2.87 (s, 4H); $^{13}$C NMR(125 MHz, $CDCl_3$): δ 175.8, 134.0, 132.8, 130.7, 129.5, 128.6, 127.9, 127.7, 127.1, 125.9, 111.3, 111.0, 63.0, 28.1, 27.6; HRMS calcd $C_{18}H_{13}O_2N_3Br$ $(M+H)^+$: 382.0186, found: *m/z* 382.0180; Enantiomeric excess was determined by HPLC with a Chiralcel IA column (hexane:2-propanol = 90/10, 1.0 mL/min, 254 nm); major enantiomer tr = 22.8 min, minor enantiomer tr = 27.4 min 99% ee; $[\alpha]_D^{25.0}$= -36.5 (c= 0.5, $CHCl_3$, 99% ee)

**methyl (*S*)-4-(2-bromo-2,2-dicyano-1-(2,5-dioxopyrrolidin-1-yl)ethyl)benzoate (2q)**

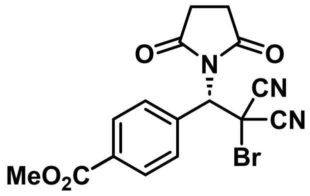


The reaction was performed following general procedure. The residue was purified by silica gel column chromatography (hexane/ethyl acetate = 9/1 to 2/1 ) to give a pale yellow amorphous solid (31.4 mg, 78% yield); $^1$H NMR (500 MHz, $CDCl_3$): δ 8.11 (dt, *J*=8.6 and 1.9 Hz, 2H), 7.83 (dt, *J*=8.5 and 1.9 Hz, 2H), 5.80 (s, 1H), 3.94 (s, 3H), 2.90 (s, 4H); $^{13}$C NMR (125 MHz, $CDCl_3$): δ 175.7, 165.9, 134.8, 132.5, 130.4, 130.1, 110.9, 110.7, 62.2, 52.5, 28.0, 26.9; HRMS calcd $C_{16}H_{13}O_4N_3Br$ $(M+H)^+$: 390.0084, found: *m/z* 390.0081; Enantiomeric excess was determined by HPLC with a Chiralcel AZ-3 column (hexane:2-propanol = 90/10, 1.0 mL/min, 254 nm); major enantiomer tr = 27.8 min, minor enantiomer tr = 32.8 min 91% ee; $[\alpha]_D^{25.6}$= -30.4 (c= 0.5, $CHCl_3$, 91% ee)

**(*S*)-2-bromo-2-((2,5-dioxopyrrolidin-1-yl)(4-methoxyphenyl)methyl)malononitrile (2r)**

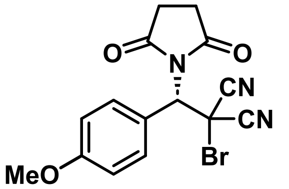


The reaction was performed following general procedure. The residue was purified by silica gel column chromatography (hexane/ethyl acetate = 9/1 to 2/1) to give a white amorphous solid (28.4 mg, 78% yield); $^{1}$H NMR (500 MHz, $CDCl_3$): δ 7.69-7.66 (m, 2H), 6.96-6.93 (m, 2H), 5.70 (s, 1H), 3.83 (s, 3H), 2.85 (s, 4H); $^{13}$C NMR(125 MHz, $CDCl_3$): δ 175.8, 161.4, 131.6, 122.3, 114.6, 111.3, 111.0, 62.3, 55.4, 28.0; HRMS calcd $C_{15}H_{13}O_3N_3Br$ $(M+H)^+$: 362.0135, found: *m/z* 362.0134; Enantiomeric excess was determined by HPLC with a Chiralcel IBN-3 column (hexane:2-propanol = 80/20, 1.0 mL/min, 254 nm); major enantiomer tr = 27.7 min, minor enantiomer tr = 41.2 min 86% ee; $[\alpha]_D^{25.3}$= -27.1 (c= 0.5, $CHCl_3$, 86% ee)

**(*S*)-2-bromo-2-((2,5-dioxopyrrolidin-1-yl)(thiophen-2-yl)methyl)malononitrile (2s)**

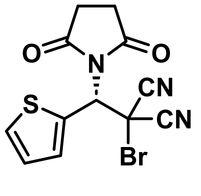


The reaction was performed following general procedure. The residue was purified by silica gel column chromatography (hexane/ethyl acetate = 9/1 to 2/1 ) to give a green amorphous solid (6.1 mg, 18% yield); $^{1}$H NMR (500 MHz, $CDCl_3$): δ 7.60 (dq, *J*=3.7 and 0.6 Hz, 1H), 7.48 (dd, *J*=5.2 and 0.9 Hz, 1H), 7.10 (dd, *J*=5.3 and 3.6 Hz, 1H), 6.07 (s, 1H), 2.88 (s, 4H); $^{13}$C NMR(125 MHz, $CDCl_3$): δ 175.1, 132.3, 129.6, 129.5, 127.4, 111.1, 110.7, 57.5, 28.1, 27.9; HRMS calcd $C_{12}H_9O_2N_3BrS$ $(M+H)^+$: 337.9593, found: *m/z* 337.9593; Enantiomeric excess was determined by HPLC with a Chiralcel IBN-3 column (hexane:2-propanol = 80/20, 1.0 mL/min, 254 nm); major enantiomer tr = 22.3 min, minor enantiomer tr = 39.2 min 78% ee; $[\alpha]_D^{25.2}$= -44.1 (c= 0.1, $CHCl_3$, 78% ee)

**(*S*)-2-bromo-2-(cyclohexyl(2,5-dioxopyrrolidin-1-yl)methyl)malononitrile (2t)**

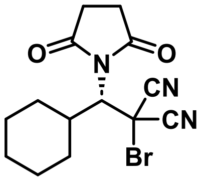


The reaction was performed following general procedure. The residue was purified by silica gel column chromatography (hexane/ethyl acetate = 9/1 to 2/1) to give a white amorphous solid (23.0 mg, 66% yield); $^{1}$H NMR (400 MHz, $CDCl_3$): δ 4.50 (d, *J*=10.5 Hz, 1H), 3.00-2.63 (m, 5H), 2.31-2.24 (m, 1H), 1.93-0.96 (m, 9H); $^{13}$C NMR (125 MHz, $CDCl_3$): δ 176.2, 175.3, 111.7, 110.7, 61.7, 37.3, 31.0, 30.2, 28.2, 27.3, 26.5, 25.3, 25.2; HRMS calcd $C_{14}H_{17}O_2N_3Br$ $(M+H)^+$: 338.0499, found: *m/z* 338.0497; Enantiomeric excess was determined by HPLC with a Chiralcel IBN-3 column (hexane:2-propanol = 70/30, 1.0 mL/min, 254 nm); major enantiomer tr = 8.2 min, minor enantiomer tr = 17.4 min 97% ee; $[\alpha]_D^{25.4}$= +19.5 (c= 0.5, $CHCl_3$, 97% ee)

**(*S*)-2-bromo-2-(cyclopentyl(2,5-dioxopyrrolidin-1-yl)methyl)malononitrile (2u)**

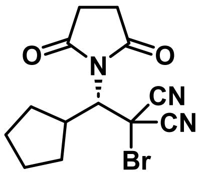


The reaction was performed following general procedure. The residue was purified by silica gel column chromatography (hexane/ethyl acetate = 9/1 to 2/1) to give a white amorphous solid (24.5 mg, 74% yield); $^{1}$H NMR (500 MHz, $CDCl_3$): δ 4.56 (d, *J* = 10.9 Hz, 1H), 3.15-3.03 (m, 1H), 2.98-2.72 (m, 4H), 2.23-2.17 (m, 1H), 1.91-1.84 (m, 1H), 1.80-1.48 (m, 5H), 1.33-1.18 (m, 1H); $^{13}$C NMR(125 MHz, $CDCl_3$): δ 176.2, 175.1, 111.6, 110.6, 77.3, 77.2, 77.0, 76.8, 61.5, 40.1, 31.2, 31.1, 28.1, 27.2, 26.9, 25.8, 23.1; HRMS calcd $C_{13}H_{15}O_2N_3Br$ $(M+H)^+$: 332.0342, found: *m/z* 332.0341; Enantiomeric excess was determined by HPLC with a Chiralcel IBN-3 column (hexane:2-propanol = 70/30, 1.0 mL/min, 254 nm); major enantiomer tr = 9.5 min, minor enantiomer tr = 20.8 min 93% ee; $[\alpha]_D^{25.1}$= +16.9 (c= 0.5, $CHCl_3$, 93% ee)

**(*S*)-2-chloro-2-((2,5-dioxopyrrolidin-1-yl)(phenyl)methyl)malononitrile (3a)**

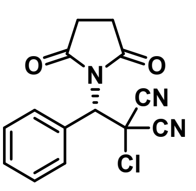


The reaction was performed following general procedure. The residue was purified by silica gel column chromatography (hexane/ethyl acetate = 4/1 ) to give a pale yellow amorphous solid (23.8 mg, 83% yield); $^{1}$H NMR (500 MHz, $CDCl_3$): δ 7.73 (d, *J*=7.16 Hz, 2H), 7.51-7.44 (m, 3H), 5.71 (s, 1H), 2.87 (s, 4H); $^{13}$C NMR(125 MHz, $CDCl_3$):δ 175.8, 131.1, 130.3, 130.0, 129.4, 110.9, 110.7, 63.0, 45.9, 28.0; HRMS calcd

$C_{14}H_{10}O_2N_3ClNa$ $(M+Na)^+$: 310.0354, found: *m/z* 310.0348; Enantiomeric excess was determined by HPLC with a Chiralcel AZ-3 column (hexane:2-propanol = 90/10, 1.0 mL/min, 254 nm); major enantiomer tr = 24.4 min, minor enantiomer tr = 19.9 min, 94% ee; $[\alpha]_D^{22.0}$= -56.0 (c= 0.5, $CHCl_3$, 94% ee)

**(*S*)-2-chloro-2-((2,5-dioxopyrrolidin-1-yl)(o-tolyl)methyl)malononitrile (3b)**

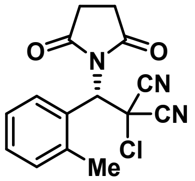

The reaction was performed following general procedure. The residue was purified by silica gel column chromatography (hexane/ethyl acetate = 4/1) to give a yellow amorphous solid (22.0 mg, 74% yield); $^1$H NMR (500 MHz, $CDCl_3$): δ 7.54 (d, *J*=8.02 Hz, 1H), 7.48 (s, 1H), 7.36-7.29 (m, 2H), 5.67(s, 1H), 2.87 (s, 4H), 2.39 (s, 3H); $^{13}$C NMR (125 MHz, $CDCl_3$):δ 175.8, 139.3, 131.8, 130.5, 130.2, 129.3, 126.9, 110.9, 110.7, 62.9, 45.9, 28.0, 21.4; HRMS calcd $C_{15}H_{12}O_2N_3ClNa$ $(M+Na)^+$: 324.0510, found: *m/z* 324.0504; Enantiomeric excess was determined by HPLC with a Chiralcel IBN-3 column (hexane:2-propanol = 90/10, 1.0 mL/min, 254 nm); major enantiomer tr = 20.2 min, minor enantiomer tr = 30.4 min, 64% ee; $[\alpha]_D^{22.3}$= -31.3 (c= 0.5, $CHCl_3$, 64% ee)

**(*S*)-2-chloro-2-((2,5-dioxopyrrolidin-1-yl)(m-tolyl)methyl)malononitrile (3c)**

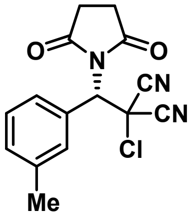

The reaction was performed following general procedure. The residue was purified by silica gel column chromatography (hexane/ethyl acetate = 4/1 ) to give a yellow amorphous solid (25.3 mg, 84% yield); $^1$H NMR (500 MHz, $CDCl_3$): δ 7.55 (d, *J*=7.45 Hz, 1H), 7.48 (s, 1H), 7.36-7.29 (m, 2H), 5.67 (s, 1H), 2.87 (s, 4H), 2.39(s, 3H); $^{13}$C NMR(125 MHz, $CDCl_3$): δ 175.8, 139.3, 131.8, 130.5, 130.2, 129.3, 126.9, 110.9, 110.7, 62.9, 45.9, 28.0, 21.4; HRMS calcd $C_{15}H_{12}O_2N_3ClNa$ $(M+Na)^+$: 324.0510, found: *m/z* 324.0505; Enantiomeric excess was determined by HPLC with a Chiralcel IBN-3 column (hexane:2-propanol = 90/10, 1.0 mL/min, 254 nm); major enantiomer tr = 20.9 min, minor enantiomer tr = 26.2 min, 86% ee; $[\alpha]_D^{22.5}$= -95.8 (c= 0.5, $CHCl_3$, 86% ee)

**(*S*)-2-chloro-2-((2,5-dioxopyrrolidin-1-yl)(p-tolyl)methyl)malononitrile (3d)**

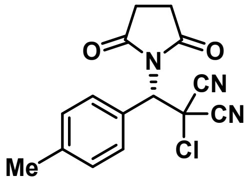

The reaction was performed following general procedure. The residue was purified by silica gel column chromatography (hexane/ethyl acetate = 4/1) to give a yellow amorphous solid (24.4 mg, 81% yield); $^1$H NMR (500 MHz, $CDCl_3$): δ 7.61 (d, *J*=8.31 Hz, 2H), 7.26-7.25 (m, 2H), 5.68 (s, 1H), 2.86 (s, 4H), 2.38 (s, 3H); $^{13}$C NMR (125 MHz, $CDCl_3$): δ 175.8, 141.4, 130.0, 129.8, 127.3, 111.0, 110.7, 62.7, 46.0, 28.0, 21.3; HRMS calcd $C_{15}H_{12}O_2N_3ClNa$ $(M+Na)^+$: 324.0510, found: *m/z* 324.0505; Enantiomeric excess was determined by HPLC with a Chiralcel IBN-3 column (hexane:2-propanol = 90/10, 1.0 mL/min, 254 nm); major enantiomer tr = 23.3 min, minor enantiomer tr = 30.5 min, 89% ee; $[\alpha]_D^{22.8}$= -64.4 (c= 0.5, $CHCl_3$, 89% ee)

**(*S*)-2-chloro-2-((2,5-dioxopyrrolidin-1-yl)(2-fluorophenyl)methyl)malononitrile (3e)**

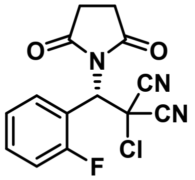

The reaction was performed following general procedure. The residue was purified by silica gel column chromatography (hexane/ethyl acetate = 4/1) to give a pale yellow amorphous solid (29.0 mg, 95% yield); $^1$H NMR (500 MHz, $CDCl_3$): δ 8.09 (dt, *J*=7.59 and 1.72 Hz, 1H), 7.52-7.47 (m, 1H), 7.29 (dt, *J*=7.73 and 0.86 Hz, 1H), 7.18 (dt, *J*=9.16 and 1.15 Hz,1H), 6.28 (d, *J*=1.15, 1H), 2.87 (s, 4H); $^{13}$C NMR (125 MHz, $CDCl_3$):δ 175.6, 161.4, 159.4, 133.0, 132.9, 130.7, 125.2, 117.9, 117.8, 116.2, 116.1, 110.6, 110.6, 54.3, 54.3, 45.4, 28.0; HRMS calcd $C_{14}H_9O_2N_3ClFNNa$ $(M+Na)^+$: 328.0260, found: *m/z* 328.0263; Enantiomeric excess was determined by HPLC with a Chiralcel IBN-3 column (hexane:2-propanol = 90/10, 1.0 mL/min, 254 nm); major enantiomer tr = 27.9 min, minor enantiomer tr = 37.1 min, 88% ee; $[\alpha]_D^{22.6}$= -34.4 (c= 0.5, $CHCl_3$, 88% ee)

**(*S*)-2-chloro-2-((2,5-dioxopyrrolidin-1-yl)(2-fluorophenyl)methyl)malononitrile (3f)**

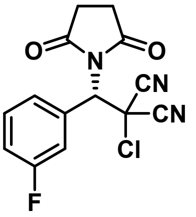

The reaction was performed following general procedure. The residue was purified by silica gel column chromatography (hexane/ethyl acetate = 4/1) to give a yellow amorphous solid (24.5 mg, 80% yield); $^{1}$H NMR (500 MHz, $CDCl_3$): δ 7.52-7.43 (m, 3H), 7.23-7.19 (m, 1H), 5.71 (s, 1H), 2.90 (s, 4H); $^{13}$C NMR(125 MHz, $CDCl_3$): δ 175.7, 163.7, 161.7, 132.2, 132.1, 131.1, 131.1, 125.8, 125.8, 118.4, 118.2, 117.3, 117.1, 110.6, 110.4, 62.3, 45.6, 28.0; HRMS calcd $C_{14}H_9O_2N_3ClFNNa$ $(M+Na)^+$: 328.0260, found: *m/z* 328.0263; Enantiomeric excess was determined by HPLC with a Chiralcel IBN-3 column (hexane:2-propanol = 90/10, 2.0 mL/min, 254 nm); major enantiomer tr = 12.0 min, minor enantiomer tr = 15.3 min, 96% ee; $[\alpha]_D^{22.4}$= -62.0 (c= 0.5, $CHCl_3$, 96% ee)

**(*S*)-2-chloro-2-((2,5-dioxopyrrolidin-1-yl)(4-fluorophenyl)methyl)malononitrile (3g)**

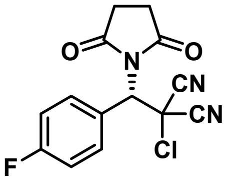

The reaction was performed following general procedure. The residue was purified by silica gel column chromatography (hexane/ethyl acetate = 4/1) to give a yellow amorphous solid (20.5 mg, 67% yield); $^{1}$H NMR (500 MHz, $CDCl_3$): δ 7.77-7.73 (m, 2H), 7.18-7.13 (m, 2H), 5.71 (s, 1H), 2.89 (s, 4H); $^{13}$C NMR(125 MHz, $CDCl_3$): δ 175.8, 165.1, 163.1, 132.3, 132.2, 126.2, 126.2, 116.7, 116.5, 110.7, 110.5, 62.2, 45.9, 28.0; HRMS calcd $C_{14}H_9O_2N_3ClFNNa$ $(M+Na)^+$: 328.0260, found: *m/z* 328.0265; Enantiomeric excess was determined by HPLC with a Chiralcel IBN-3 column (hexane:2-propanol = 90/10, 1.0 mL/min, 254 nm); major enantiomer tr = 29.5 min, minor enantiomer tr = 40.6 min, 96% ee; $[\alpha]_D^{22.9}$= -78.4 (c= 0.5, $CHCl_3$, 96% ee)

**(*S*)-2-chloro-2-((2-chlorophenyl)(2,5-dioxopyrrolidin-1-yl)methyl)malononitrile (3h)**

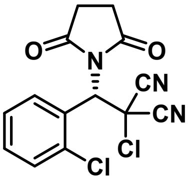

The reaction was performed following general procedure. The residue was purified by silica gel column chromatography (hexane/ethyl acetate = 4/1) to give a pale yellow amorphous solid (23.8 mg, 74% yield); $^{1}$H NMR (500 MHz, $CDCl_3$): δ 8.28-8.24 (m, 1H), 7.52-7.47 (m, 1H), 7.51-7.49 (m, 1H), 7.45-7.40 (m, 1H), 6.53 (s, 1H), 2.86 (s, 4H); $^{13}$C NMR(125 MHz, $CDCl_3$): δ 175.5, 135.1, 132.0, 131.2, 130.5, 128.3, 127.7, 110.7, 110.6, 57.3, 45.5, 28.0; HRMS calcd $C_{14}H_9O_2N_3Cl_2NNa$ $(M+Na)^+$: 343.9964, found: *m/z* 343.9966; Enantiomeric excess was determined by HPLC with a Chiralcel IBN-3 column (hexane:2-propanol = 90/10, 1.0 mL/min, 254 nm); major enantiomer tr = 28.0 min, minor enantiomer tr = 36.3 min, 34% ee; $[\alpha]_D^{23.1}$= -12.5 (c= 0.5, $CHCl_3$, 34% ee)

**(*S*)-2-chloro-2-((3-chlorophenyl)(2,5-dioxopyrrolidin-1-yl)methyl)malononitrile (3i)**

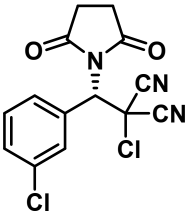

The reaction was performed following general procedure. The residue was purified by silica gel column chromatography (hexane/ethyl acetate = 4/1) to give a pale yellow amorphous solid (27.1 mg, 84% yield); $^{1}$H NMR (500 MHz, $CDCl_3$): δ 7.68 (t, *J* = 2.0 Hz, 1H), 7.66-7.65 (m, 1H), 7.49-7.47 (m, 1H), 7.41 (t, *J* = 7.9 Hz), 5.67 (s, 1H), 2.89 (s, 4H); $^{13}$C NMR(125 MHz, $CDCl_3$): δ 175.7, 135.3, 131.9, 131.4, 130.7, 130.1, 128.0, 110.6, 110.4, 62.3, 45.6, 28.0; HRMS calcd $C_{14}H_9O_2N_3Cl_2NNa$ $(M+Na)^+$: 343.9964, found: *m/z*343.9968; Enantiomeric excess was determined by HPLC with a Chiralcel IBN-3 column (hexane:2-propanol = 90/10, 1.0 mL/min, 254 nm); major enantiomer tr = 28.1 min, minor enantiomer tr = 37.6 min, 90% ee; $[\alpha]_D^{23.2}$= -66.1 (c= 0.5, $CHCl_3$, 90% ee)

**(*S*)-2-chloro-2-((4-chlorophenyl)(2,5-dioxopyrrolidin-1-yl)methyl)malononitrile (3j)**

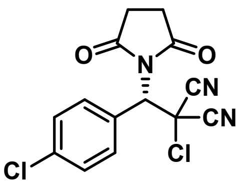

The reaction was performed following general procedure. The residue was purified by silica gel column chromatography (hexane/ethyl acetate = 4/1) to give a pale yellow amorphous solid (28.3 mg, 88% yield); $^1$H NMR (500 MHz, $CDCl_3$): δ 7.68 (d, *J*=8.59 Hz, 2H), 7.43(d, *J*=8.59 Hz, 2H), 5.68 (s, 1H), 2.88 (s, 4H); $^{13}$C NMR(125 MHz, $CDCl_3$): δ 175.7, 137.5, 131.4, 129.7, 128.6, 110.7, 110.4, 62.3, 45.7, 28.0; HRMS calcd $C_{14}H_9O_2N_3Cl_2NNa$ $(M+Na)^+$: 343.9964, found: *m/z* 343.9966; Enantiomeric excess was determined by HPLC with a Chiralcel AZ-3 column (hexane:2-propanol = 90/10, 1.0 mL/min, 254 nm); major enantiomer tr = 22.5 min, minor enantiomer tr = 25.6 min, 94% ee; $[\alpha]_D^{23.2}$= -46.7 (c= 0.5, $CHCl_3$, 94% ee)

**(*S*)-2-((2-bromophenyl)(2,5-dioxopyrrolidin-1-yl)methyl)-2-chloromalononitrile (3k)**

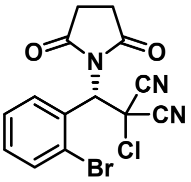

The reaction was performed following general procedure. The residue was purified by silica gel column chromatography (hexane/ethyl acetate = 4/1) to give a pale yellow amorphous solid (26.0 mg, 71% yield); $^1$H NMR (500 MHz, $CDCl_3$): δ 8.29 (dd, *J*=8.02 and 1.43 Hz, 1H), 7.69 (dd, *J*=8.02 and 1.43 Hz, 1H), 7.47 (dt, *J*=7.73 and 1.43 Hz, 1H), 7.34(dt, *J*=7.73 and 1.43 Hz, 1H), 6.53 (s, 1H), 2.86 (s, 4H); $^{13}$C NMR (125 MHz, $CDCl_3$): δ 175.5, 134.0, 132.2, 131.5, 130.0, 128.3, 125.6, 110.7, 110.6, 59.9, 45.6, 28.0; HRMS calcd $C_{14}H_9O_2N_3BrClNNa$ $(M+Na)^+$: 387.9459, found: *m/z* 387.9458; Enantiomeric excess was determined by HPLC with a Chiralcel IBN-3 column (hexane:2-propanol = 90/10, 1.0 mL/min, 254 nm); major enantiomer tr = 34.8 min, minor enantiomer tr = 39.9 min, 13% ee; $[\alpha]_D^{22.8}$= +6.3 (c= 0.5, $CHCl_3$, 13% ee)

**(*S*)-2-((3-bromophenyl)(2,5-dioxopyrrolidin-1-yl)methyl)-2-chloromalononitrile (3l)**

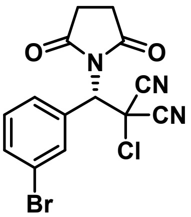

The reaction was performed following general procedure. The residue was purified by silica gel column chromatography (hexane/ethyl acetate = 4/1) to give a pale yellow amorphous solid (27.9 mg, 76% yield); $^1$H NMR (500 MHz, $CDCl_3$): δ 7.83 (t, *J*=1.72 Hz, 1H), 7.72 (d, *J*=7.73, 1H), 7.64 (d, *J*=8.59 Hz, 1H), 7.35 (t, *J*=7.88 Hz, 1H), 5.66 (s, 1H), 2.90 (s, 4H); $^{13}$C NMR (125 MHz, $CDCl_3$): δ 175.7, 134.3, 133.0, 132.1, 130.9, 128.5, 123.2, 110.5, 110.4, 62.2, 45.6, 28.0; HRMS calcd $C_{14}H_9O_2N_3BrClNNa$ $(M+Na)^+$: 387.9459, found: *m/z* 387.9466; Enantiomeric excess was determined by HPLC with a Chiralcel IBN-3 column (hexane:2-propanol = 90/10, 1.0 mL/min, 254 nm); major enantiomer tr = 31.9 min, minor enantiomer tr = 43.2 min, 97% ee; $[\alpha]_D^{23.3}$= -57.3 (c= 0.5, $CHCl_3$, 97% ee)

**(*S*)-2-((4-bromophenyl)(2,5-dioxopyrrolidin-1-yl)methyl)-2-chloromalononitrile (3m)**

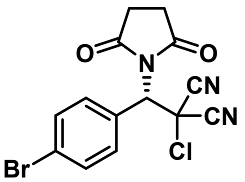

The reaction was performed following general procedure. The residue was purified by silica gel column chromatography (hexane/ethyl acetate = 4/1) to give a pale yellow amorphous solid (29.7 mg, 81% yield); $^1$H NMR (500 MHz, $CDCl_3$): δ 7.64-7.59 (m, 4H), 5.67 (s, 1H), 2.88 (s, 4H); $^{13}$C NMR(125 MHz, $CDCl_3$): δ 175.7, 132.7, 131.6, 129.1, 125.8, 110.7, 110.4, 62.4, 45.6, 28.0; HRMS calcd $C_{14}H_9O_2N_3BrClNNa$ $(M+Na)^+$: 387.9459, found: *m/z* 387.9452 Enantiomeric excess was determined by HPLC with a Chiralcel IBN-3 column (hexane:2-propanol = 90/10, 1.0 mL/min, 254 nm); major enantiomer tr = 32.8 min, minor enantiomer tr = 40.1 min, 94% ee; $[\alpha]_D^{22.9}$= -46.6 (c= 0.5, $CHCl_3$, 94% ee)

**(*S*)-2-chloro-2-((2,5-dioxopyrrolidin-1-yl)(3-(trifluoromethyl)phenyl)methyl)malononitrile (3n)**

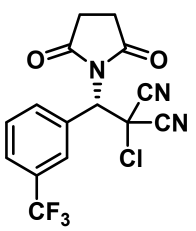

The reaction was performed following general procedure. The residue was purified by silica gel column chromatography (hexane/ethyl acetate = 4/1) to give a pale yellow amorphous solid (31.3 mg, 88% yield); $^{1}$H NMR (500 MHz, CDCl$_3$): δ 8.02 (d, *J*=8.02, Hz, 1H), 7.94 (s, 1H), 7.78 (d, *J*=8.02, 1H), 7.63 (t, *J*=7.88 Hz, 1H), 5.78 (s, 1H), 2.91 (s, 4H); $^{13}$C NMR(125 MHz, CDCl$_3$): δ 175.7, 133.2, 132.0, 131.7, 131.2, 130.2, 128.0, 127.1, 110.5, 110.3, 62.5, 45.6, 28.0; HRMS calcd $C_{15}H_9O_2N_3ClF_3NNa$ (M+Na)$^+$: 378.0228, found: *m/z* 378.0226; Enantiomeric excess was determined by HPLC with a Chiralcel OZ-3 column (hexane:2-propanol = 90/10, 1.0 mL/min, 254 nm); major enantiomer tr = 18.4 min, minor enantiomer tr = 16.3 min, 95% ee; $[\alpha]_D^{23.5}$= -44.7 (c= 0.5, CHCl$_3$, 95% ee)

**(*S*)-2-chloro-2-((2,5-dioxopyrrolidin-1-yl)(4-(trifluoromethyl)phenyl)methyl)malononitrile (3o)**

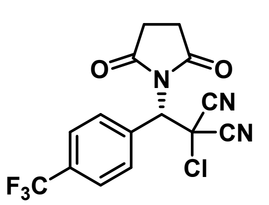

The reaction was performed following general procedure. The residue was purified by silica gel column chromatography (hexane/ethyl acetate = 4/1) to give a pale yellow amorphous solid (30.2 mg, 85% yield); $^{1}$H NMR (500 MHz, CDCl$_3$): δ 7.89 (d, *J*=8.31 Hz, 2H), 7.74 (d, *J*=8.31 Hz, 2H), 5.78 (s, 1H), 2.91 (s, 4H); $^{13}$C NMR(125 MHz, CDCl$_3$): δ 175.7, 133.9, 133.2, 133.0, 130.6, 126.4, 126.4, 124.4, 110.5, 110.3, 62.3, 45.5, 28.0; HRMS calcd $C_{15}H_9O_2N_3ClF_3Na$ (M+Na)$^+$: 378.0228, found: *m/z* 378.0229; Enantiomeric excess was determined by HPLC with a Chiralcel OZ-3 column (hexane:2-propanol = 90/10, 1.0 mL/min, 254 nm); major enantiomer tr = 16.1 min, minor enantiomer tr = 27.3 min, 93% ee; $[\alpha]_D^{23.5}$= -55.7 (c= 0.5, CHCl$_3$, 93% ee)

**(*S*)-2-chloro-2-((2,5-dioxopyrrolidin-1-yl)(naphthalen-2-yl)methyl)malononitrile (3p)**

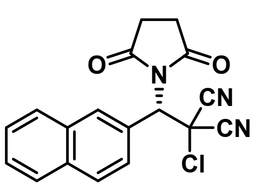

The reaction was performed following general procedure. The residue was purified by silica gel column chromatography (hexane/ethyl acetate = 4/1) to give a pale yellow amorphous solid (26.0 mg, 77% yield); $^{1}$H NMR (500 MHz, CDCl$_3$): δ 8.20 (s, 1H), 7.93-7.87 (m, 3H), 7.81 (dd, *J*=8.59 and 1.72 Hz, 1H), 7.60-7.55 (m, 2H), 5.88 (s, 1H), 2.88 (s, 4H); $^{13}$C NMR(125 MHz, CDCl$_3$): δ 175.9, 134.1, 132.8, 130.7, 129.5, 128.6, 127.9, 127.7, 127.5, 127.1, 125.7, 110.9, 110.7, 63.2, 45.9, 28.1; HRMS calcd $C_{18}H_{12}O_2N_3ClNNa$ (M+Na)$^+$: 360.0510, found: *m/z* 360.0504; Enantiomeric excess was determined by HPLC with a Chiralcel AZ-3 column (hexane:2-propanol = 90/10, 1.0 mL/min, 254 nm); major enantiomer tr = 25.5 min, minor enantiomer tr = 32.2 min, 91% ee; $[\alpha]_D^{23.6}$= -99.5 (c= 0.5, CHCl$_3$, 91% ee)

**methyl (*S*)-4-(2-chloro-2,2-dicyano-1-(2,5-dioxopyrrolidin-1-yl)ethyl)benzoate (3q)**

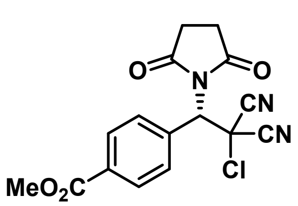

The reaction was performed following general procedure. The residue was purified by silica gel column chromatography (hexane/ethyl acetate = 4/1) to give a yellow amorphous solid (25.2 mg, 73% yield); $^{1}$H NMR (500 MHz, CDCl$_3$): δ 8.10 (d, *J*=8.59 Hz, 2H), 7.80 (d, *J*=8.31 Hz, 2H), 5.76 (s, 1H), 3.93 (s, 3H), 2.89 (s, 4H); $^{13}$C NMR (125 MHz, CDCl$_3$): δ 175.7, 165.9, 134.6, 132.6, 130.5, 130.0, 110.6, 110.4, 62.5, 52.5, 45.6, 28.1; HRMS calcd $C_{16}H_{12}O_4N_3ClNa$ (M+Na)$^+$: 368.0409, found: *m/z* 368.0401; Enantiomeric excess was determined by HPLC with a Chiralcel AZ-3 column (hexane:2-propanol = 80/20, 1.0 mL/min, 254 nm); major enantiomer tr = 27.1 min, minor enantiomer tr = 48.2 min, 91% ee; $[\alpha]_D^{23.6}$= -40.5 (c= 0.5, CHCl$_3$, 91% ee)

**methyl (*S*)-4-(2-chloro-2,2-dicyano-1-(2,5-dioxopyrrolidin-1-yl)ethyl)benzoate(3r)**

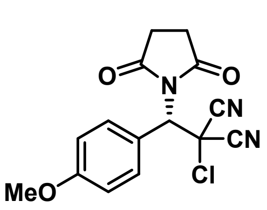

The reaction was performed following general procedure. The residue was purified by silica gel column chromatography (hexane/ethyl acetate = 4/1) to give a pale yellow amorphous solid (28.3 mg, 89% yield); $^1$H NMR (500 MHz, $CDCl_3$): δ 7.67 (d, *J*=8.98 Hz, 2H), 6.95 (d, *J*=8.98 Hz, 2H), 5.67 (s, 1H), 3.83 (s, 3H), 2.86 (s, 4H); $^{13}$C NMR (125 MHz, $CDCl_3$): δ 175.8, 161.5, 131.6, 122.1, 114.7, 111.0, 110.7, 62.5, 55.4, 46.1, 28.0; HRMS calcd $C_{15}H_{12}O_3N_3ClNa$ $(M+Na)^+$: 340.0459, found: *m/z* 340.0459; Enantiomeric excess was determined by HPLC with a Chiralcel AZ-3 column (hexane:2-propanol = 80/20, 1.0 mL/min, 254 nm); major enantiomer tr = 38.5 min, minor enantiomer tr = 33.1 min, 87% ee; $[\alpha]_D^{22.2}$= -31.6 (c= 0.5, $CHCl_3$, 87% ee)

**(*S*)-2-chloro-2-((2,5-dioxopyrrolidin-1-yl)(thiophen-2-yl)methyl)malononitrile (3s)**

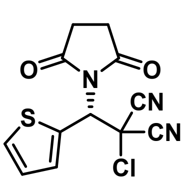

The reaction was performed following general procedure. The residue was purified by silica gel column chromatography (hexane/ethyl acetate = 4/1) to give a pale red amorphous solid (17.9 mg, 61% yield); $^1$H NMR (500 MHz, $CDCl_3$): δ 7.58 (dq, *J* = 3.7, 0.6 Hz, 1H), 7.48 (dd, *J* = 5.2, 0.9 Hz, 1H), 7.09 (dd, *J* = 5.2, 3.7 Hz, 1H), 6.05 (s, 1H), 2.88 (s, 4H) $^{13}$C NMR (125 MHz, $CDCl_3$): δ 175.1, 132.3, 129.6, 129.3, 127.5, 110.8, 110.4, 57.7, 46.3, 27.9; HRMS calcd $C_{12}H_9O_2N_3ClS$ $(M+H)^+$: 294.0099, found: *m/z* 294.0098; Enantiomeric excess was determined by HPLC with a Chiralcel IBN-3 column (hexane:2-propanol = 80/20, 1.0 mL/min, 254 nm); major enantiomer tr = 19.1 min, minor enantiomer tr = 30.7 min, 72% ee; $[\alpha]_D^{23.7}$= -40.1 (c= 0.5, $CHCl_3$, 72% ee)

**(*S*)-2-chloro-2-(cyclohexyl(2,5-dioxopyrrolidin-1-yl)methyl)malononitrile (3t)**

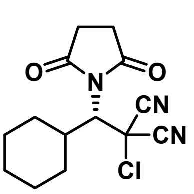

The reaction was performed following general procedure. The residue was purified by silica gel column chromatography (hexane/ethyl acetate = 4/1) to give a pale red oil (22.6 mg, 77% yield); $^1$H NMR (500 MHz, $CDCl_3$): δ 4.50 (d, *J*=10.6 Hz, 1H), 2.97-2.76 (m, 4H), 2.73-2.65 (m, 1H), 2.23 (d, *J*=12.6 Hz, 1H), 1.88-1.85 (m, 1H), 1.75-1.69 (m, 2H), 1.47 (d, *J*=12.6 Hz, 1H), 1.41-1.13 (m, 4H), 1.01 (dq, *J*=11.5 Hz and 3.72 Hz, 1H); $^{13}$C NMR (125 MHz, $CDCl_3$): δ 176.3, 175.3, 111.3, 62.3, 45.1, 36.9, 30.9, 30.1, 28.2, 27.3, 25.4, 25.2, 25.2; HRMS calcd $C_{14}H_{16}O_2N_3ClNa$ $(M+Na)^+$: 316.0823, found: *m/z* 316.0818; Enantiomeric excess was determined by HPLC with a Chiralcel IBN-3 column (hexane:2-propanol = 90/10, 1.0 mL/min, 254 nm); major enantiomer tr = 15.2 min, minor enantiomer tr = 28.4 min, 96% ee; $[\alpha]_D^{23.8}$= +9.64 (c= 0.5, $CHCl_3$, 96% ee)

**(*S*)-2-chloro-2-(cyclopentyl(2,5-dioxopyrrolidin-1-yl)methyl)malononitrile (3u)**

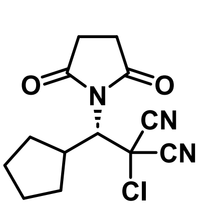

The reaction was performed following general procedure. The residue was purified by silica gel column chromatography (hexane/ethyl acetate = 4/1 ) to give a pale yellow oil (16.6 mg, 59% yield); 1H NMR (500 MHz, CDCl3): δ 4.57 (d, *J*=11.0 Hz, 1H), 3.18-3.07 (m, 1H), 2.97-2.71 (m, 4H), 2.24-2.15 (m, 1H), 1.92-1.83 (m, 1H), 1.80-1.47 (m, 6H), 1.28-1.17 (m, 1H); 13C NMR(125 MHz, CDCl3): δ 176.2, 175.2, 111.2, 110.5, 62.3, 45.6, 39.7, 31.2, 31.0, 28.2, 27.3, 25.9, 23.2; HRMS calcd $C_{13}H_{15}O_2N_3Cl$ $(M+H)^+$: 280.0847, found: *m/z* 280.0846; Enantiomeric excess was determined by HPLC with a Chiralcel IBN-3 column (hexane:2-propanol = 90/10, 1.0 mL/min, 254 nm); major enantiomer tr = 16.8 min, minor enantiomer tr = 31.6 min, 33% ee; $[\alpha]_D^{24.3}$= +8.52 (c= 0.5, $CHCl_3$, 33% ee)

**(*S,E*)-2-(2-bromo-2-phenylvinyl)-2-((2,5-dioxopyrrolidinlyl)(phenyl)methyl)malononitrile (4a)**

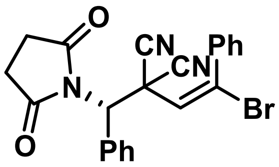


white amorphous solid; $^1$H NMR (500 MHz,$CDCl_3$): δ 7.72-7.69 (m, 2H), 7.50-7.42 (m, 6H), 7.41-7.37 (m, 2H), 6.20 (s, 1H), 5.59 (s, 1H), 2.80 (s, 4H); $^{13}$C NMR(125 MHz,$CDCl_3$): δ 176.1, 135.8, 131.9, 131.3, 130.7, 130.6, 129.9, 129.4, 128.8, 128.4, 121.0, 112.3, 111.2, 61.0, 40.7, 28.0; HRMS calcd $C_{22}H_{16}BrN_3O_2Na$ $(M+Na)^+$: 456.0318, found: *m/z* 456.0315; Enantiomeric excess was determined by HPLC with a Chiralcel AZ-3 column (hexane:2-propanol = 90/10, 1.0 mL/min, 254 nm); major enantiomer tr = 24.4 min, minor enantiomer tr = 38.7 min, 94% ee; $[\alpha]_D^{23.5}$= -17.9 (c= 1.0, $CHCl_3$, 94% ee)

**(*S*)-2-chloro-2-((2,5-dioxopyrrolidin-1-yl)(phenyl)methyl)malonamide (4b)**

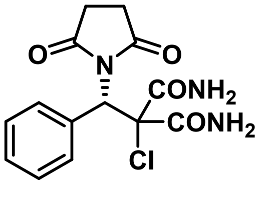


white amorphous solid; $^1$H NMR (500 MHz, Acetone-*d6*):δ 7.56-7.54 (m, 3H), 7.36 (s, 1H), 7.27-7.24 (m, 3H), 7.22 (d, J = 8.0 Hz, 1H), 7.05 (s, 1H), 6.86 (s, 1H), 2.69 (s, 4H); $^{13}$C NMR(125 MHz, Acetone-*d6*): δ 177.8, 169.2, 167.1, 136.7, 130.4, 128.7, 128.5, 77.4, 59.7, 28.7; HRMS calcd $C_{14}H_{15}ClN_3O_4$ $(M+H)^+$: 346.0565, found: *m/z* 346.0559; Enantiomeric excess was determined by HPLC with a Chiralcel AZ-3 column (hexane:EtOH = 10/90, 0.5 mL/min, 215 nm); major enantiomer tr = 21.9 min, minor enantiomer tr = 9.04 min, 92% ee; $[\alpha]_D^{23.9}$= -6.12 (c= 1.0, acetone, 92% ee)

**(2*S*,3*S*)-2-chloro-2-cyano-3-(2,5-dioxopyrrolidin-1-yl)-3-phenylpropanamide (4c)**

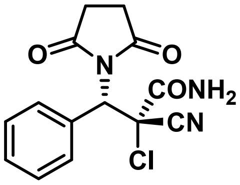


white solid; $^1$H NMR (500 MHz, Acetone-*d6*): δ 7.66-7.63 (m, 2H), 7.57 (d, J = 16.9 Hz, 2H), 7.41-7.35 (m, 3H), 6.27 (s, 1H), 2.87 (s, 4H); $^{13}$C NMR(125 MHz, Acetone-*d6*): δ 178.0, 163.8, 134.4, 130.5, 130.3, 129.5, 116.7, 59.3, 58.8, 28.7; HRMS calcd $C_{14}H_{13}ClN_3O_3$ $(M+H)^+$:306.0640, found: *m/z* 306.0636; Enantiomeric excess was determined by HPLC with a Chiralcel IBN-3 column (hexane:2-propanol = 60/40, 0.5 mL/min, 254 nm); major enantiomer tr = 25.7 min, minor enantiomer tr = 28.2 min, 95% ee; $[\alpha]_D^{24.9}$= -81.2 (c= 0.5, acetone, 95% ee)

**10. X-ray crystallographic Analysis**

**Crystal Structure Report for 2a (CCDC2363133)**

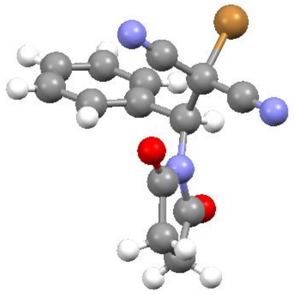

A fine crystal was obtained from a solution of *i*-PrOH/hexane.

A specimen of $C_{14}H_{10}BrN_3O_2$ was used for the X-ray crystallographic analysis. The X-ray intensity data were measured on a Bruker D8 goniometer system equipped with a INCOATEC IµS 3.0 (MoKα, λ = 0.71073 Å) and a Incoatec Helios multilayer confocal mirror (Incoatec) monochromator.

The total exposure time was 2.00 hours. The frames were integrated with the Bruker SAINT software package using a narrow-frame algorithm. The integration of the data using a monoclinic unit cell yielded a total of 3996 reflections to a maximum θ angle of 27.52° (0.77 Å resolution), of which 2696 were independent (average redundancy 1.482, completeness = 97.3%, Rint = 2.03%, Rsig = 5.47%) and 2430 (90.13%) were greater than 2σ(F2). The final cell constants of a = 8.180(3) Å, b = 6.428(2) Å, c = 13.353(5) Å, β = 92.574(5)°, volume = 701.4(4) Å$^3$, are based upon the refinement of the XYZ-centroids of 2145 reflections above 20 σ(I) with 4.985° < 2θ < 54.29°. Data were corrected for absorption effects using the multi-scan method (SADABS). The ratio of minimum to maximum apparent transmission was 0.743.

The structure was solved and refined using the Bruker SHELXTL Software Package, using the space group P 1 21 1, with Z = 2 for the formula unit, $C_{14}H_{10}BrN_3O_2$. The final anisotropic full-matrix least-squares refinement on F2 with 181 variables converged at R1 = 3.23%, for the observed data and wR2 = 5.88% for all data. The goodness-of-fit was 0.906. The largest peak in the final difference electron density synthesis was 0.450 e-/Å3 and the largest hole was -0.344 e-/Å3 with an RMS deviation of 0.069 e-/Å3. On the basis of the final model, the calculated density was 1.573 g/cm3 and F(000), 332 e-.

**Table 1. Sample and crystal data for 2a**

| | | |
|---|---|---|
| **Identification code** | **2a** | |
| **Chemical formula** | $C_{14}H_{10}BrN_3O_2$ | |
| **Formula weight** | 332.16 g/mol | |
| **Temperature** | 173(0) K | |
| **Wavelength** | 0.71073 Å | |
| **Crystal system** | Monoclinic | |
| **Space group** | P 1 21 1 | |
| **Unit cell dimensions** | a = 8.180(3) Å | α = 90° |

| | | |
|---|---|---|
| | b = 6.428(2) Å | β = 92.574(5)° |
| | c = 13.353(5) Å | γ = 90° |
| **Volume** | 701.4(4) $Å^3$ | |
| **Z** | 2 | |
| **Density (calculated)** | 1.573 $g/cm^3$ | |
| **Absorption coefficient** | 2.935 $mm^{-1}$ | |
| **F(000)** | 332 | |

**Table 2. Data collection and structure refinement for 2a.**

| | |
|---|---|
| **Diffractometer** | Bruker D8 goniometer |
| **Radiation source** | INCOATEC IμS 3.0 ($MoK_{\alpha}$, λ = 0.71073 Å) |
| **Theta range for data collection** | 1.53 to 27.52° |
| **Index ranges** | -10<=h<=9, -7<=k<=8, -14<=l<=17 |
| **Reflections collected** | 3996 |
| **Independent reflections** | 2696 [R(int) = 0.0203] |
| **Coverage of independent reflections** | 97.3% |
| **Absorption correction** | multi-scan |
| **Structure solution technique** | direct methods |
| **Structure solution program** | SHELXT 2018/2 (Sheldrick, 2018) |
| **Refinement method** | Full-matrix least-squares on $F^2$ |
| **Refinement program** | SHELXL-2018/3 (Sheldrick, 2018) |
| **Function minimized** | $\Sigma w(F_o^2 - F_c^2)^2$ |
| **Data / restraints / parameters** | 2696 / 1 / 181 |
| **Goodness-of-fit on $F^2$** | 0.655 |
| **$\Delta/\sigma_{max}$** | 0.001 |
| **Final R indices** | 2487 data; I>2σ(I) R1 = 0.0272, wR2 = 0.0588 |
| | all data R1 = 0.0323, wR2 = 0.0606 |
| **Weighting scheme** | $w=1/[\sigma^2(F_o^2)+(0.1000P)^2]$ where $P=(F_o^2+2F_c^2)/3$ |
| **Absolute structure parameter** | 0.039(10) |
| **Largest diff. peak and hole** | 0.450 and -0.344 $eÅ^{-3}$ |
| **R.M.S. deviation from mean** | 0.069 $eÅ^{-3}$ |

**Crystal Structure Report for *N*-Bn-PyBidine-Cu(OAc)$_2$ (CCDC2363134)**

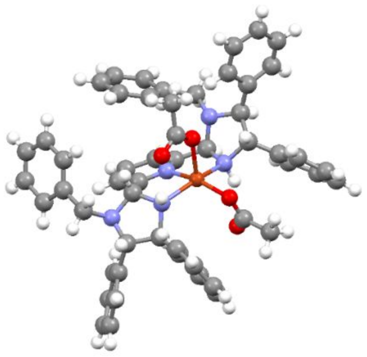

A fine crystal was obtained from a solution of DCE/hexane.

A blue, block-like specimen of $C_{26.50}H_{24.50}Cu_{0.50}N_{2.50}O_{2.25}$, approximate dimensions 0.300 mm x 0.350 mm x 0.400 mm, was used for the X-ray crystallographic analysis. The X-ray intensity data were measured on a Bruker D8 goniometer system equipped with a INCOATEC IμS 3.0 (MoK$_{\alpha}$, λ = 0.71073 Å) and a Incoatec Helios multilayer confocal mirror (Incoatec) monochromator.

The total exposure time was 1.72 hours. The frames were integrated with the Bruker SAINT software package using a narrow-frame algorithm. The integration of the data using a monoclinic unit cell yielded a total of 24263 reflections to a maximum θ angle of 27.52° (0.77 Å resolution), of which 10243 were independent (average redundancy 2.369, completeness = 96.5%, $R_{int}$ = 5.07%, $R_{sig}$ = 7.47%) and 9221 (90.02%) were greater than 2σ(F$^2$). The final cell constants of a = 18.5282(11) Å, b = 14.1975(10) Å, c = 19.1877(12) Å, β = 99.746(2)°, volume = 4974.6(6) Å$^3$, are based upon the refinement of the XYZ-centroids of 9954 reflections above 20 σ(I) with 4.412° < 2θ < 54.75°. Data were corrected for absorption effects using the multi-scan method (SADABS). The ratio of minimum to maximum apparent transmission was 0.660. The calculated minimum and maximum transmission coefficients (based on crystal size) are 0.8290 and 0.8680.

The structure was solved and refined using the Bruker SHELXTL Software Package, using the space group C 1 2 1, with Z = 8 for the formula unit, $C_{26.50}H_{24.50}Cu_{0.50}N_{2.50}O_{2.25}$. The final anisotropic full-matrix least-squares refinement on F$^2$ with 575 variables converged at R1 = 10.99%, for the observed data and wR2 = 30.44% for all data. The goodness-of-fit was 1.345. The largest peak in the final difference electron density synthesis was 4.193 e$^-$/Å$^3$ and the largest hole was -0.831 e$^-$/Å$^3$ with an RMS deviation of 0.235 e$^-$/Å$^3$. On the basis of the final model, the calculated density was 1.190 g/cm$^3$ and F(000), 1868 e$^-$.

**Table 1. Sample and crystal data for PyBidineCuOAc2.**

| | |
|---|---|
| **Identification code** | PyBidineCuOAc2 |
| **Chemical formula** | $C_{26.50}H_{24.50}Cu_{0.50}N_{2.50}O_{2.25}$ |
| **Formula weight** | 445.75 g/mol |
| **Temperature** | 173(2) K |
| **Wavelength** | 0.71073 Å |
| **Crystal size** | 0.300 x 0.350 x 0.400 mm |
| **Crystal habit** | blue block |

| | | |
|---|---|---|
| **Crystal system** | Monoclinic | |
| **Space group** | C 1 2 1 | |
| **Unit cell dimensions** | a = 18.5282(11) Å | α = 90° |
| | b = 14.1975(10) Å | β = 99.746(2)° |
| | c = 19.1877(12) Å | γ = 90° |
| **Volume** | 4974.6(6) $Å^3$ | |
| **Z** | 8 | |
| **Density (calculated)** | 1.190 $g/cm^3$ | |
| **Absorption coefficient** | 0.488 $mm^{-1}$ | |
| **F(000)** | 1868 | |

**Table 2. Data collection and structure refinement for PyBidineCuOAc2.**

| | | |
|---|---|---|
| **Diffractometer** | Bruker D8 goniometer | |
| **Radiation source** | INCOATEC IμS 3.0 ($MoK_\alpha$, λ = 0.71073 Å) | |
| **Theta range for data collection** | 1.08 to 27.52° | |
| **Index ranges** | -24<=h<=23, -17<=k<=17, -24<=l<=24 | |
| **Reflections collected** | 24263 | |
| **Independent reflections** | 10243 [R(int) = 0.0507] | |
| **Coverage of independent reflections** | 96.5% | |
| **Absorption correction** | multi-scan | |
| **Max. and min. transmission** | 0.8680 and 0.8290 | |
| **Structure solution technique** | direct methods | |
| **Structure solution program** | SHELXT 2018/2 (Sheldrick, 2018) | |
| **Refinement method** | Full-matrix least-squares on $F^2$ | |
| **Refinement program** | SHELXL-2019/1 (Sheldrick, 2019) | |
| **Function minimized** | $\Sigma w(F_o^2 - F_c^2)^2$ | |
| **Data / restraints / parameters** | 10243 / 1 / 575 | |
| **Goodness-of-fit on $F^2$** | 1.345 | |
| **$\Delta/\sigma_{max}$** | 0.022 | |
| **Final R indices** | 9221 data; I>2σ(I) | R1 = 0.1099, wR2 = 0.2943 |
| | all data | R1 = 0.1179, wR2 = 0.3044 |
| **Weighting scheme** | $w=1/[\sigma^2(F_o^2)+(0.2000P)^2]$<br>where $P=(F_o^2+2F_c^2)/3$ | |
| **Absolute structure parameter** | 0.080(6) | |
| **Largest diff. peak and hole** | 4.193 and -0.831 $eÅ^{-3}$ | |
| **R.M.S. deviation from mean** | 0.235 $eÅ^{-3}$ | |

**Crystal Structure Report for *N*-9-Anth-PyBidine-Cu(OAc)$_2$ (CCDC2363137)**

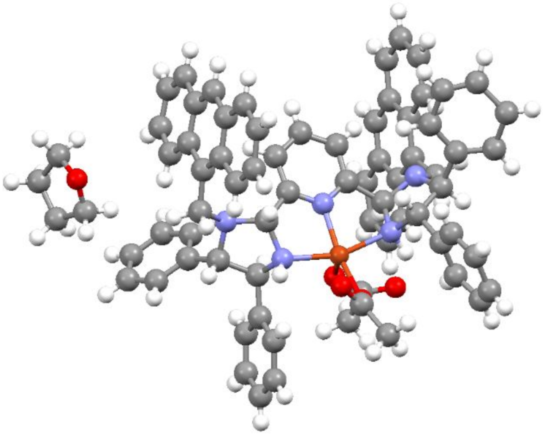

A fine crystal was obtained from a solution of DCE/hexane.

A blue, prism-like specimen of $C_{438}H_{402}Cu_6N_{30}O_{30}$, approximate dimensions 0.350 mm x 0.400 mm x 0.400 mm, was used for the X-ray crystallographic analysis. The X-ray intensity data were measured on a Bruker D8 goniometer system equipped with a INCOATEC IμS 3.0 (MoK$_\alpha$, λ = 0.71073 Å) and a Incoatec Helios multilayer confocal mirror (Incoatec) monochromator.

The total exposure time was 1.72 hours. The frames were integrated with the Bruker SAINT software package using a narrow-frame algorithm. The integration of the data using a hexagonal unit cell yielded a total of 114965 reflections to a maximum θ angle of 27.51° (0.77 Å resolution), of which 16328 were independent (average redundancy 7.041, completeness = 99.9%, $R_{int}$ = 5.11%, $R_{sig}$ = 4.09%) and 14057 (86.09%) were greater than 2σ($F^2$). The final cell constants of a = 20.382(4) Å, b = 20.382(4) Å, c = 31.375(6) Å, α = 90.00(3)°, β = 90.00(3)°, γ = 120.00(3)°, volume = 11288.(5) Å$^3$, are based upon the refinement of the XYZ-centroids of 9017 reflections above 20 σ(I) with 5.295° < 2θ < 51.90°. Data were corrected for absorption effects using the multi-scan method (SADABS). The ratio of minimum to maximum apparent transmission was 0.883. The calculated minimum and maximum transmission coefficients (based on crystal size) are 0.8770 and 0.8910.

The structure was solved and refined using the Bruker SHELXTL Software Package, using the space group P 62, with Z = 1 for the formula unit, $C_{438}H_{402}Cu_6N_{30}O_{30}$. The final anisotropic full-matrix least-squares refinement on $F^2$ with 759 variables converged at R1 = 5.92%, for the observed data and wR2 = 17.15% for all data. The goodness-of-fit was 1.123. The largest peak in the final difference electron density synthesis was 0.863 e$^-$/Å$^3$ and the largest hole was -0.421 e$^-$/Å$^3$ with an RMS deviation of 0.115 e$^-$/Å$^3$. On the basis of the final model, the calculated density was 1.022 g/cm$^3$ and F(000), 3654 e$^-$.

**Table 1. Sample and crystal data for AnthPyBidineCuOAc2.**

| | |
|---|---|
| **Identification code** | AnthPyBidineCuOAc2 |
| **Chemical formula** | $C_{438}H_{402}Cu_6N_{30}O_{30}$ |
| **Formula weight** | 6947.11 g/mol |
| **Temperature** | 173(0) K |

| | |
|---|---|
| **Wavelength** | 0.71073 Å |
| **Crystal size** | 0.350 x 0.400 x 0.400 mm |
| **Crystal habit** | blue prism |
| **Crystal system** | Hexagonal |
| **Space group** | P 62 |
| **Unit cell dimensions** | a = 20.382(4) Å α = 90.00(3)° |
| | b = 20.382(4) Å β = 90.00(3)° |
| | c = 31.375(6) Å γ = 120.00(3)° |
| **Volume** | 11288.(5) $Å^3$ |
| **Z** | 1 |
| **Density (calculated)** | 1.022 $g/cm^3$ |
| **Absorption coefficient** | 0.336 $mm^{-1}$ |
| **F(000)** | 3654 |

**Table 2. Data collection and structure refinement for AnthPyBidineCuOAc2.**

| | |
|---|---|
| **Diffractometer** | Bruker D8 goniometer |
| **Radiation source** | INCOATEC IµS 3.0 ($MoK_{\alpha}$, λ = 0.71073 Å) |
| **Theta range for data collection** | 2.10 to 27.51° |
| **Index ranges** | -26<=h<=25, -26<=k<=26, -35<=l<=40 |
| **Reflections collected** | 114965 |
| **Independent reflections** | 16328 [R(int) = 0.0511] |
| **Coverage of independent reflections** | 99.9% |
| **Absorption correction** | multi-scan |
| **Max. and min. transmission** | 0.8910 and 0.8770 |
| **Structure solution technique** | direct methods |
| **Structure solution program** | SHELXT 2018/2 (Sheldrick, 2018) |
| **Refinement method** | Full-matrix least-squares on $F^2$ |
| **Refinement program** | SHELXL-2018/3 (Sheldrick, 2018) |
| **Function minimized** | $\Sigma w(F_o^2 - F_c^2)^2$ |
| **Data / restraints / parameters** | 16328 / 1 / 759 |
| **Goodness-of-fit on $F^2$** | 1.123 |
| **$\Delta/\sigma_{max}$** | 0.001 |
| **Final R indices** | 14057 data; I>2σ(I) R1 = 0.0592, wR2 = 0.1603 |
| | all data R1 = 0.0705, wR2 = 0.1715 |
| **Weighting scheme** | $w=1/[\sigma^2(F_o^2)+(0.1221P)^2]$ |

| | |
|---|---|
| | where $P=(F_o^2+2F_c^2)/3$ |
| **Absolute structure parameter** | 0.008(3) |
| **Largest diff. peak and hole** | 0.863 and -0.421 eÅ$^{-3}$ |
| **R.M.S. deviation from mean** | 0.115 eÅ$^{-3}$ |

**Crystal Structure Report for *rac*-4c (CCDC 2394709)**

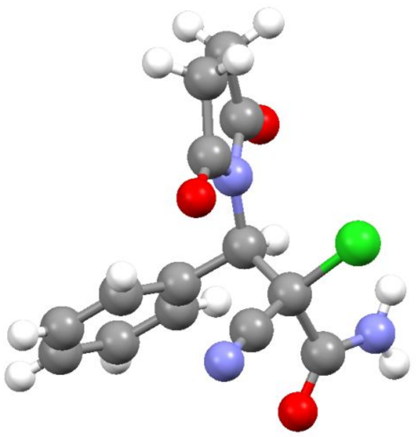

A fine crystal was obtained from a solution of DCE/hexane.

A colorless, plate-like specimen of $C_{14}H_{12}ClN_3O_3$, approximate dimensions 0.050 mm x 0.150 mm x 0.250 mm, was used for the X-ray crystallographic analysis. The X-ray intensity data were measured on a Bruker D8 goniometer system equipped with a INCOATEC IμS 3.0 (MoK$_{\alpha}$, λ = 0.71073 Å) and a Incoatec Helios multilayer confocal mirror (Incoatec) monochromator.

A total of 780 frames were collected. The total exposure time was 0.88 hours. The frames were integrated with the Bruker SAINT software package using a narrow-frame algorithm. The integration of the data using an orthorhombic unit cell yielded a total of 13939 reflections to a maximum θ angle of 27.52° (0.77 Å resolution), of which 3223 were independent (average redundancy 4.325, completeness = 99.0%, $R_{int}$ = 4.23%, $R_{sig}$ = 3.63%) and 3165 (98.20%) were greater than 2σ($F^2$). The final cell constants of a = 6.9673(3) Å, b = 13.5585(5) Å, c = 15.1049(5) Å, volume = 1426.90(9) Å$^3$, are based upon the refinement of the XYZ-centroids of 9140 reflections above 20 σ(I) with 5.393° < 2θ < 55.04°. Data were corrected for absorption effects using the multi-scan method (SADABS). The ratio of minimum to maximum apparent transmission was 0.955. The calculated minimum and maximum transmission coefficients (based on crystal size) are 0.9330 and 0.9860.

The structure was solved and refined using the Bruker SHELXTL Software Package, using the space group P 21 21 21, with Z = 4 for the formula unit, $C_{14}H_{12}ClN_3O_3$. The final anisotropic full-matrix least-squares refinement on $F^2$ with 190 variables converged at R1 = 2.79%, for the observed data and wR2 = 8.90% for all data. The goodness-of-fit was 0.811. The largest peak in the final difference electron density synthesis was 0.214 e$^-$/Å$^3$ and the largest hole was -0.226 e$^-$/Å$^3$ with an RMS deviation of 0.046 e$^-$/Å$^3$. On the basis of the final model, the calculated density was 1.423 g/cm$^3$ and F(000), 632 e$^-$.

| **Table 1. Sample and crystal data for *rac*-4c (CCDC 2394709).** | |
|---|---|
| **Identification code** | saito20241028 |
| **Chemical formula** | $C_{14}H_{12}ClN_3O_3$ |
| **Formula weight** | 305.72 g/mol |
| **Temperature** | 173(0) K |
| **Wavelength** | 0.71073 Å |

| | | |
|---|---|---|
| **Crystal size** | 0.050 x 0.150 x 0.250 mm | |
| **Crystal habit** | colorless plate | |
| **Crystal system** | orthorhombic | |
| **Space group** | P 21 21 21 | |
| **Unit cell dimensions** | a = 6.9673(3) Å | α = 90° |
| | b = 13.5585(5) Å | β = 90° |
| | c = 15.1049(5) Å | γ = 90° |
| **Volume** | 1426.90(9) $Å^3$ | |
| **Z** | 4 | |
| **Density (calculated)** | 1.423 $g/cm^3$ | |
| **Absorption coefficient** | 0.281 $mm^{-1}$ | |
| **F(000)** | 632 | |

| **Table 2. Data collection and structure refinement for *rac*-4c (CCDC 2394709).** | | |
|---|---|---|
| **Diffractometer** | Bruker D8 goniometer | |
| **Radiation source** | INCOATEC IμS 3.0 ($MoK_\alpha$, λ = 0.71073 Å) | |
| **Theta range for data collection** | 2.70 to 27.52° | |
| **Index ranges** | -8<=h<=9, -17<=k<=17, -19<=l<=19 | |
| **Reflections collected** | 13939 | |
| **Independent reflections** | 3223 [R(int) = 0.0423] | |
| **Coverage of independent reflections** | 99.0% | |
| **Absorption correction** | multi-scan | |
| **Max. and min. transmission** | 0.9860 and 0.9330 | |
| **Structure solution technique** | direct methods | |
| **Structure solution program** | SHELXT 2018/2 (Sheldrick, 2018) | |
| **Refinement method** | Full-matrix least-squares on $F^2$ | |
| **Refinement program** | SHELXL-2018/3 (Sheldrick, 2018) | |
| **Function minimized** | $\Sigma w(F_o^2 - F_c^2)^2$ | |
| **Data / restraints / parameters** | 3223 / 0 / 190 | |
| **Goodness-of-fit on $F^2$** | 0.811 | |
| **$\Delta/\sigma_{max}$** | 0.001 | |
| **Final R indices** | 3165 data; I>2σ(I) | R1 = 0.0279, wR2 = 0.0874 |
| | all data | R1 = 0.0285, wR2 = 0.0890 |
| **Weighting scheme** | $w=1/[\sigma^2(F_o^2)+(0.1000P)^2]$<br>where $P=(F_o^2+2F_c^2)/3$ | |
| **Absolute structure parameter** | 0.346(16) | |
| **Largest diff. peak and hole** | 0.214 and -0.226 $eÅ^{-3}$ | |
| **R.M.S. deviation from mean** | 0.046 $eÅ^{-3}$ | |

## 11. DFT calculations

All calculations were performed with the Gaussian 16 package.[S1]

For the possible transition state, the model was constructed for benzylidenemalononitrile (1a) and *N*-9-AnthPyBidine (L10)-Cu(II) complex coordinated acetoxy anion and succinimide anion, and optimized with the B3LYP method using LANL2DZ for Cu and 6-31G* for the rest. Frequency analyses were carried out to identify the stationary points (TS: one imaginary frequency) and to estimate thermodynamic properties at 195.15 K and Gibbs free energies. In the possible transition state, benzylidenemalononitrile made a hydrogen bond between N-H proton of PyBidine ligand and C≡N nitrogen atom of nitrle in 2.17 Å. The proposed TS explain the formation of the (S)-enriched product using (S,S)-diphenylethanediamine-derived *N*-9-AnthPyBidine-Cu catalyst.

(Ref.) S1. Gaussian 16, Revision C.01, Frisch, M. J.; Trucks, G. W.; Schlegel, H. B.; Scuseria, G. E.; Robb, M. A.; Cheeseman, J. R.; Scalmani, G.; Barone, V.; Petersson, G. A.; Nakatsuji, H.; Li, X.; Caricato, M.; Marenich, A. V.; Bloino, J.; Janesko, B. G.; Gomperts, R.; Mennucci, B.; Hratchian, H. P.; Ortiz, J. V.; Izmaylov, A. F.; Sonnenberg, J. L.; Williams-Young D.; Ding, F.; Lipparini, F.; Egidi, F.; Goings, J.; Peng, B.; Petrone, A.; Henderson, T.; Ranasinghe, D.; Zakrzewski, V. G.; Gao, J.; Rega, N.; Zheng, G.; Liang, W.; Hada, M.; Ehara, M.; Toyota, K.; Fukuda, R.; Hasegawa, J.; Ishida, M.; Nakajima, T.; Honda, Y.; Kitao, O.; Nakai, H.; Vreven, T.; Throssell, K.; Montgomery, J. A. Jr.; Peralta, J. E.; Ogliaro, F.; Bearpark, M. J.; Heyd, J. J.; Brothers, E. N.; Kudin, K. N.; Staroverov, V. N.; Keith, T. A.; Kobayashi, R.; Normand, J.; Raghavachari, K.; Rendell,A. P.; Burant, J. C.; Iyengar, S. S.; Tomasi, J.; Cossi, M.; Millam, J. M.; Klene, M.; Adamo, C.; Cammi, R.; Ochterski, J. W.; Martin, R. L.; Morokuma, K.; Farkas, O.; Foresman, J. B.; Fox, D. J. Gaussian, Inc., Wallingford CT

Cartesian coordinate

***Plausible TS***

SCF Done: E(UB3LYP) = -4063.202335 A.U.

Sum of electronic and thermal Free Energy = -4061.973496

| Center Number | Atomic Number | Atomic Type | Coordinates (Angstroms) X | Y | Z |
|---|---|---|---|---|---|
| 1 | 29 | 0 | -0.385714 | 0.496795 | 1.046428 |
| 2 | 7 | 0 | 2.775958 | 2.854085 | -0.356559 |
| 3 | 6 | 0 | 5.399811 | 1.996207 | 1.706030 |
| 4 | 6 | 0 | 5.094447 | 2.561525 | 0.443992 |
| 5 | 8 | 0 | 0.010907 | 2.454853 | 3.401235 |
| 6 | 6 | 0 | 6.903404 | 1.218710 | -0.584555 |
| 7 | 6 | 0 | 1.713107 | -6.203739 | 3.871768 |
| 8 | 6 | 0 | 0.674219 | -5.427767 | 3.351633 |
| 9 | 6 | 0 | -1.119132 | 1.929159 | 3.371729 |
| 10 | 6 | 0 | 2.179051 | 1.762193 | 0.411388 |
| 11 | 6 | 0 | 1.793350 | -2.072002 | -1.453375 |
| 12 | 6 | 0 | 1.779121 | 4.260076 | -2.168867 |
| 13 | 6 | 0 | 4.686701 | 2.308137 | 2.914366 |
| 14 | 6 | 0 | 0.877045 | -4.639621 | 2.212814 |
| 15 | 6 | 0 | 7.197392 | 0.673181 | 0.669054 |
| 16 | 6 | 0 | 1.702845 | 3.794844 | -0.731705 |
| 17 | 6 | 0 | 2.143538 | 3.368714 | -3.188125 |
| 18 | 6 | 0 | 2.137993 | -4.637316 | 1.600249 |

| | | | | | |
|---|---|---|---|---|---|
| 19 | 6 | 0 | 0.396744 | 3.005836 | -0.429154 |
| 20 | 6 | 0 | 7.316030 | 1.331996 | -2.984674 |
| 21 | 6 | 0 | 1.418802 | 5.984700 | -3.840068 |
| 22 | 6 | 0 | 5.538161 | 2.681970 | -2.015979 |
| 23 | 6 | 0 | 7.631161 | 0.824866 | -1.751761 |
| 24 | 6 | 0 | 2.150799 | 3.783377 | -4.520353 |
| 25 | 6 | 0 | 1.787930 | 5.093407 | -4.850240 |
| 26 | 6 | 0 | 3.175657 | -5.415259 | 2.115977 |
| 27 | 6 | 0 | 1.416037 | 5.568778 | -2.506039 |
| 28 | 1 | 0 | 1.541331 | -6.813816 | 4.754411 |
| 29 | 1 | 0 | -0.300755 | -5.431941 | 3.833315 |
| 30 | 1 | 0 | 2.709927 | 1.581407 | 1.350430 |
| 31 | 1 | 0 | 1.642538 | -3.065859 | -1.854125 |
| 32 | 1 | 0 | 3.883076 | 3.034896 | 2.897158 |
| 33 | 1 | 0 | 8.000244 | -0.055759 | 0.755753 |
| 34 | 1 | 0 | 1.720913 | 4.670346 | -0.063581 |
| 35 | 1 | 0 | 2.434047 | 2.356035 | -2.925425 |
| 36 | 1 | 0 | 2.290730 | -4.035709 | 0.709344 |
| 37 | 1 | 0 | 0.199033 | 2.315949 | -1.254089 |
| 38 | 1 | 0 | 7.870382 | 1.021718 | -3.865754 |
| 39 | 1 | 0 | 1.137073 | 7.004419 | -4.088006 |
| 40 | 1 | 0 | 4.720132 | 3.376730 | -2.155850 |
| 41 | 1 | 0 | 8.437990 | 0.105210 | -1.636916 |
| 42 | 1 | 0 | 2.440775 | 3.085118 | -5.301031 |
| 43 | 1 | 0 | 1.795609 | 5.416987 | -5.887465 |
| 44 | 1 | 0 | 4.146952 | -5.410429 | 1.628301 |
| 45 | 1 | 0 | 1.126694 | 6.263236 | -1.720983 |
| 46 | 6 | 0 | -0.813255 | 3.875795 | -0.214134 |
| 47 | 6 | 0 | -0.983918 | 4.630801 | 0.955094 |
| 48 | 6 | 0 | -1.749205 | 4.002009 | -1.247151 |
| 49 | 6 | 0 | -2.068596 | 5.499079 | 1.079259 |
| 50 | 1 | 0 | -0.284243 | 4.534499 | 1.780308 |
| 51 | 6 | 0 | -2.834751 | 4.870259 | -1.123698 |
| 52 | 1 | 0 | -1.622101 | 3.419906 | -2.155048 |
| 53 | 6 | 0 | -2.993221 | 5.625445 | 0.038812 |
| 54 | 1 | 0 | -2.194812 | 6.073542 | 1.992908 |
| 55 | 1 | 0 | -3.557210 | 4.949498 | -1.930945 |
| 56 | 1 | 0 | -3.843570 | 6.292149 | 0.144147 |
| 57 | 6 | 0 | 3.931734 | 3.517742 | 0.272130 |
| 58 | 1 | 0 | 3.648916 | 3.989258 | 1.223030 |
| 59 | 1 | 0 | 4.218119 | 4.340701 | -0.389364 |
| 60 | 6 | 0 | 4.996215 | 1.708728 | 4.108828 |
| 61 | 6 | 0 | 6.052920 | 0.757338 | 4.198407 |

| | | | | | |
|---|---|---|---|---|---|
| 62 | 6 | 0 | 6.474512 | 1.028962 | 1.810515 |
| 63 | 6 | 0 | 6.771635 | 0.435407 | 3.078759 |
| 64 | 6 | 0 | 5.830902 | 2.179171 | -0.704592 |
| 65 | 6 | 0 | 6.251444 | 2.269972 | -3.113472 |
| 66 | 1 | 0 | 4.432351 | 1.967766 | 5.000562 |
| 67 | 1 | 0 | 6.283758 | 0.296139 | 5.154217 |
| 68 | 1 | 0 | 7.583013 | -0.286480 | 3.129395 |
| 69 | 1 | 0 | 5.998958 | 2.662323 | -4.094781 |
| 70 | 6 | 0 | -0.592558 | -1.962770 | 3.592578 |
| 71 | 6 | 0 | 0.220594 | -1.175597 | 4.417273 |
| 72 | 6 | 0 | -1.857890 | -2.352581 | 4.052624 |
| 73 | 6 | 0 | -0.218833 | -0.779798 | 5.681780 |
| 74 | 1 | 0 | 1.197760 | -0.858719 | 4.060375 |
| 75 | 6 | 0 | -2.298839 | -1.956189 | 5.316170 |
| 76 | 1 | 0 | -2.511433 | -2.943541 | 3.418459 |
| 77 | 6 | 0 | -1.481918 | -1.169167 | 6.133208 |
| 78 | 1 | 0 | 0.421044 | -0.164271 | 6.307610 |
| 79 | 1 | 0 | -3.283356 | -2.259910 | 5.660879 |
| 80 | 1 | 0 | -1.829565 | -0.859822 | 7.114919 |
| 81 | 6 | 0 | -1.167988 | -4.544345 | -0.449771 |
| 82 | 1 | 0 | -0.907878 | -5.546330 | -0.094014 |
| 83 | 1 | 0 | -2.201222 | -4.369907 | -0.131405 |
| 84 | 6 | 0 | -1.026847 | -4.482265 | -1.957826 |
| 85 | 6 | 0 | -1.996470 | -3.828453 | -2.757983 |
| 86 | 6 | 0 | 0.125293 | -5.047461 | -2.558135 |
| 87 | 6 | 0 | -3.173005 | -3.199116 | -2.224311 |
| 88 | 6 | 0 | -1.808955 | -3.764306 | -4.193783 |
| 89 | 6 | 0 | 0.306507 | -4.963652 | -3.989385 |
| 90 | 6 | 0 | -4.089649 | -2.580379 | -3.035801 |
| 91 | 1 | 0 | -3.351299 | -3.202312 | -1.157063 |
| 92 | 6 | 0 | -2.795256 | -3.118298 | -5.005965 |
| 93 | 6 | 0 | -0.667727 | -4.330463 | -4.767813 |
| 94 | 6 | 0 | -3.908993 | -2.548967 | -4.449013 |
| 95 | 1 | 0 | -2.638934 | -3.094154 | -6.081614 |
| 96 | 1 | 0 | -0.531372 | -4.272563 | -5.845612 |
| 97 | 6 | 0 | 2.900117 | -1.295510 | -1.809730 |
| 98 | 6 | 0 | 3.083451 | -0.019973 | -1.264081 |
| 99 | 6 | 0 | 0.875338 | -1.530706 | -0.557288 |
| 100 | 6 | 0 | 2.136251 | 0.442013 | -0.356441 |
| 101 | 7 | 0 | 1.080123 | -0.315119 | -0.043452 |
| 102 | 1 | 0 | 3.625766 | -1.686880 | -2.516315 |
| 103 | 1 | 0 | 3.929090 | 0.599787 | -1.532076 |
| 104 | 1 | 0 | -4.655904 | -2.071696 | -5.076593 |

| | | | | | |
|---|---|---|---|---|---|
| 105 | 1 | 0 | -4.961819 | -2.102899 | -2.598437 |
| 106 | 6 | 0 | 1.159029 | -5.688792 | -1.797331 |
| 107 | 6 | 0 | 2.278312 | -6.200589 | -2.404074 |
| 108 | 6 | 0 | 2.446798 | -6.119042 | -3.816554 |
| 109 | 1 | 0 | 3.047942 | -6.672566 | -1.799367 |
| 110 | 1 | 0 | 1.074732 | -5.746014 | -0.719796 |
| 111 | 1 | 0 | 3.339174 | -6.532775 | -4.277548 |
| 112 | 6 | 0 | 1.483674 | -5.518858 | -4.583965 |
| 113 | 1 | 0 | 1.597995 | -5.448253 | -5.662921 |
| 114 | 8 | 0 | -1.566723 | 1.157749 | 2.430889 |
| 115 | 6 | 0 | -2.106865 | 2.214109 | 4.493336 |
| 116 | 1 | 0 | -2.925337 | 1.492445 | 4.500915 |
| 117 | 1 | 0 | -1.585191 | 2.199202 | 5.453689 |
| 118 | 1 | 0 | -2.520334 | 3.220078 | 4.351052 |
| 119 | 7 | 0 | -0.242205 | -3.610110 | 0.222609 |
| 120 | 6 | 0 | -0.236033 | -3.754241 | 1.695908 |
| 121 | 1 | 0 | -1.203156 | -4.155439 | 2.029494 |
| 122 | 6 | 0 | -0.117606 | -2.290044 | 2.200688 |
| 123 | 1 | 0 | 0.930736 | -1.987547 | 2.109593 |
| 124 | 6 | 0 | 2.966187 | -6.200777 | 3.253921 |
| 125 | 1 | 0 | 3.773082 | -6.808912 | 3.653621 |
| 126 | 7 | 0 | -0.860079 | -1.526069 | 1.155514 |
| 127 | 1 | 0 | -1.861632 | -1.694673 | 1.274716 |
| 128 | 7 | 0 | 0.785209 | 2.162295 | 0.726067 |
| 129 | 1 | 0 | 0.732009 | 2.638303 | 1.636592 |
| 130 | 6 | 0 | -0.422751 | -2.194437 | -0.097516 |
| 131 | 1 | 0 | -1.179217 | -2.031022 | -0.866393 |
| 132 | 6 | 0 | -5.148557 | 1.897506 | 0.902221 |
| 133 | 6 | 0 | -6.383482 | 2.222820 | 0.313120 |
| 134 | 6 | 0 | -6.975850 | 3.456044 | 0.575701 |
| 135 | 6 | 0 | -6.355988 | 4.372731 | 1.432741 |
| 136 | 6 | 0 | -5.117168 | 4.064796 | 1.997974 |
| 137 | 6 | 0 | -4.506133 | 2.843139 | 1.718526 |
| 138 | 1 | 0 | -6.839279 | 1.530904 | -0.383053 |
| 139 | 1 | 0 | -7.925221 | 3.704473 | 0.109144 |
| 140 | 1 | 0 | -6.829226 | 5.328295 | 1.642573 |
| 141 | 1 | 0 | -4.614226 | 4.781699 | 2.639855 |
| 142 | 1 | 0 | -3.529061 | 2.609308 | 2.125909 |
| 143 | 6 | 0 | -4.503751 | 0.581119 | 0.771027 |
| 144 | 1 | 0 | -3.458067 | 0.561823 | 1.054864 |
| 145 | 6 | 0 | -5.179055 | -0.639306 | 0.875538 |
| 146 | 6 | 0 | -4.413766 | -1.815518 | 1.052269 |
| 147 | 6 | 0 | -6.583333 | -0.814303 | 0.754114 |

| | | | | | |
|---|---|---|---|---|---|
| 148 | 7 | 0 | -7.733688 | -0.992247 | 0.673815 |
| 149 | 7 | 0 | -3.746925 | -2.764234 | 1.208434 |
| 150 | 6 | 0 | -2.341216 | 0.624898 | -3.209182 |
| 151 | 6 | 0 | -3.764123 | 0.908666 | -3.692261 |
| 152 | 1 | 0 | -1.585582 | 1.348021 | -3.531333 |
| 153 | 1 | 0 | -1.994030 | -0.372809 | -3.502686 |
| 154 | 1 | 0 | -3.883610 | 1.934574 | -4.060180 |
| 155 | 1 | 0 | -4.127870 | 0.232070 | -4.467980 |
| 156 | 6 | 0 | -2.487432 | 0.653657 | -1.684175 |
| 157 | 6 | 0 | -4.601794 | 0.758922 | -2.409748 |
| 158 | 8 | 0 | -1.500239 | 0.581888 | -0.925304 |
| 159 | 8 | 0 | -5.826521 | 0.682908 | -2.385681 |
| 160 | 7 | 0 | -3.775981 | 0.730653 | -1.305569 |

---------------------------------------------------------------------------------------------------------

## 12. NMR spectra

$^1$HNMR (500 MHz, chloroform-*d*) spectrum of 2a

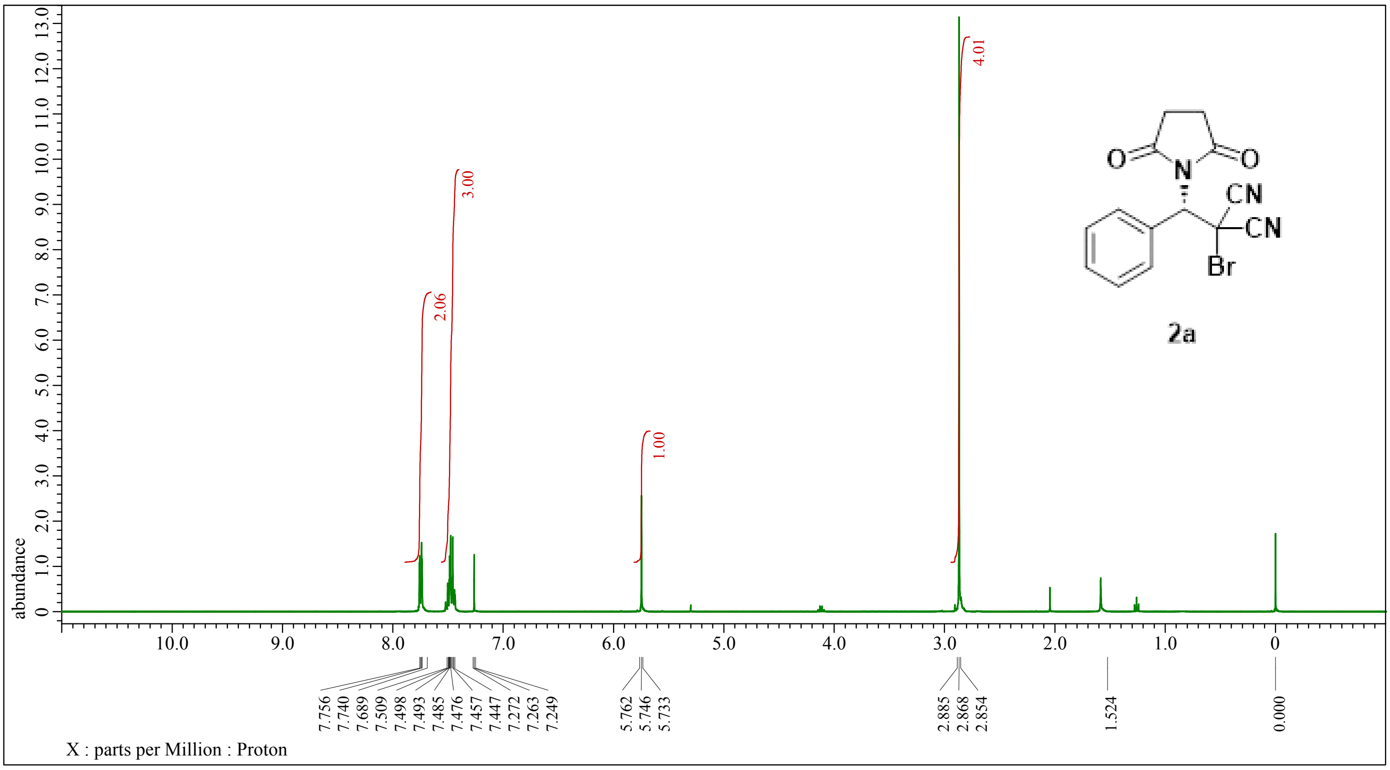


$^{13}$CNMR (125 MHz, chloroform-*d*) spectrum of 2a

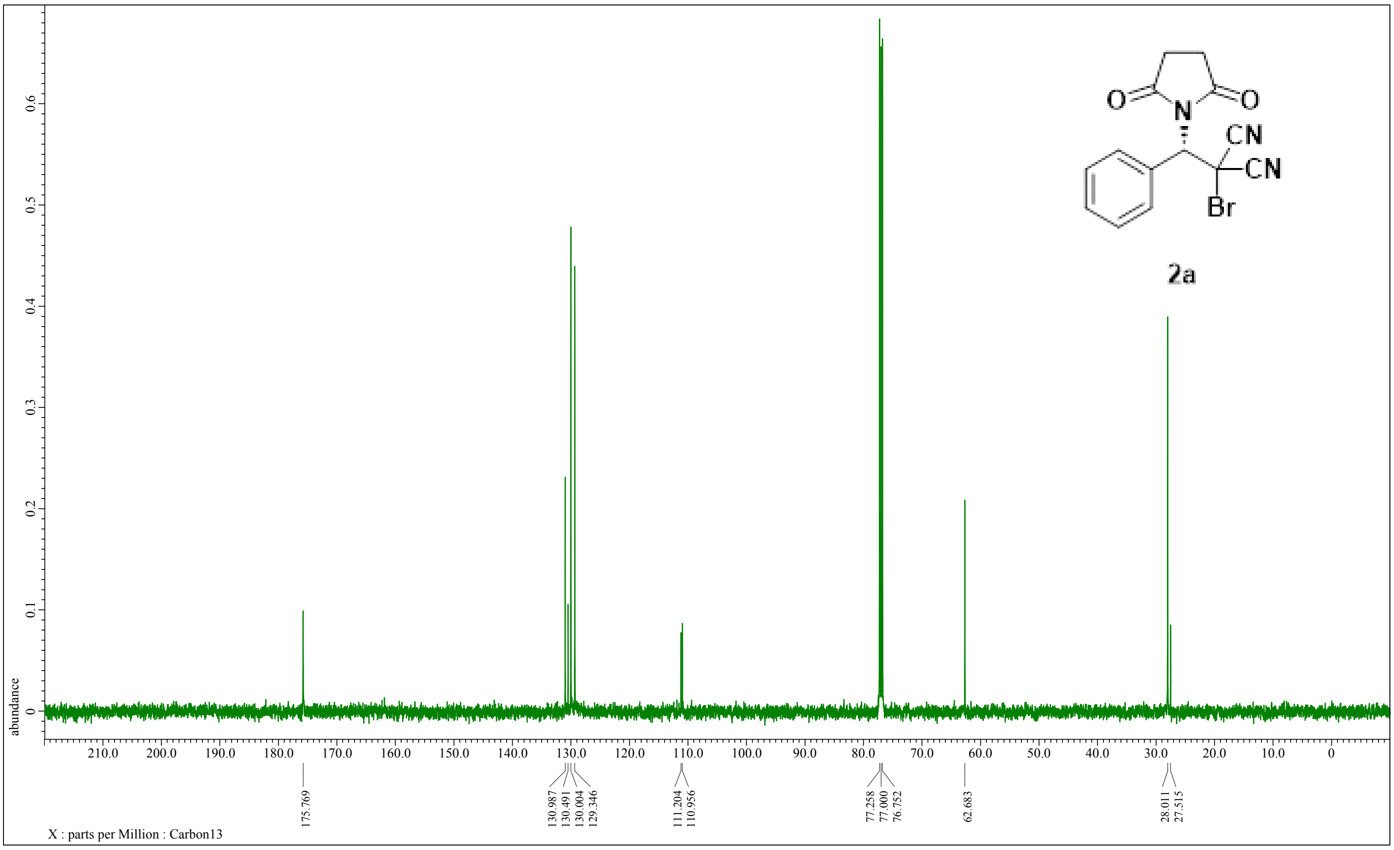

$^{1}$HNMR (500 MHz, chloroform-*d*) spectrum of 2b

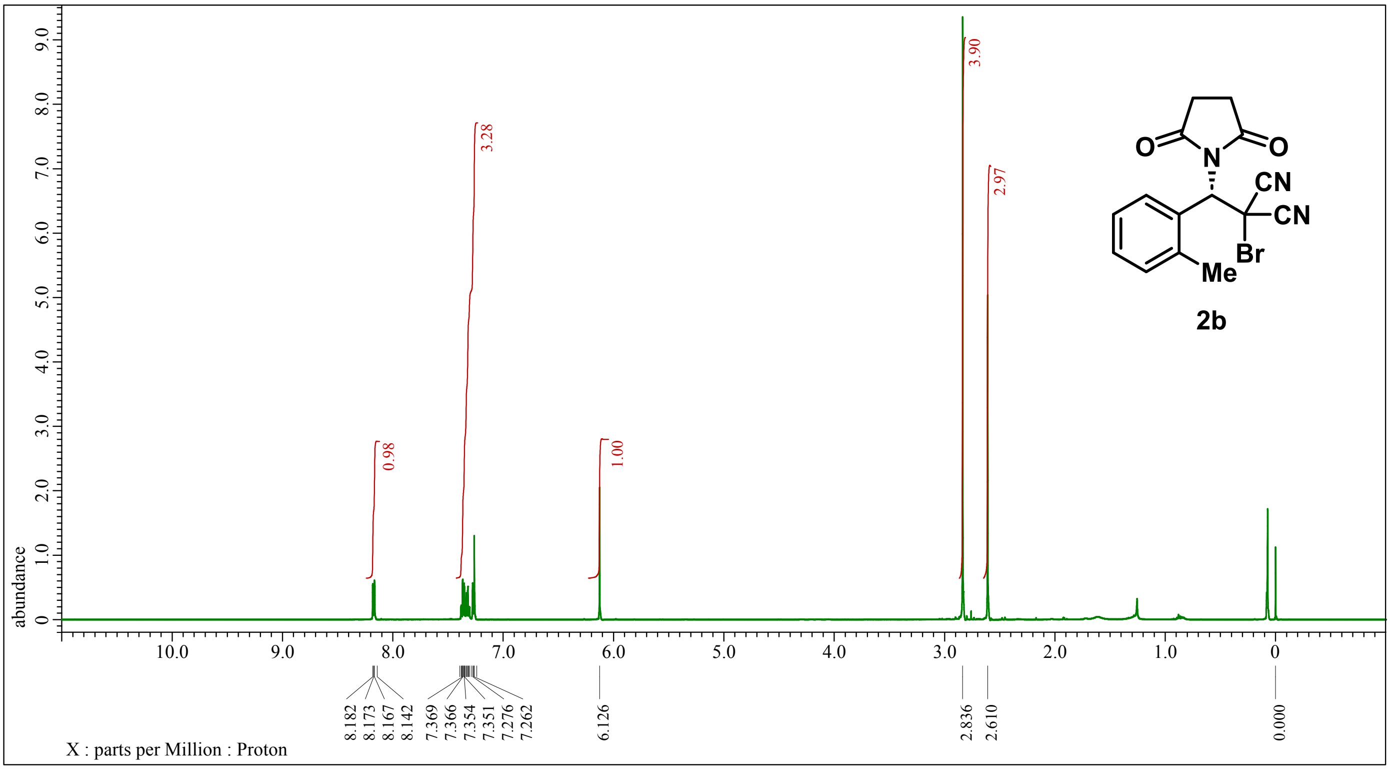


$^{13}$CNMR (125 MHz, chloroform-*d*) spectrum of 2b

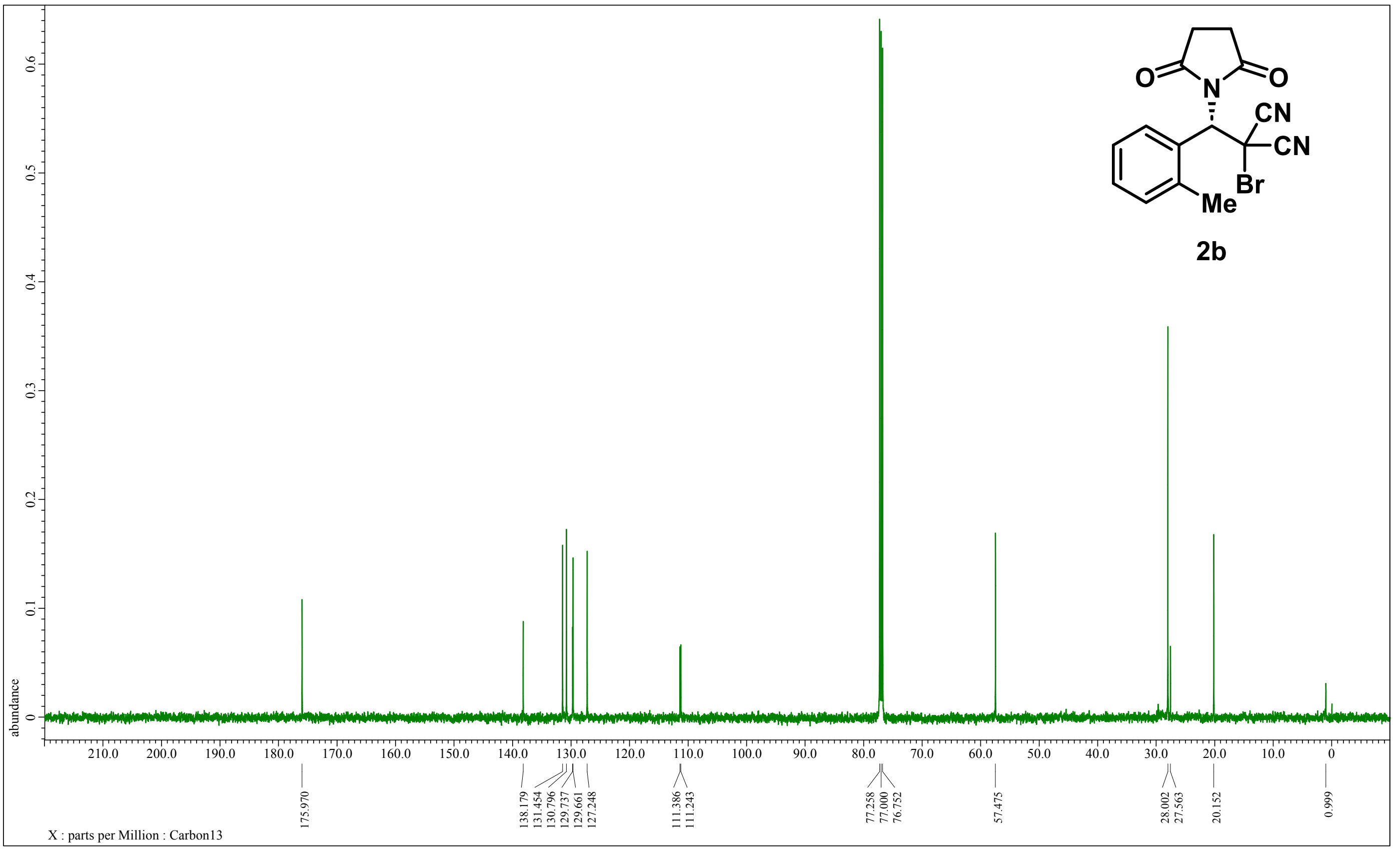

$^{1}$HNMR (500 MHz, chloroform-*d*) spectrum of 2c

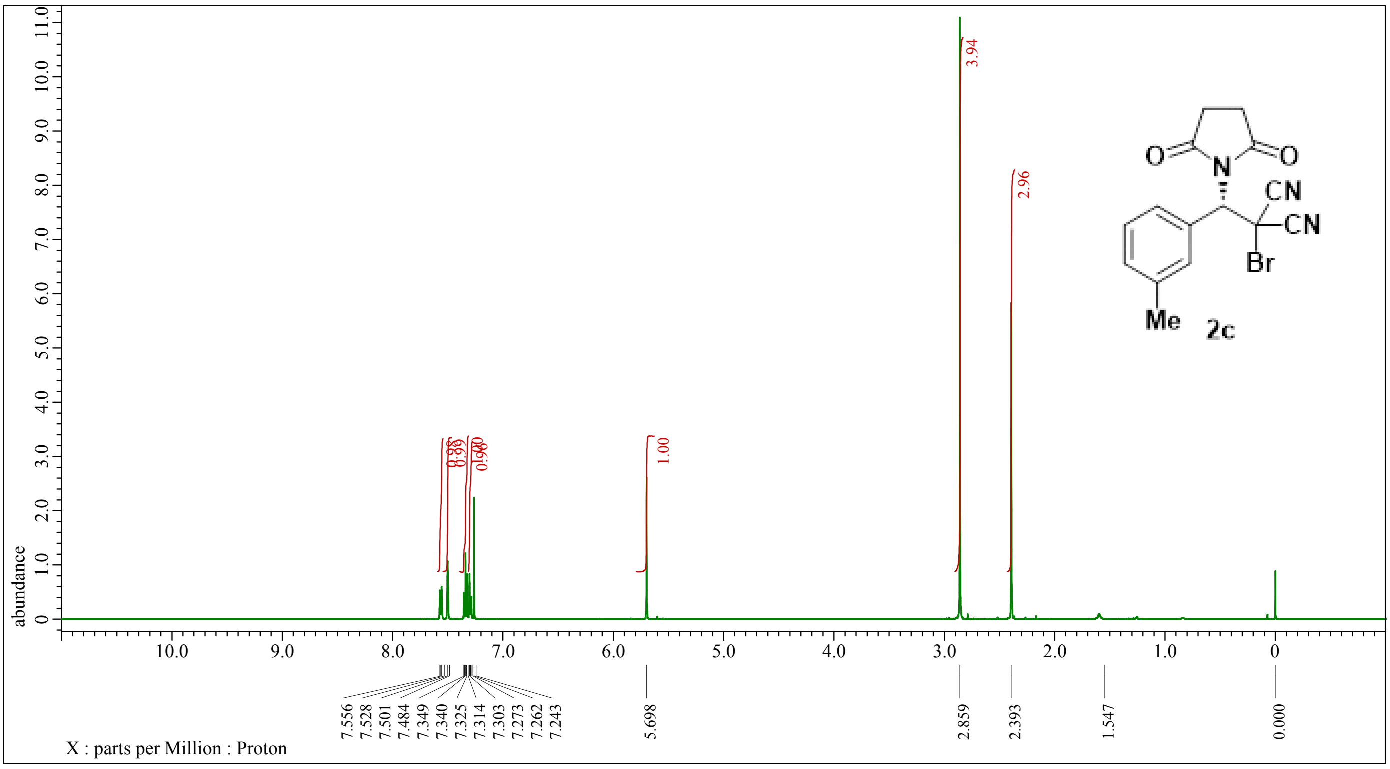


$^{13}$CNMR (125 MHz, chloroform-*d*) spectrum of 2c

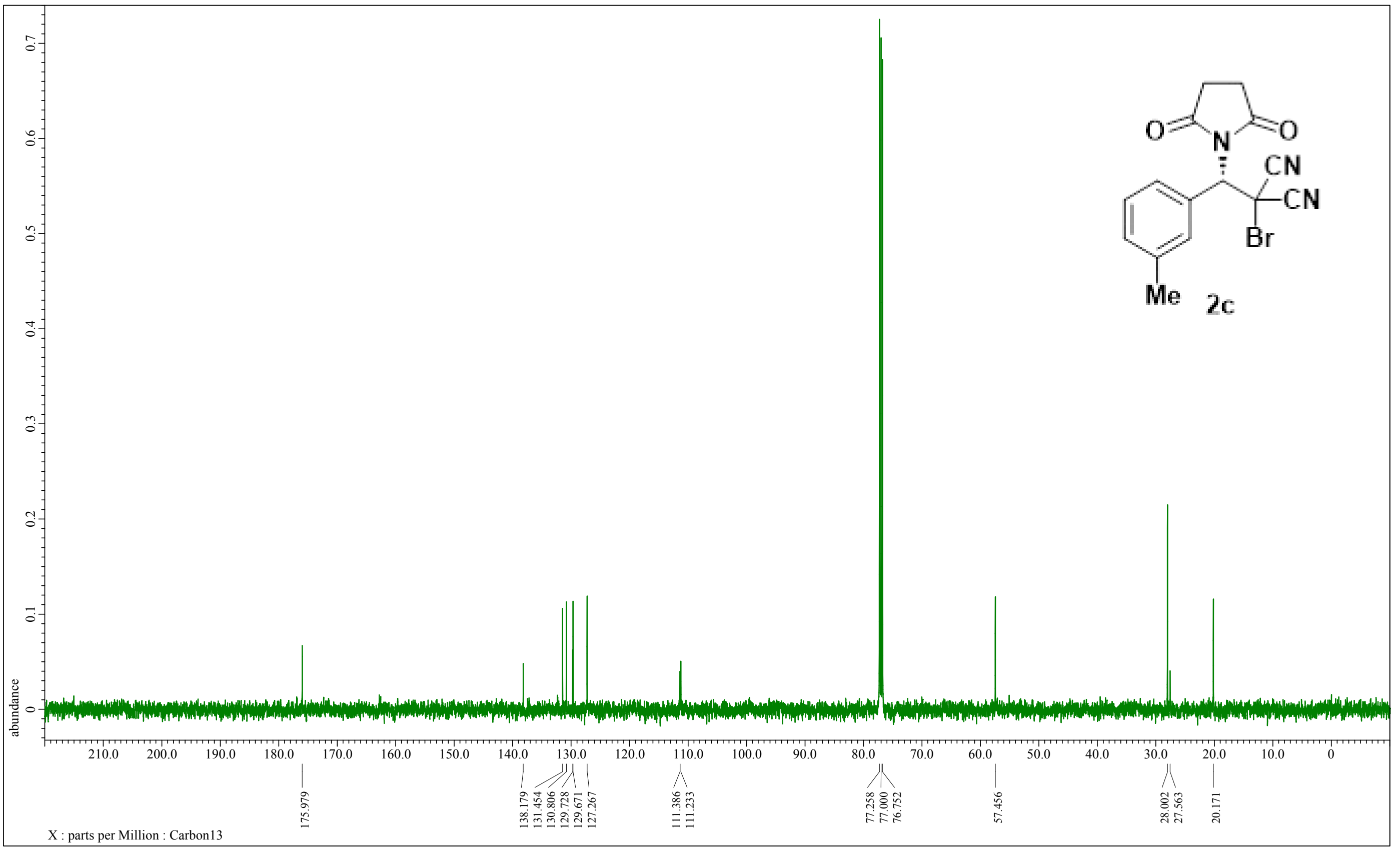

$^{1}$HNMR (500 MHz, chloroform-*d*) spectrum of 2d

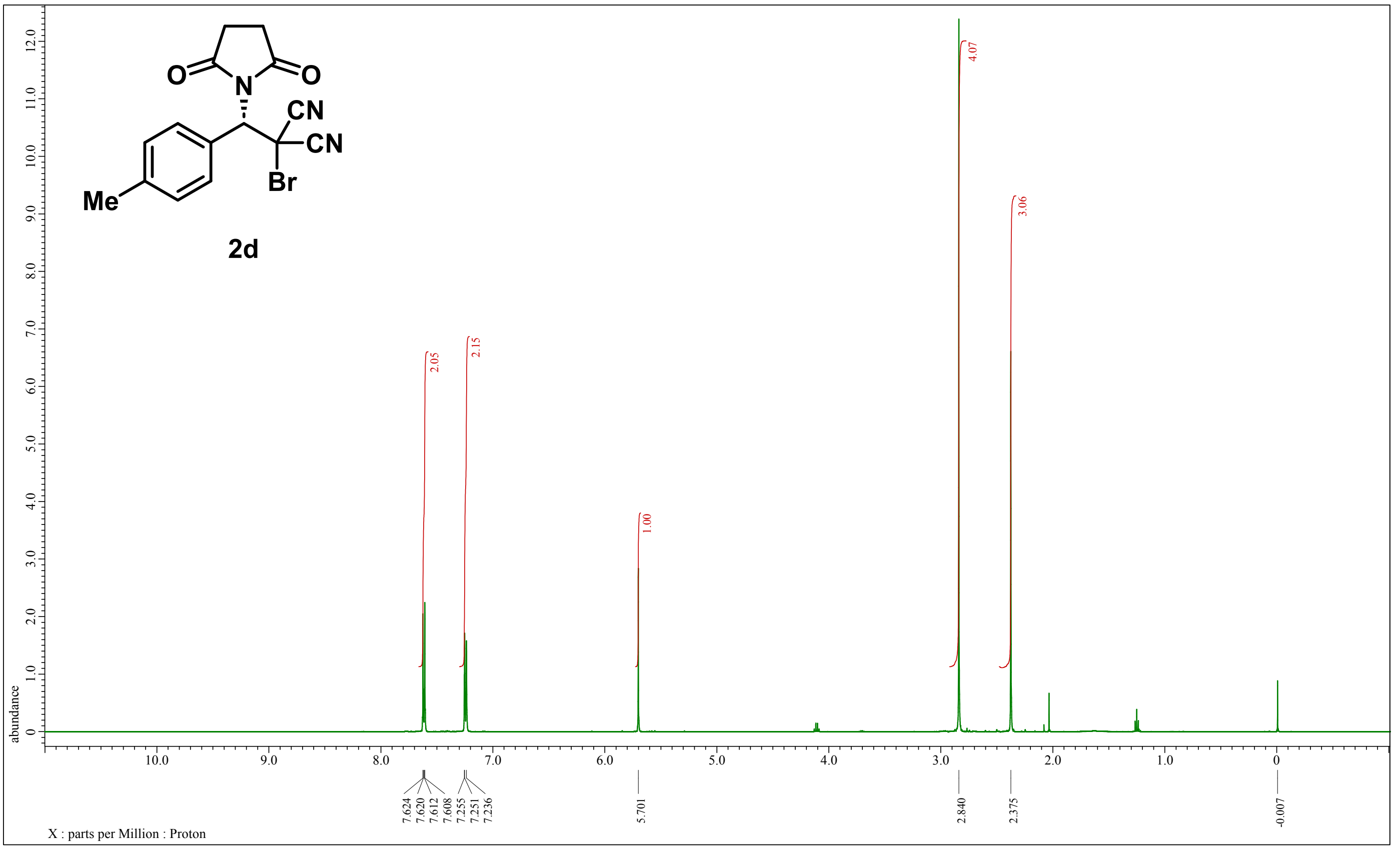


$^{13}$CNMR (125 MHz, chloroform-*d*) spectrum of 2d

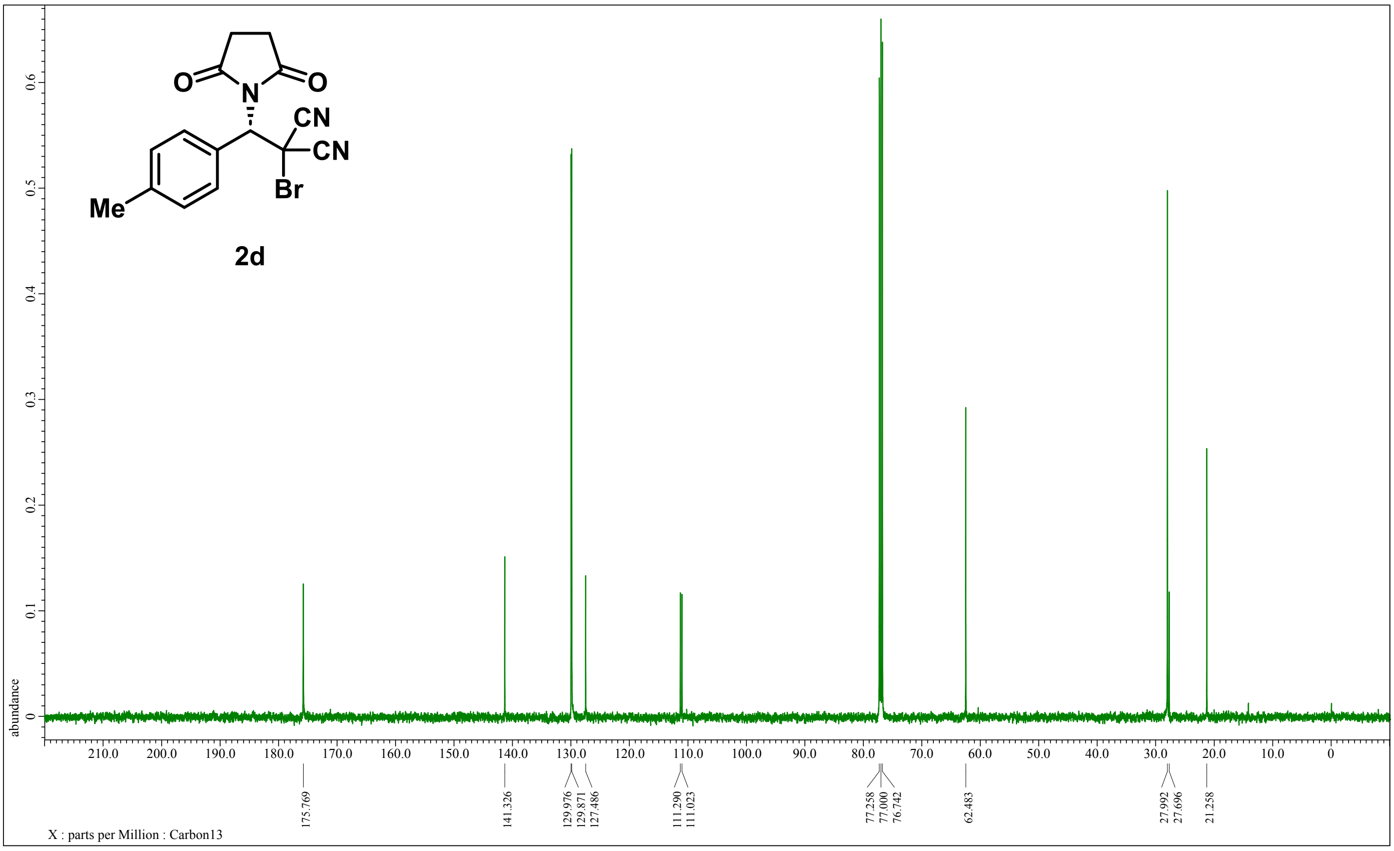

$^{1}$HNMR (500 MHz, chloroform-*d*) spectrum of 2e

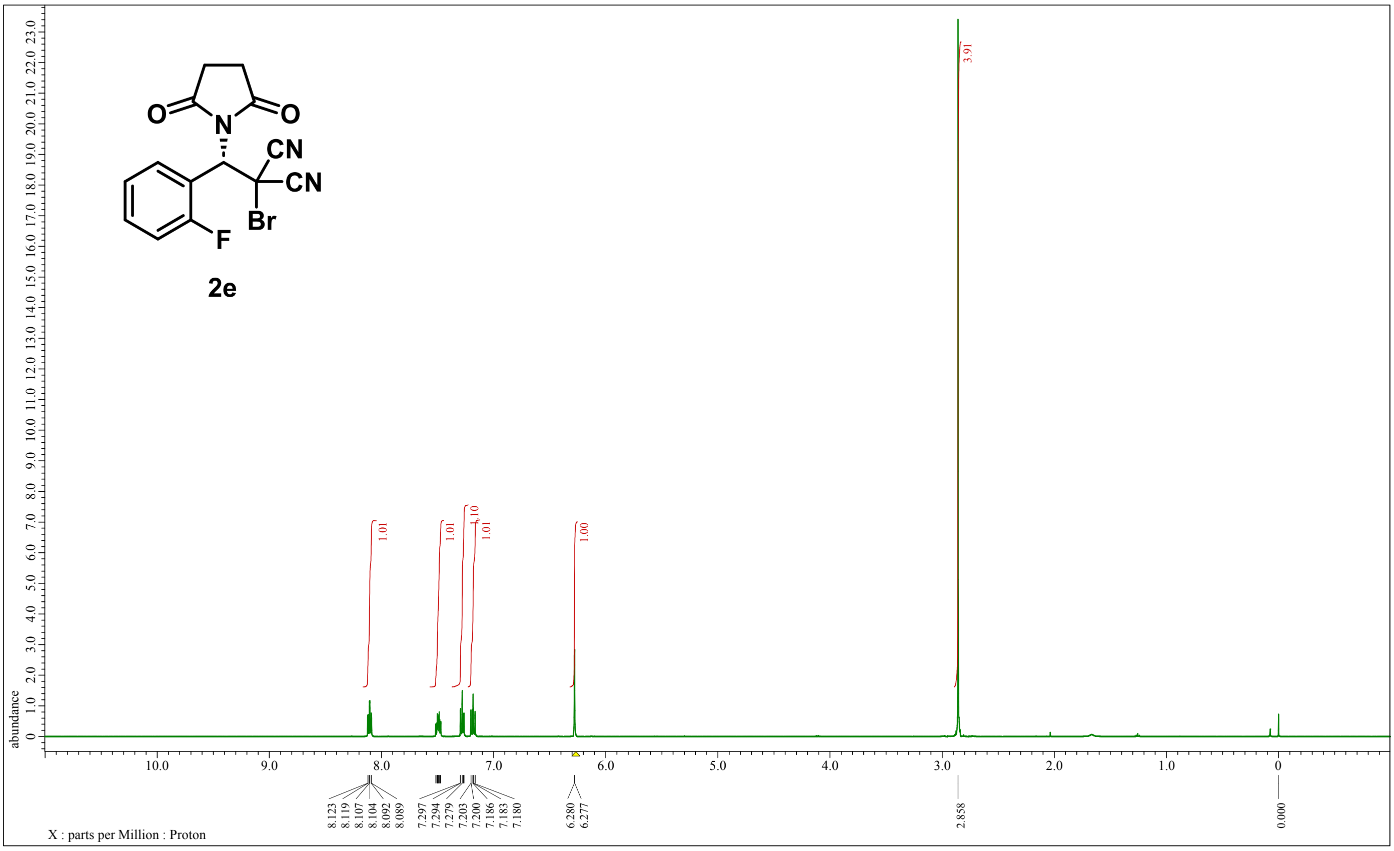


$^{13}$CNMR (125 MHz, chloroform-*d*) spectrum of 2e

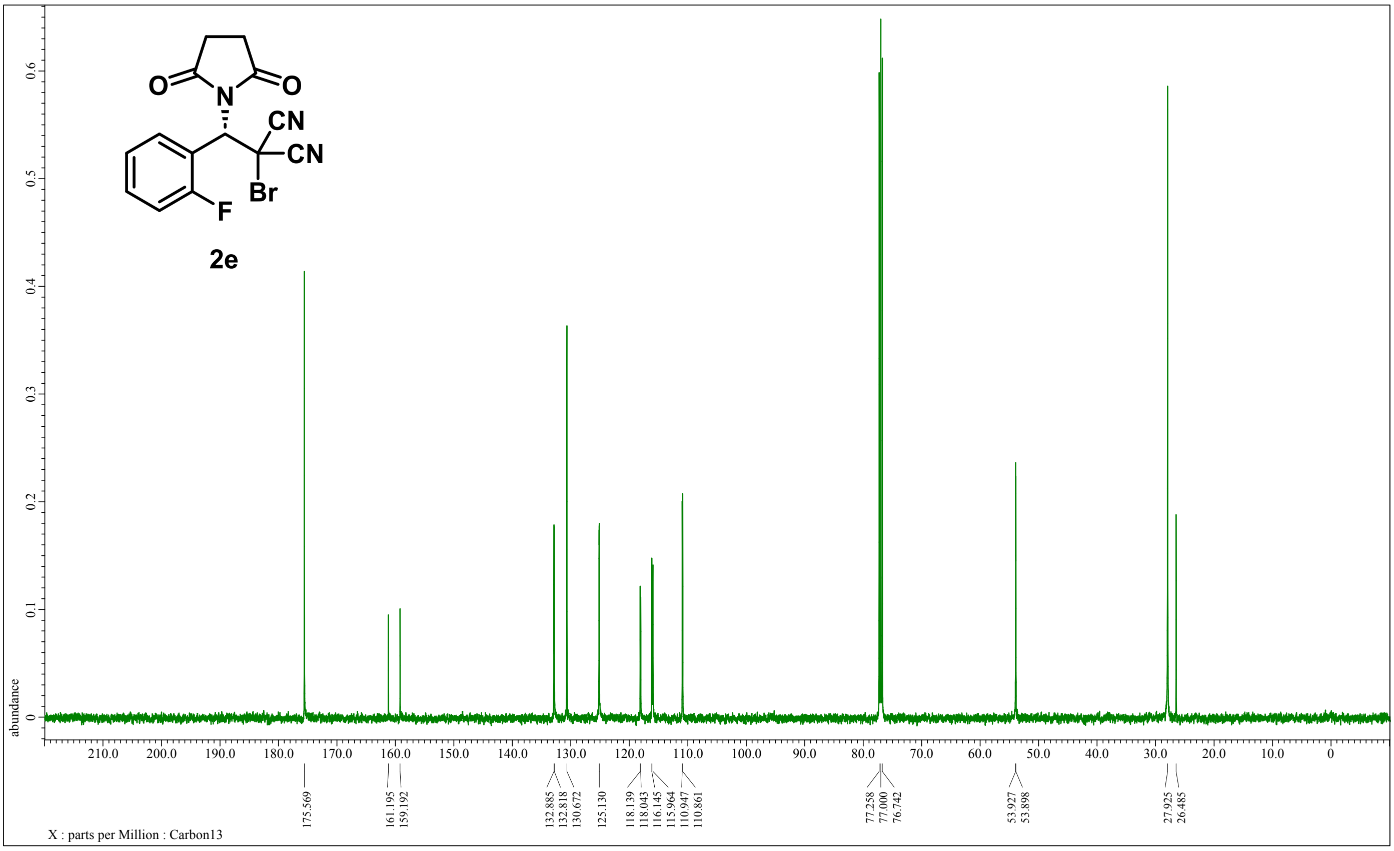

$^{1}$HNMR (500 MHz, chloroform-*d*) spectrum of 2f

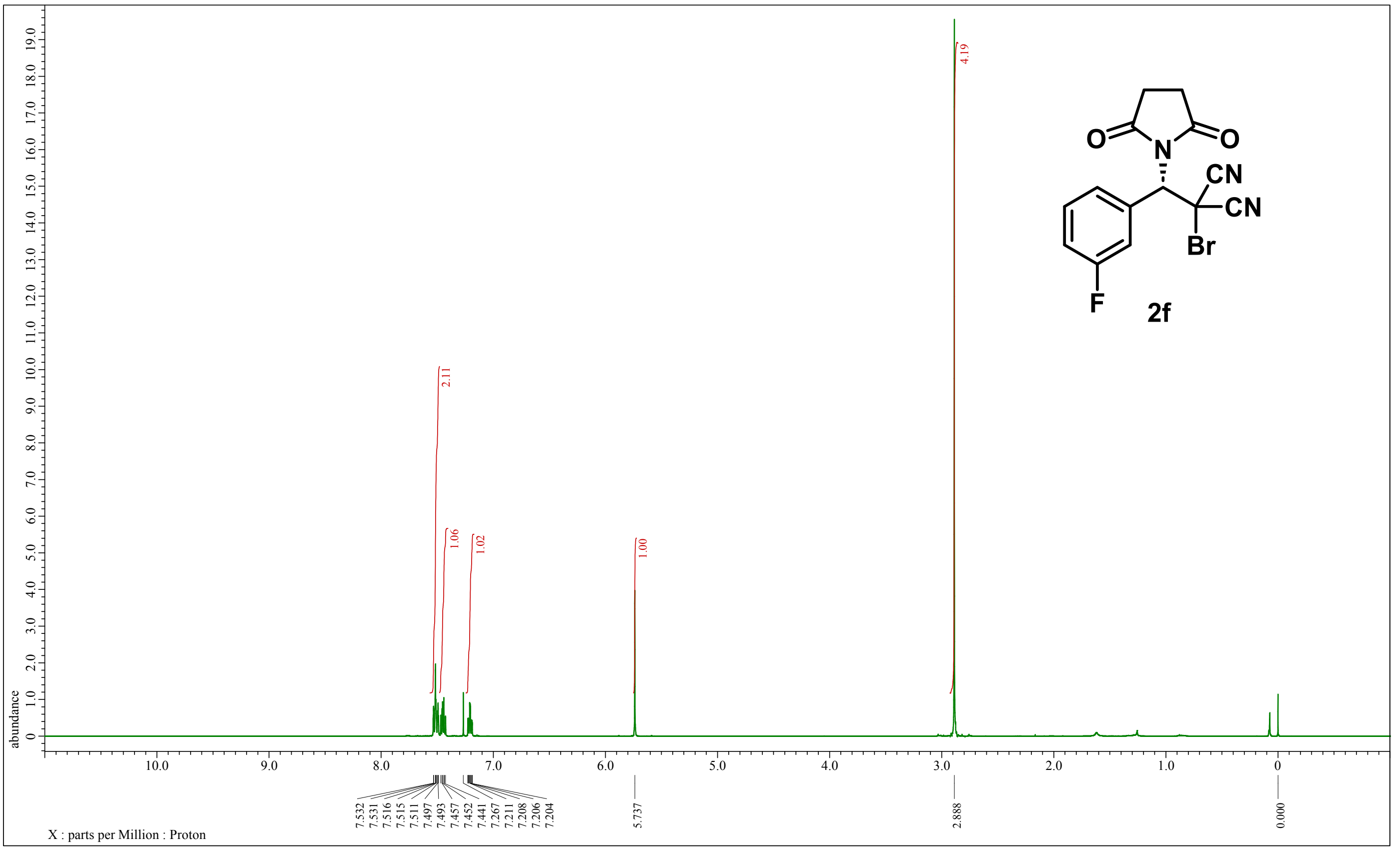


$^{13}$CNMR (125 MHz, chloroform-*d*) spectrum of 2f

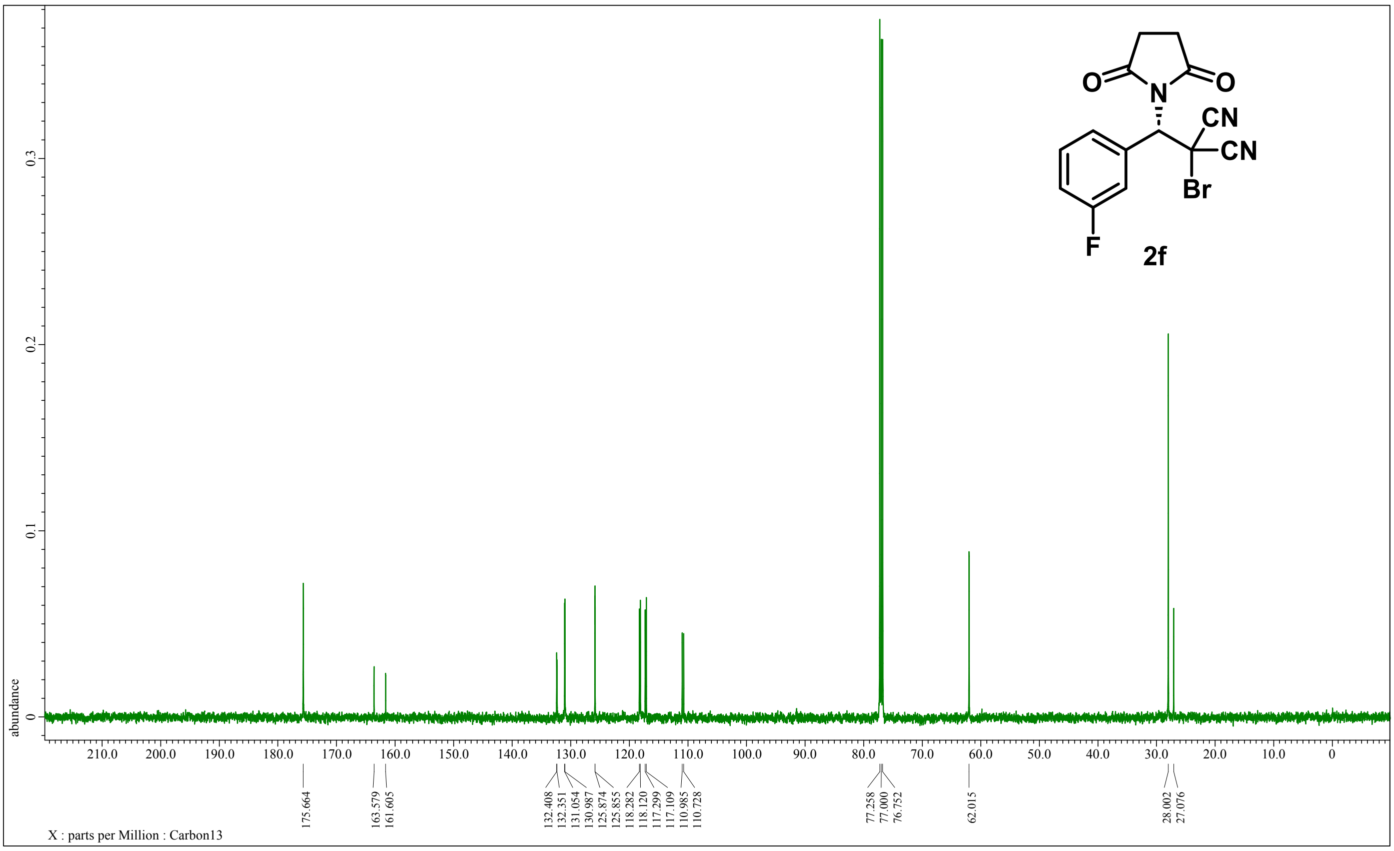

$^{1}$HNMR (500 MHz, chloroform-*d*) spectrum of 2g

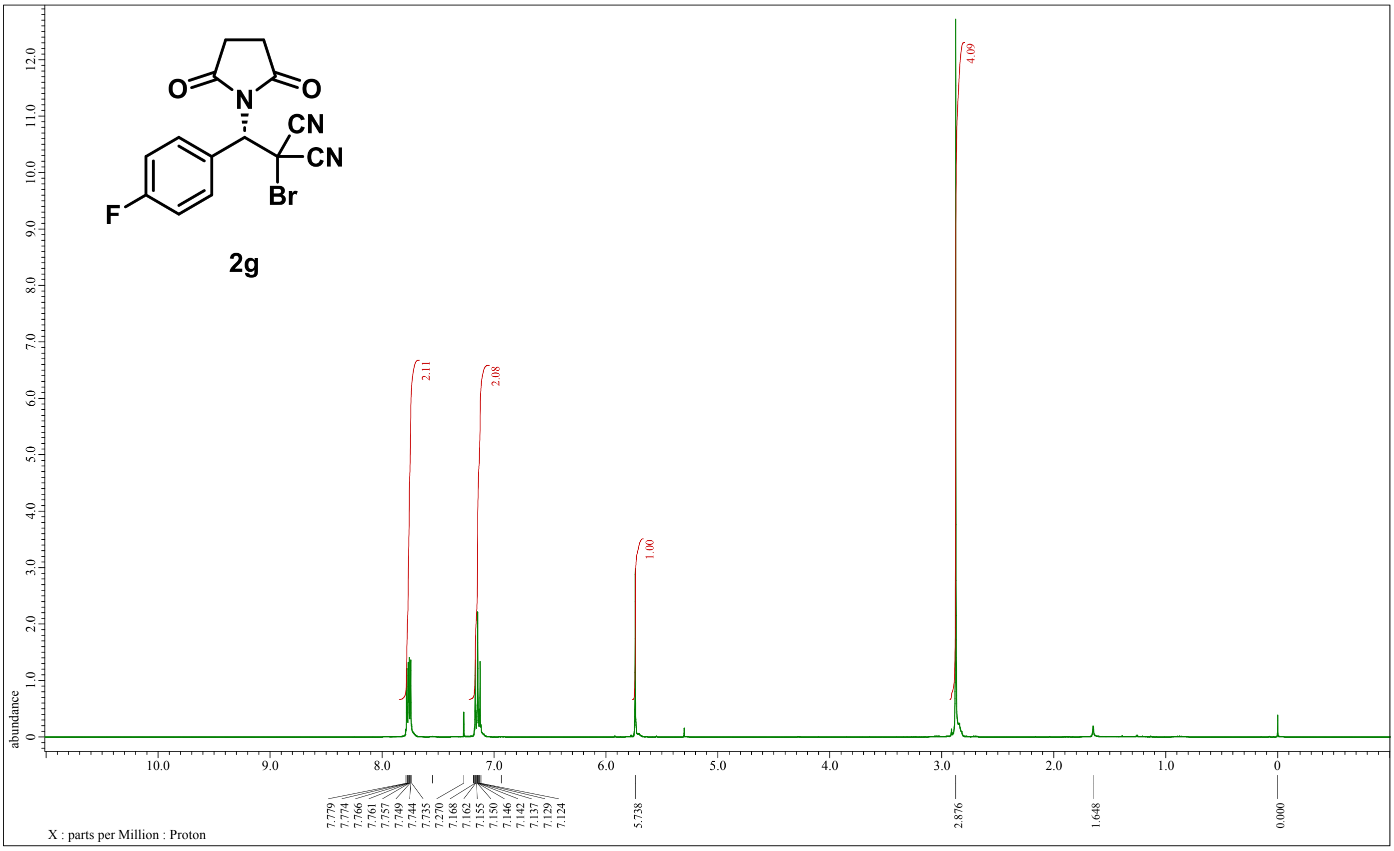


$^{13}$CNMR (125 MHz, chloroform-*d*) spectrum of 2g

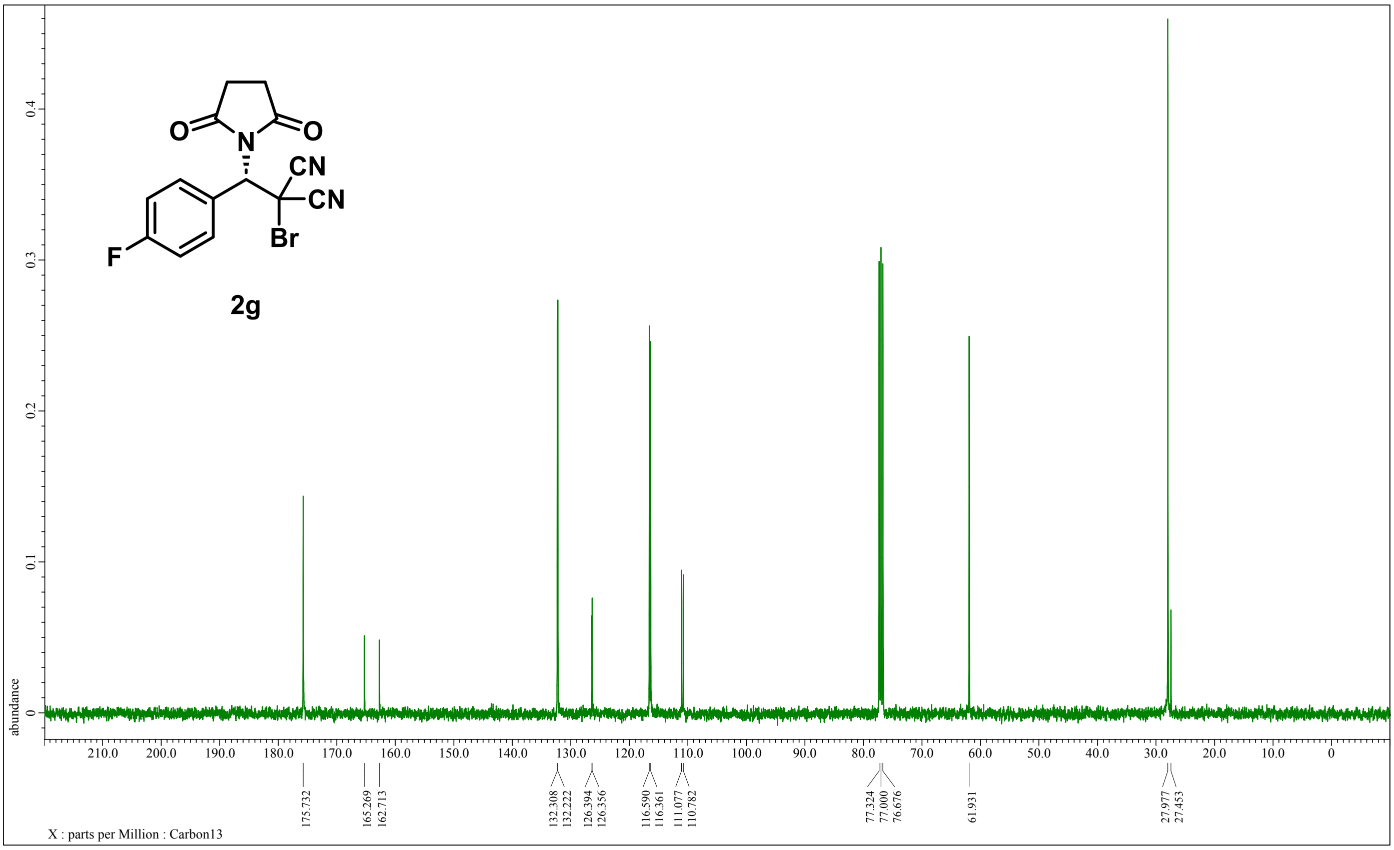

$^{1}$HNMR (500 MHz, chloroform-*d*) spectrum of 2h

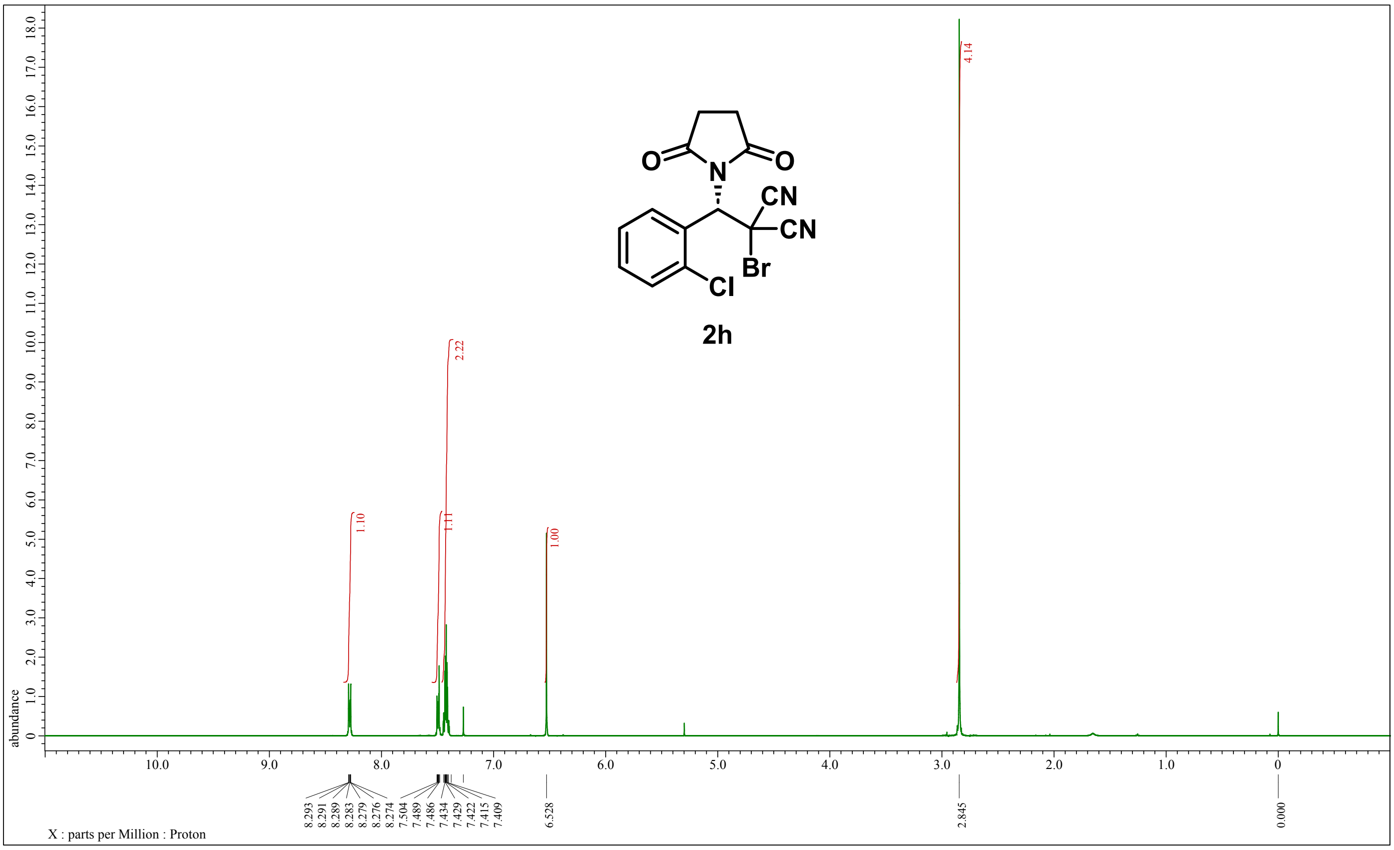


$^{13}$CNMR (125 MHz, chloroform-*d*) spectrum of 2h

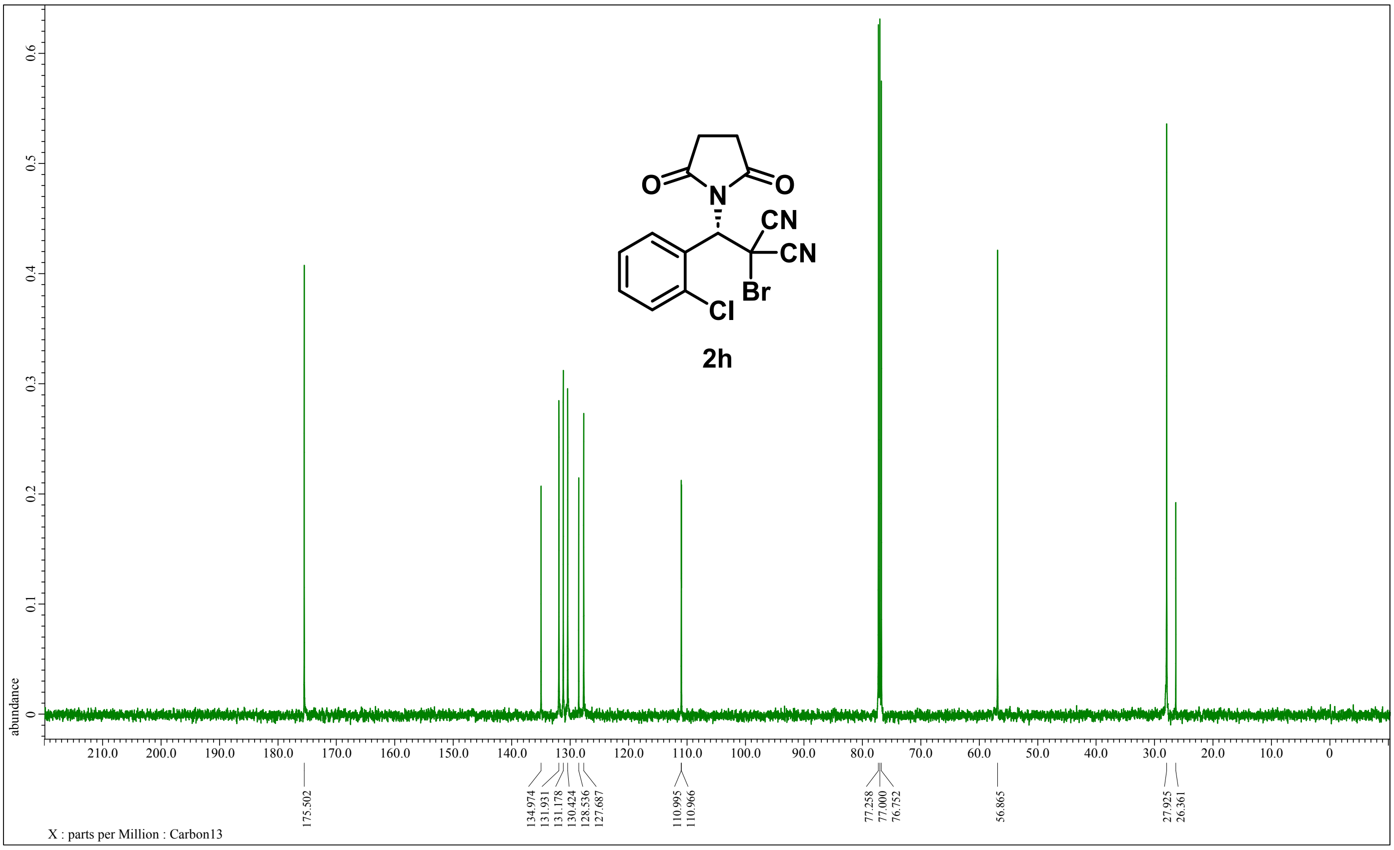

$^{1}$HNMR (500 MHz, chloroform-*d*) spectrum of 2i

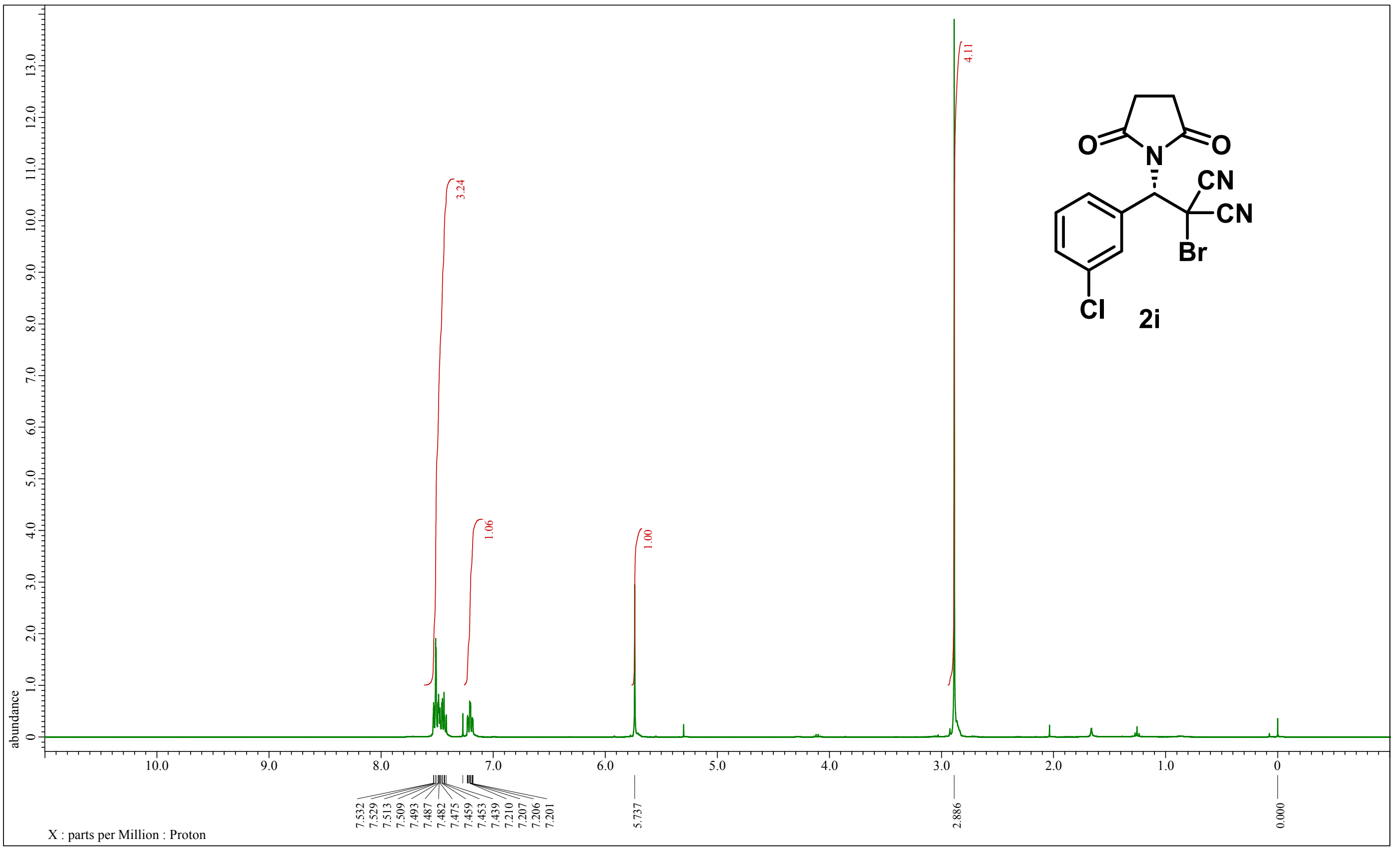


$^{13}$CNMR (125 MHz, chloroform-*d*) spectrum of 2i

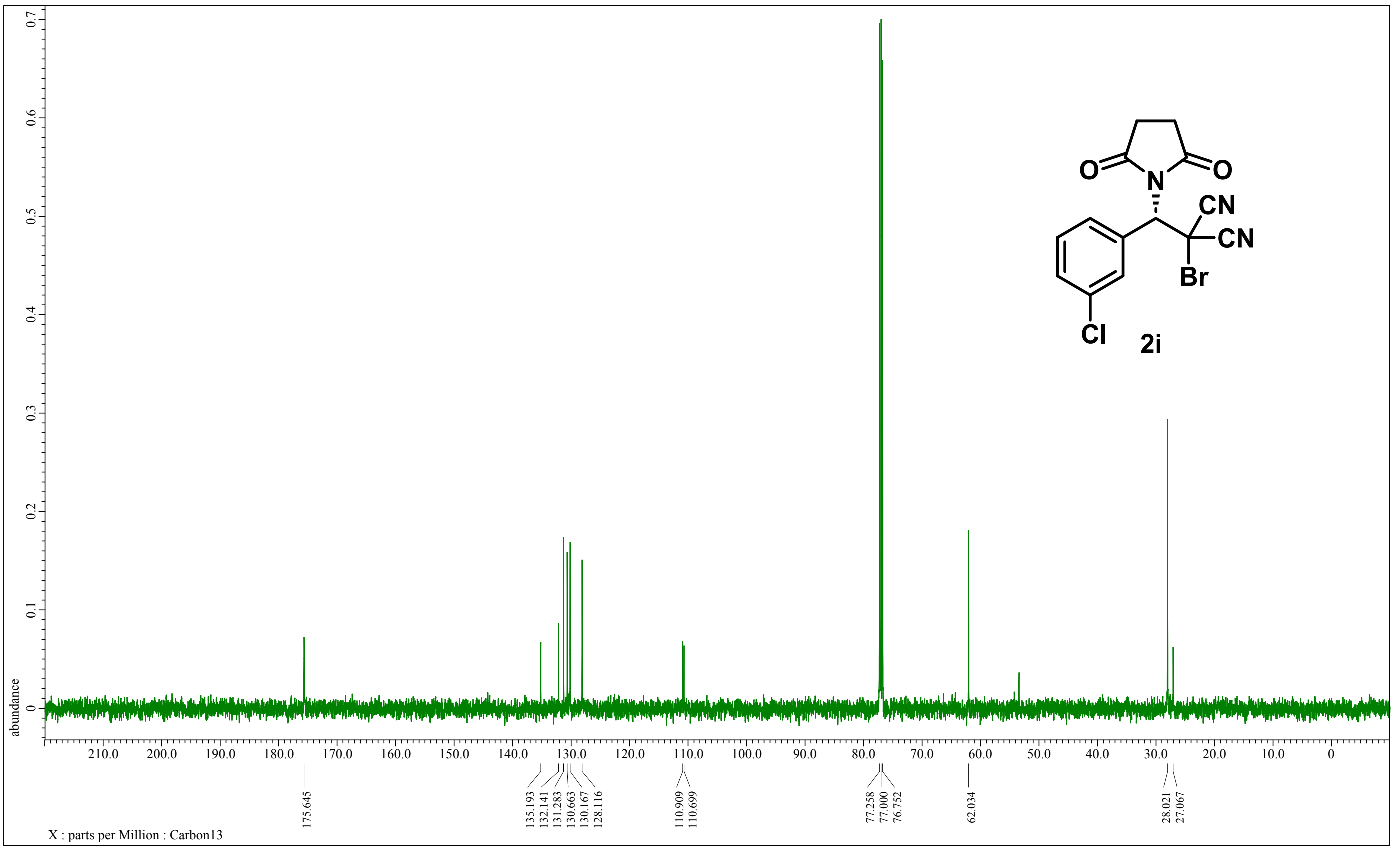

$^{1}$HNMR (400 MHz, chloroform-*d*) spectrum of 2j

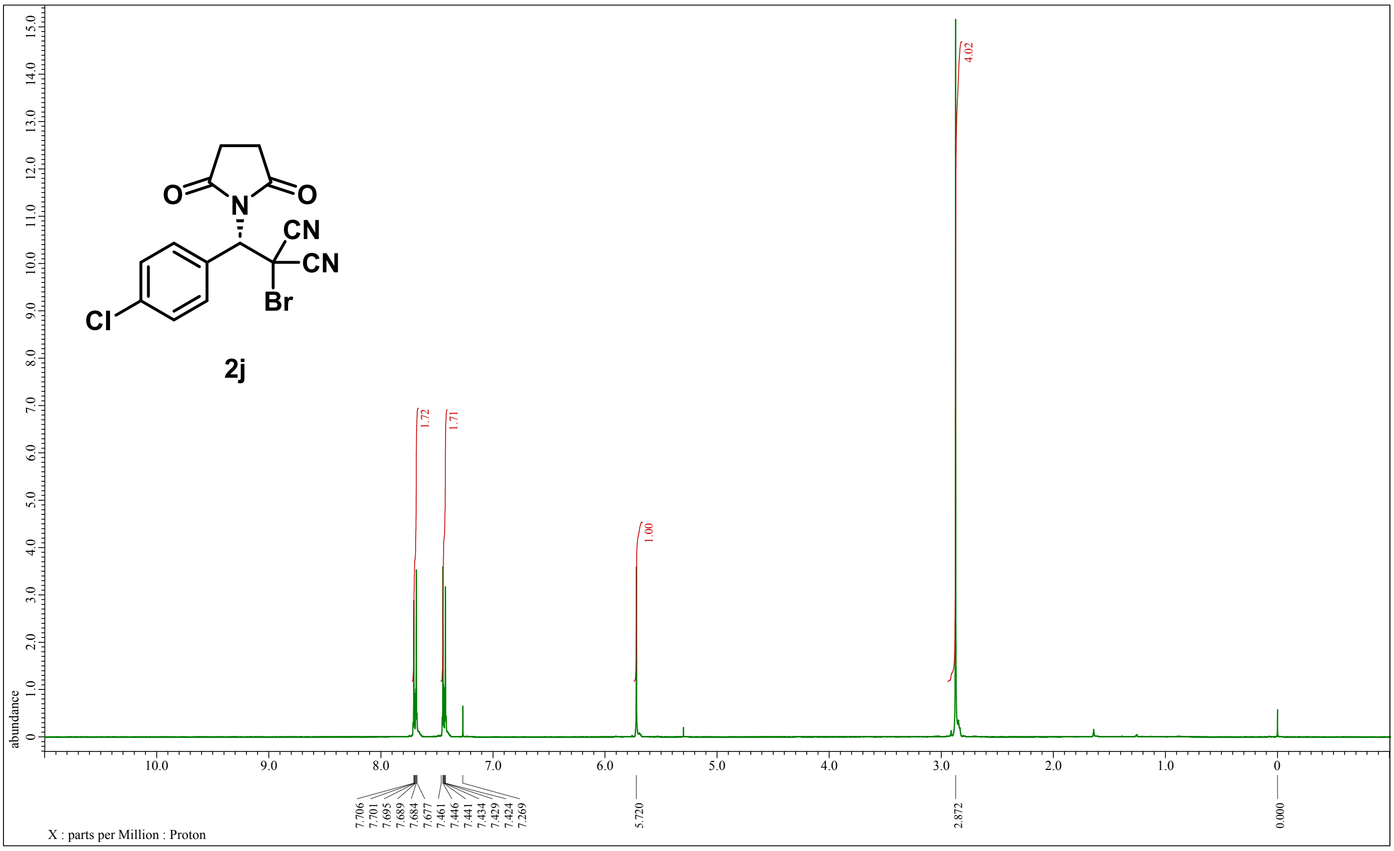


$^{13}$CNMR (125 MHz, chloroform-*d*) spectrum of 2j

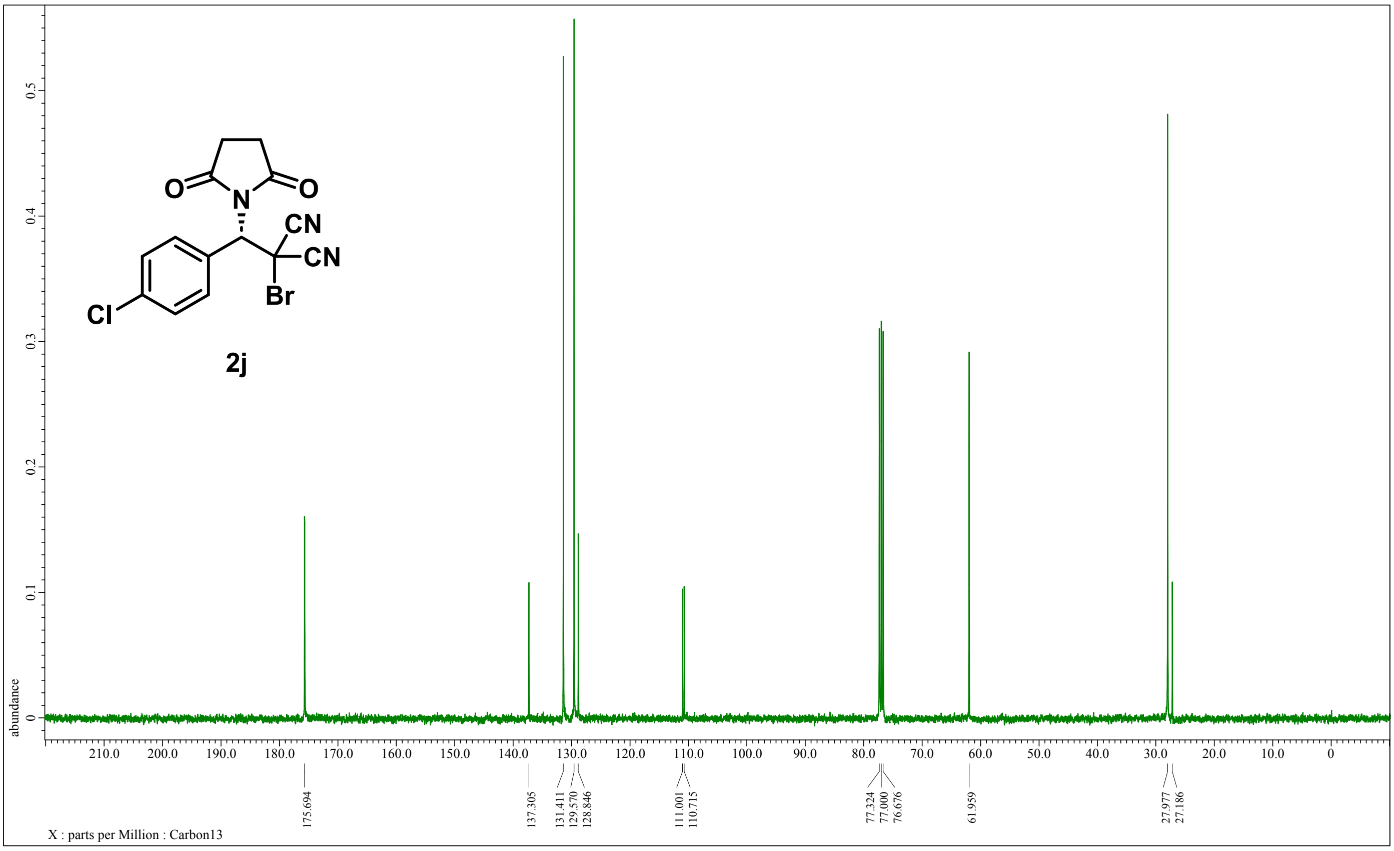

$^{1}$HNMR (500 MHz, chloroform-*d*) spectrum of 2k

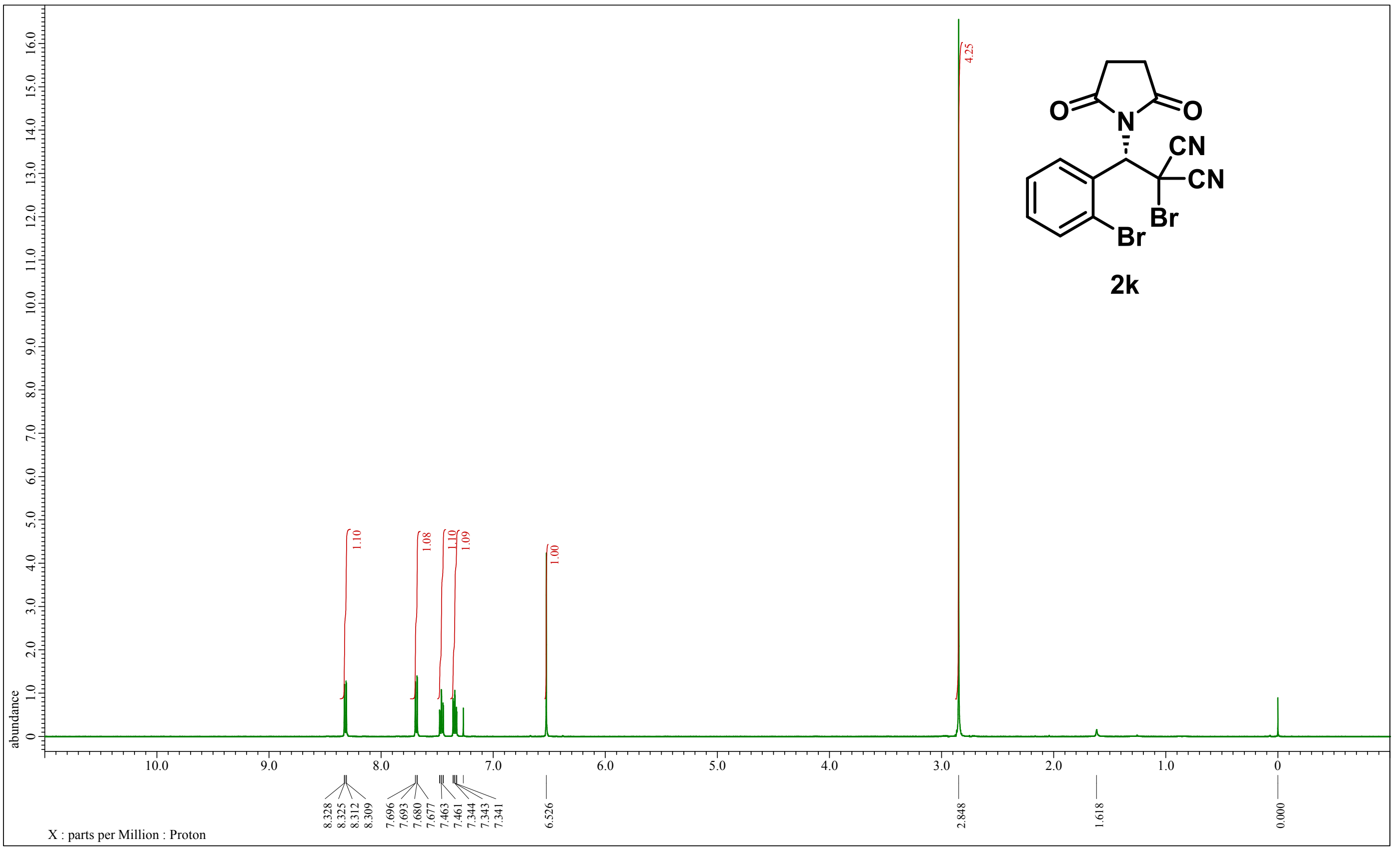

$^{13}$CNMR (125 MHz, chloroform-*d*) spectrum of 2k

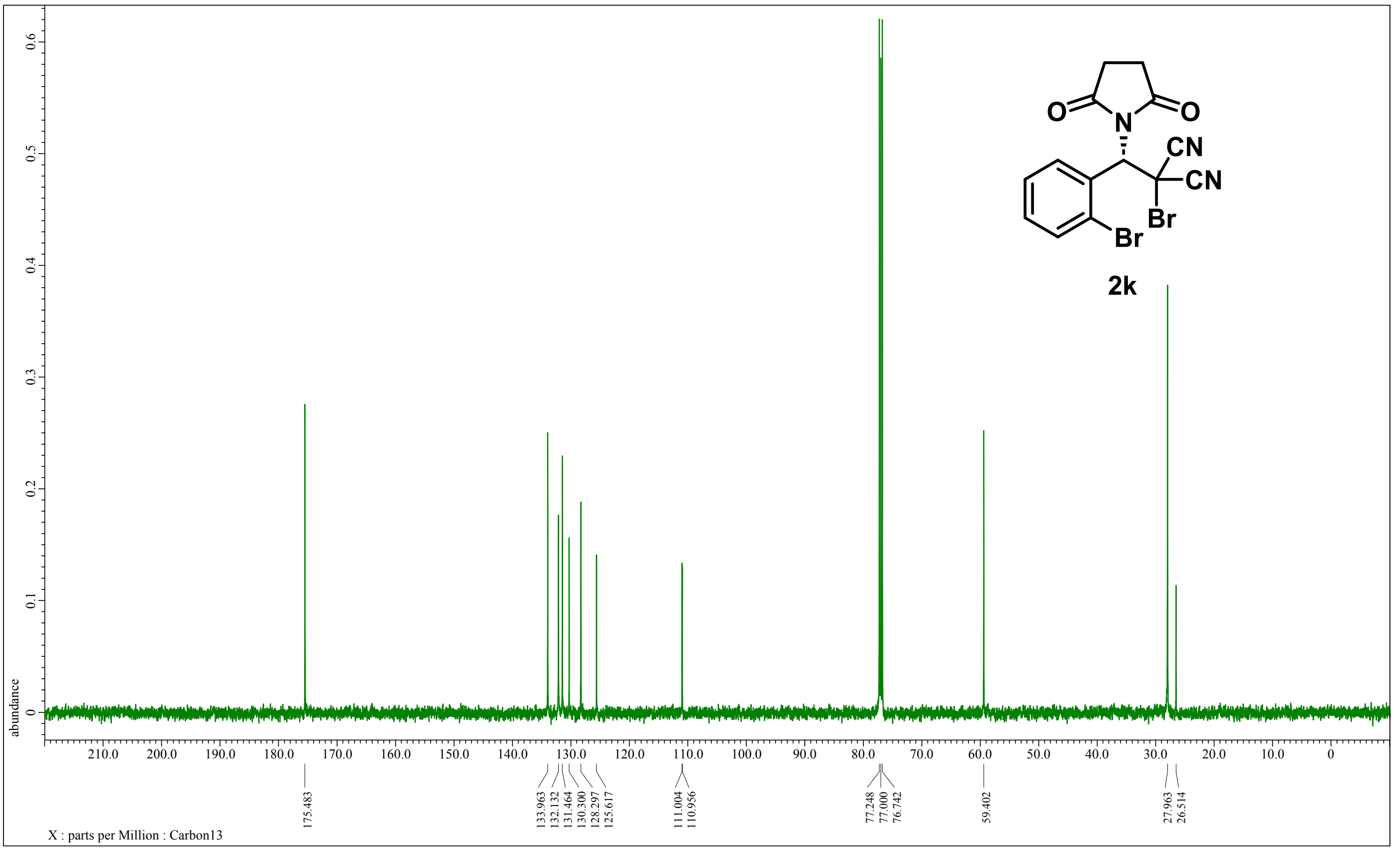

$^{1}$HNMR (400 MHz, chloroform-*d*) spectrum of 2l

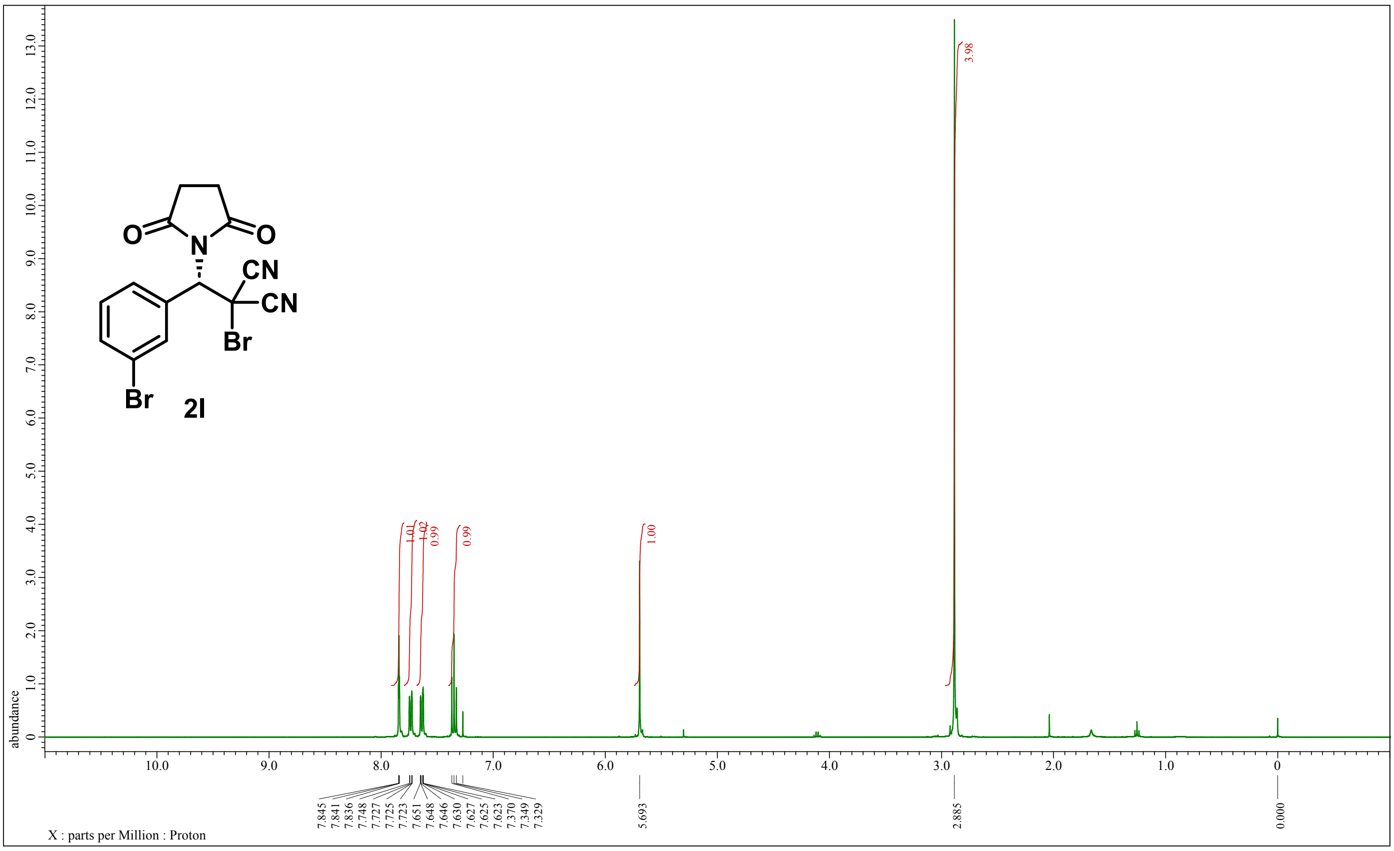


$^{13}$CNMR (125 MHz, chloroform-*d*) spectrum of 2l

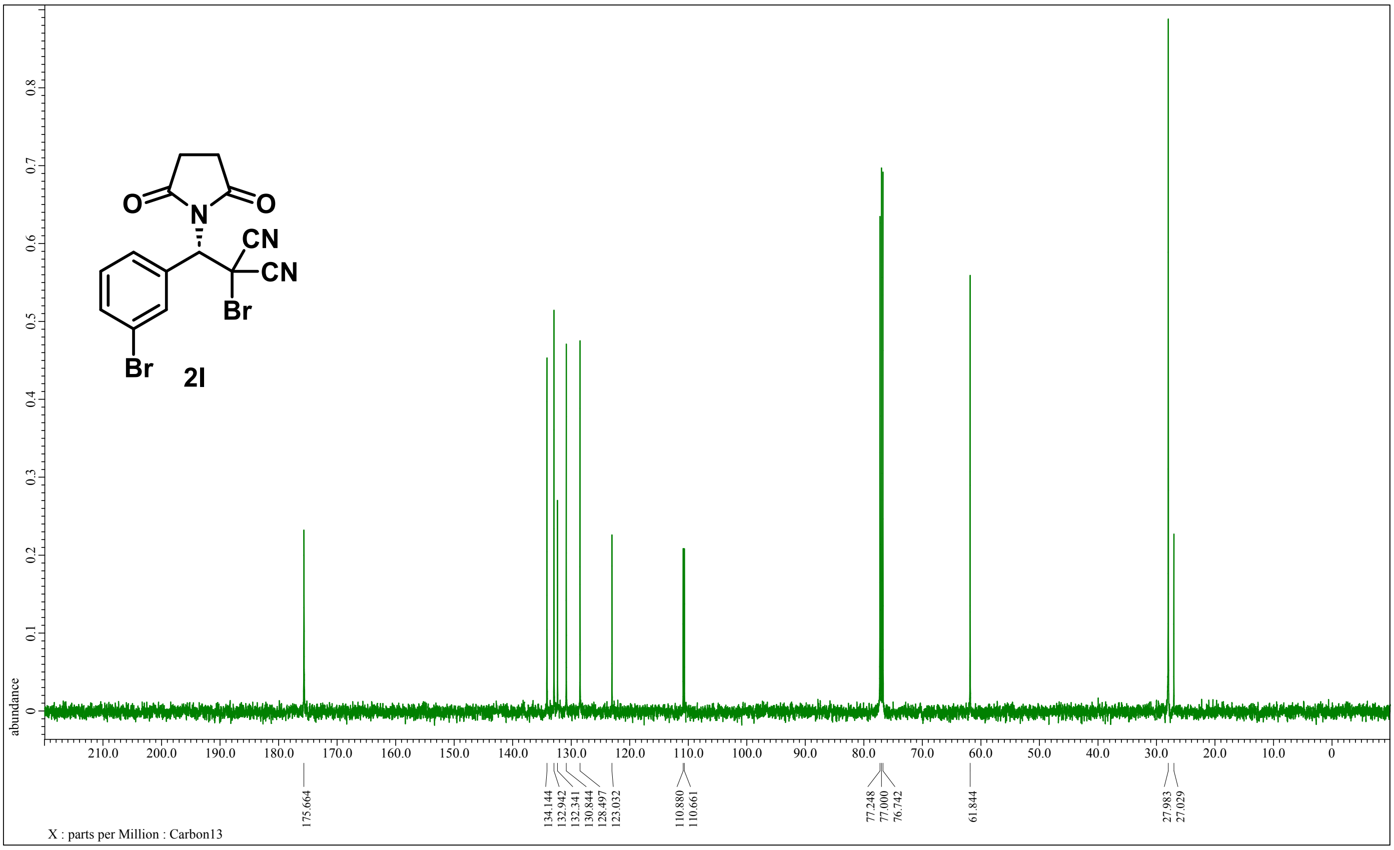

$^{1}$HNMR (400 MHz, chloroform-*d*) spectrum of 2m

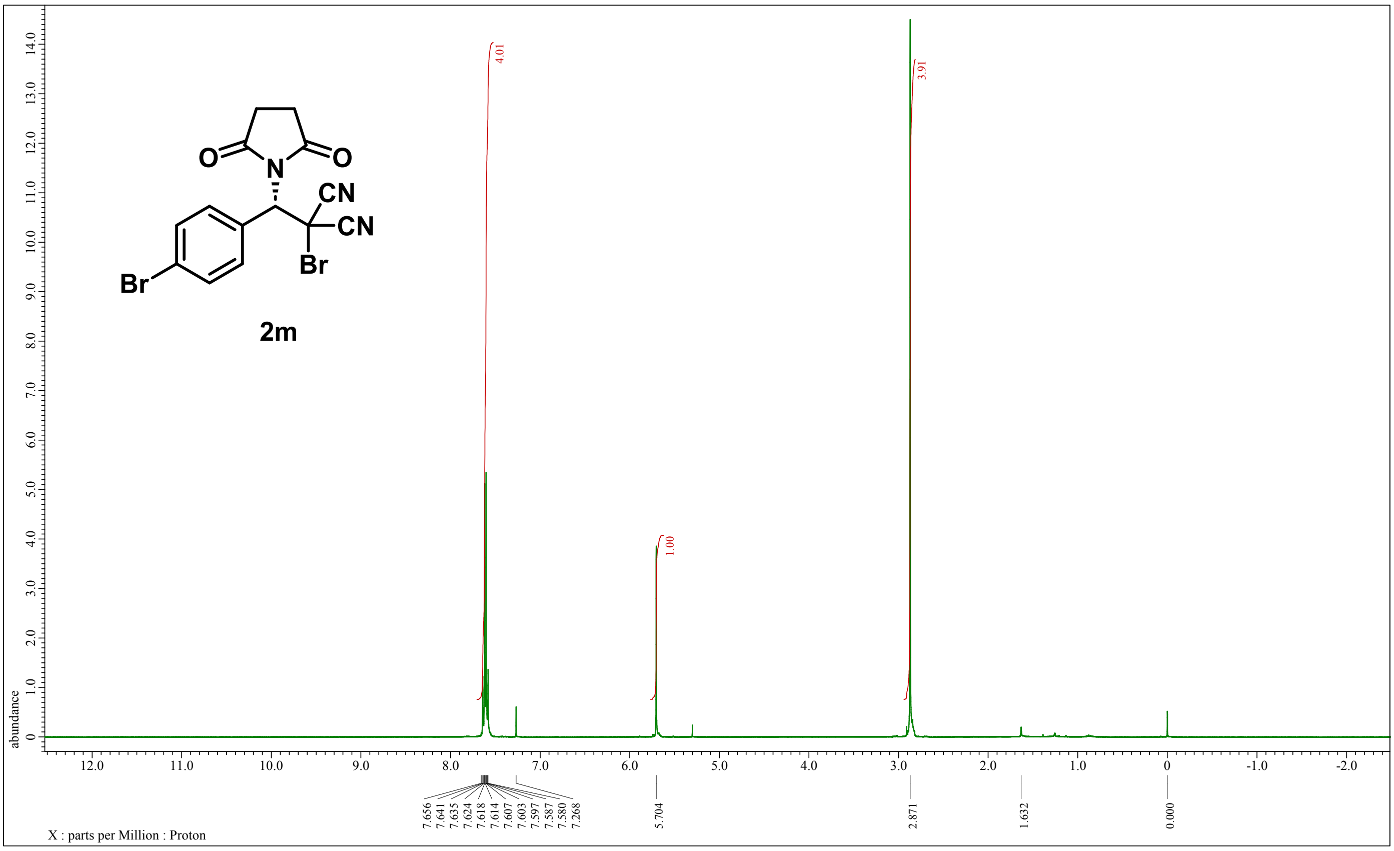


$^{13}$CNMR (125 MHz, chloroform-*d*) spectrum of 2m

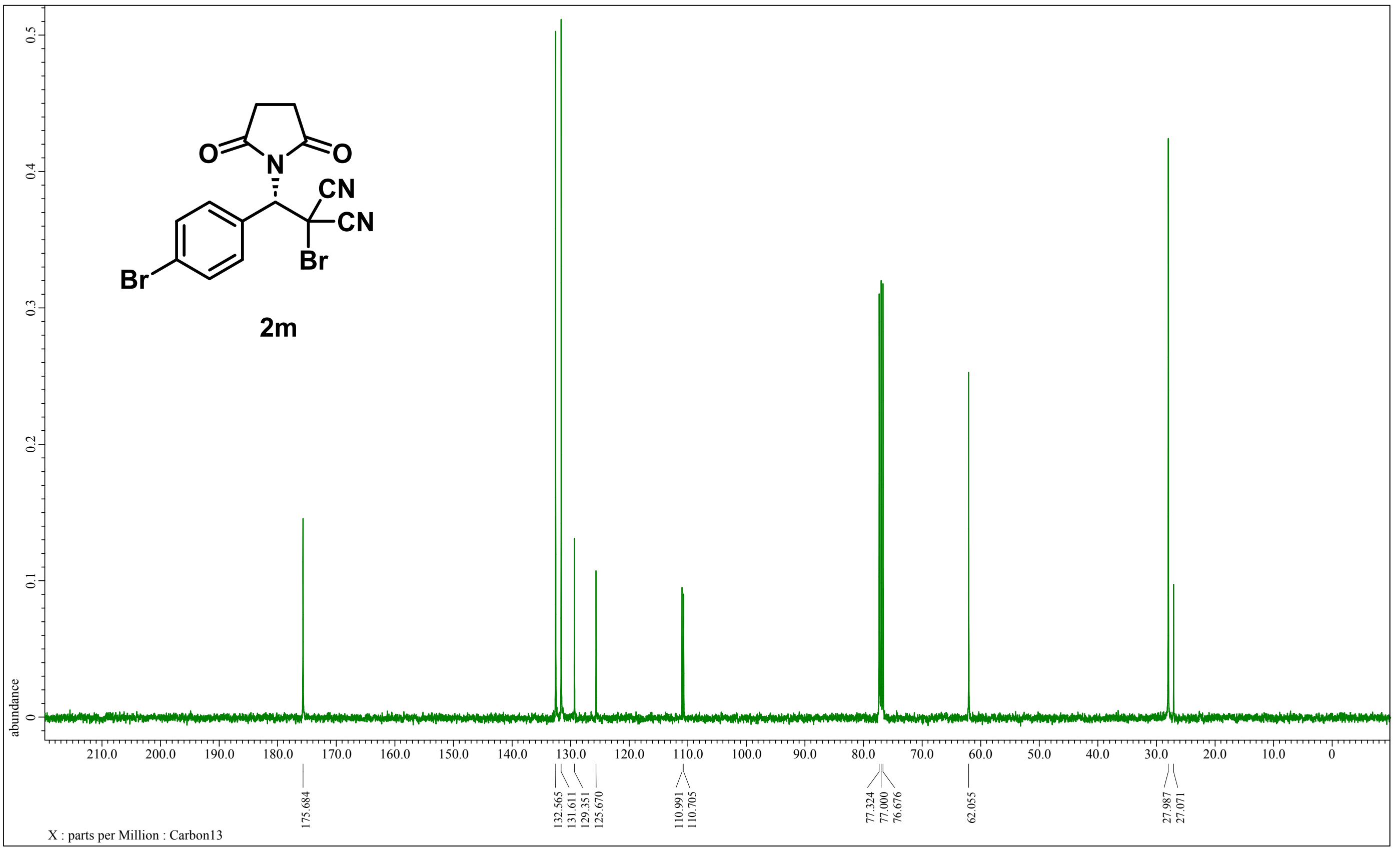

$^{1}$HNMR (500 MHz, chloroform-*d*) spectrum of 2n

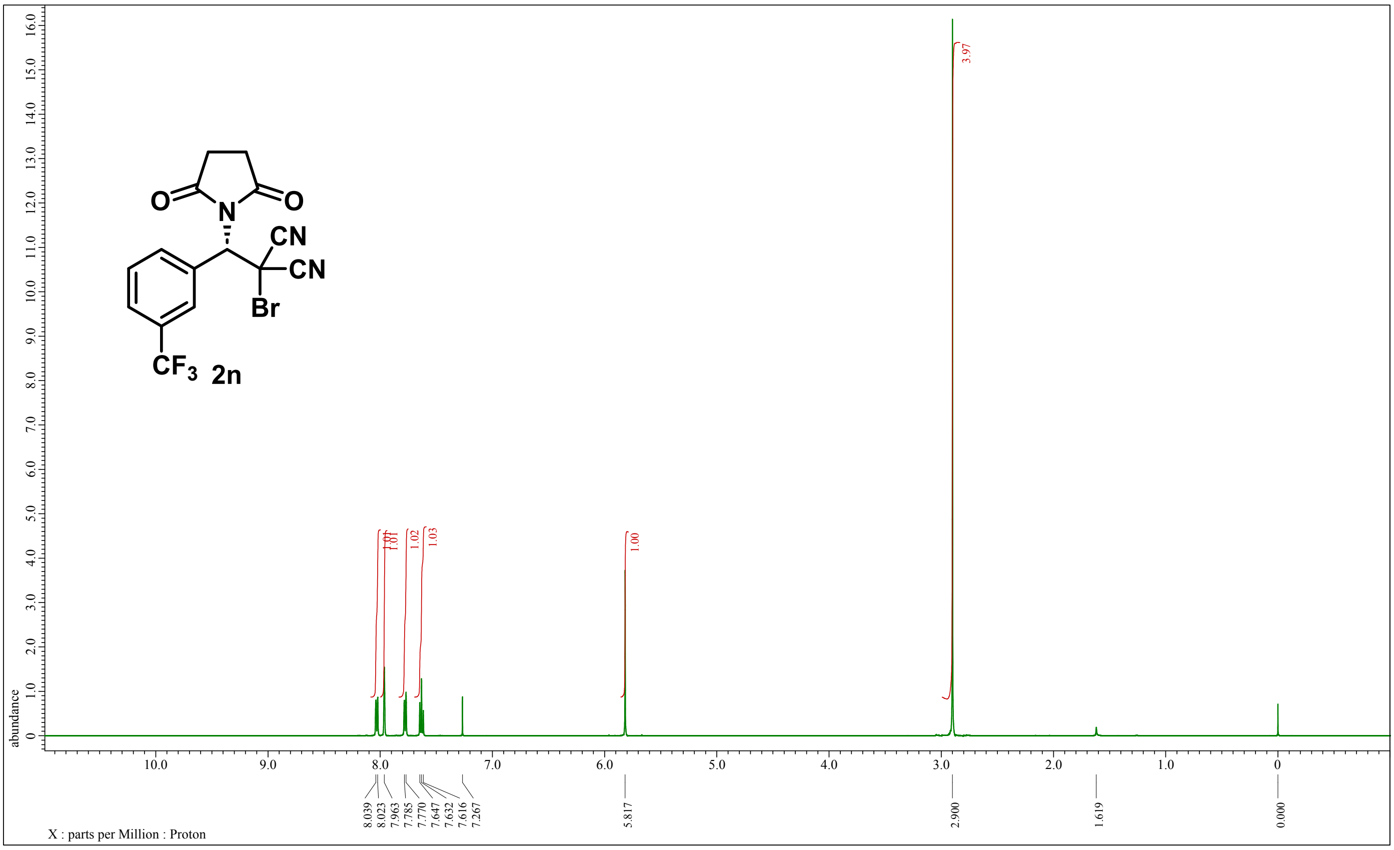


$^{13}$CNMR (125 MHz, chloroform-*d*) spectrum of 2n

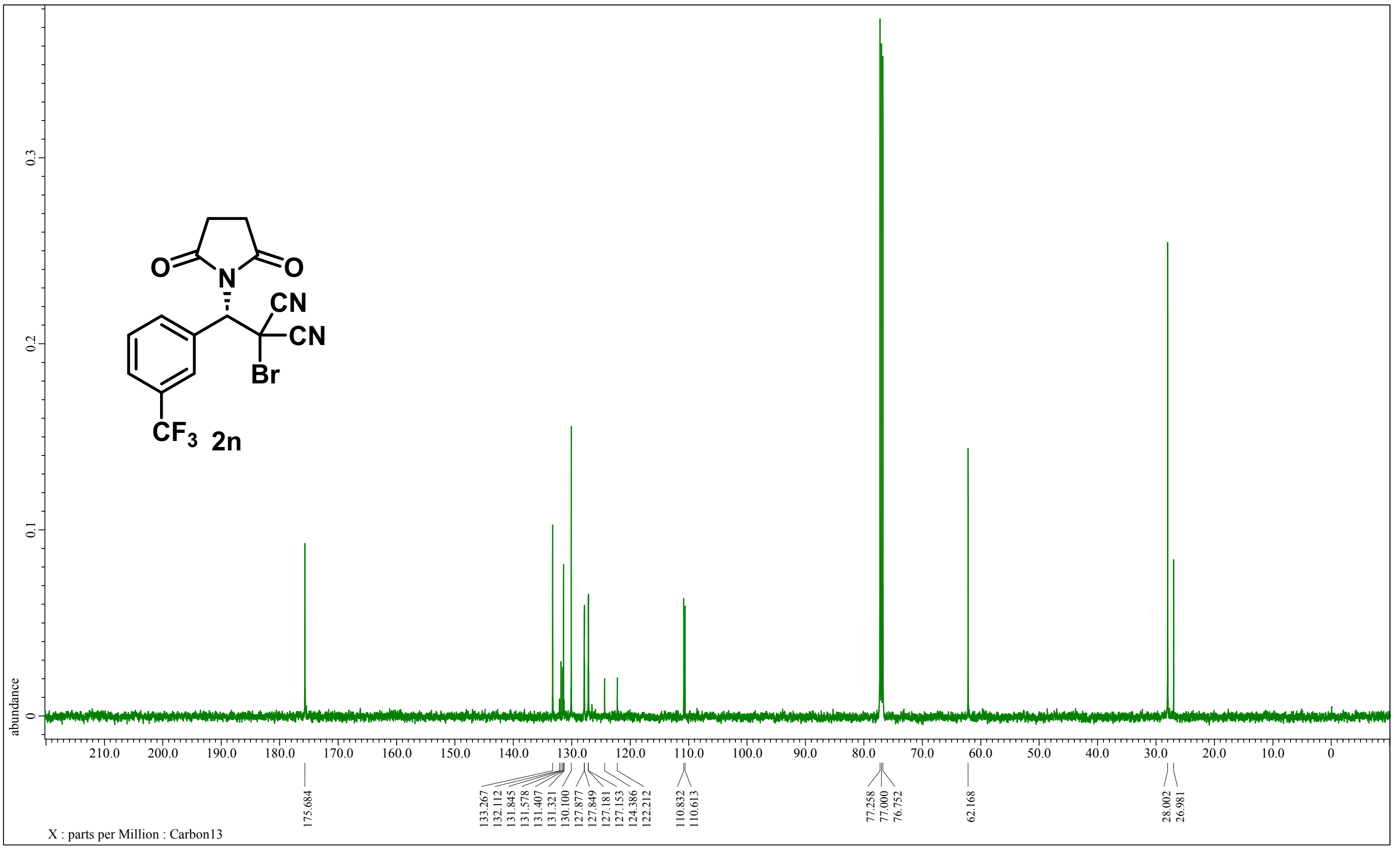

$^{1}$HNMR (500 MHz, chloroform-*d*) spectrum of 2o

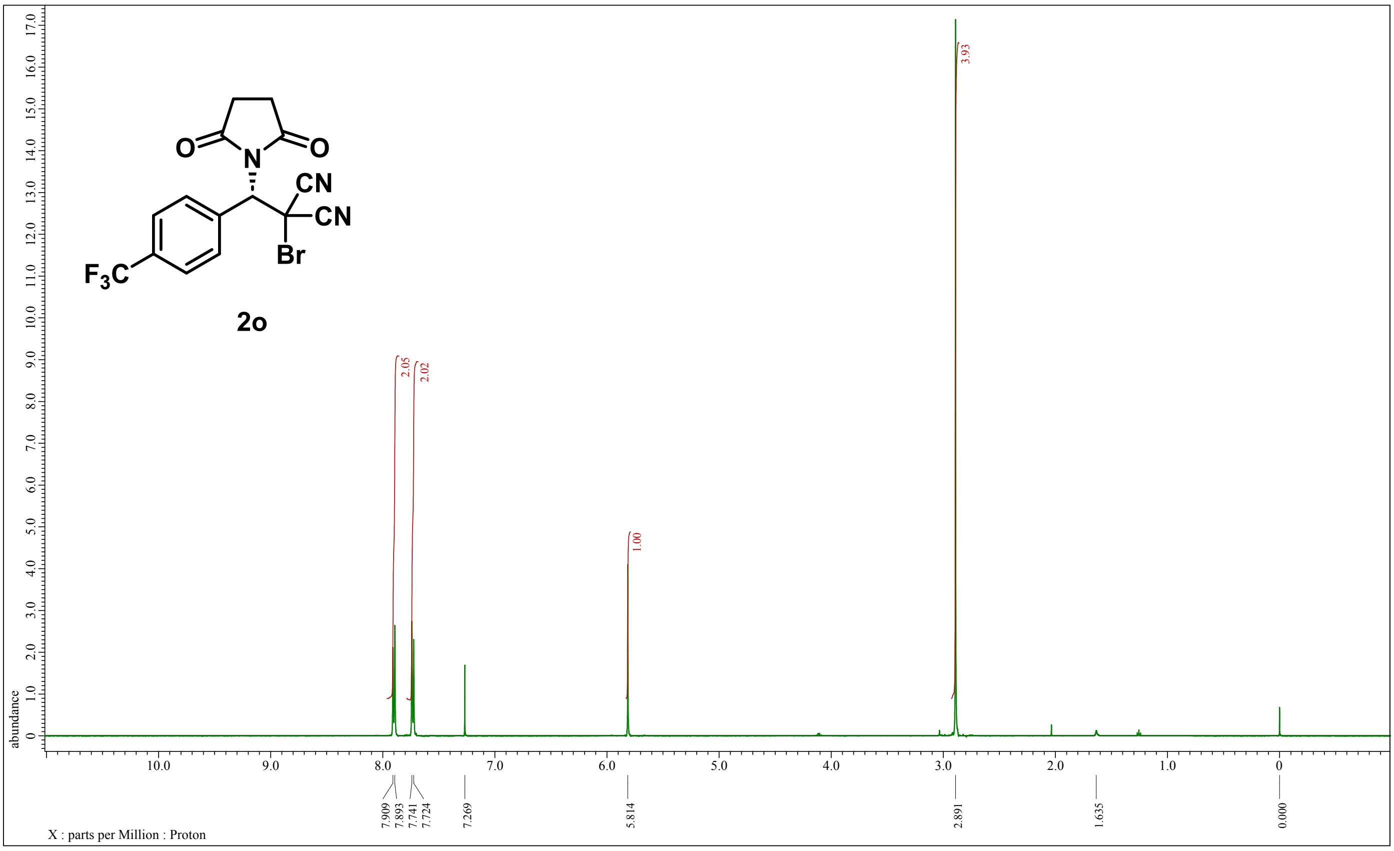


$^{13}$CNMR (125 MHz, chloroform-*d*) spectrum of 2o

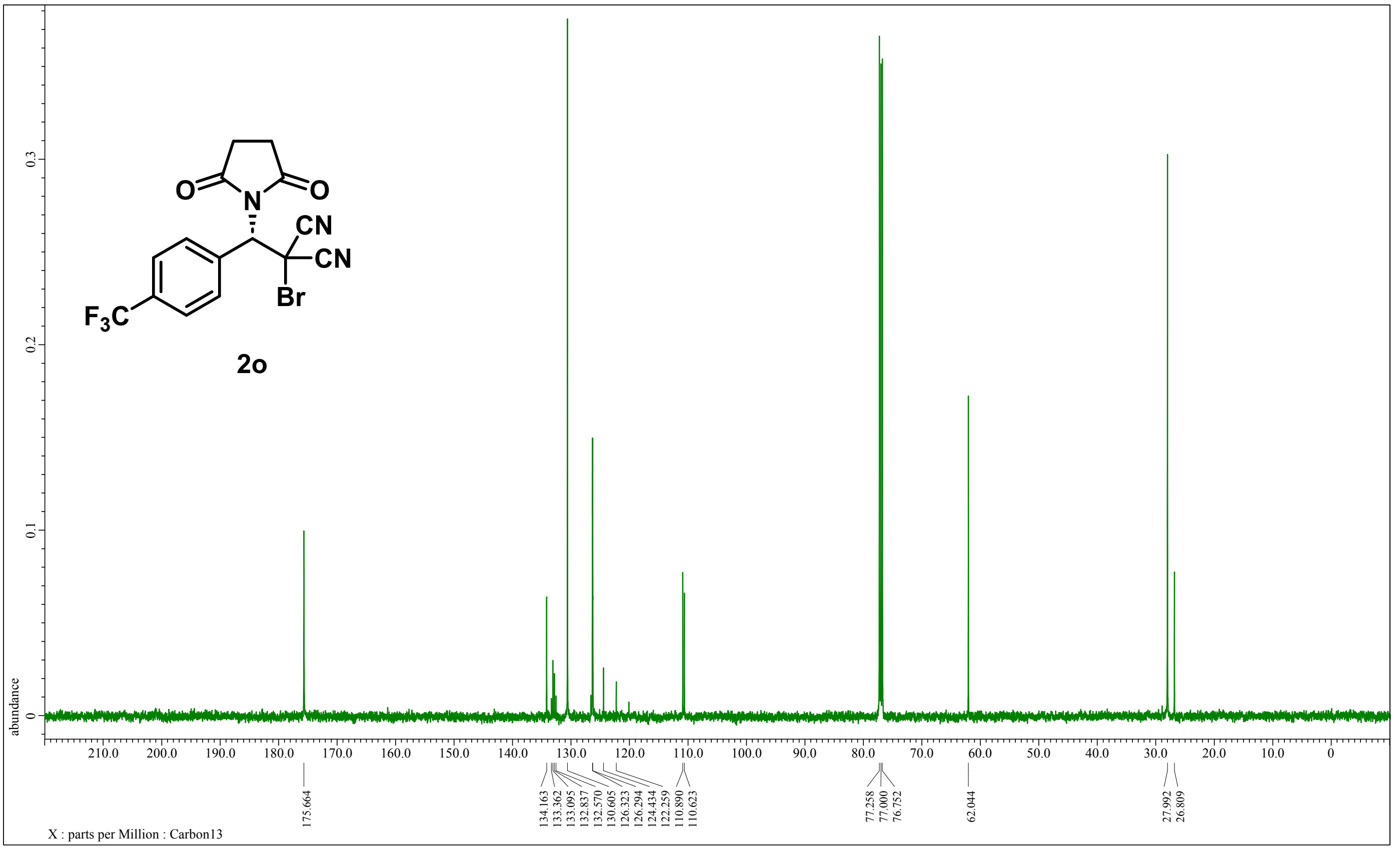

$^{1}$HNMR (500 MHz, chloroform-*d*) spectrum of 2p

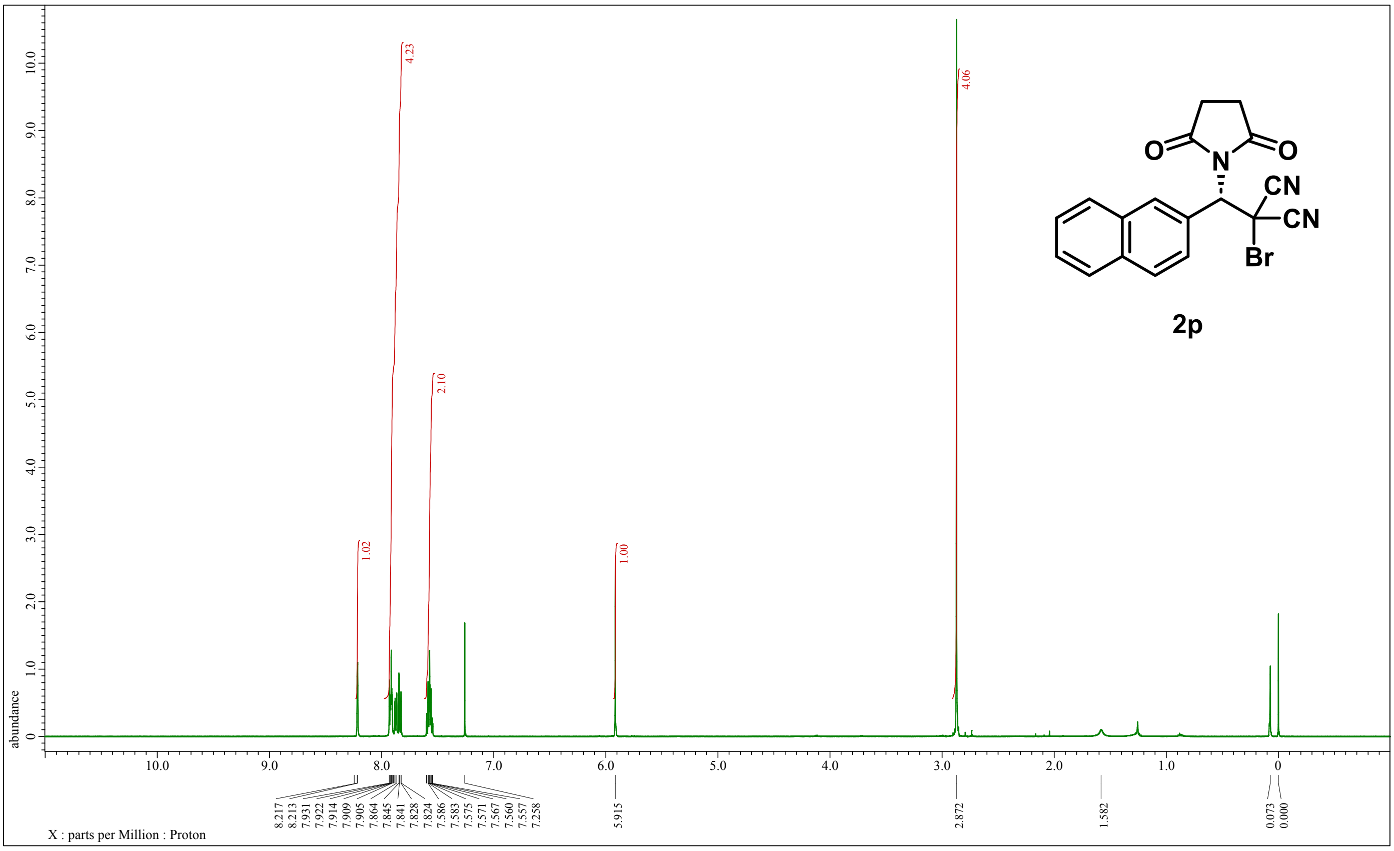


$^{13}$CNMR (125 MHz, chloroform-*d*) spectrum of 2p

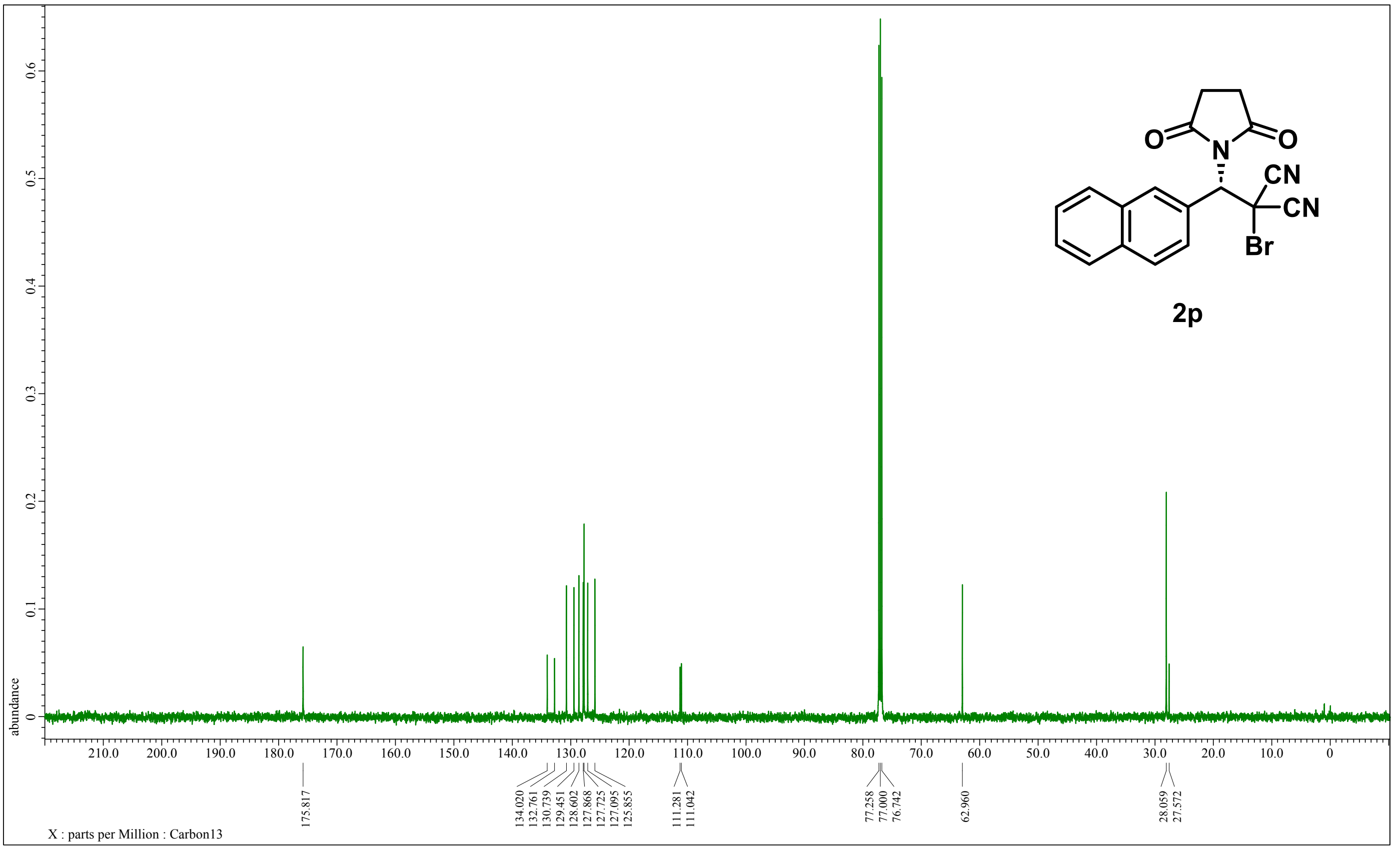

$^{1}$HNMR (500 MHz, chloroform-*d*) spectrum of 2q

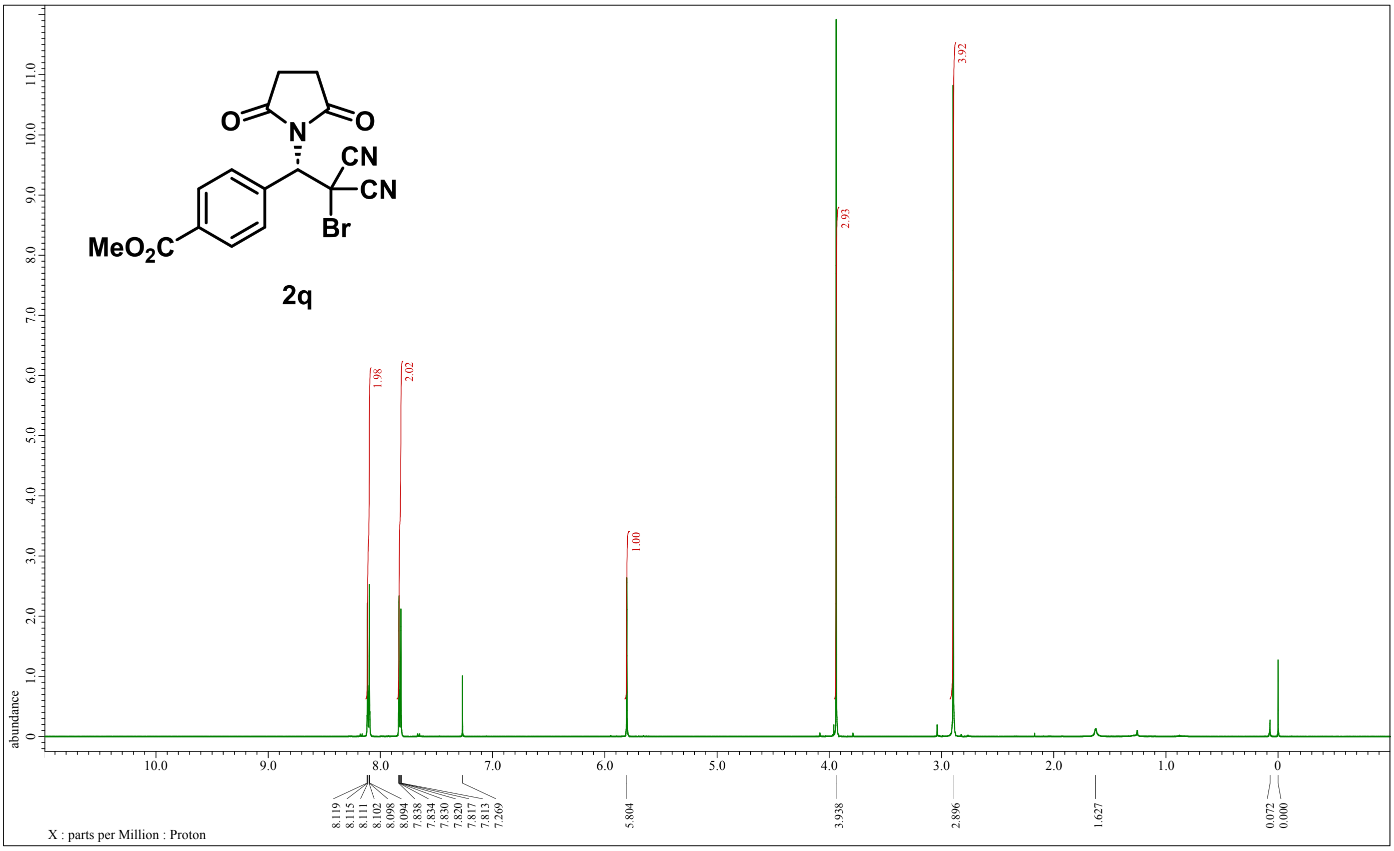


$^{13}$CNMR (125 MHz, chloroform-*d*) spectrum of 2q

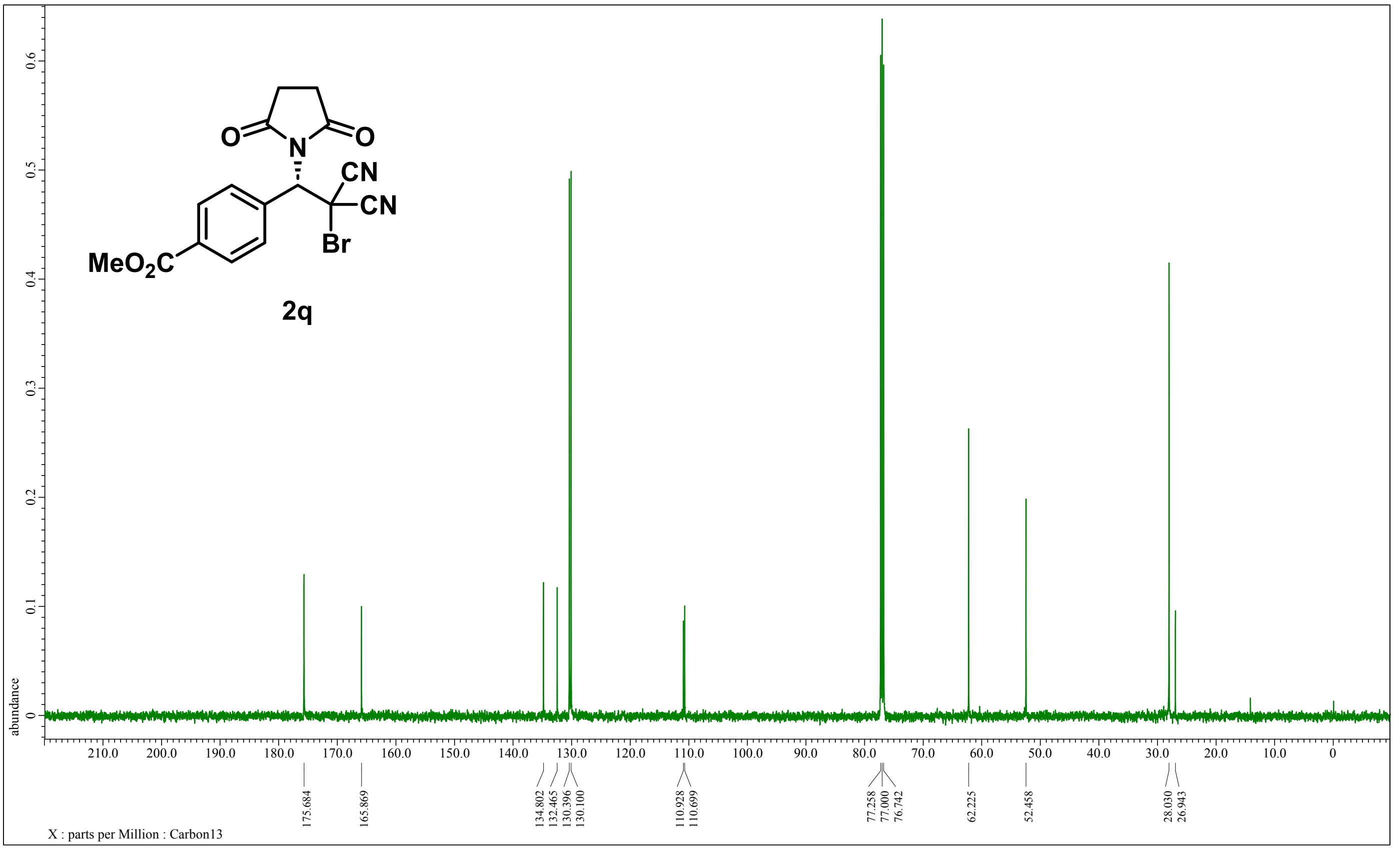

$^{1}$HNMR (500 MHz, chloroform-*d*) spectrum of 2r

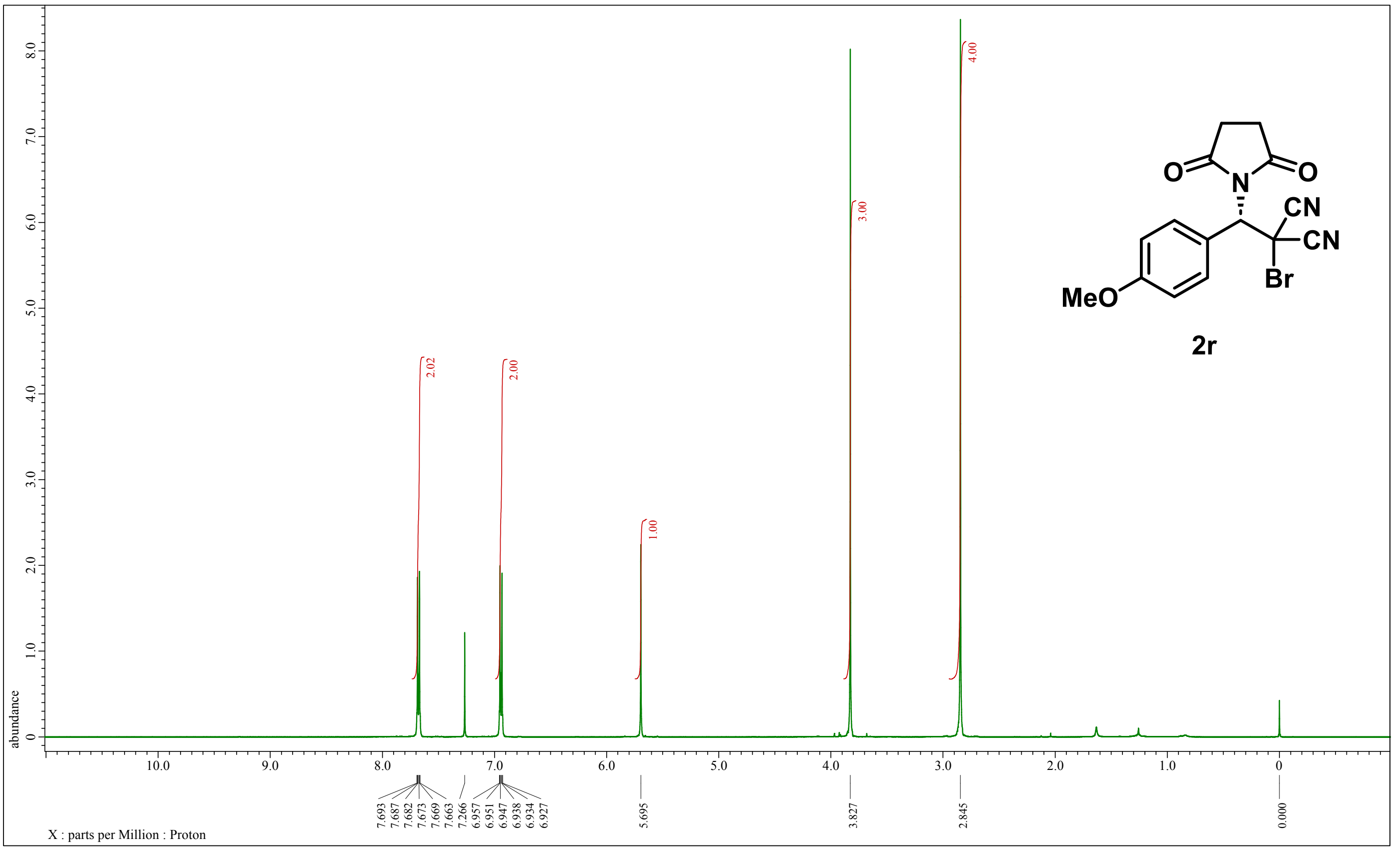


$^{13}$CNMR (125 MHz, chloroform-*d*) spectrum of 2r

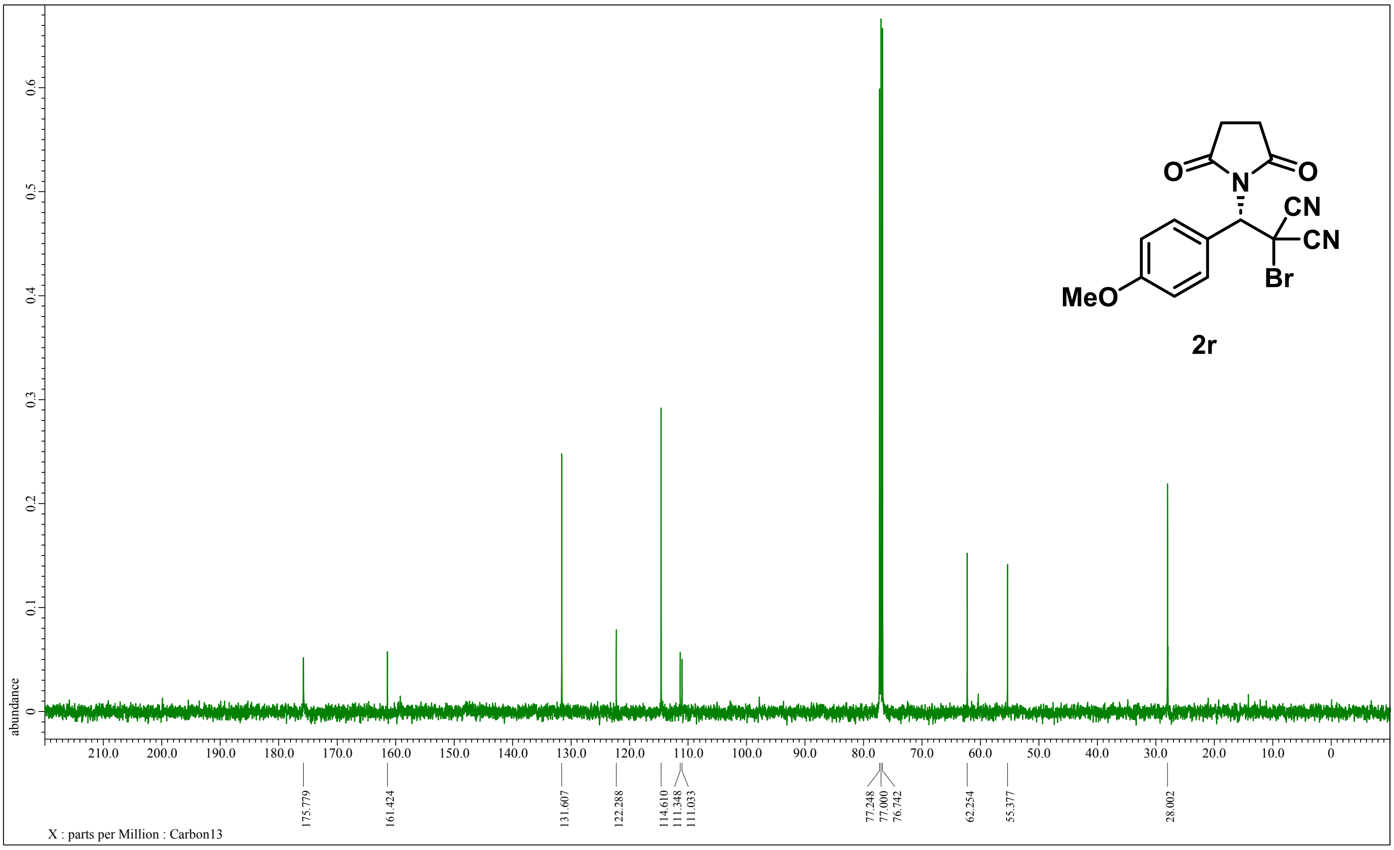

$^{1}$HNMR (500 MHz, chloroform-*d*) spectrum of 2s

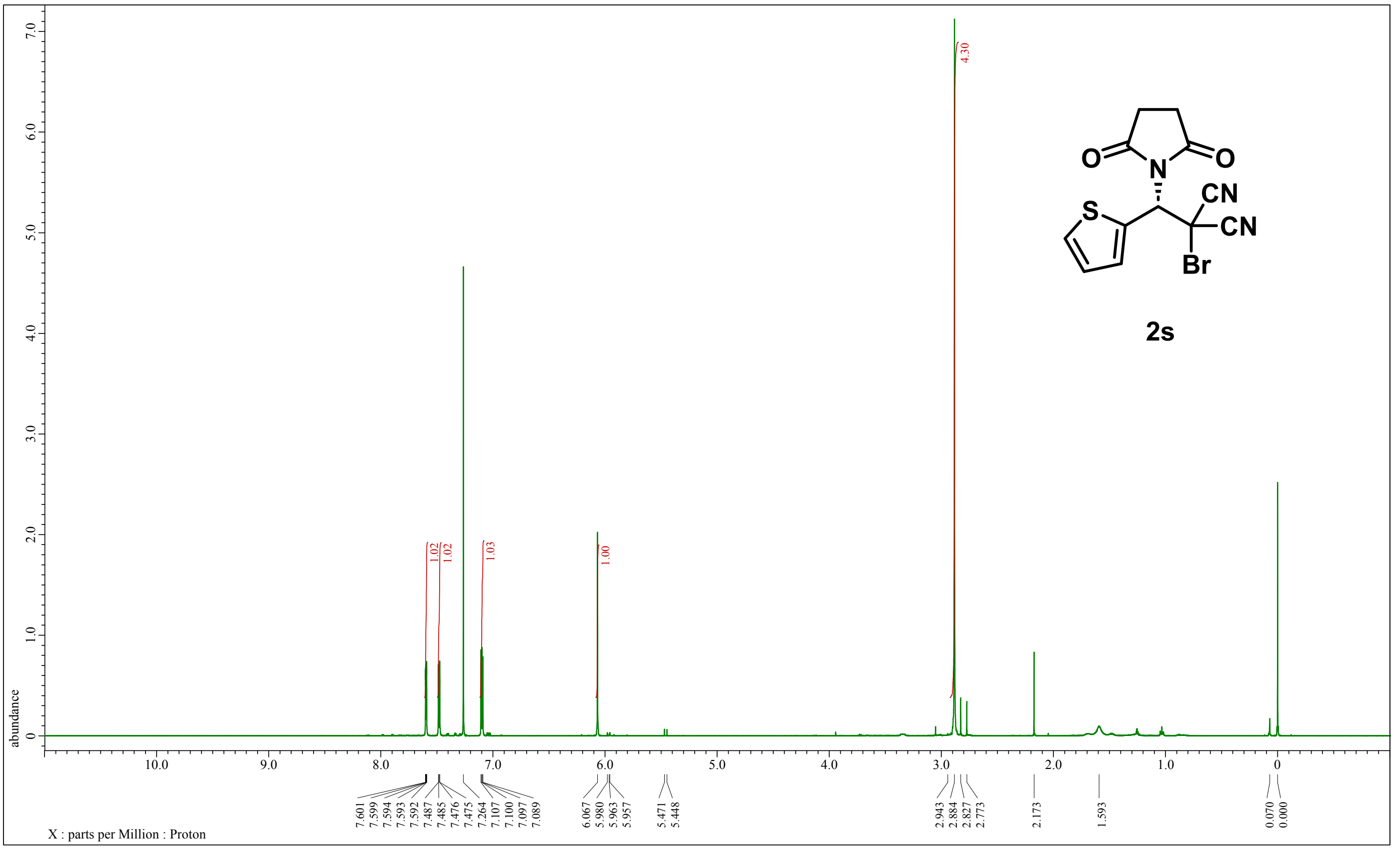


$^{13}$CNMR (125 MHz, chloroform-*d*) spectrum of 2s

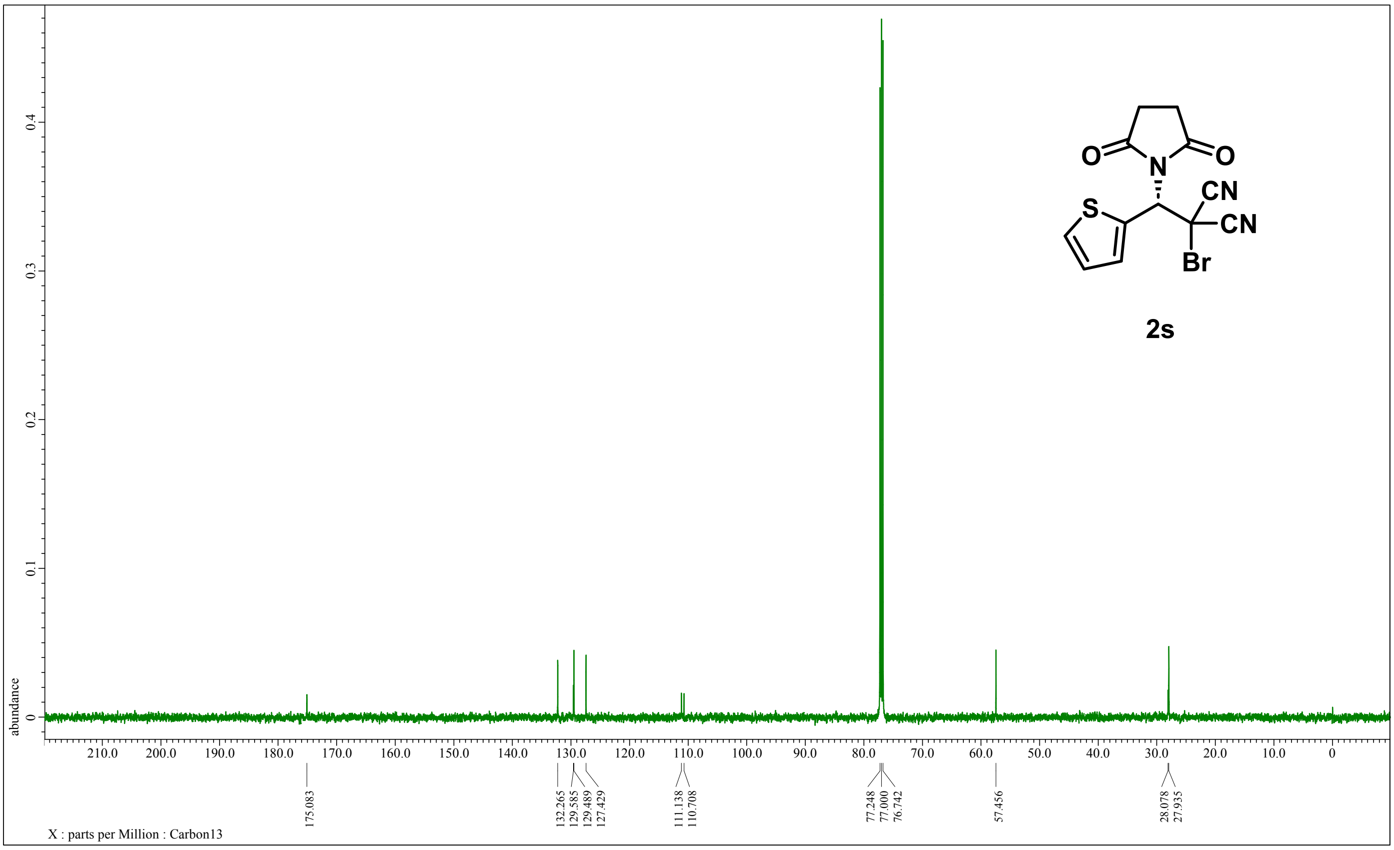

$^{1}$HNMR (400 MHz, chloroform-*d*) spectrum of 2t

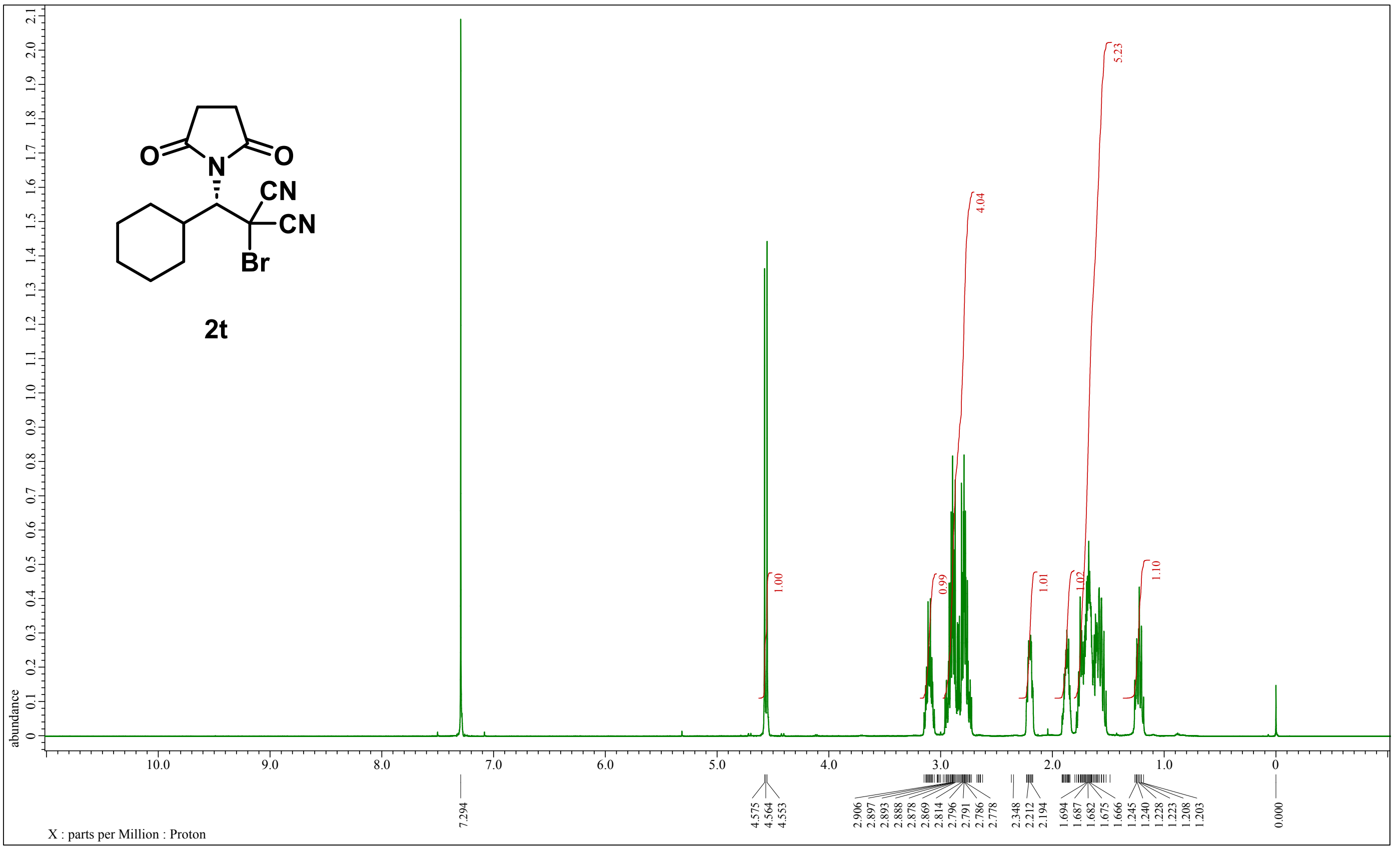


$^{13}$CNMR (125 MHz, chloroform-*d*) spectrum of 2t

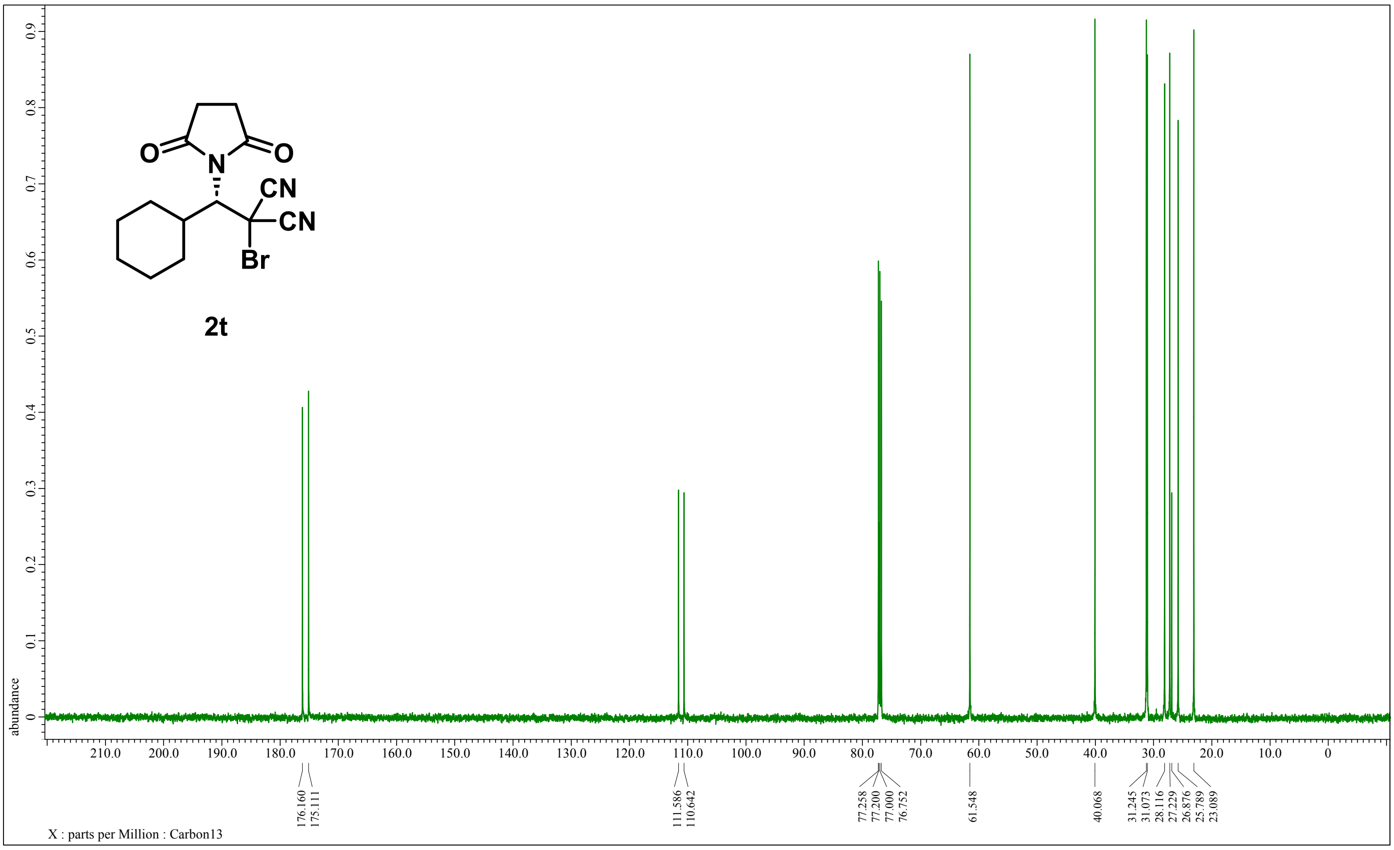

$^{1}$HNMR (500 MHz, chloroform-*d*) spectrum of 2u

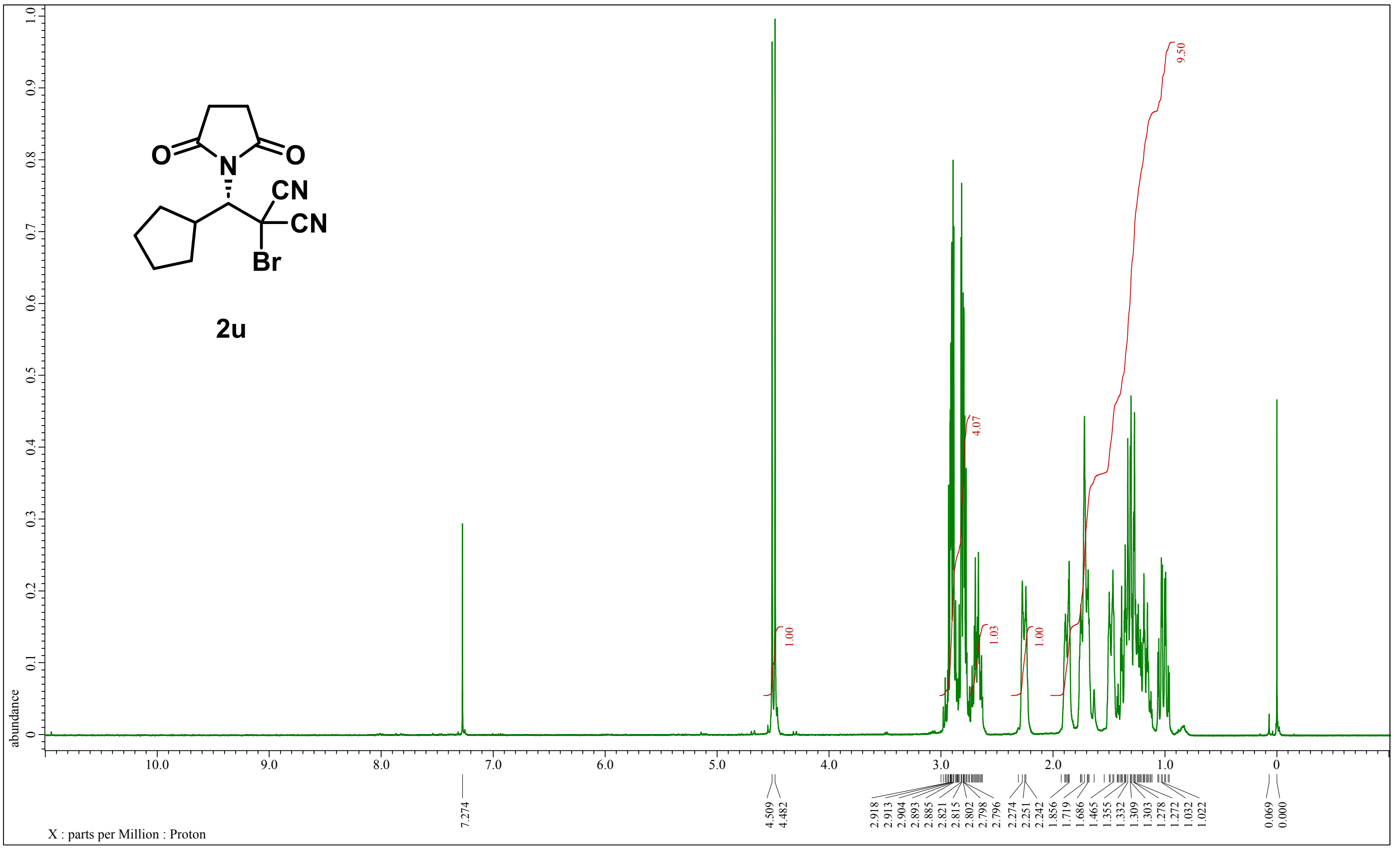


$^{13}$CNMR (125 MHz, chloroform-*d*) spectrum of 2u

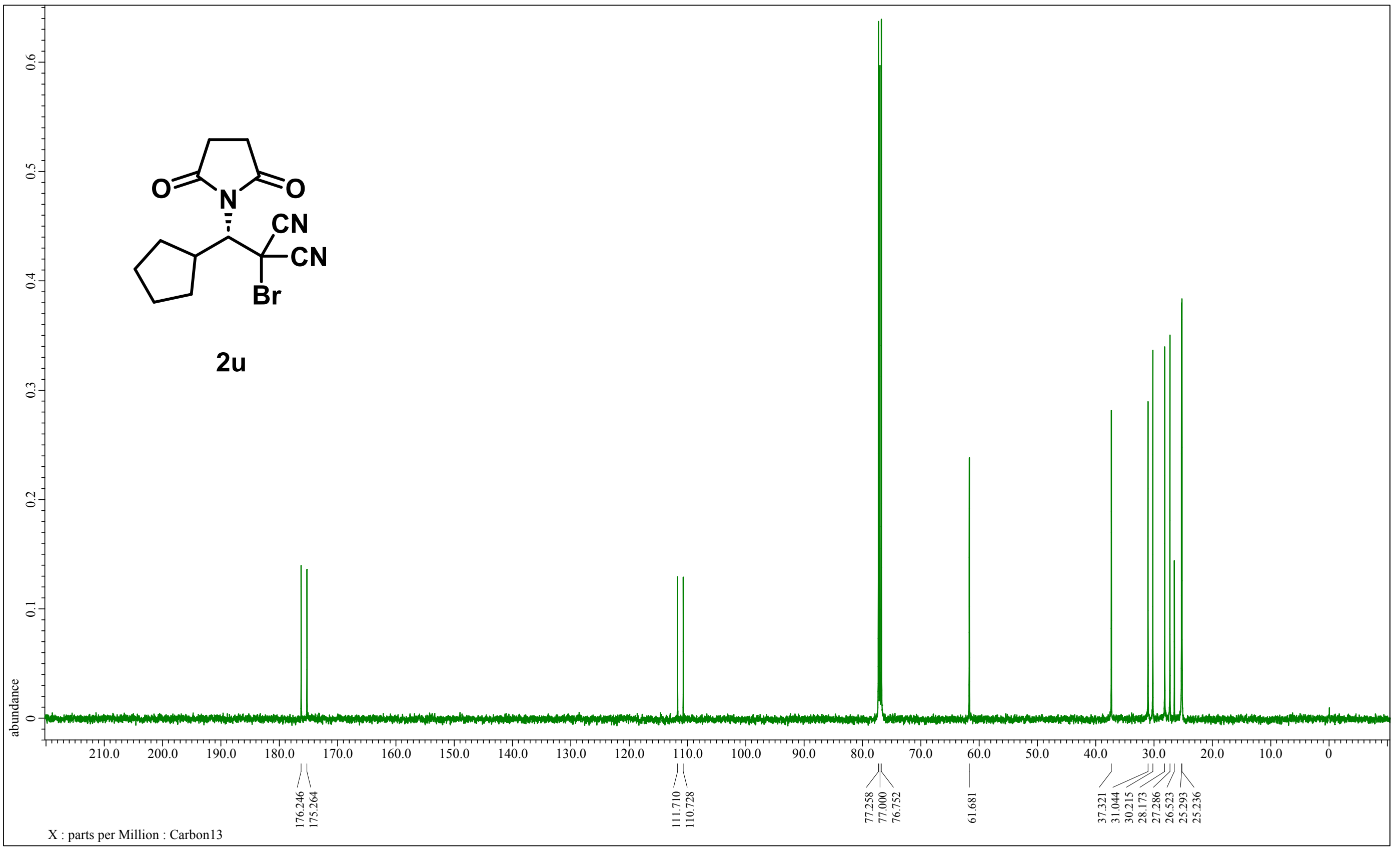

$^{1}$HNMR (400 MHz, chloroform-*d*) spectrum of 3a

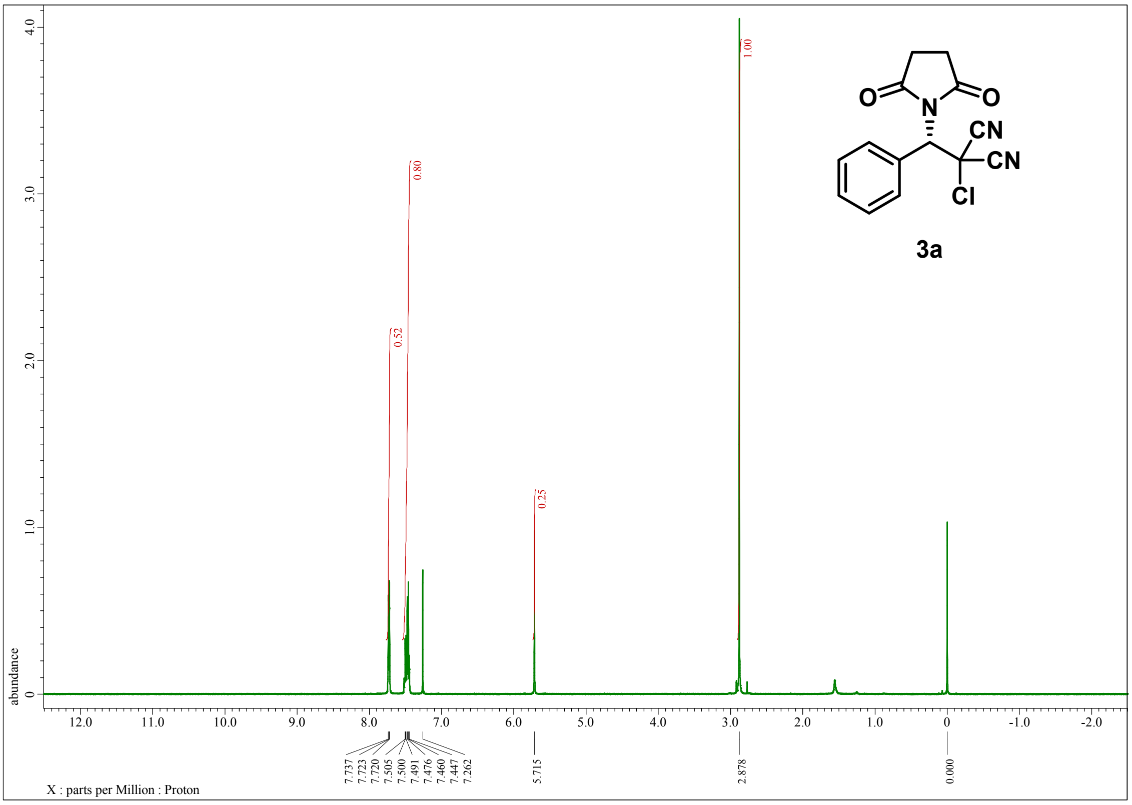


$^{13}$CNMR (125 MHz, chloroform-*d*) spectrum of 3a

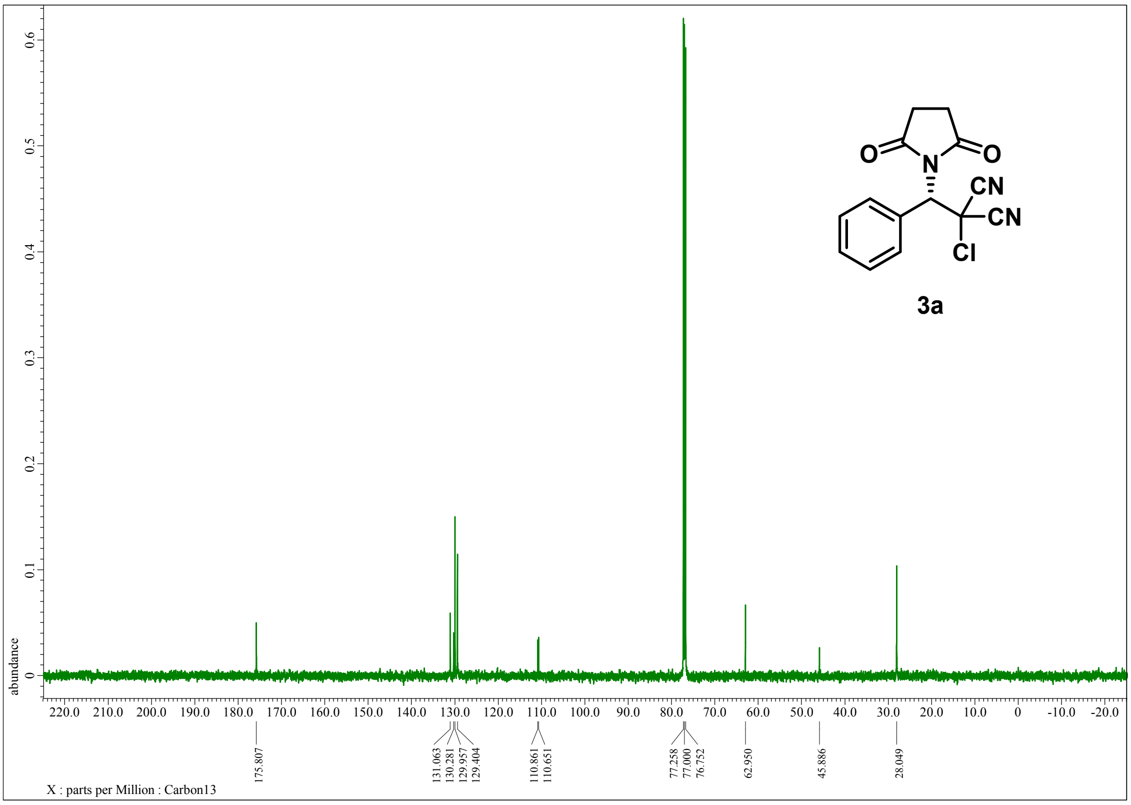

$^{1}$HNMR (400 MHz, chloroform-*d*) spectrum of 3b

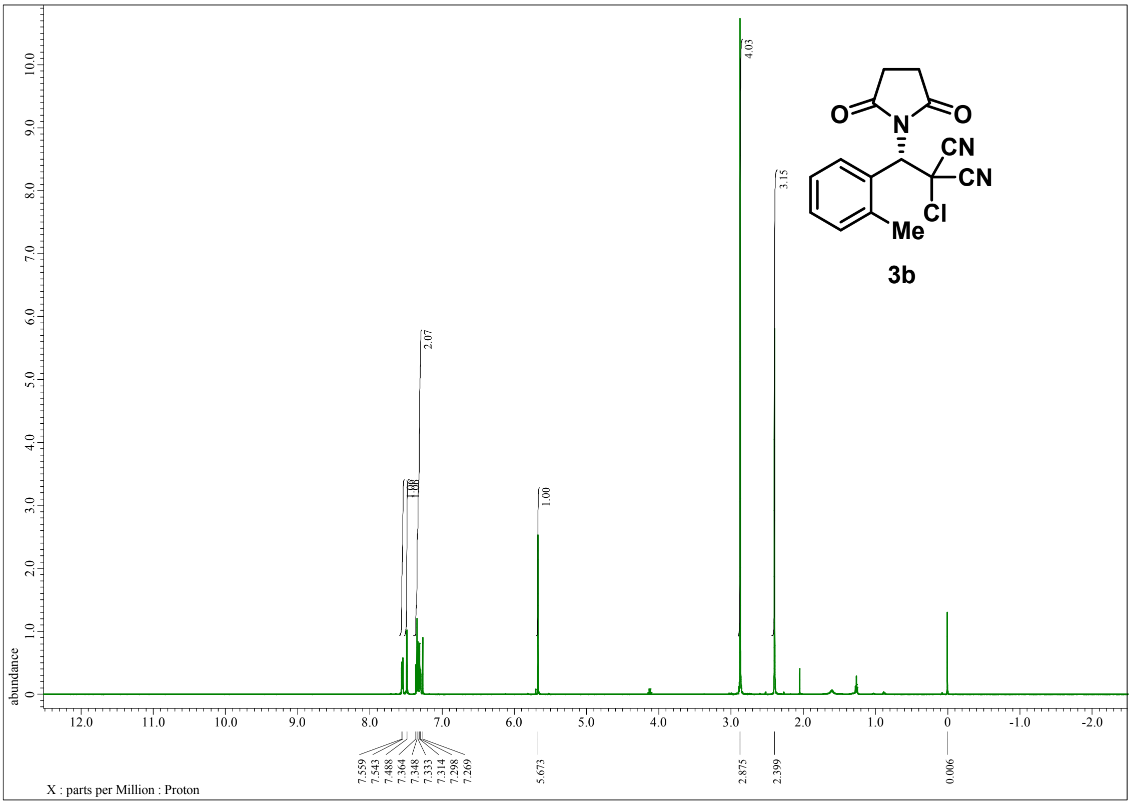


$^{13}$CNMR (125 MHz, chloroform-*d*) spectrum of 3b

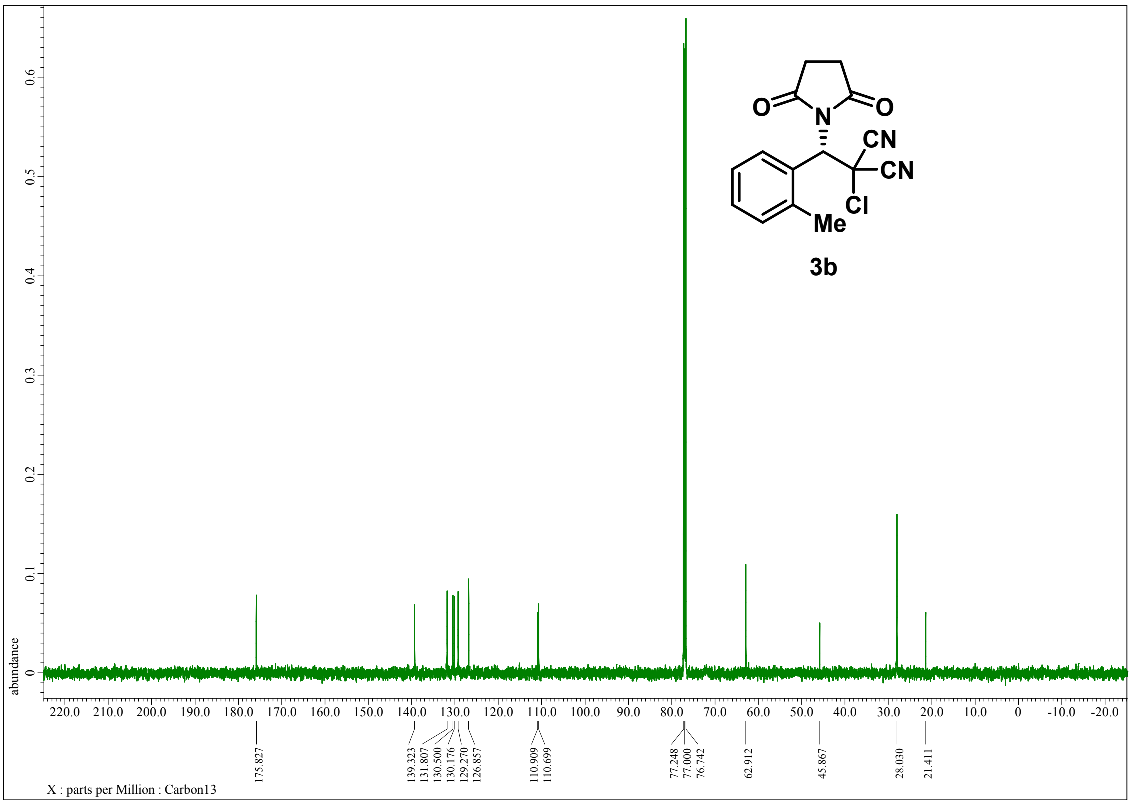

$^{1}$HNMR (400 MHz, chloroform-*d*) spectrum of 3c

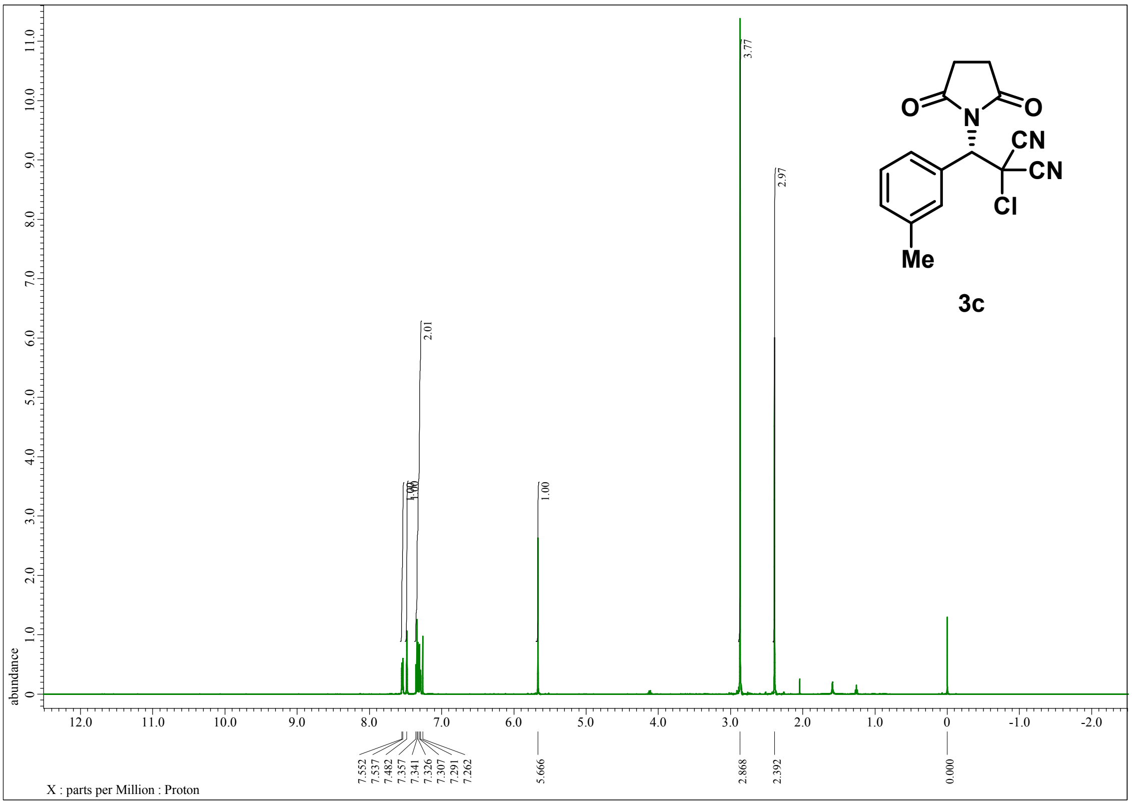


$^{13}$CNMR (125 MHz, chloroform-*d*) spectrum of 3c

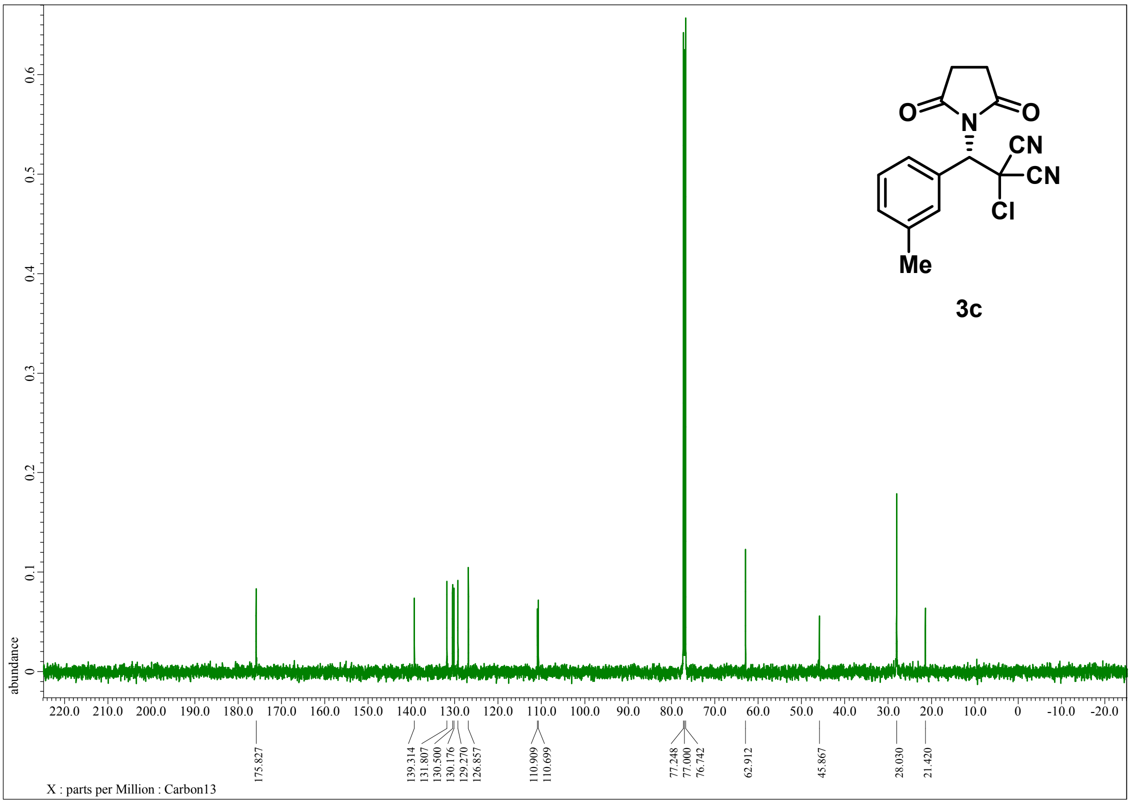

$^{1}$HNMR (400 MHz, chloroform-*d*) spectrum of 3d

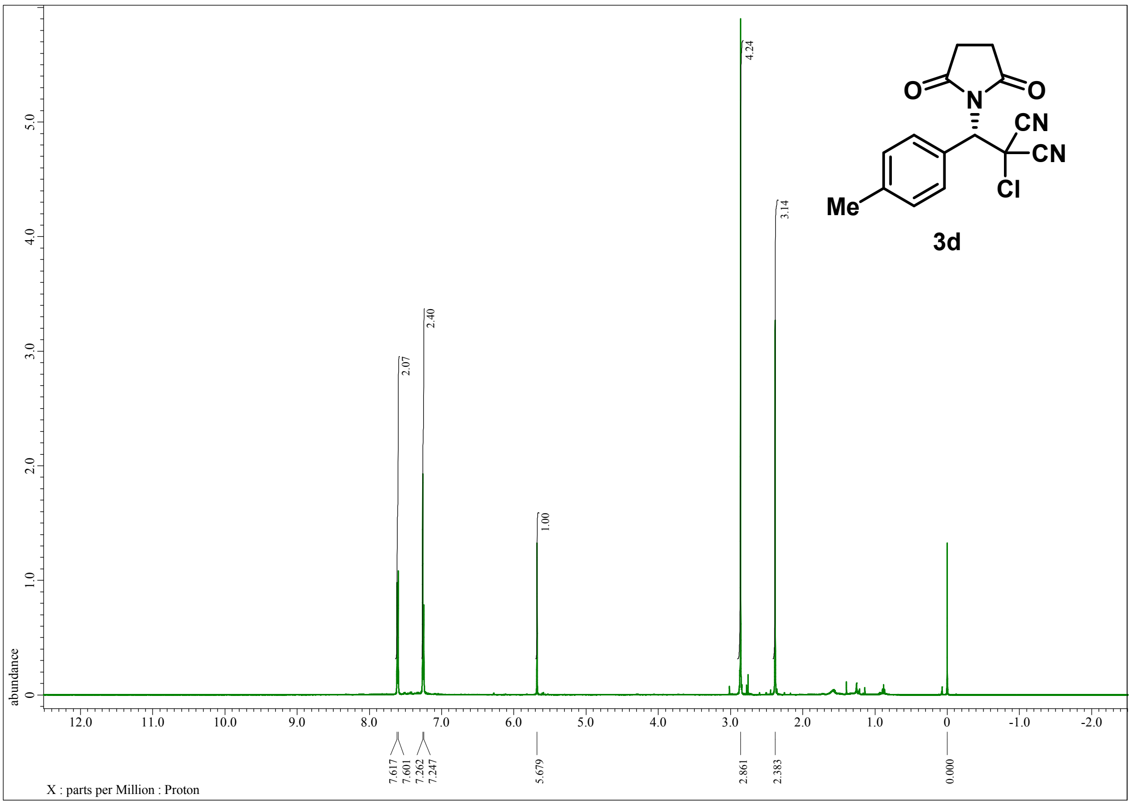


$^{13}$CNMR (125 MHz, chloroform-*d*) spectrum of 3

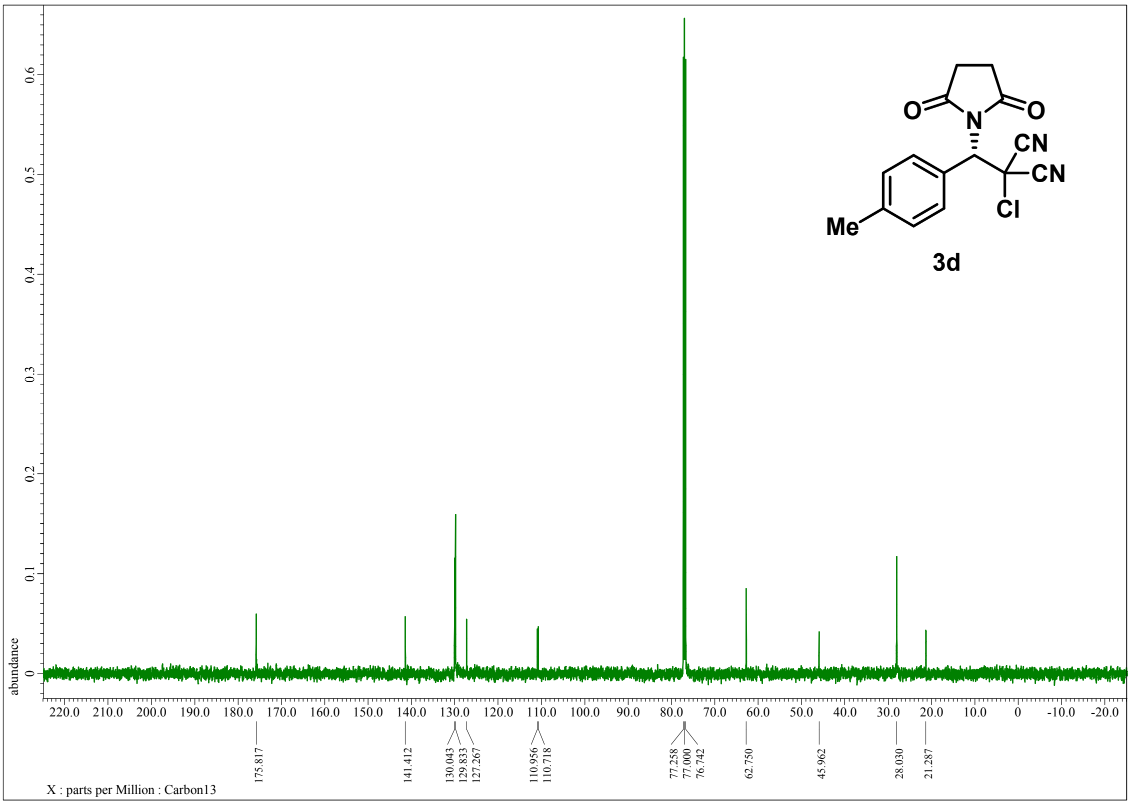

$^{1}$HNMR (400 MHz, chloroform-*d*) spectrum of 3e

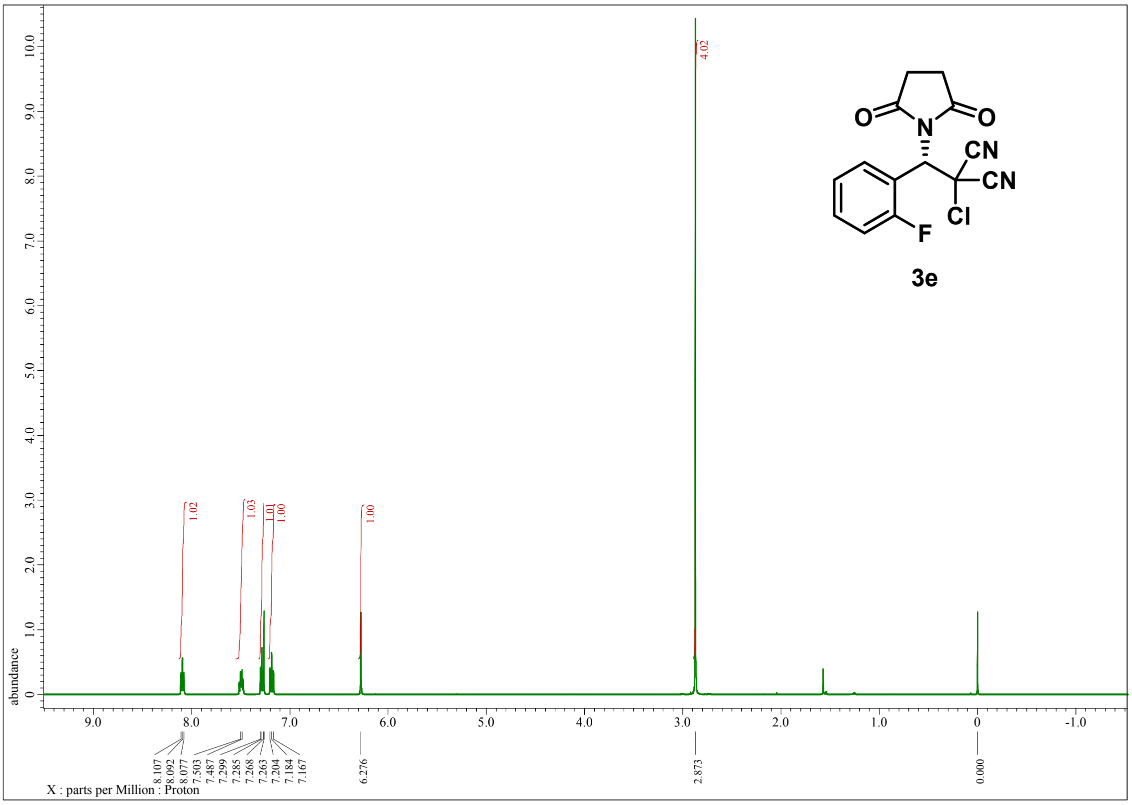


$^{13}$CNMR (125 MHz, chloroform-*d*) spectrum of 3e

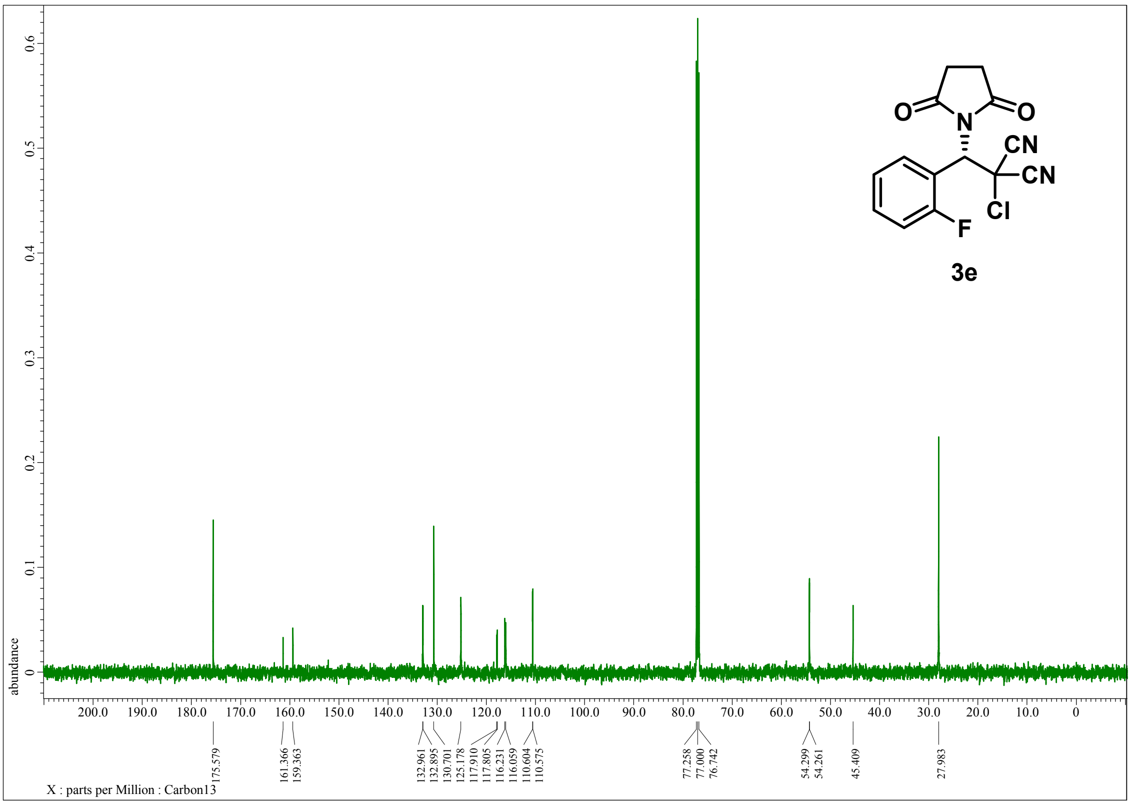

$^{1}$HNMR (400 MHz, chloroform-*d*) spectrum of 3f

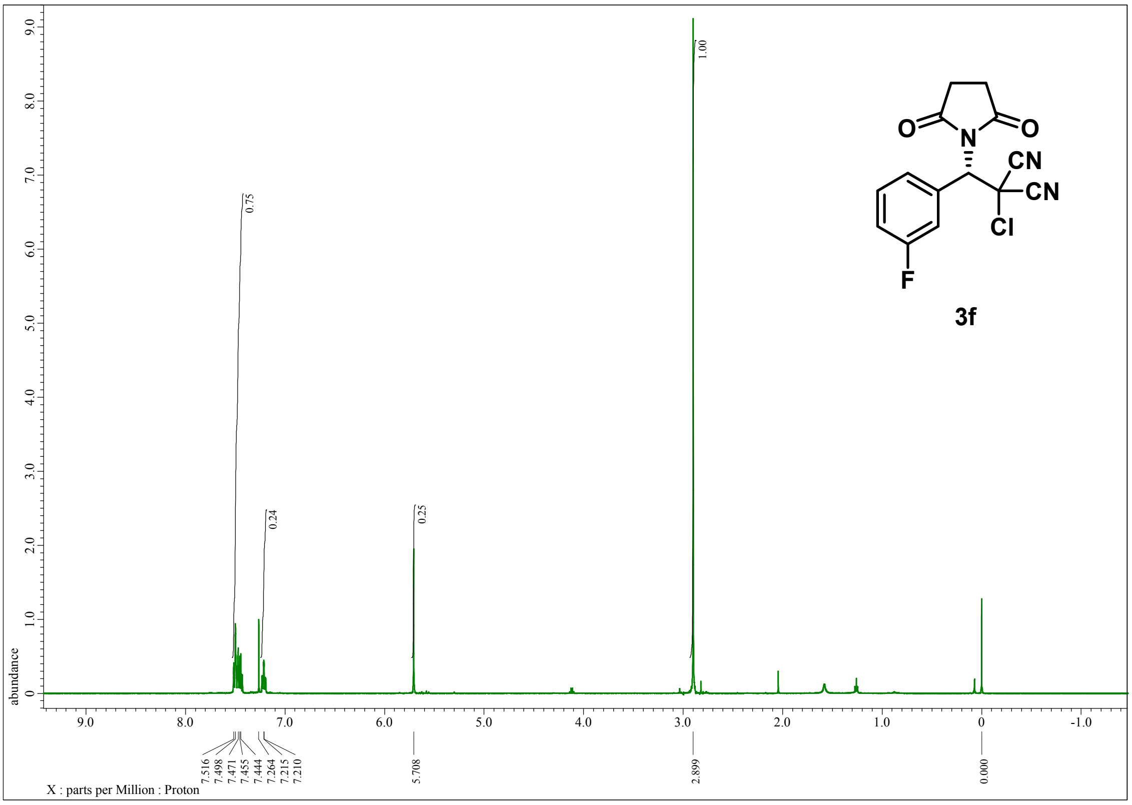


$^{13}$CNMR (125 MHz, chloroform-*d*) spectrum of 3f

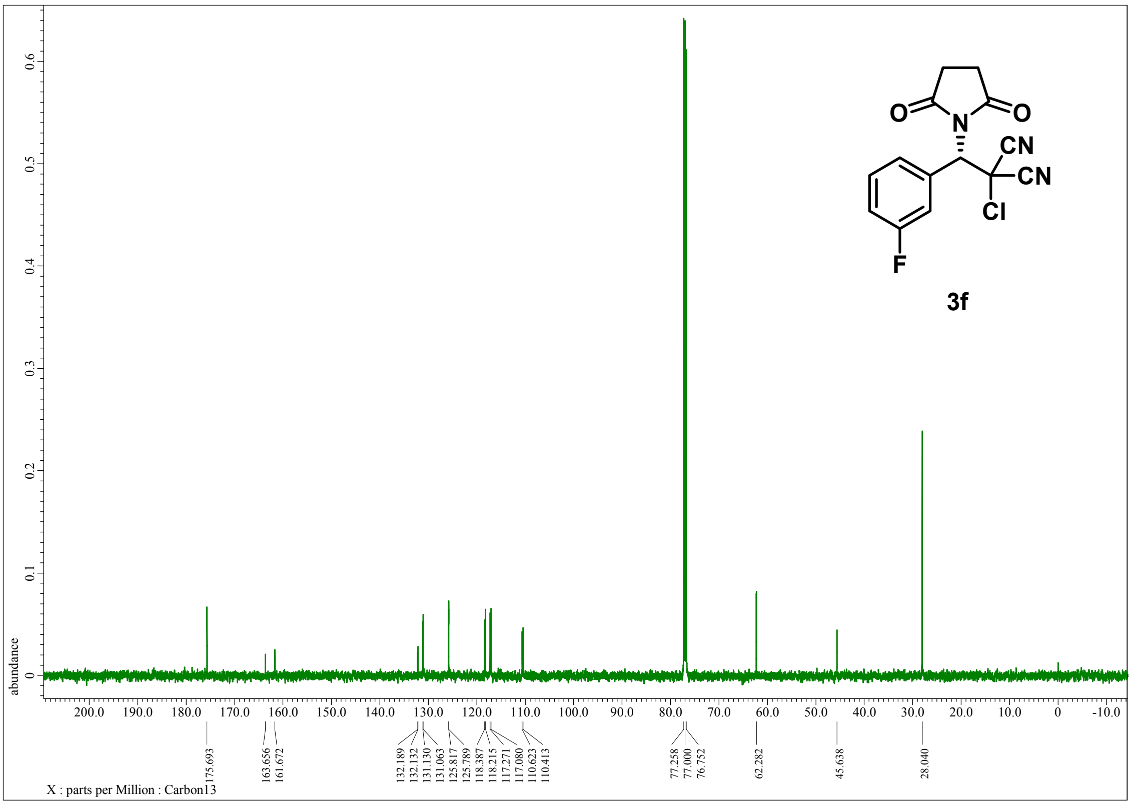

$^{1}$HNMR (400 MHz, chloroform-*d*) spectrum of 3g

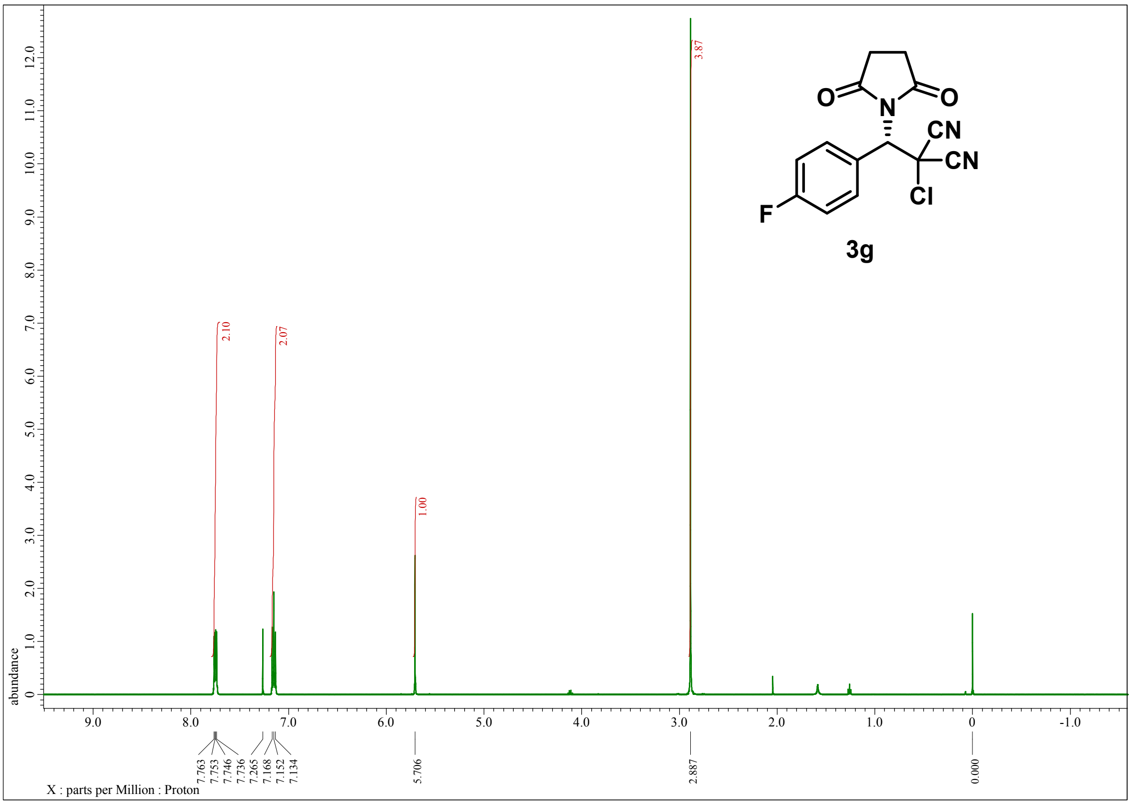


$^{13}$CNMR (125 MHz, chloroform-*d*) spectrum of 3g

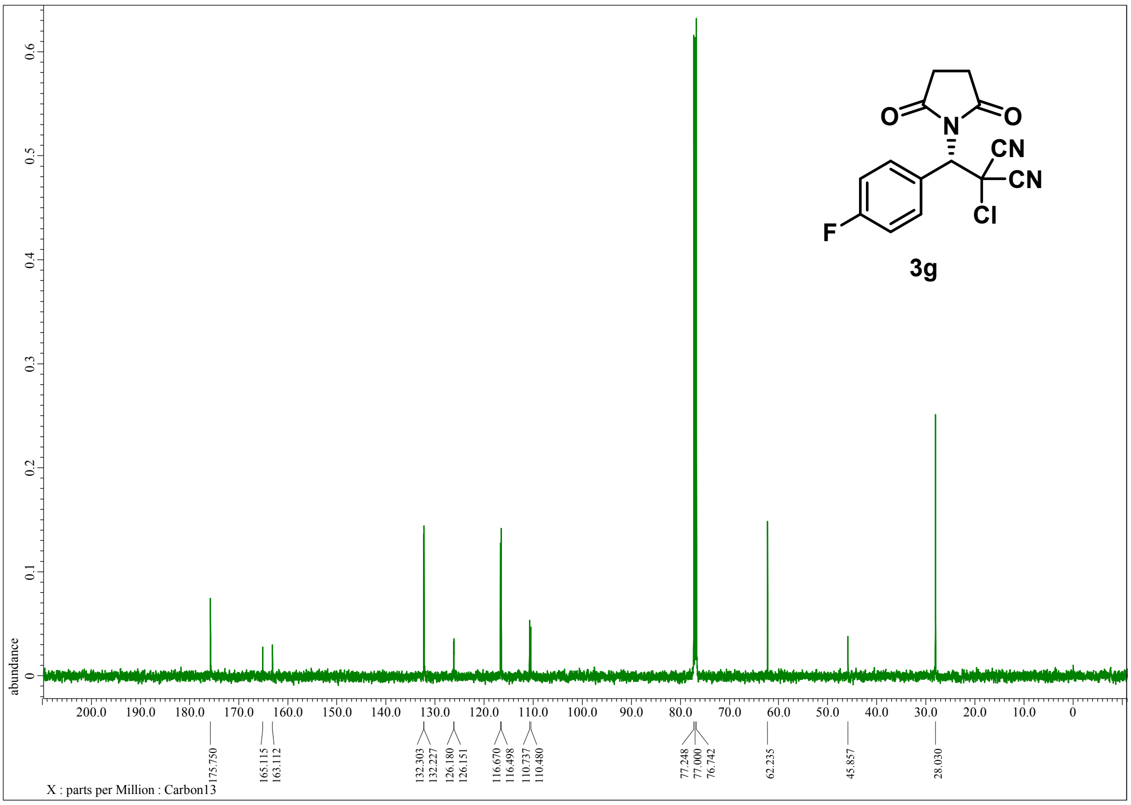

$^{1}$HNMR (400 MHz, chloroform-*d*) spectrum of 3h

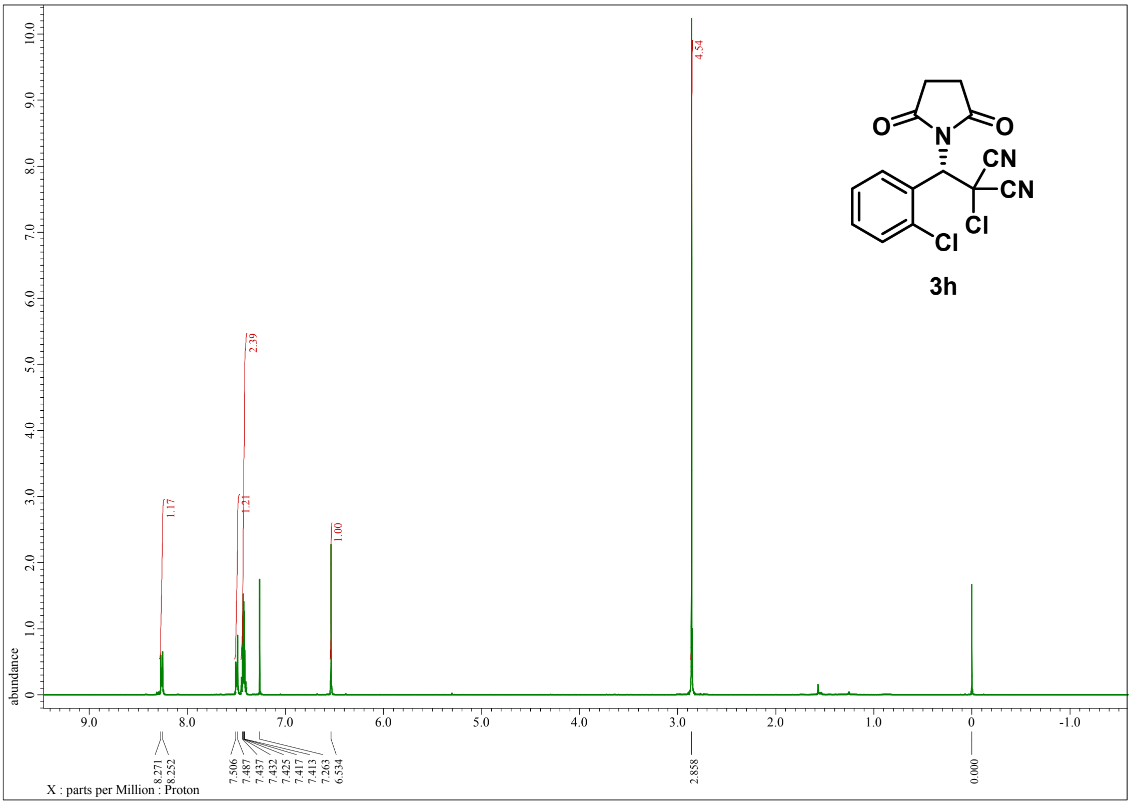


$^{13}$CNMR (125 MHz, chloroform-*d*) spectrum of 3h

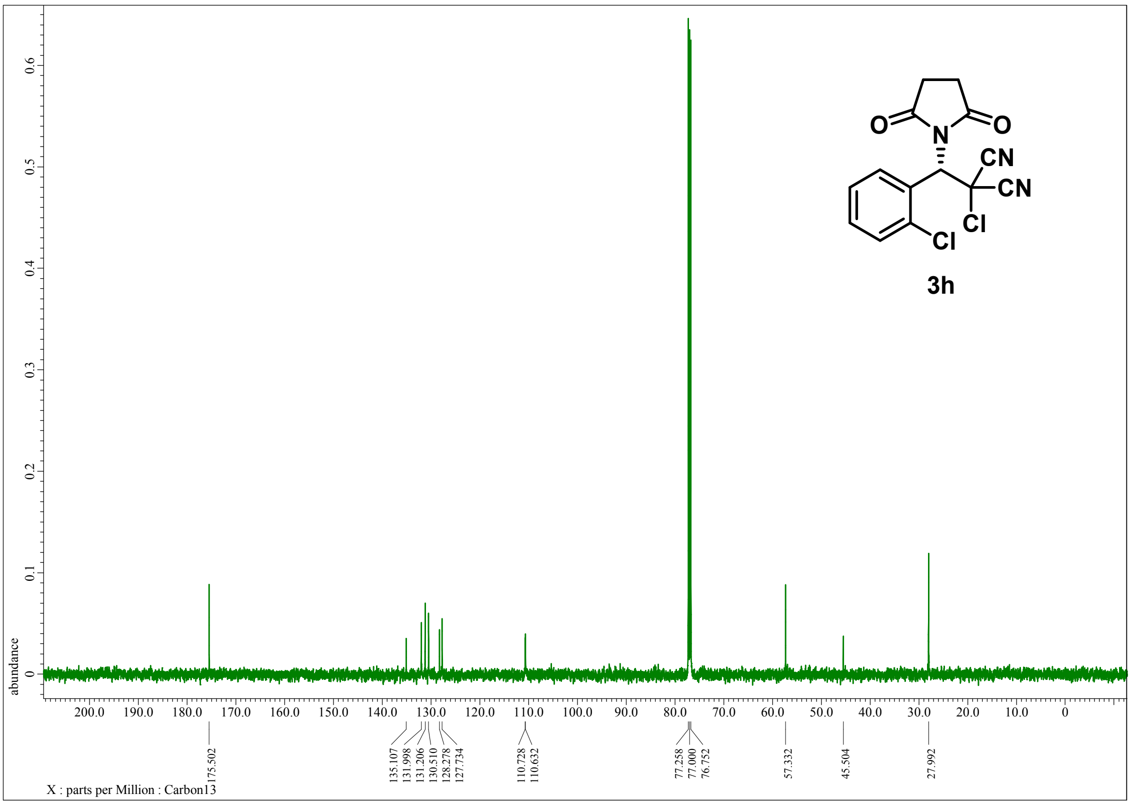

$^{1}$HNMR (400 MHz, chloroform-*d*) spectrum of 3i

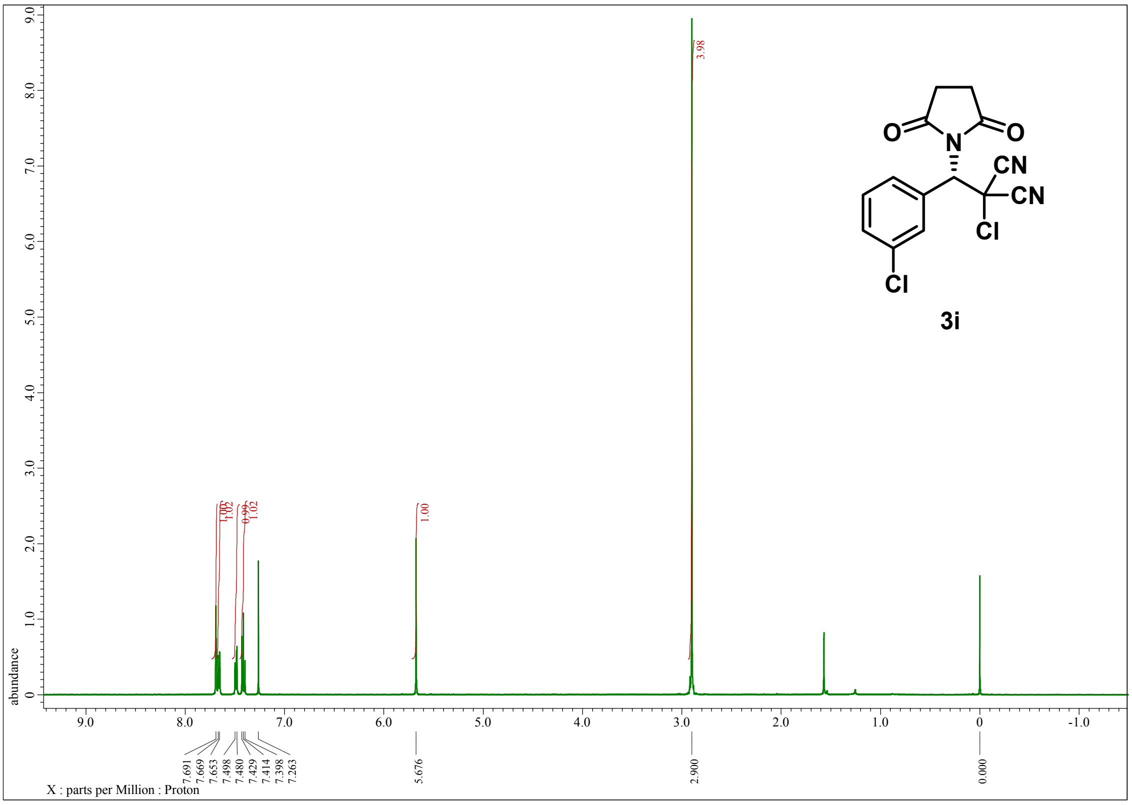


$^{13}$CNMR (125 MHz, chloroform-*d*) spectrum of 3i

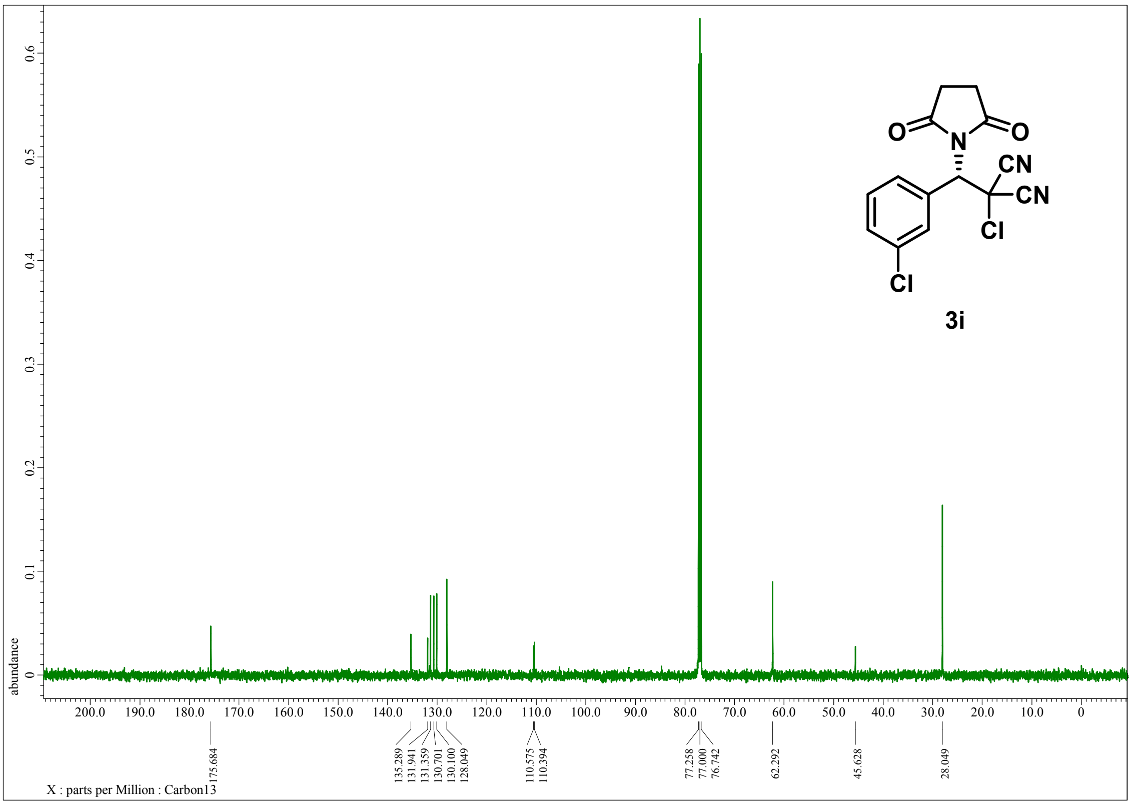

$^{1}$HNMR (400 MHz, chloroform-*d*) spectrum of 3j

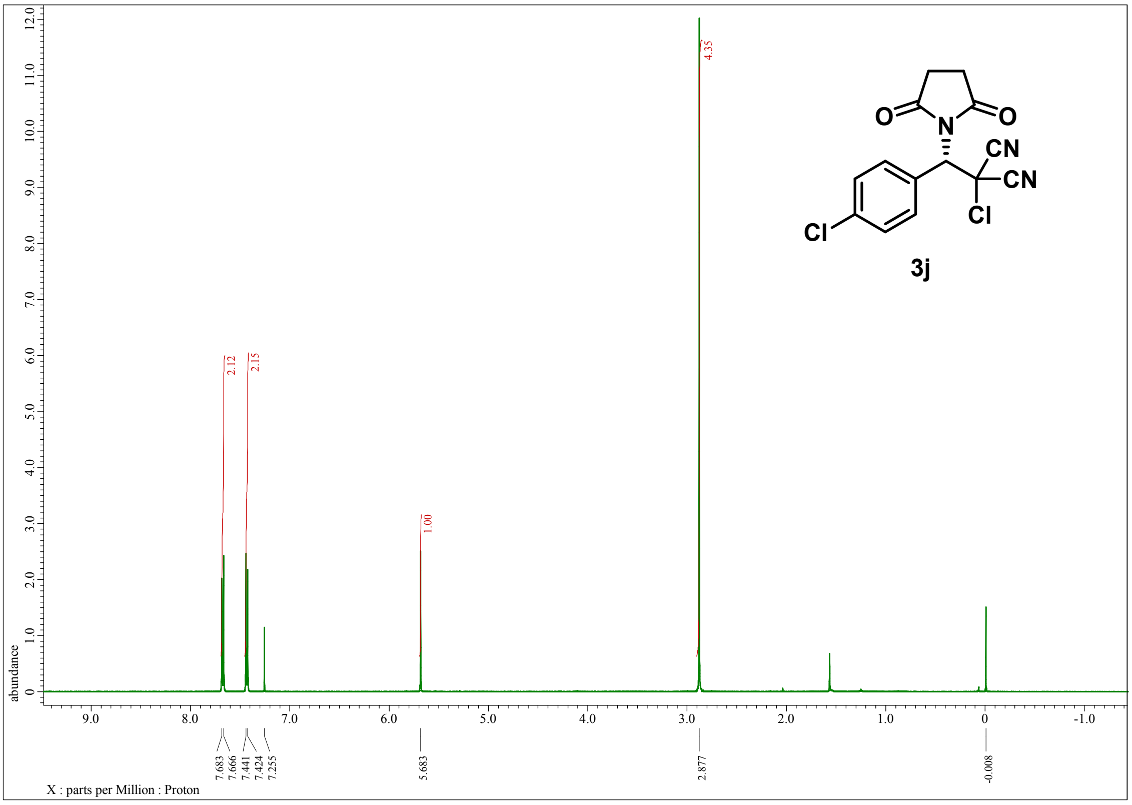


$^{13}$CNMR (125 MHz, chloroform-*d*) spectrum of 3j

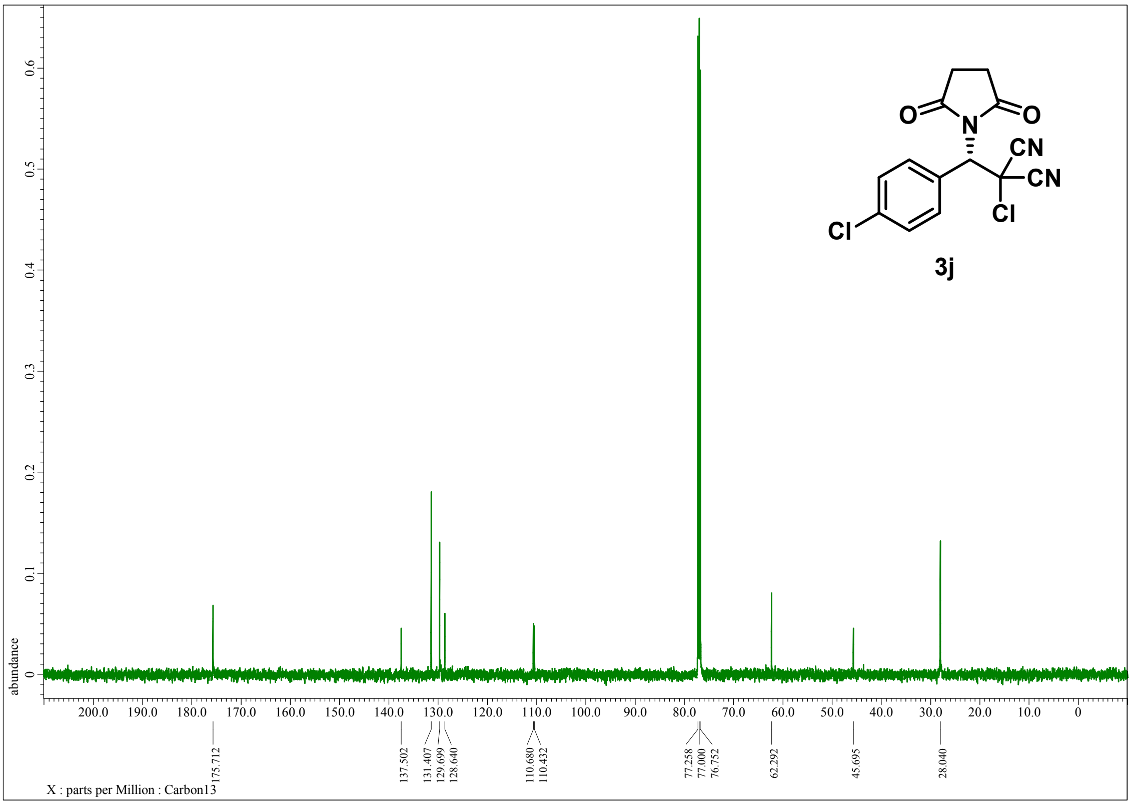

$^{1}$HNMR (400 MHz, chloroform-*d*) spectrum of 3k

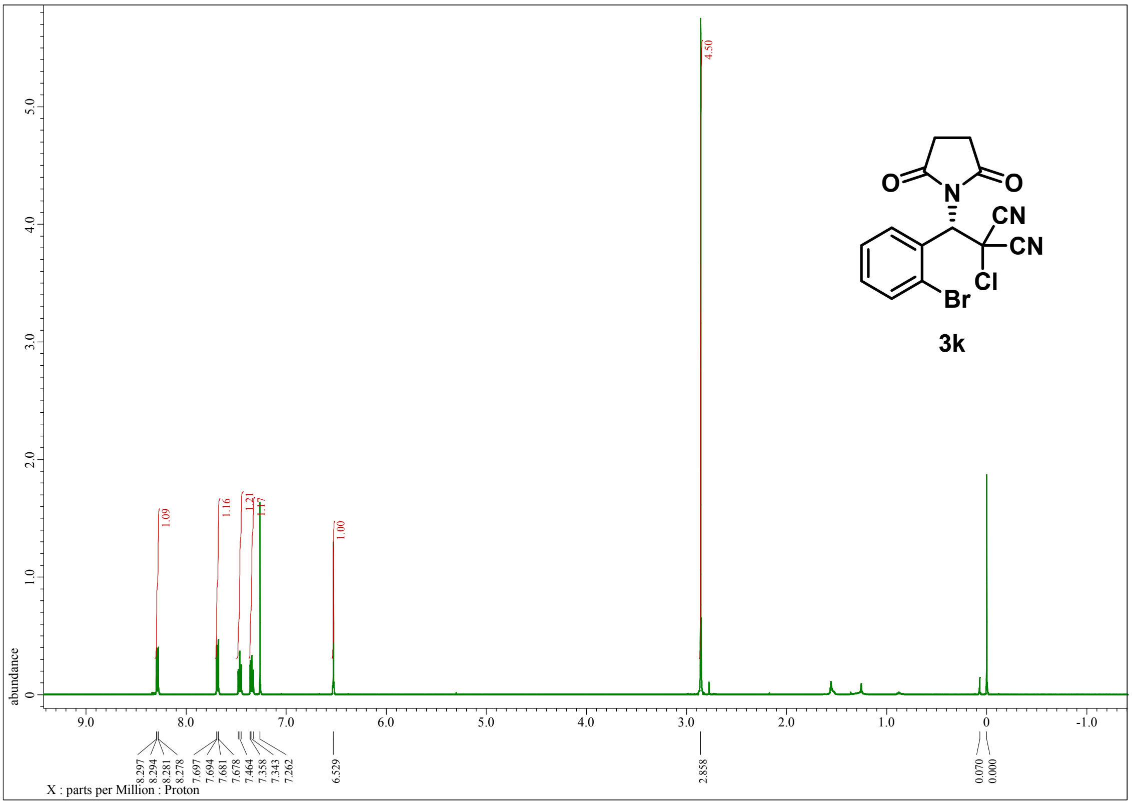

$^{13}$CNMR (125 MHz, chloroform-*d*) spectrum of 3k

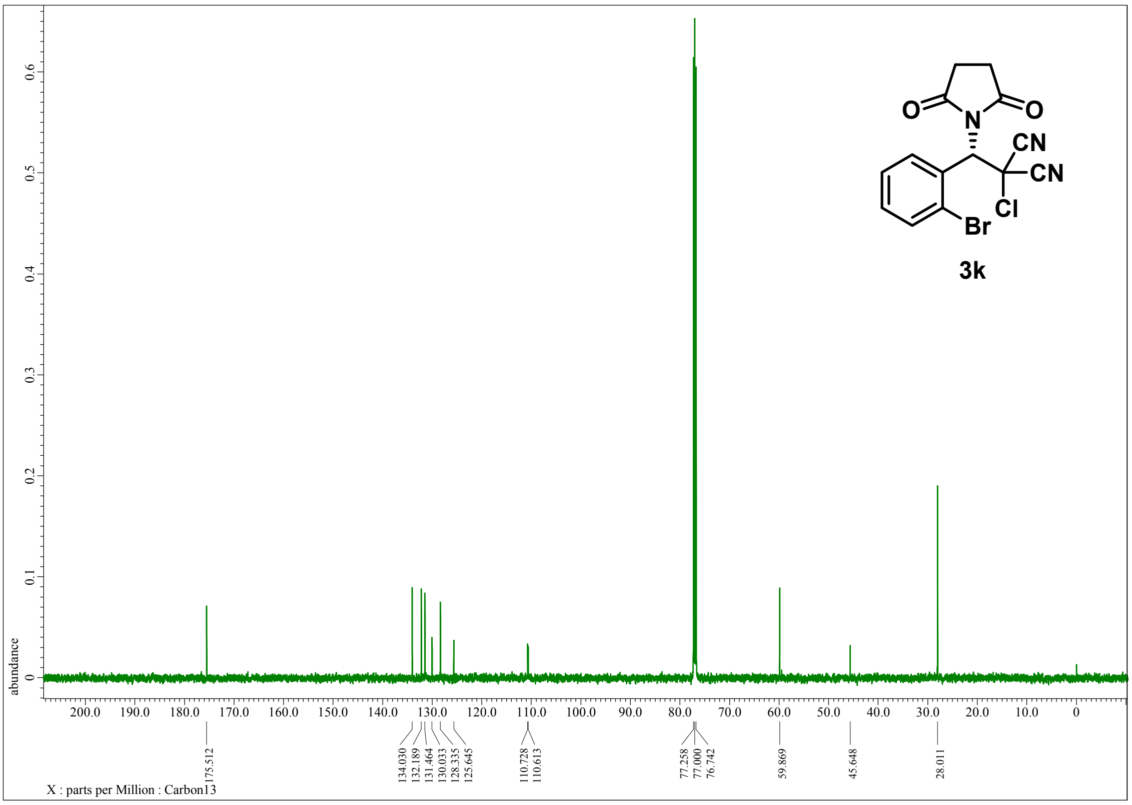

$^{1}$HNMR (400 MHz, chloroform-*d*) spectrum of 3l

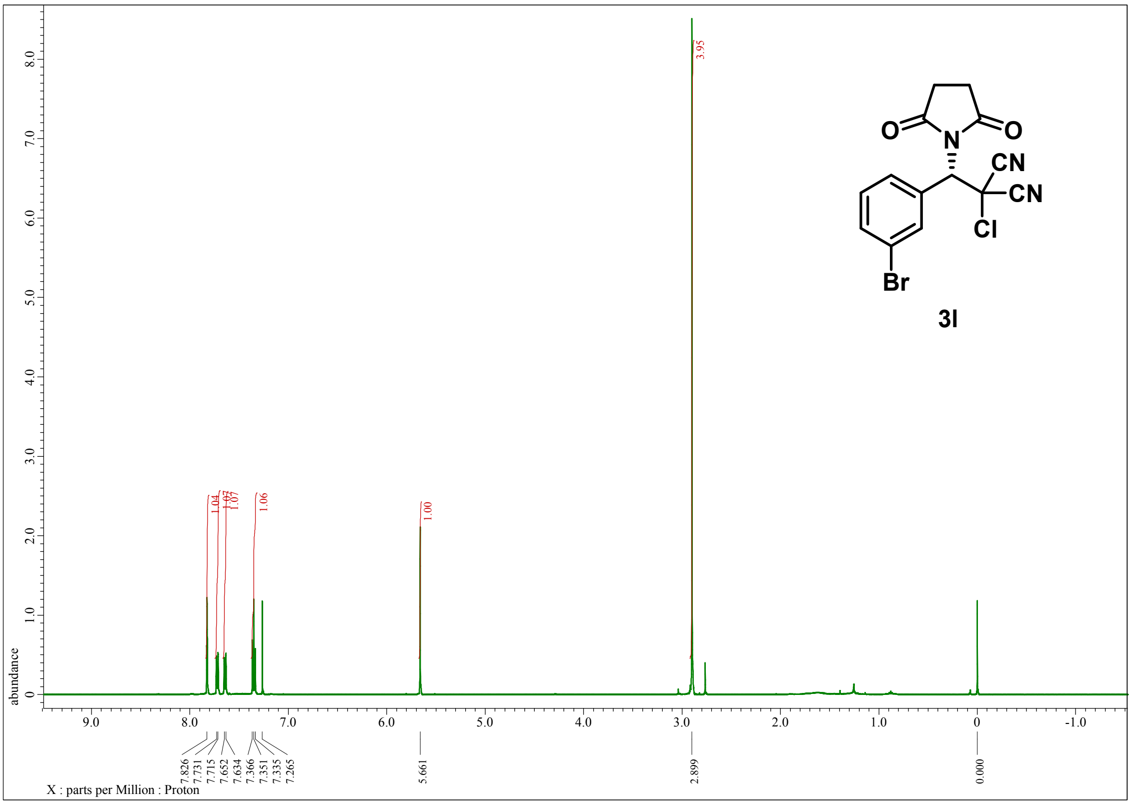

$^{13}$CNMR (125 MHz, chloroform-*d*) spectrum of 3l

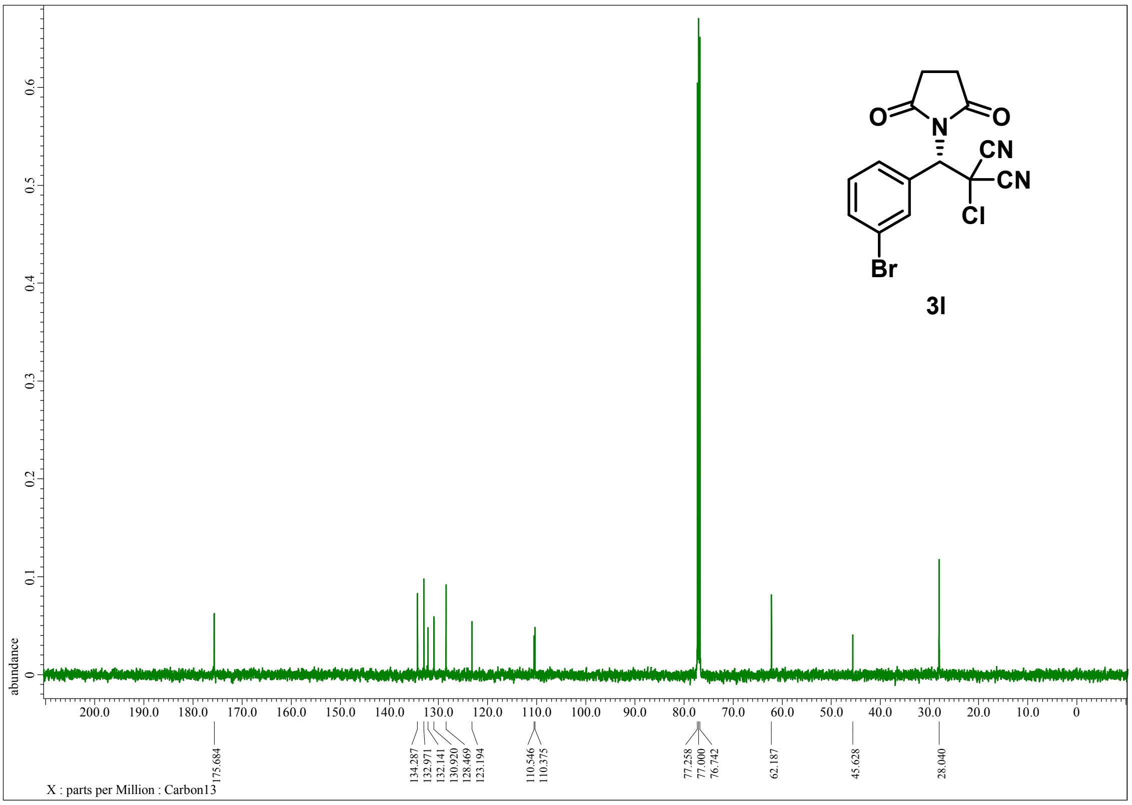

$^{1}$HNMR (400 MHz, chloroform-*d*) spectrum of 3m

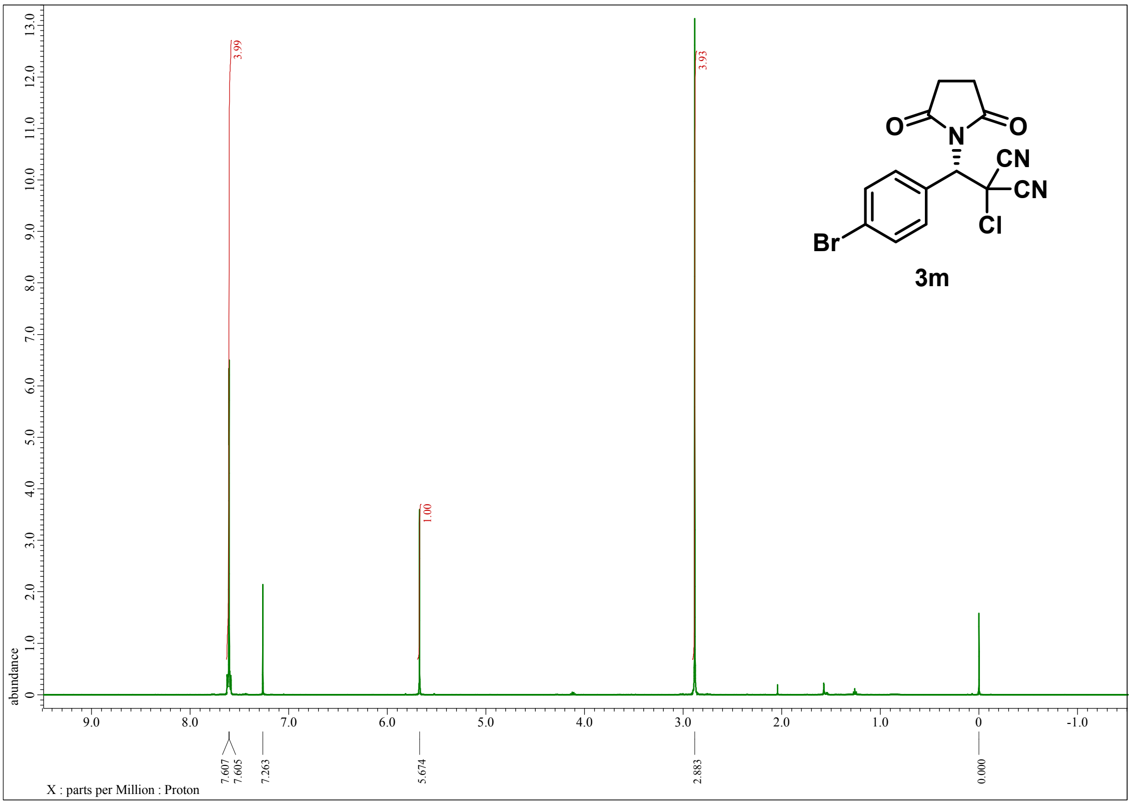


$^{13}$CNMR (125 MHz, chloroform-*d*) spectrum of 3m

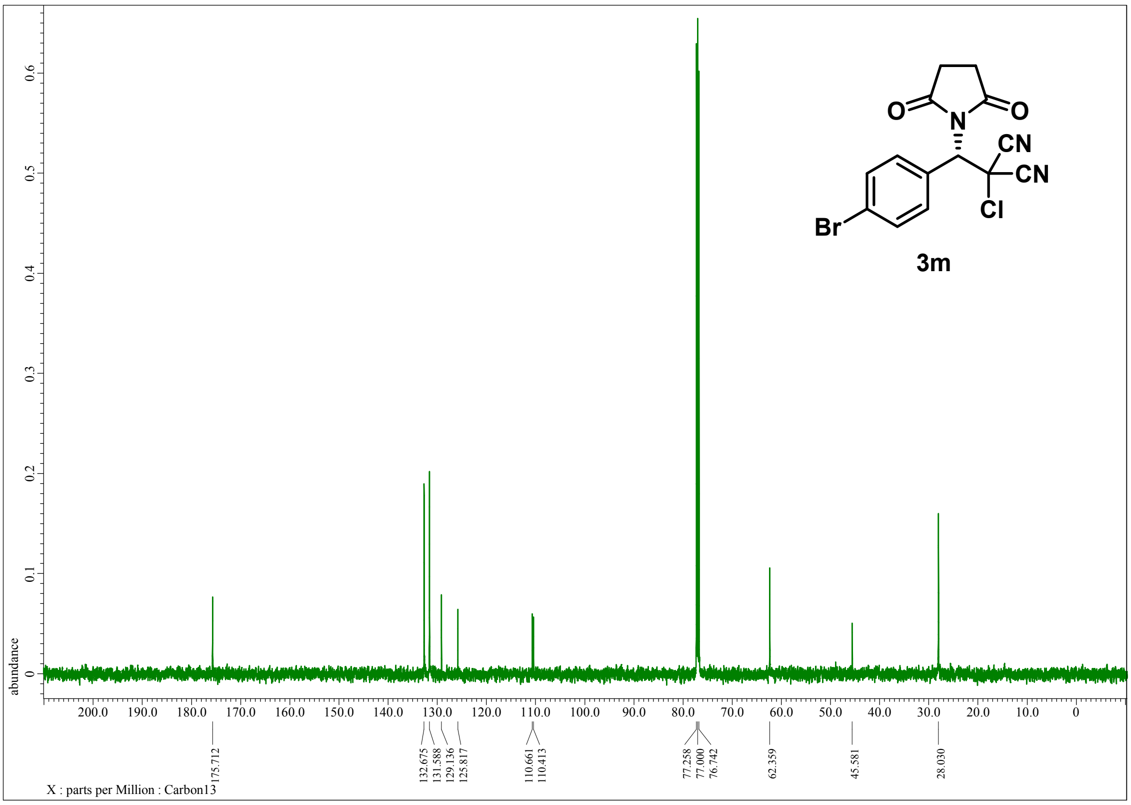

<sup></sup>

$^{1}$HNMR (400 MHz, chloroform-*d*) spectrum of 3n

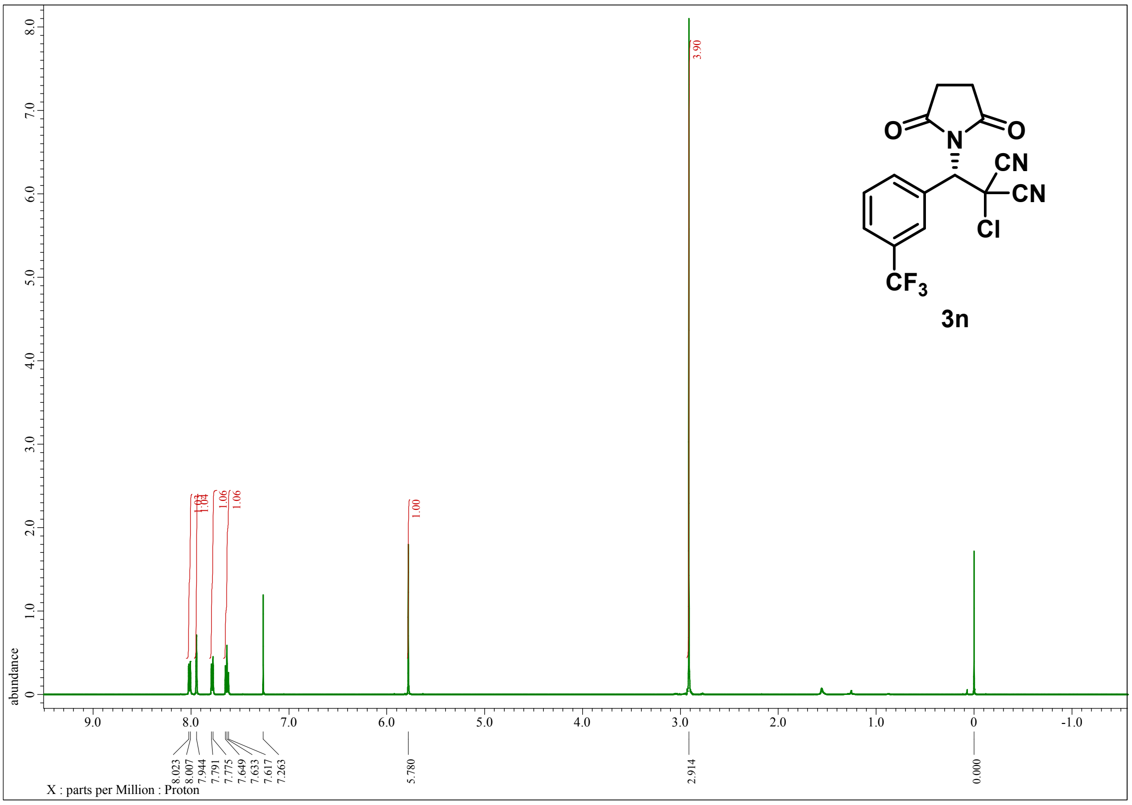


$^{13}$CNMR (125 MHz, chloroform-*d*) spectrum of 3n

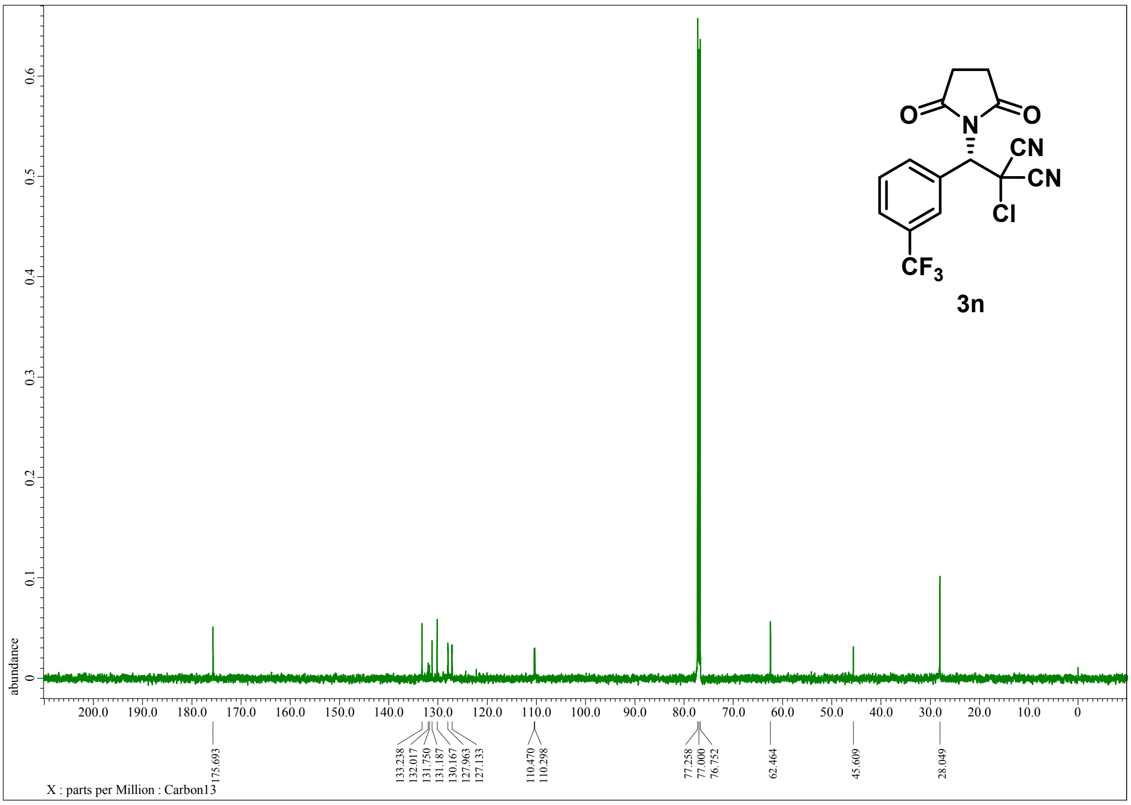

$^{1}$HNMR (400 MHz, chloroform-*d*) spectrum of 3o

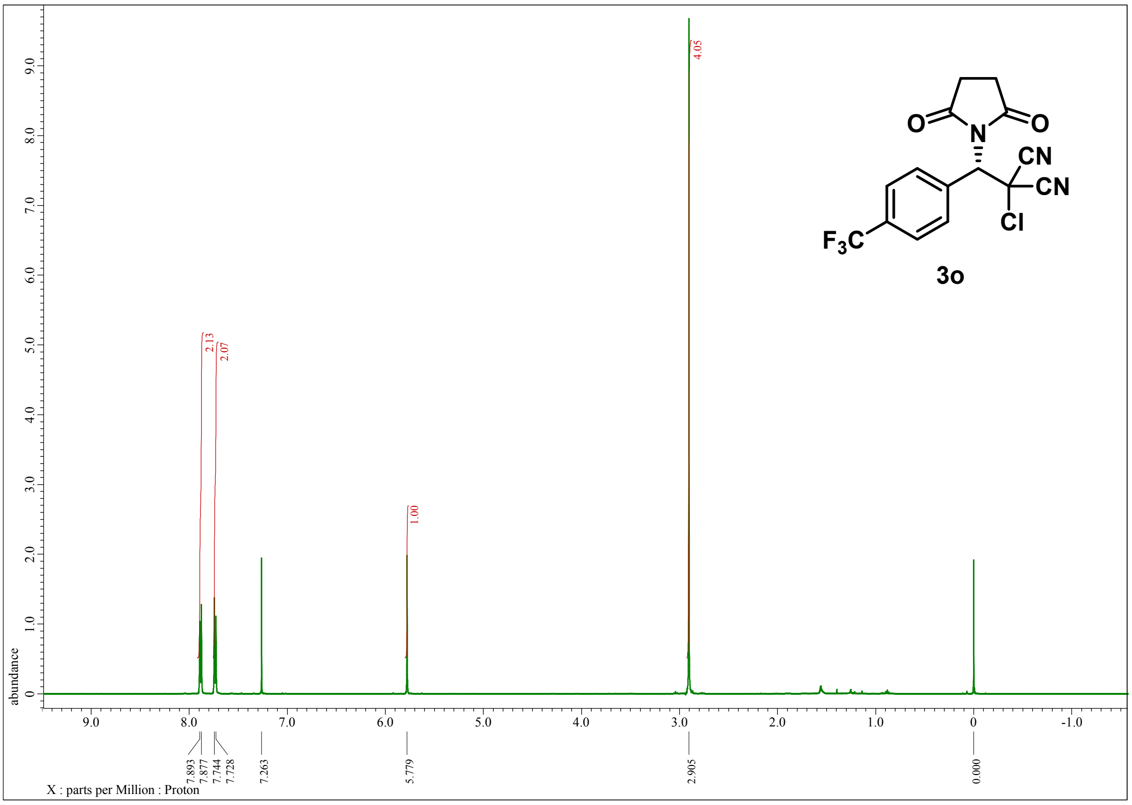


$^{13}$CNMR (125 MHz, chloroform-*d*) spectrum of 3o

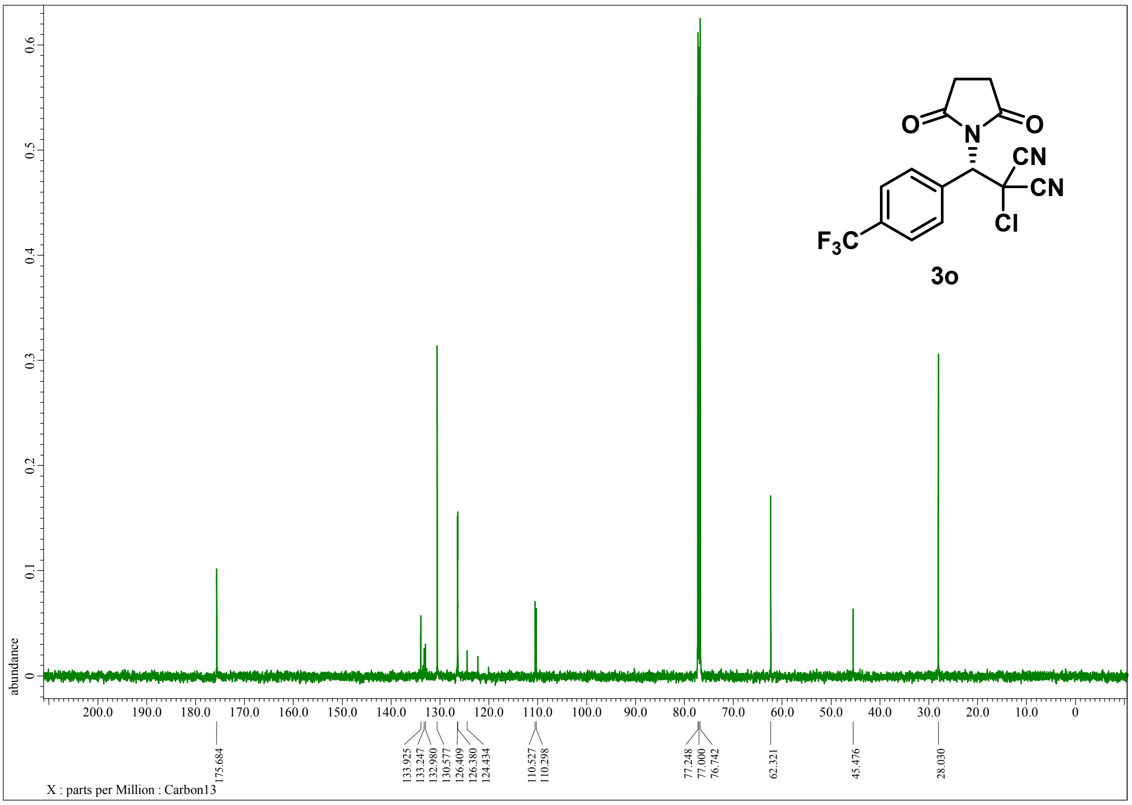

$^{1}$HNMR (400 MHz, chloroform-*d*) spectrum of 3p

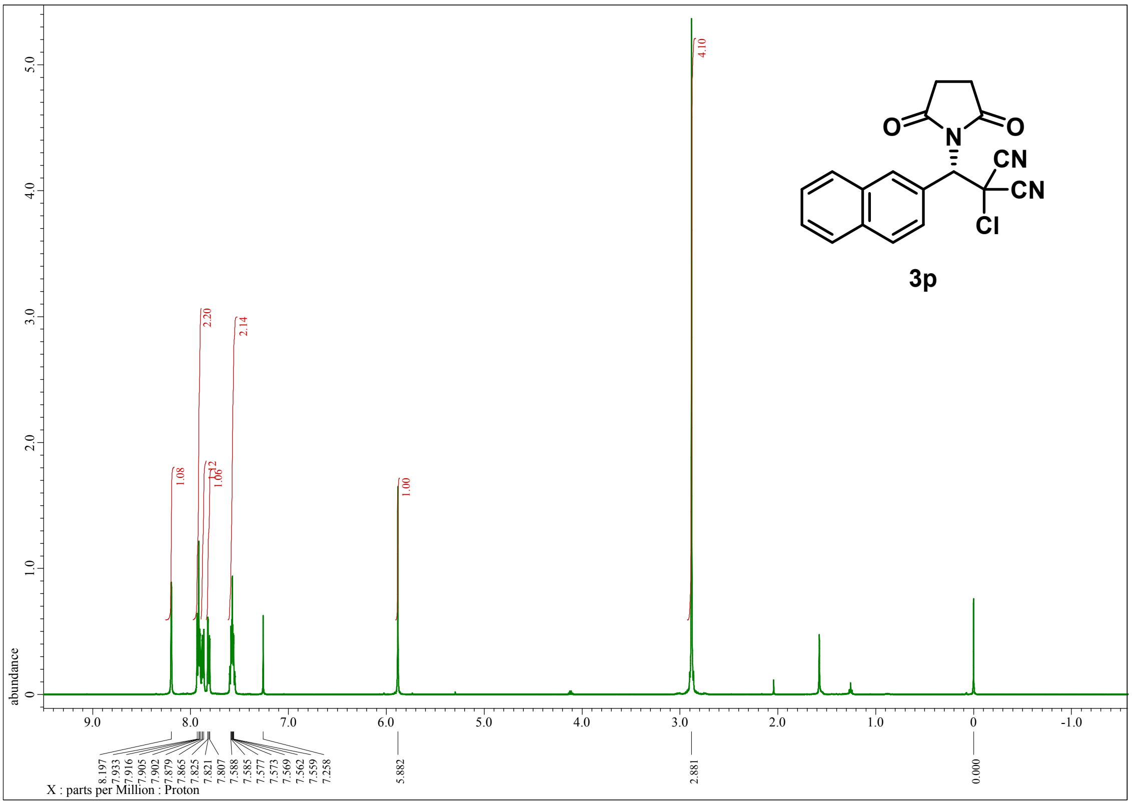


$^{13}$CNMR (125 MHz, chloroform-*d*) spectrum of 3p

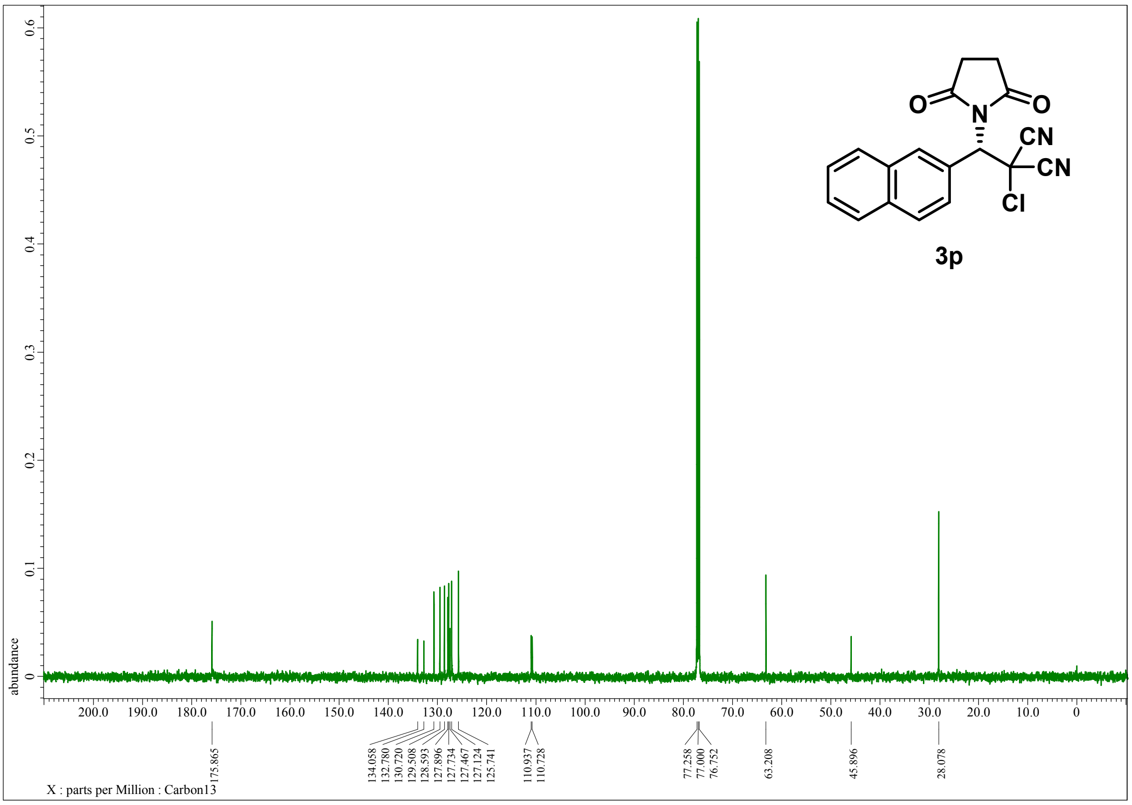

$^{1}$HNMR (400 MHz, chloroform-*d*) spectrum of 3q

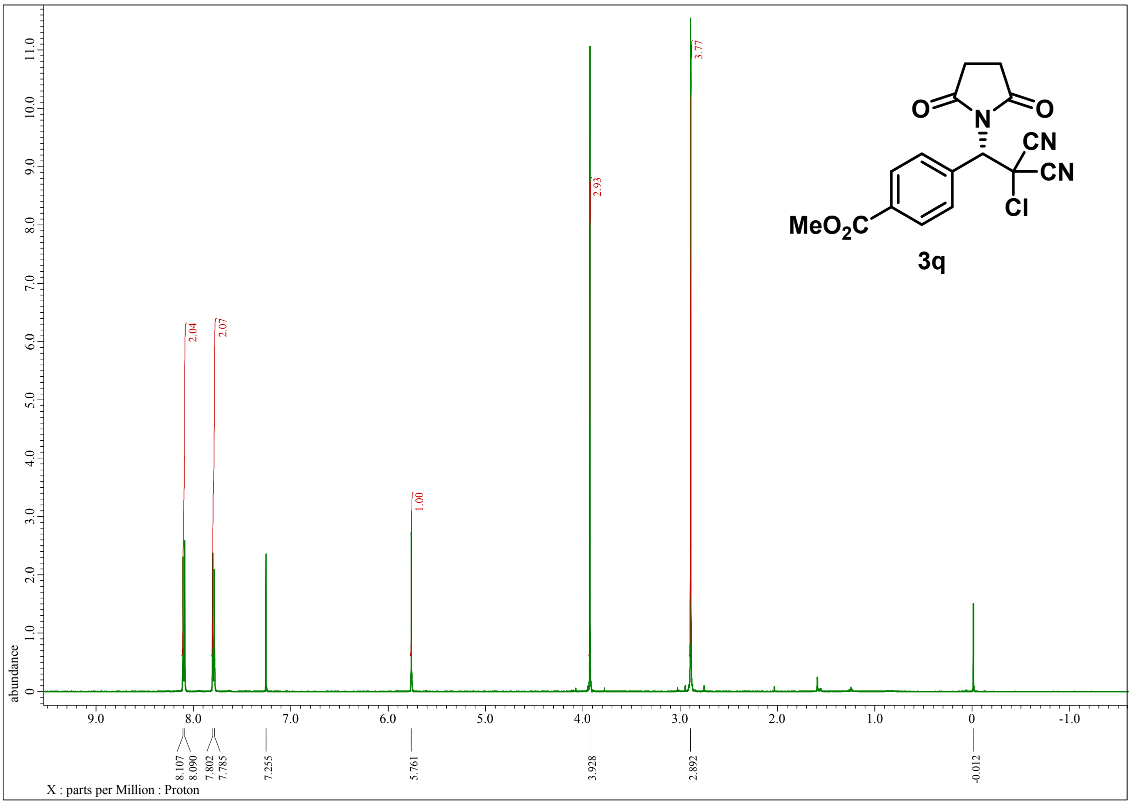


$^{13}$CNMR (125 MHz, chloroform-*d*) spectrum of 3q

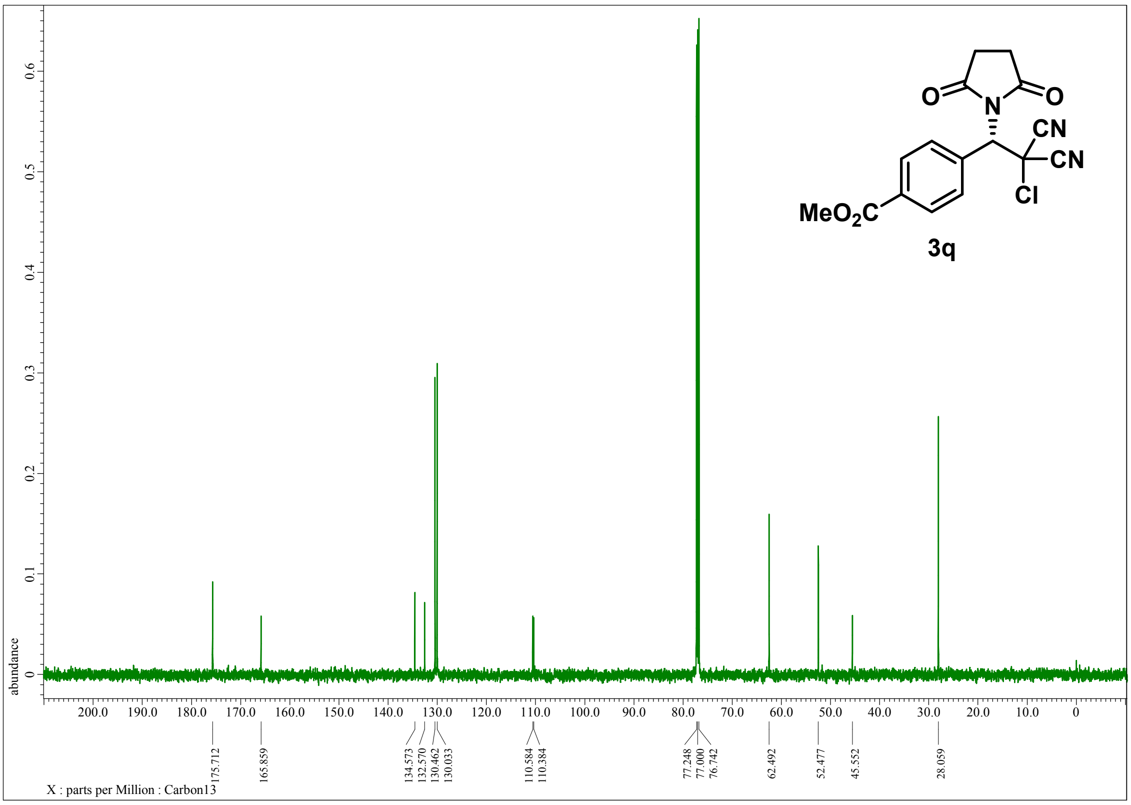

$^{1}$HNMR (400 MHz, chloroform-*d*) spectrum of 3r

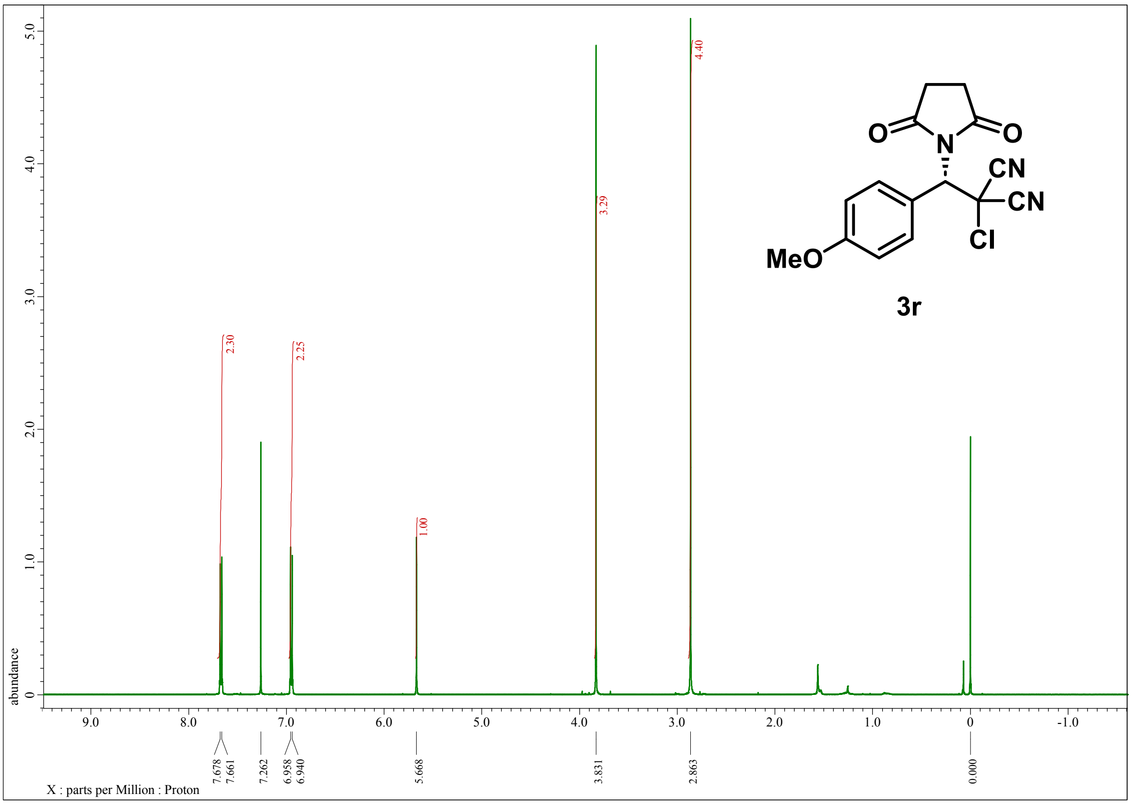


$^{13}$CNMR (125 MHz, chloroform-*d*) spectrum of 3r

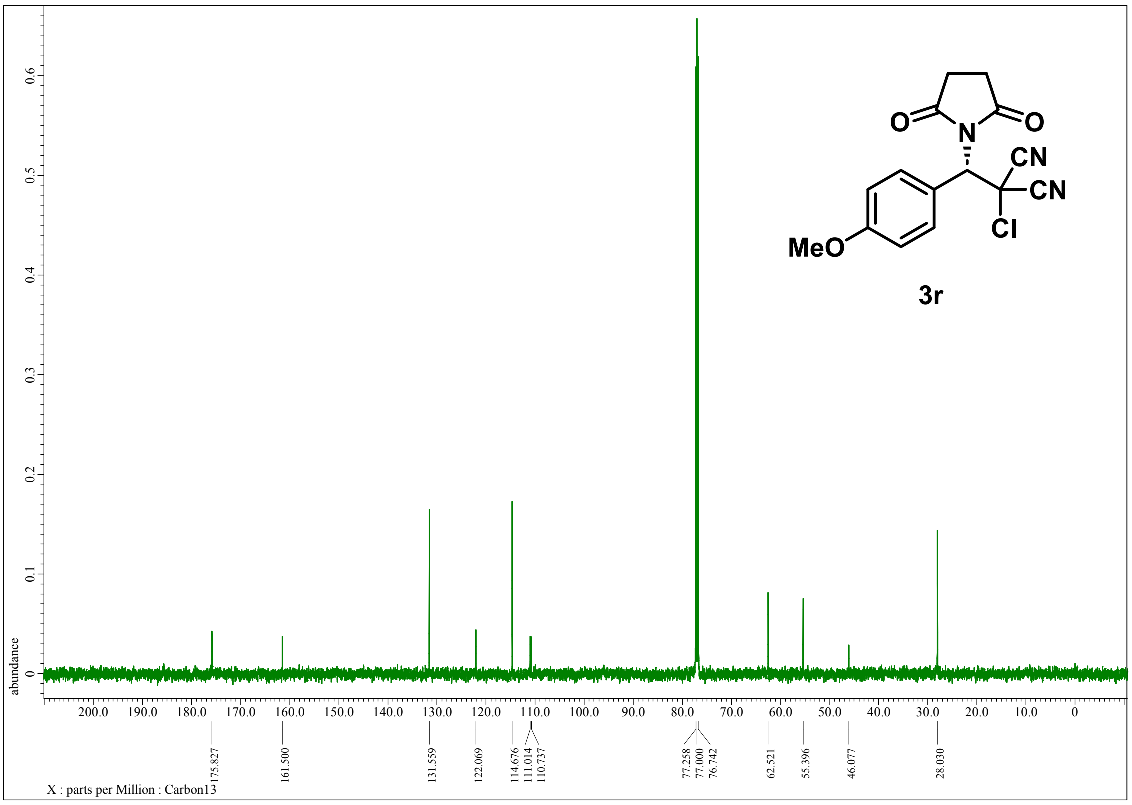

$^{1}$HNMR (400 MHz, chloroform-*d*) spectrum of 3s

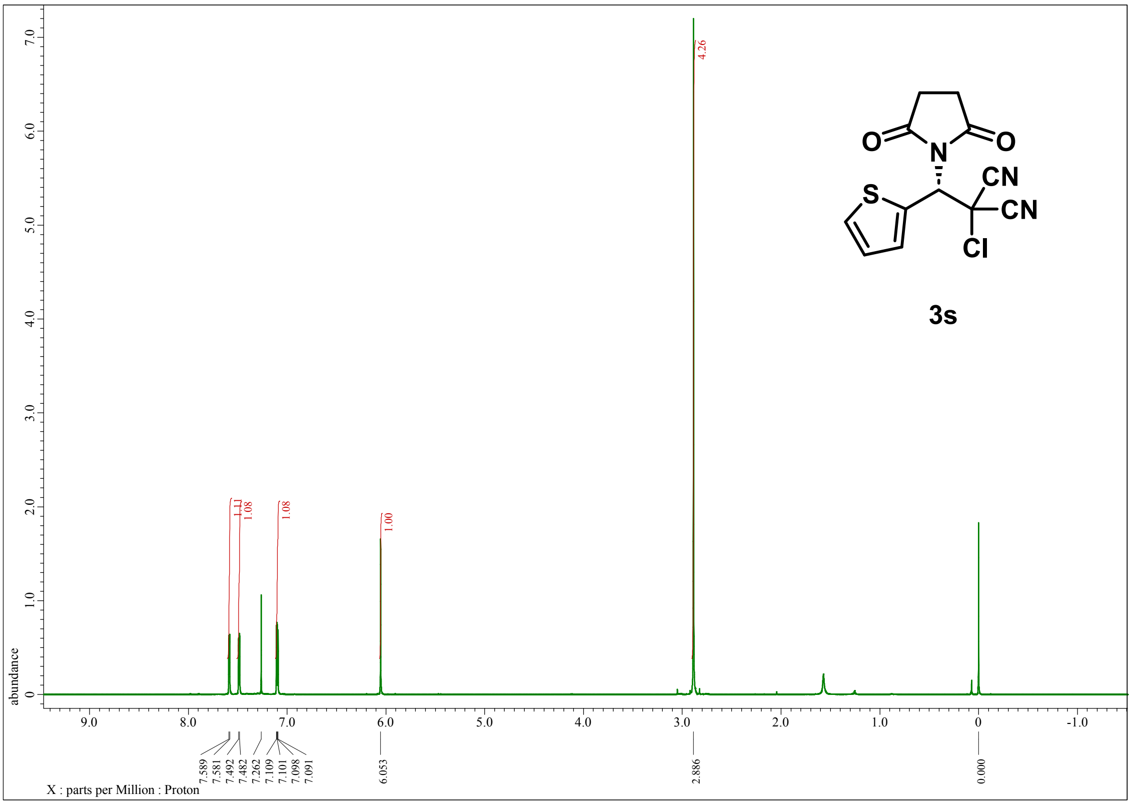


$^{13}$CNMR (125 MHz, chloroform-*d*) spectrum of 3s

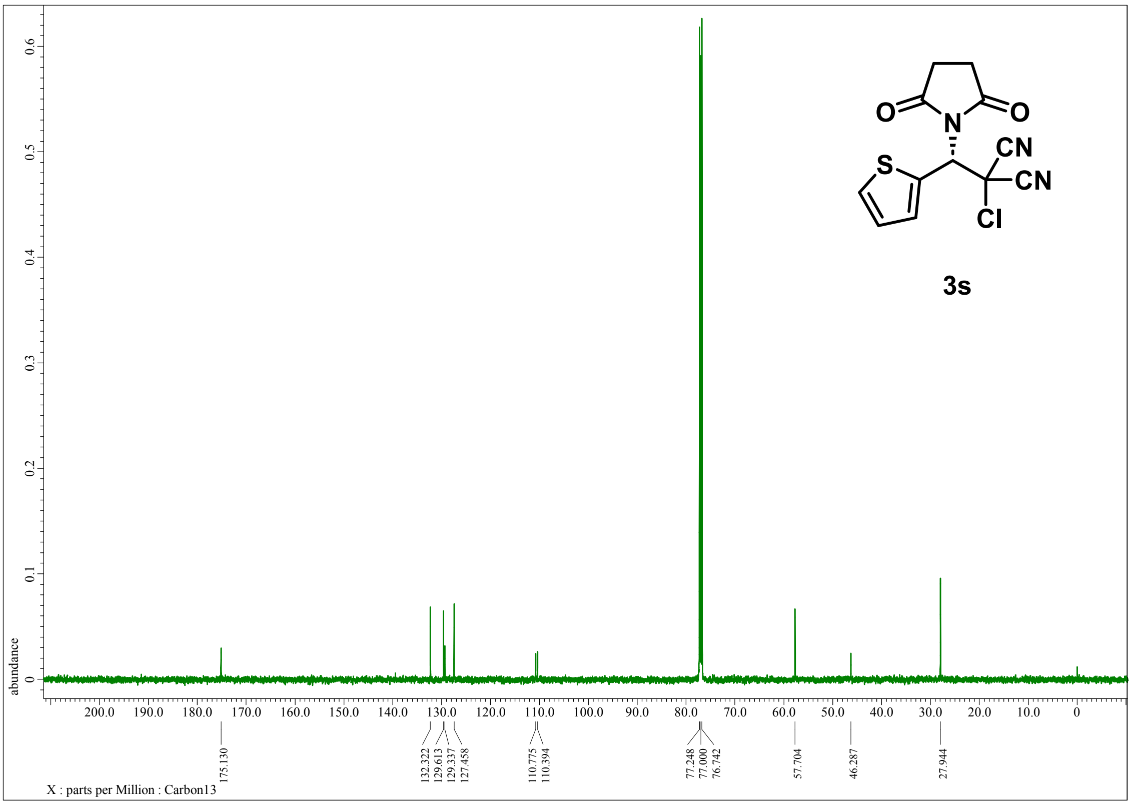

$^{1}$HNMR (400 MHz, chloroform-*d*) spectrum of 3t

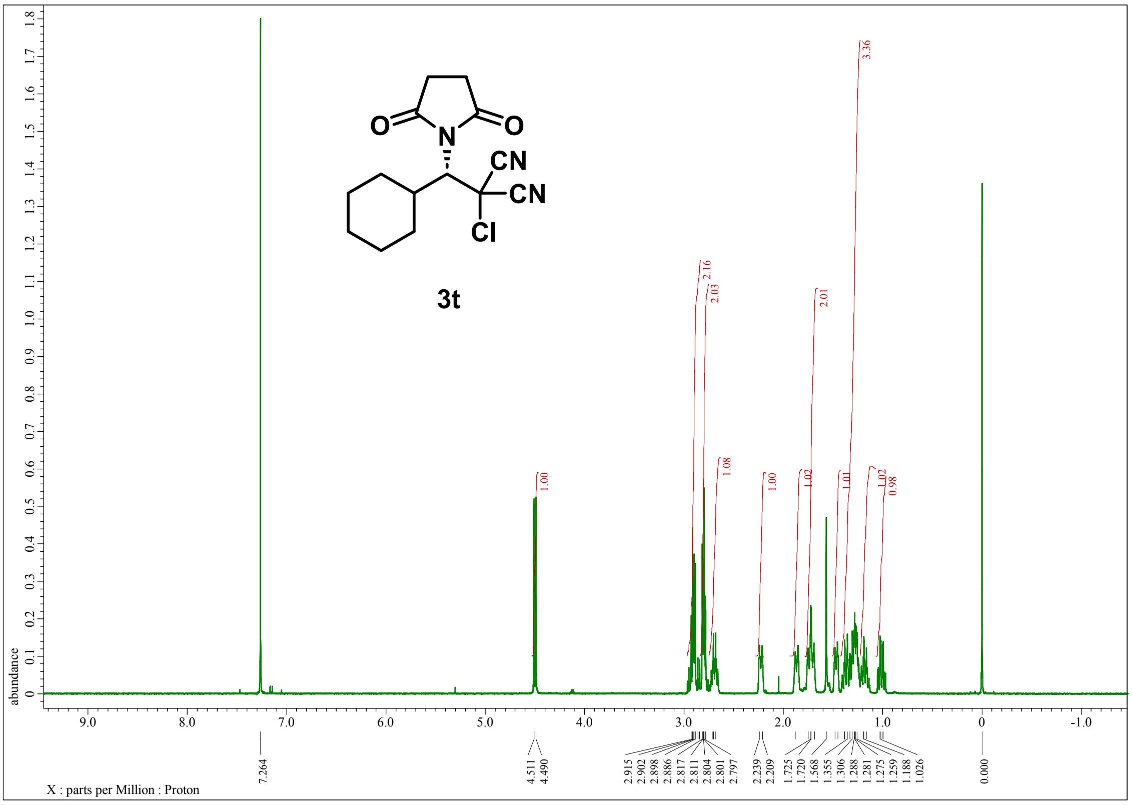

$^{13}$CNMR (125 MHz, chloroform-*d*) spectrum of 3t

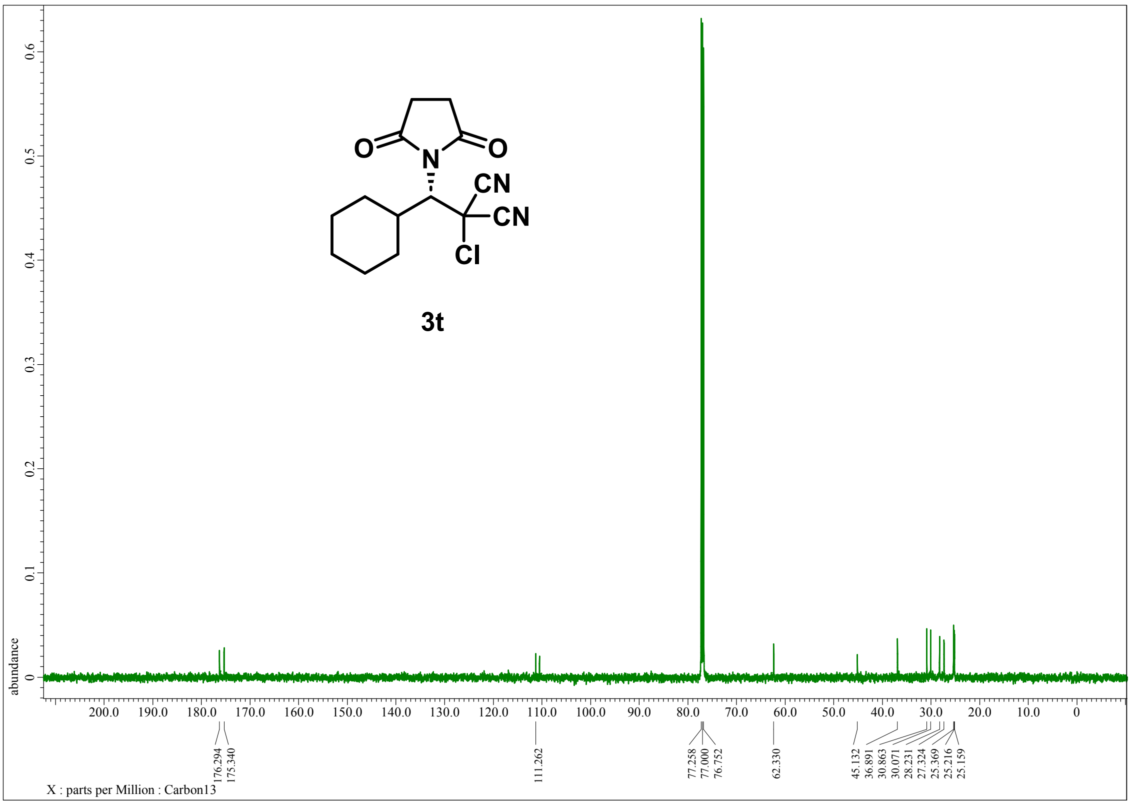

$^{1}$HNMR (400 MHz, chloroform-*d*) spectrum of 3u

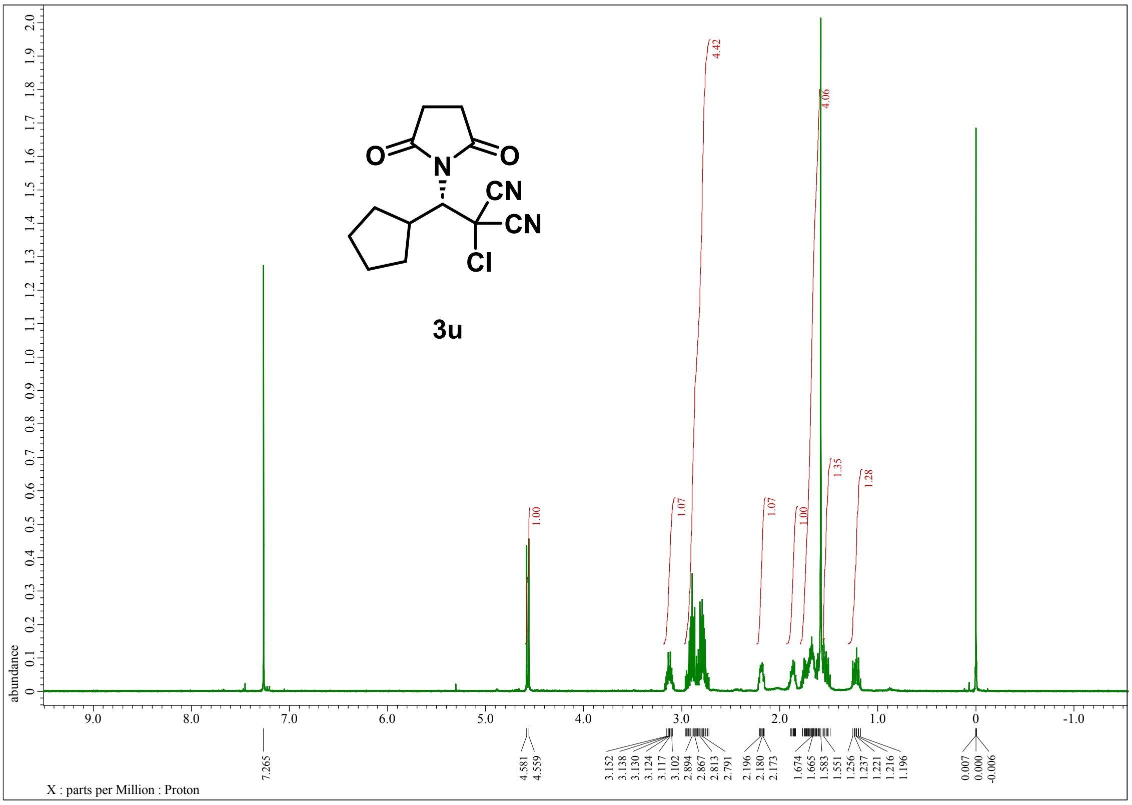


$^{13}$CNMR (125 MHz, chloroform-*d*) spectrum of 3u

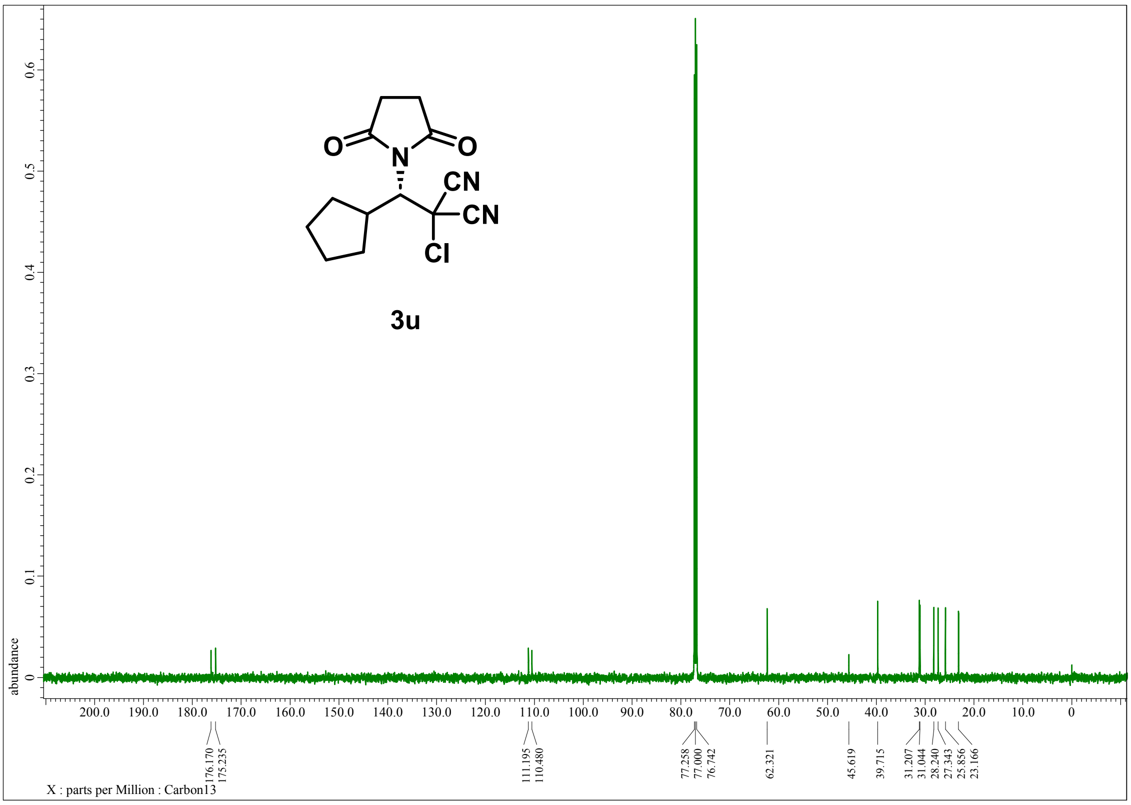

$^{1}$HNMR (500 MHz, chloroform-*d*) spectrum of 4a

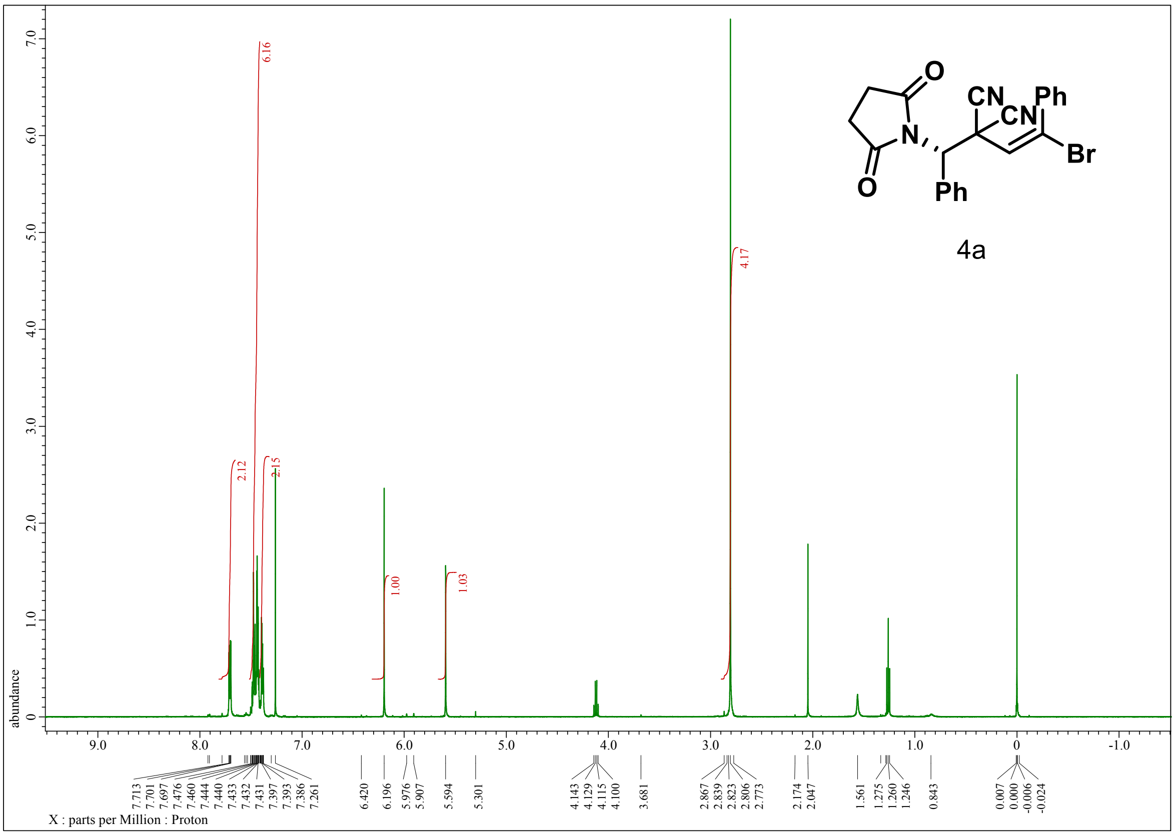


$^{13}$CNMR (125 MHz, chloroform-*d*) spectrum of 4a

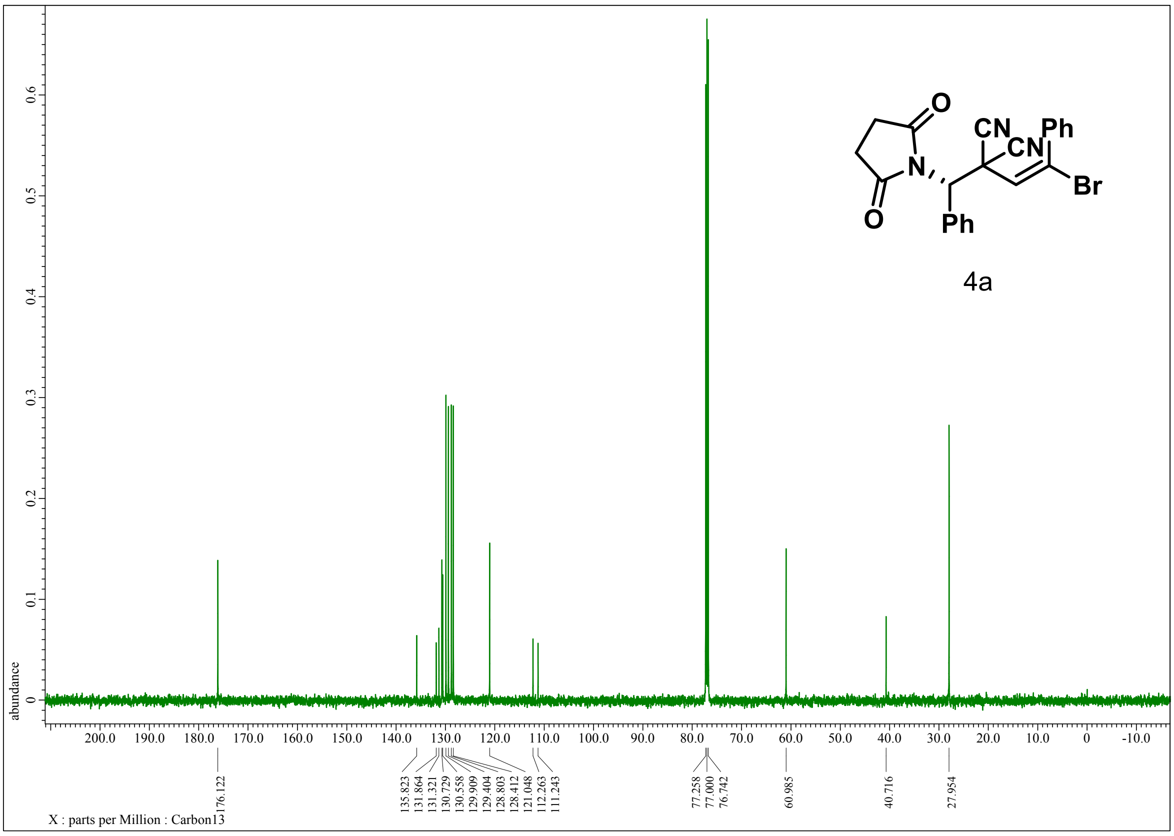

HNMR (400 MHz, acetone-$d6$) spectrum of 4b

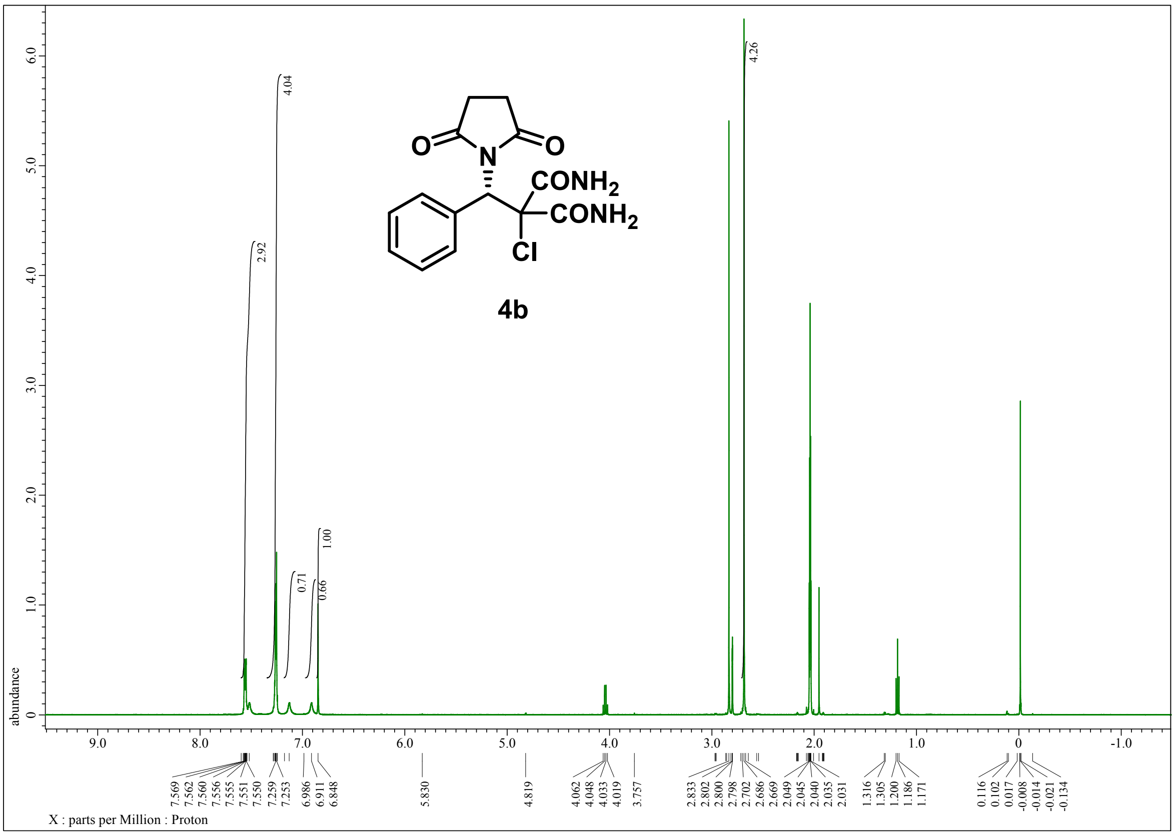

$^{13}$CNMR (125 MHz, acetone-$d6$) spectrum of 4b

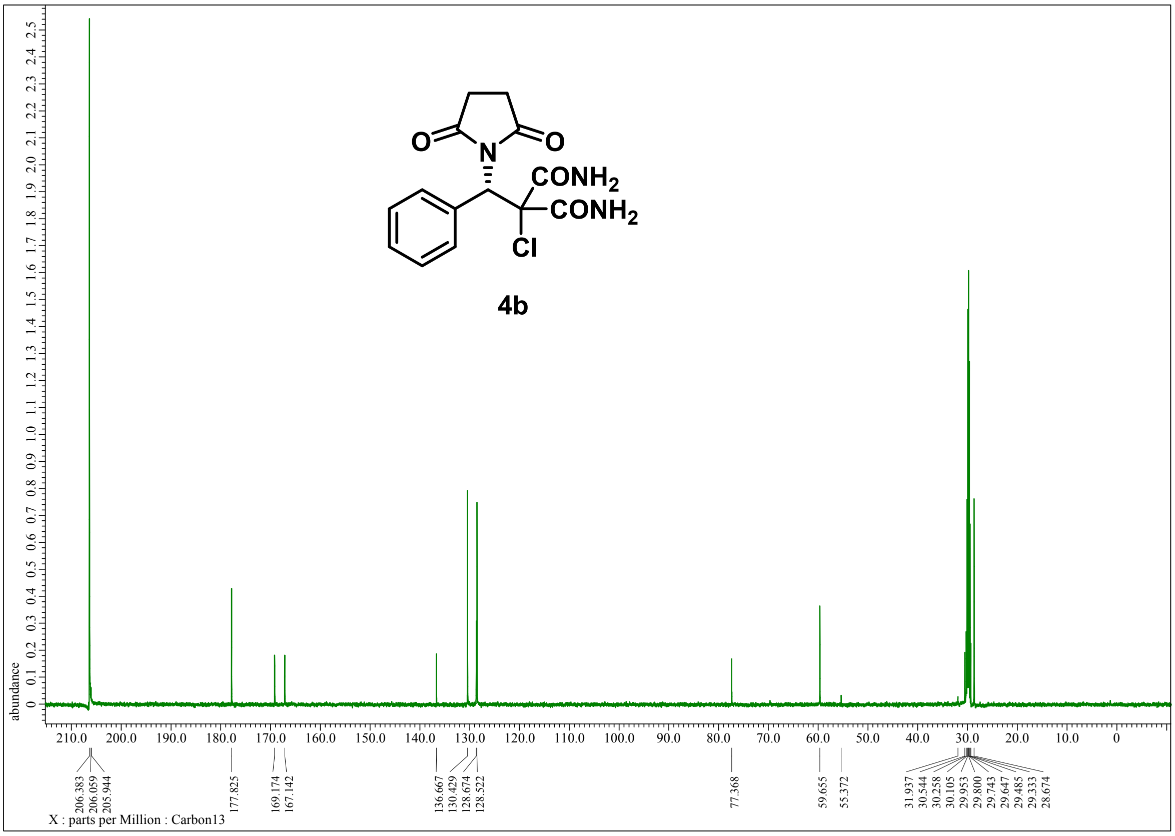

$^{1}$HNMR (400 MHz, acetone-*d*6) spectrum of 4c

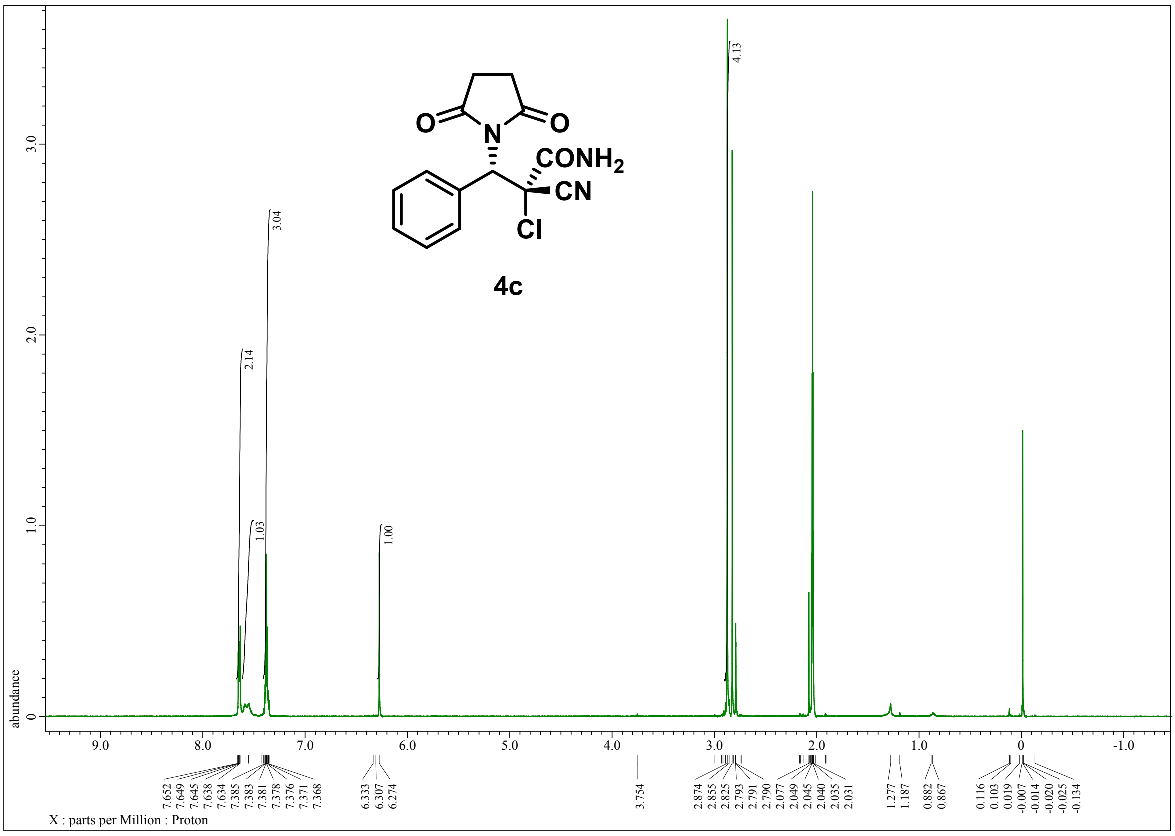


$^{13}$CNMR (125 MHz, acetone-*d*6) spectrum of 4c

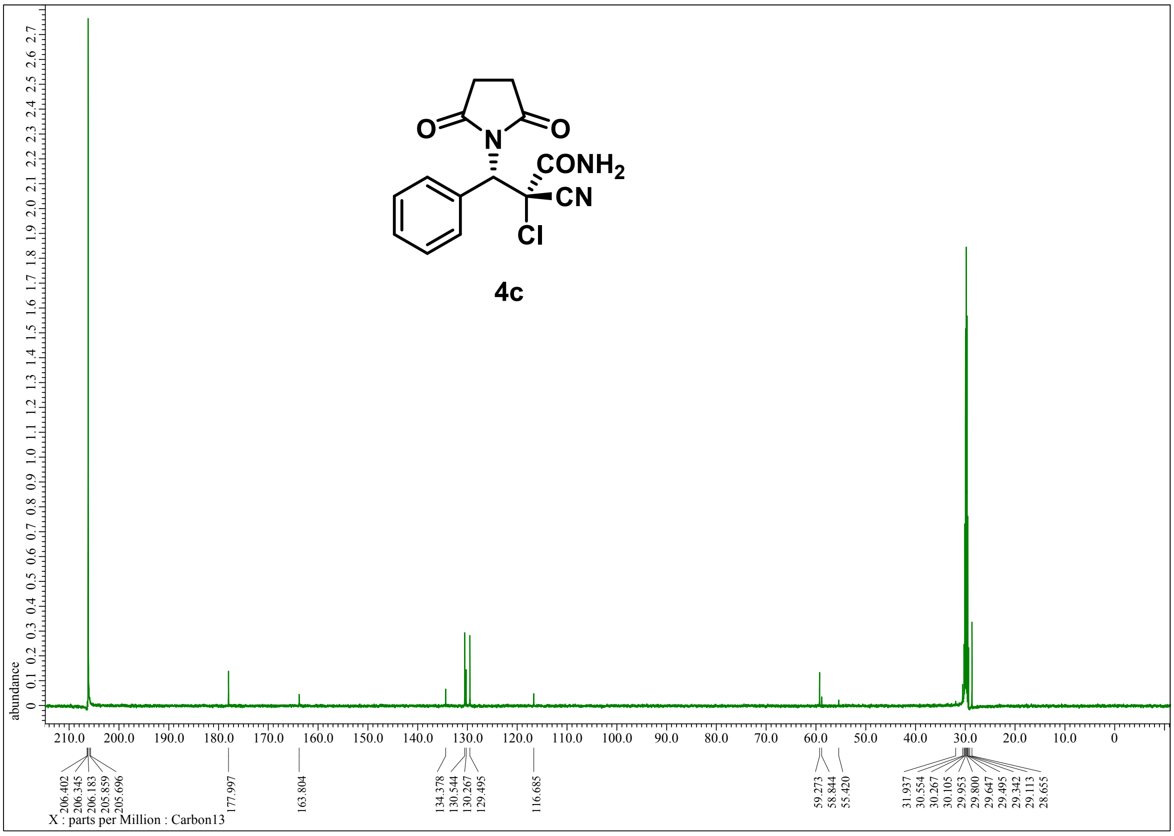

**13. HPLC chart**

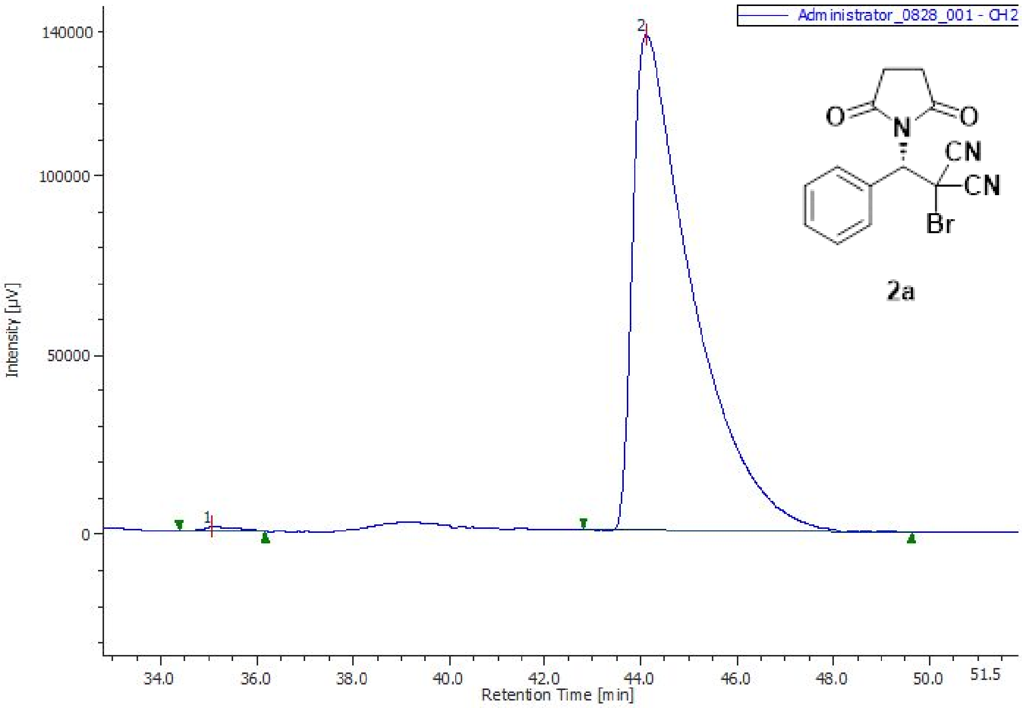


| | tR | Area (µV・min) | Area (%) |
|---|---|---|---|
| 1 | 35.075 | 61644 | 0.518 |
| 2 | 44.117 | 11830176 | 99.482 |

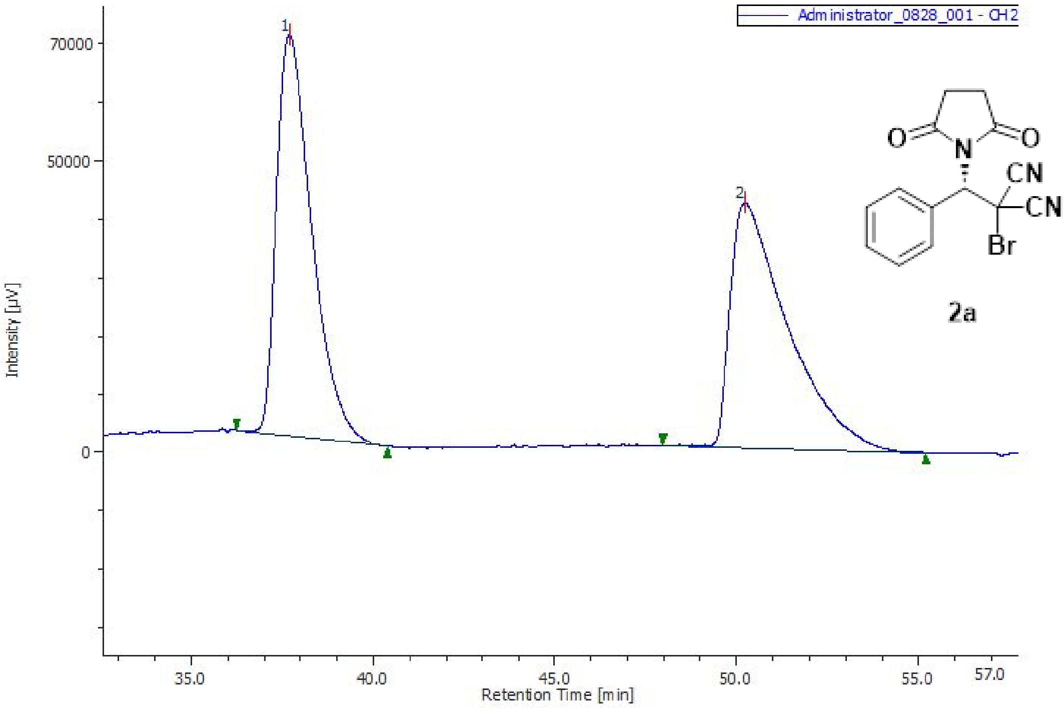


| | tR | Area (µV ・min) | Area (%) |
|---|---|---|---|
| 1 | 37.700 | 4649945 | 50.158 |
| 2 | 50.225 | 4620686 | 49.842 |

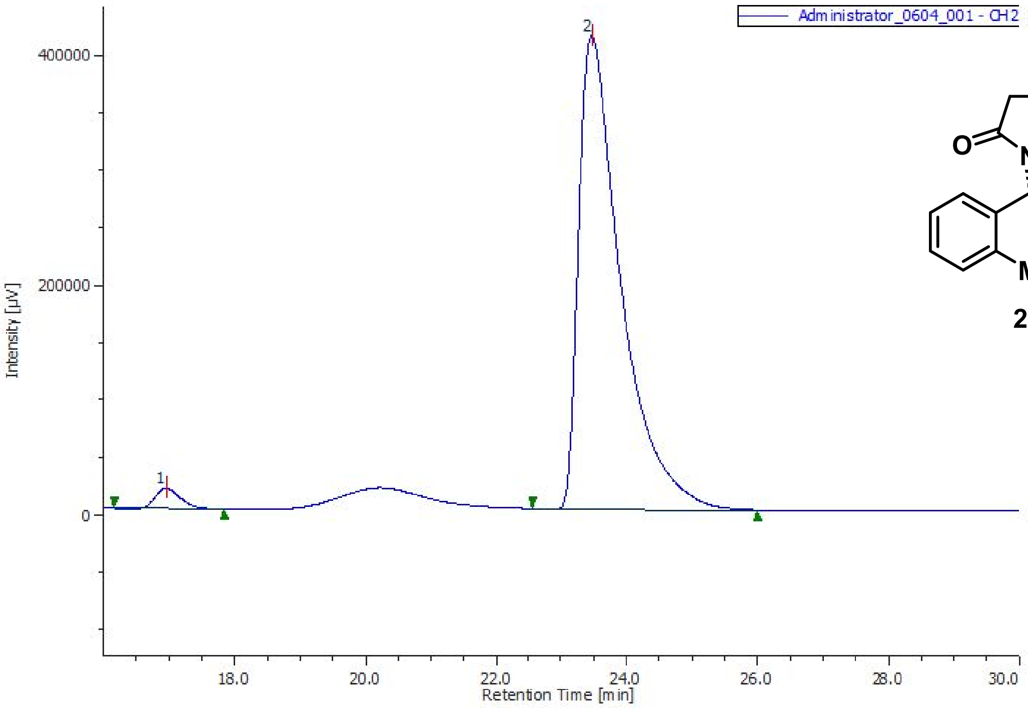


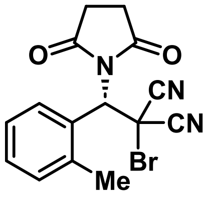


**2b**

| | tR | Area (μV・min) | Area (%) |
|---|---|---|---|
| 1 | 16.950 | 466476 | 4.126 |
| 2 | 23.475 | 18443864 | 95.874 |

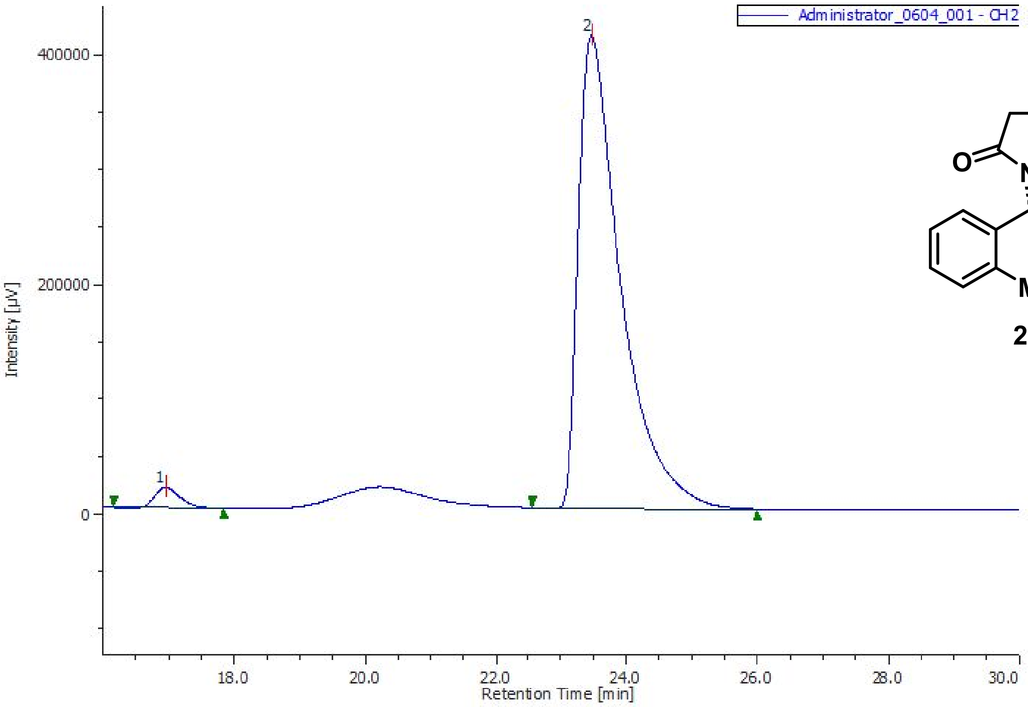


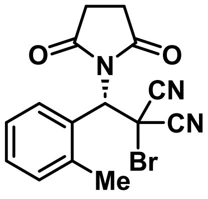


**2b**

| | tR | Area (μV ・min) | Area (%) |
|---|---|---|---|
| 1 | 17.517 | 23535502 | 49.926 |
| 2 | 24.600 | 23605496 | 50.074 |

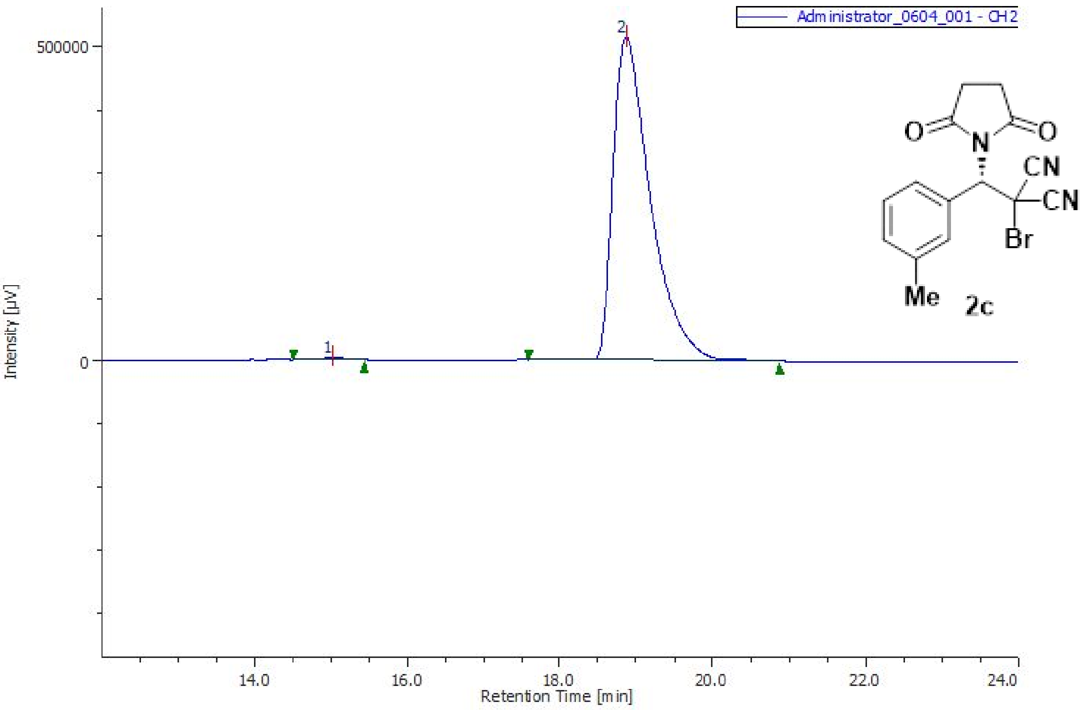


| | tR | Area (μV・min) | Area (%) |
|---|---|---|---|
| 1 | 15.017 | 147689 | 1.248 |
| 2 | 18.875 | 17678846 | 99.172 |

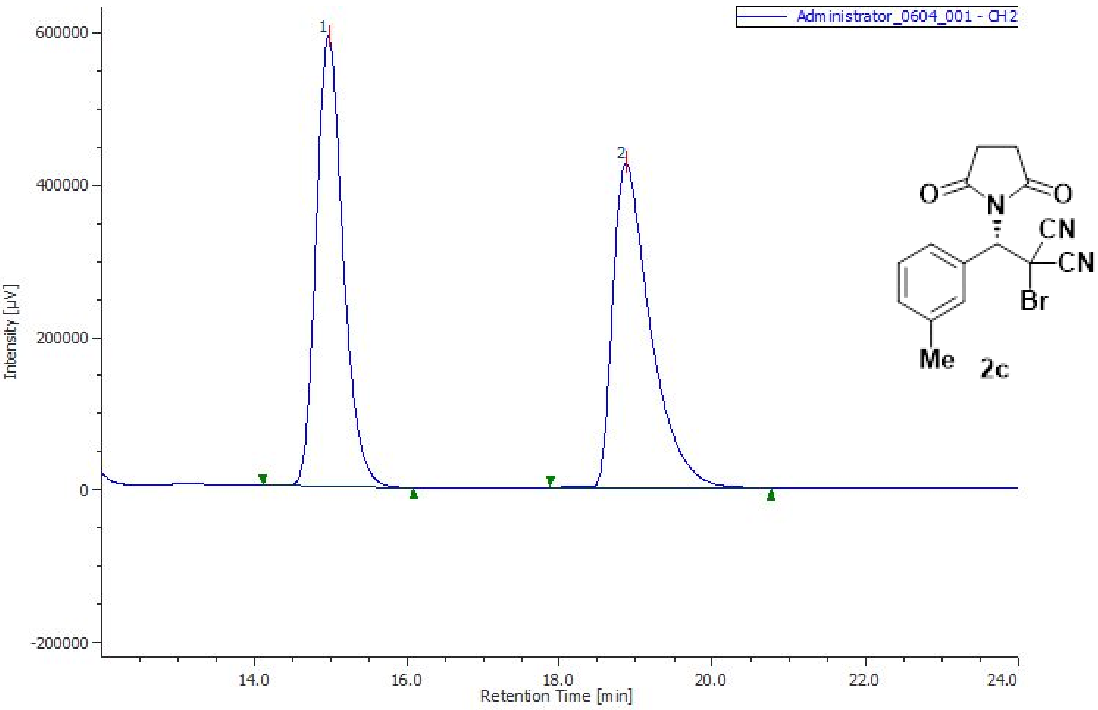


| | tR | Area (μV ・min) | Area (%) |
|---|---|---|---|
| 1 | 14.967 | 14603817 | 50.064 |
| 2 | 18.875 | 14566762 | 49.936 |

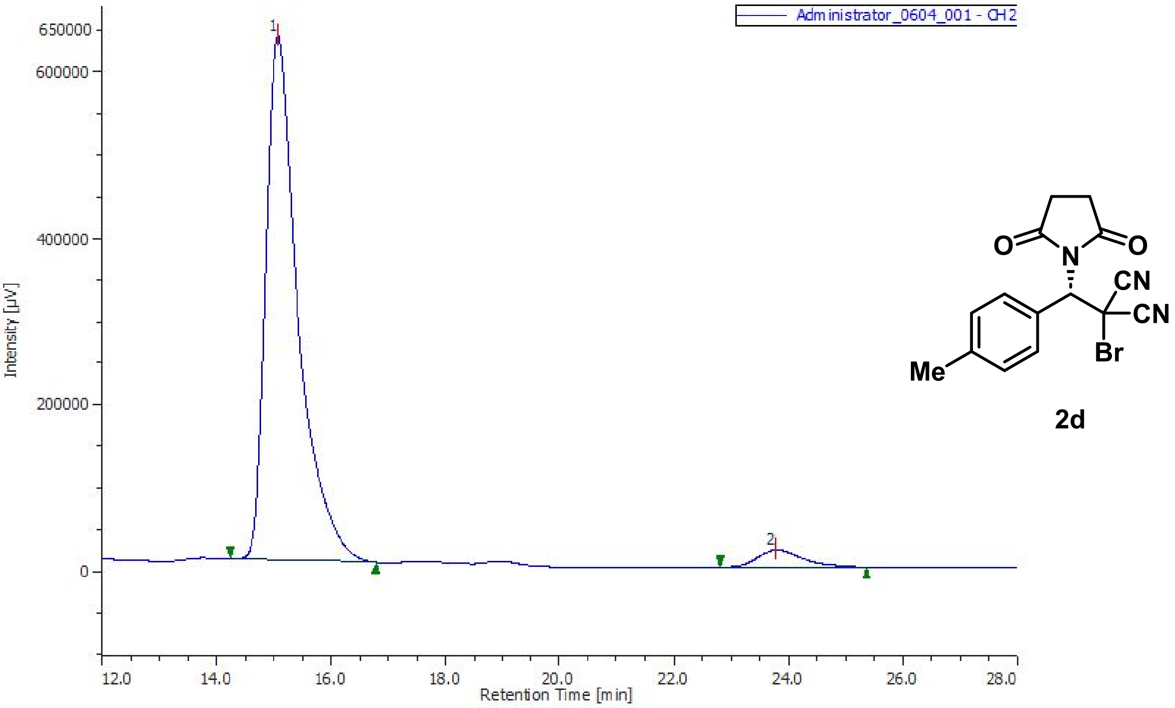


| | tR | Area (μV・min) | Area (%) |
|---|---|---|---|
| 1 | 15.075 | 24580724 | 95.605 |
| 2 | 23.792 | 1129913 | 4.395 |

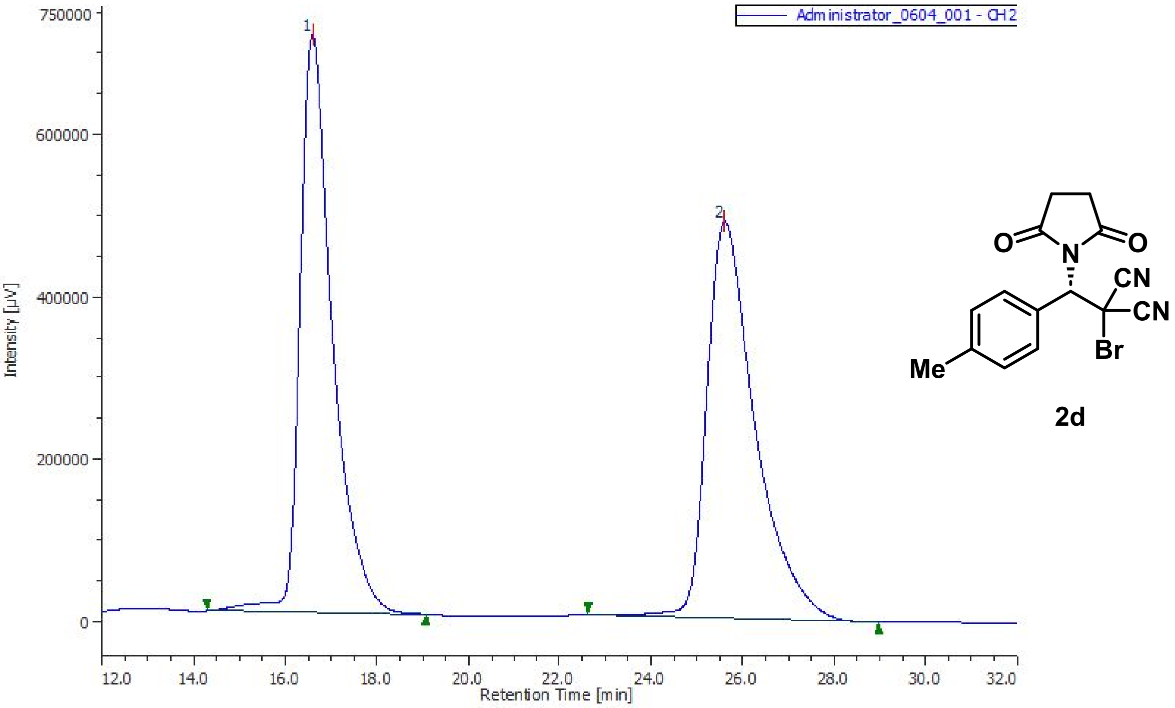


| | tR | Area (μV ・min) | Area (%) |
|---|---|---|---|
| 1 | 16.592 | 35106653 | 49.519 |
| 2 | 25.617 | 35788171 | 50.481 |

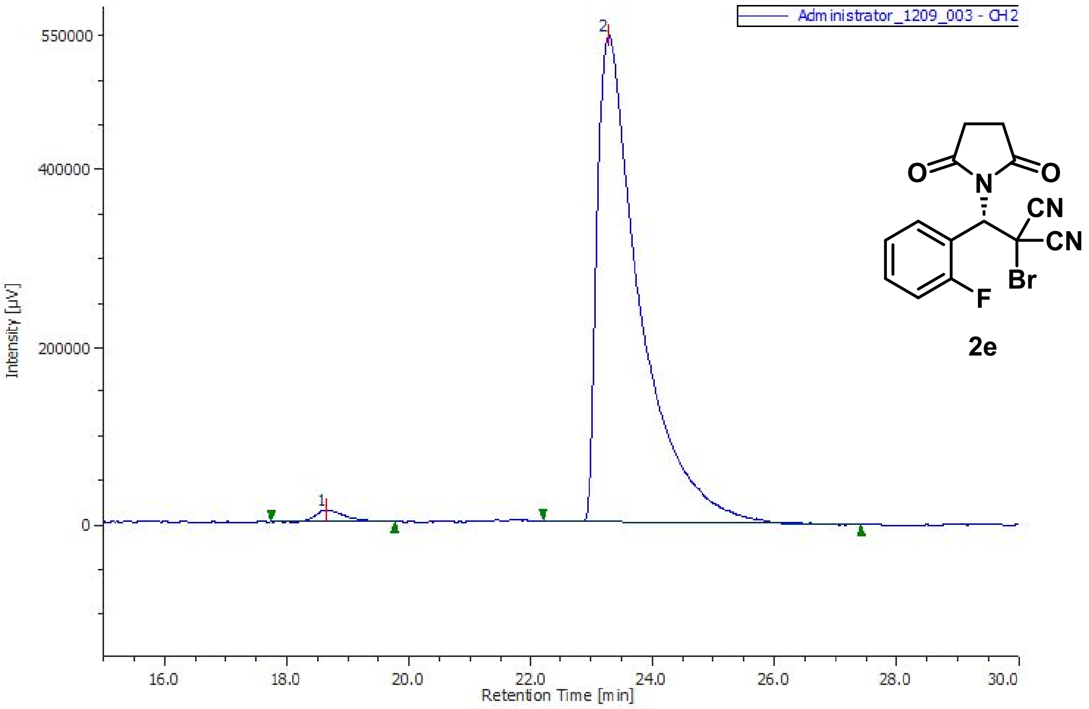


**2e**

| | tR | Area (µV・min) | Area (%) |
|---|---|---|---|
| 1 | 18.658 | 428743 | 1.597 |
| 2 | 23.292 | 26426213 | 98.403 |

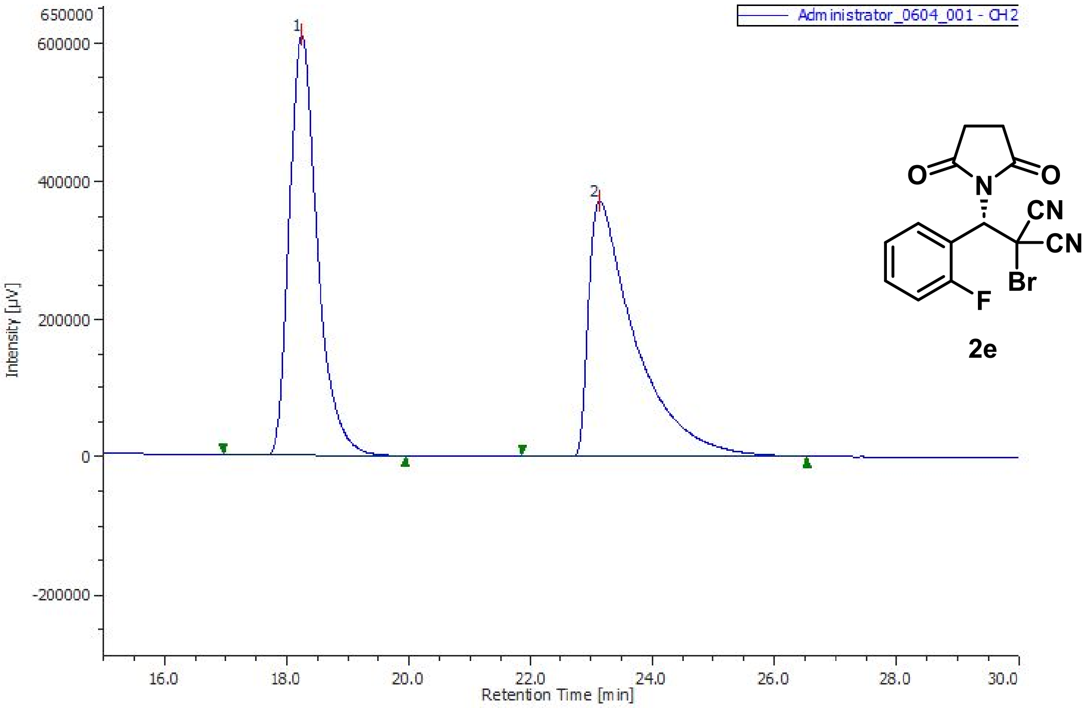


**2e**

| | tR | Area (µV ・min) | Area (%) |
|---|---|---|---|
| 1 | 18.242 | 19421404 | 49.755 |
| 2 | 23.142 | 19612375 | 50.245 |

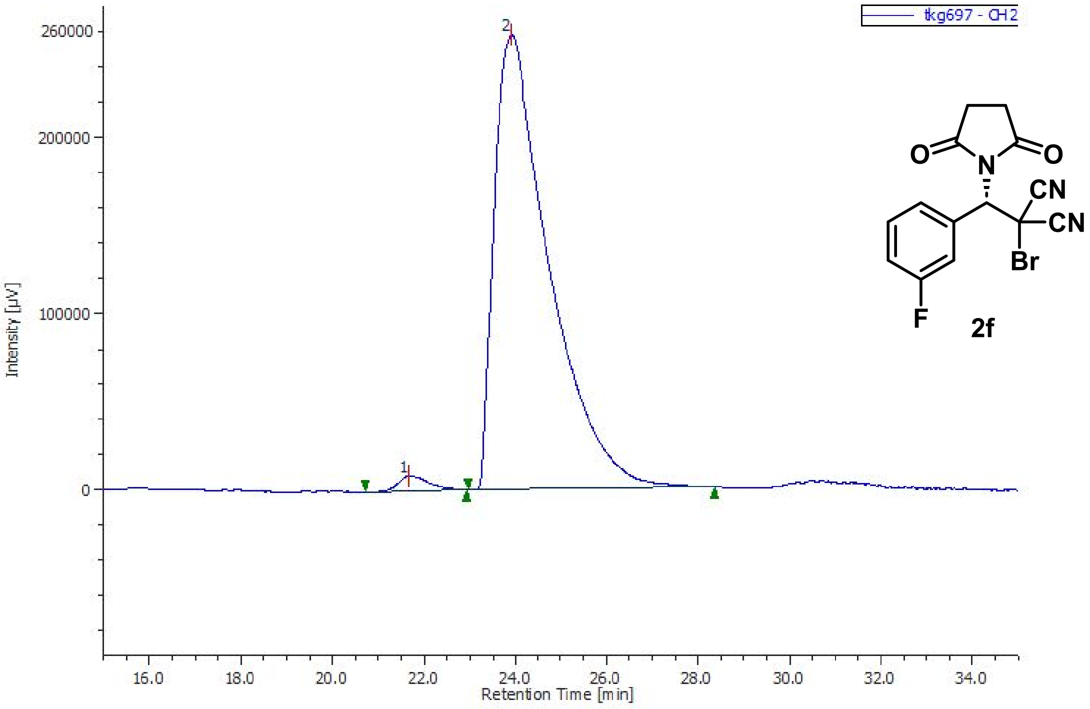


| | tR | Area (μV・min) | Area (%) |
|---|---|---|---|
| 1 | 21.667 | 380277 | 1.711 |
| 2 | 23.908 | 21840370 | 98.289 |

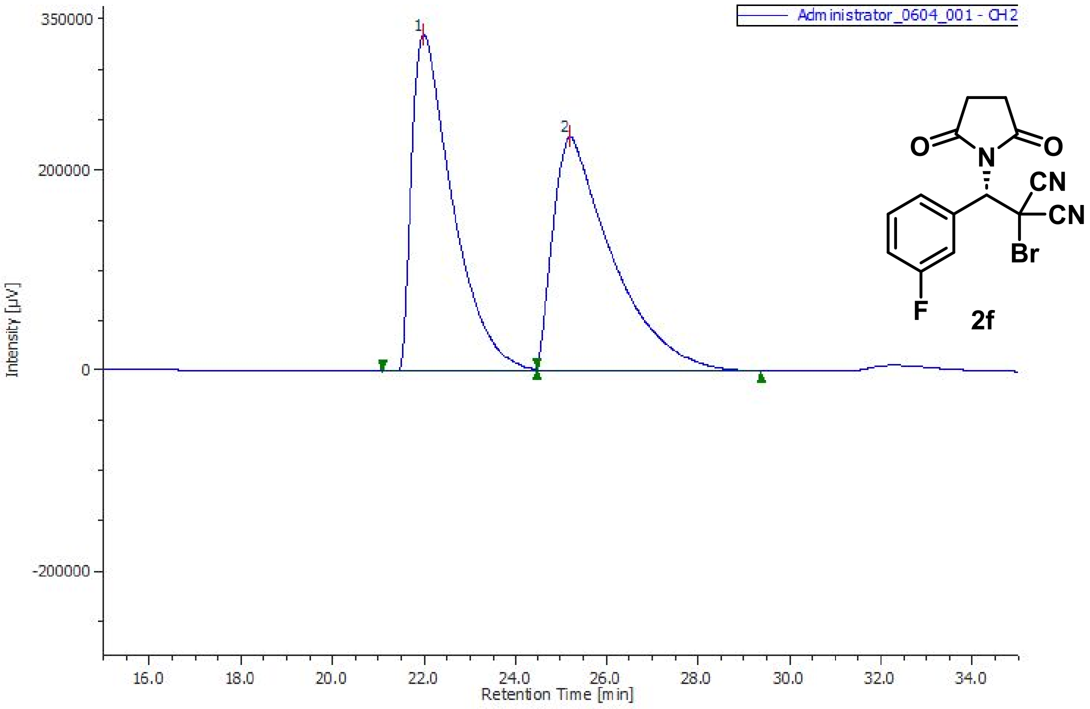


| | tR | Area (μV ・min) | Area (%) |
|---|---|---|---|
| 1 | 21.992 | 20823868 | 49.688 |
| 2 | 25.217 | 21085450 | 50.312 |

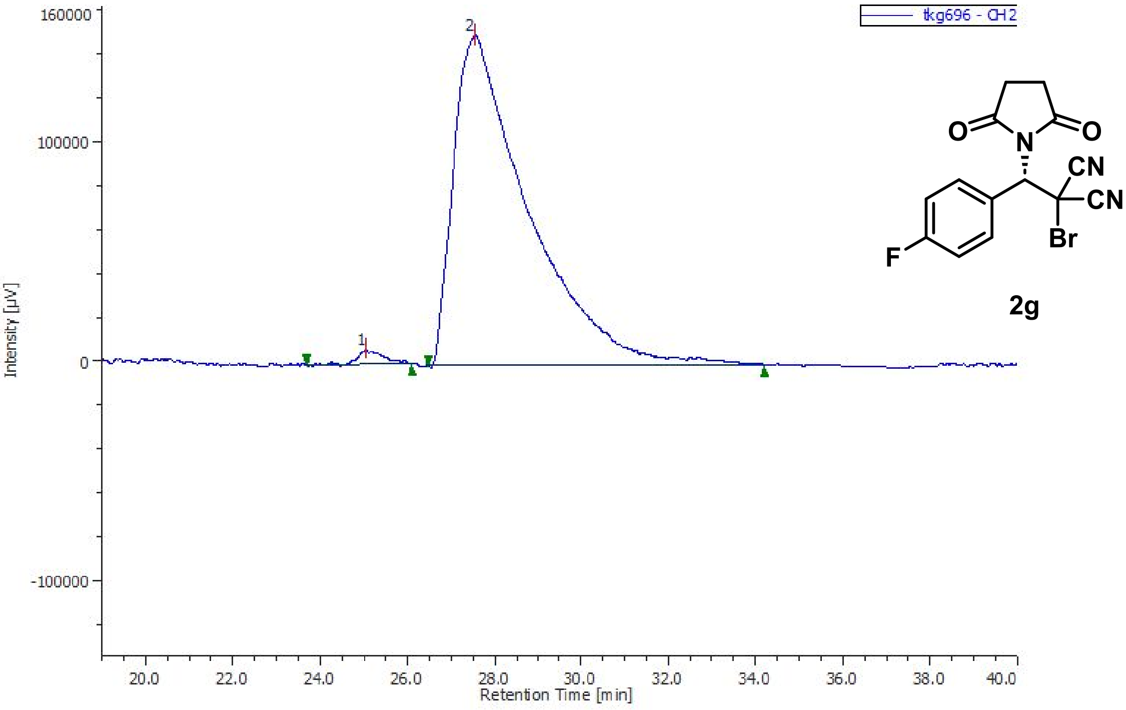


| | tR | Area (µV・min) | Area (%) |
|---|---|---|---|
| 1 | 25.042 | 7073 | 1.532 |
| 2 | 27.542 | 150495 | 98.468 |

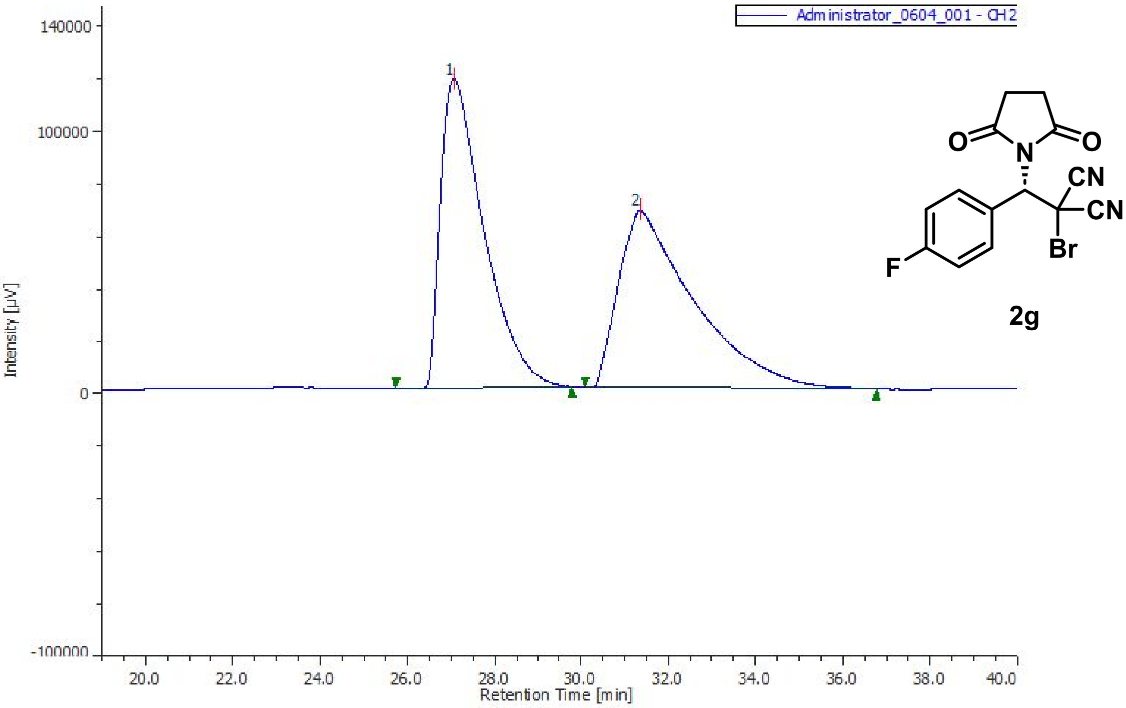


| | tR | Area (µV ・min) | Area (%) |
|---|---|---|---|
| 1 | 27.058 | 8177751 | 50.100 |
| 2 | 31.358 | 8145062 | 49.900 |

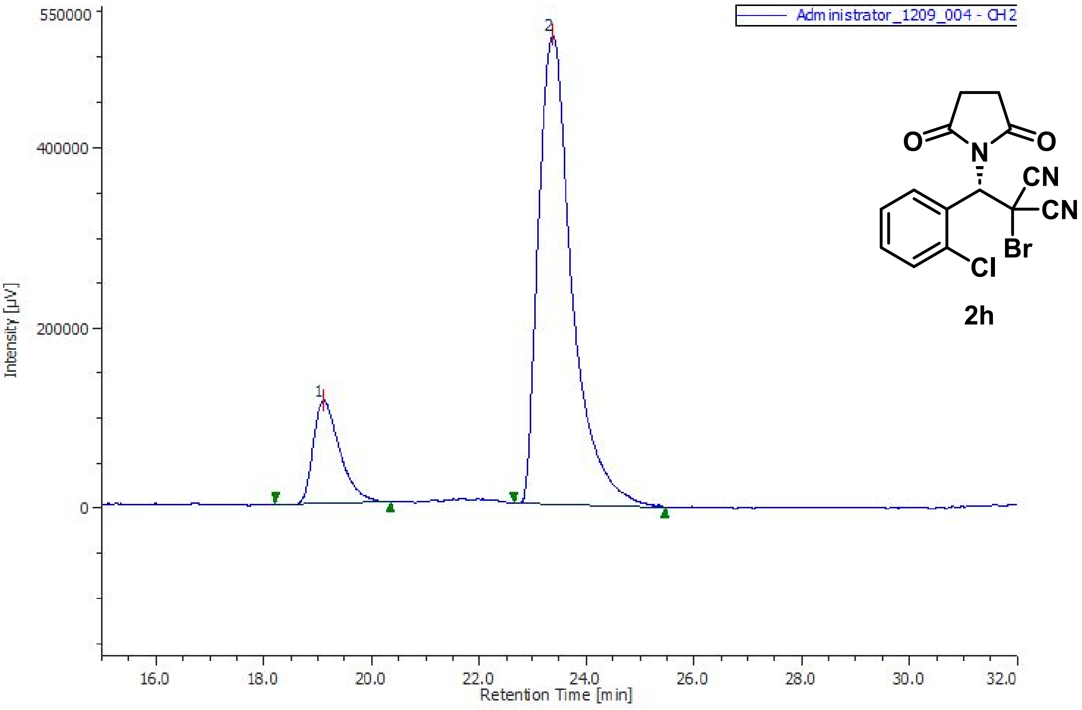


| | tR | Area (μV・min) | Area (%) |
|---|---|---|---|
| 1 | 19.108 | 3834351 | 14.607 |
| 2 | 23.392 | 22416282 | 85.393 |

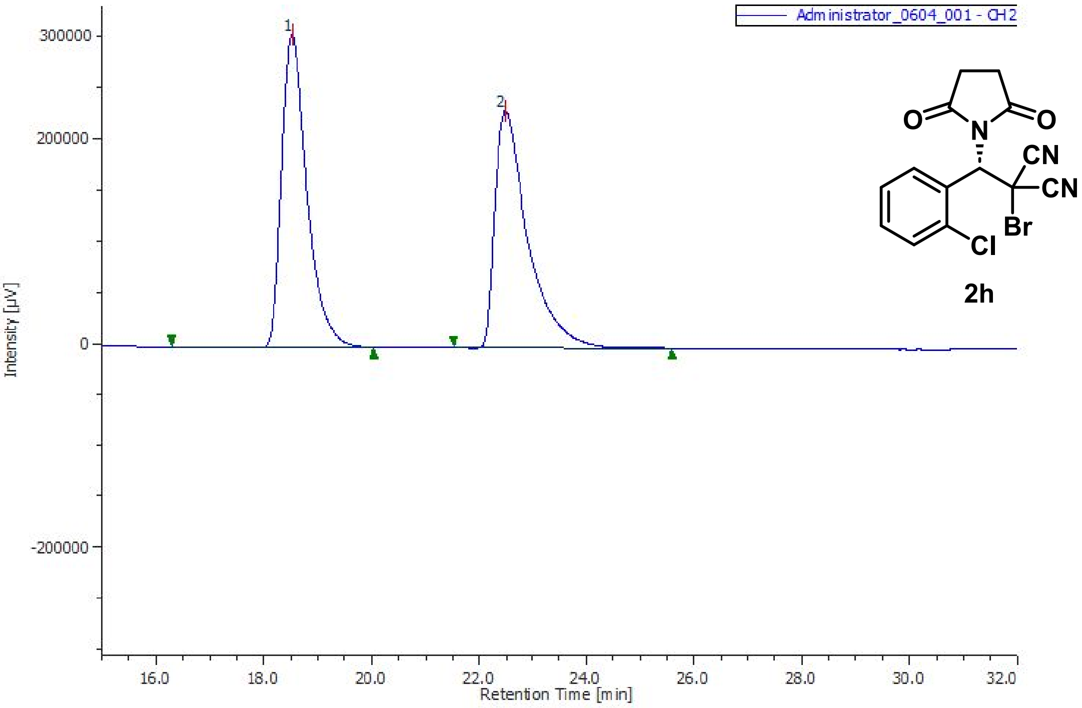


| | tR | Area (μV ・min) | Area (%) |
|---|---|---|---|
| 1 | 18.525 | 9884537 | 49.872 |
| 2 | 22.483 | 9935468 | 50.128 |

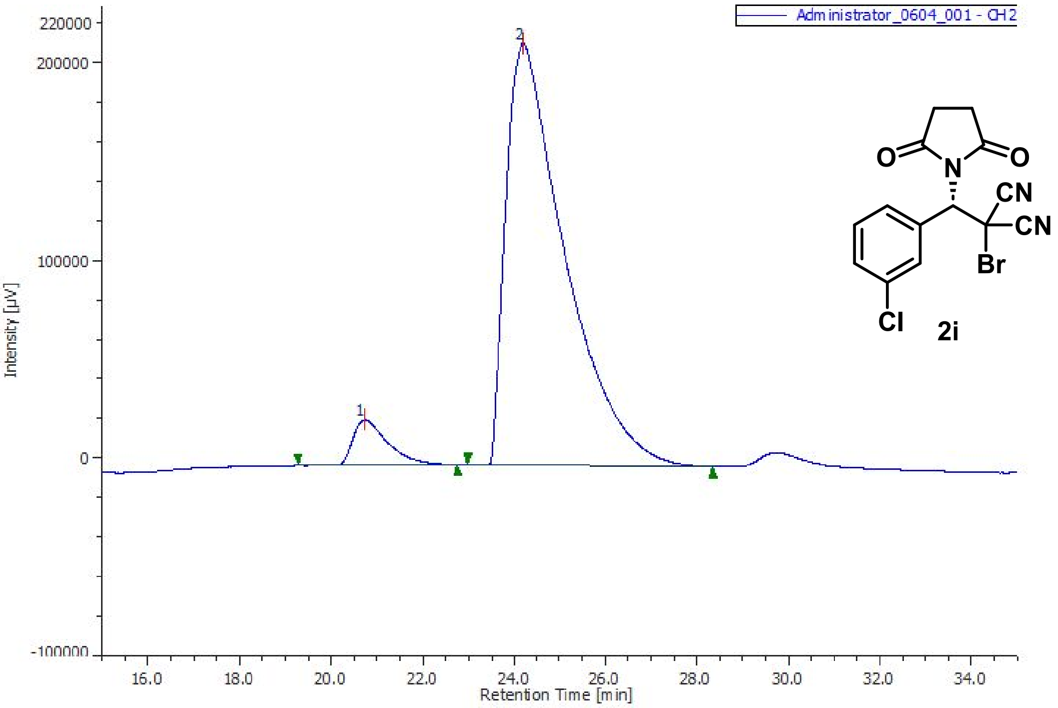


| | tR | Area (μV・min) | Area (%) |
|---|---|---|---|
| 1 | 20.733 | 1192758 | 5.759 |
| 2 | 24.208 | 19517666 | 94.241 |

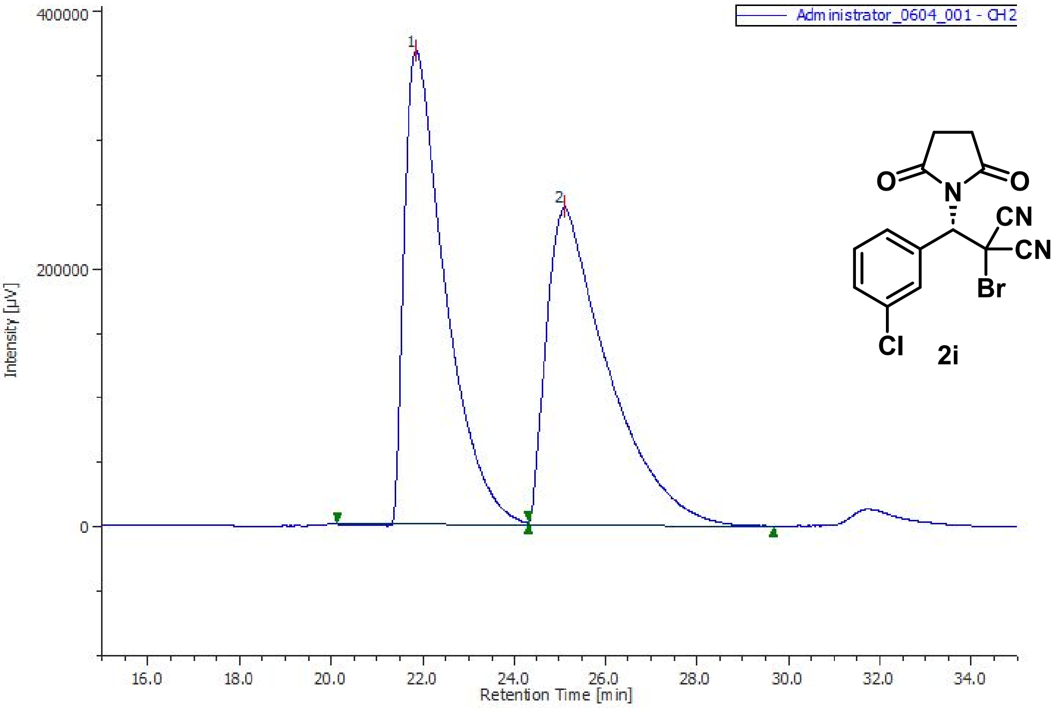


| | tR | Area (μV ・min) | Area (%) |
|---|---|---|---|
| 1 | 21.858 | 22789164 | 49.773 |
| 2 | 25.125 | 22996940 | 50.227 |

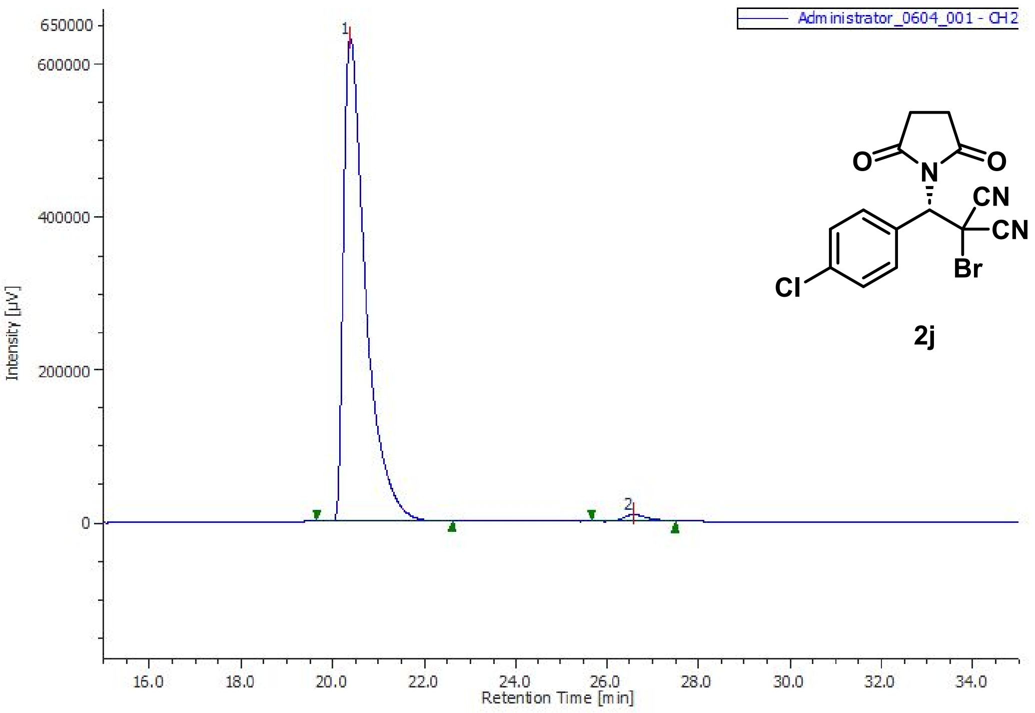


| | tR | Area (µV・min) | Area (%) |
|---|---|---|---|
| 1 | 20.392 | 21026964 | 98.653 |
| 2 | 26.592 | 27128 | 1.347 |

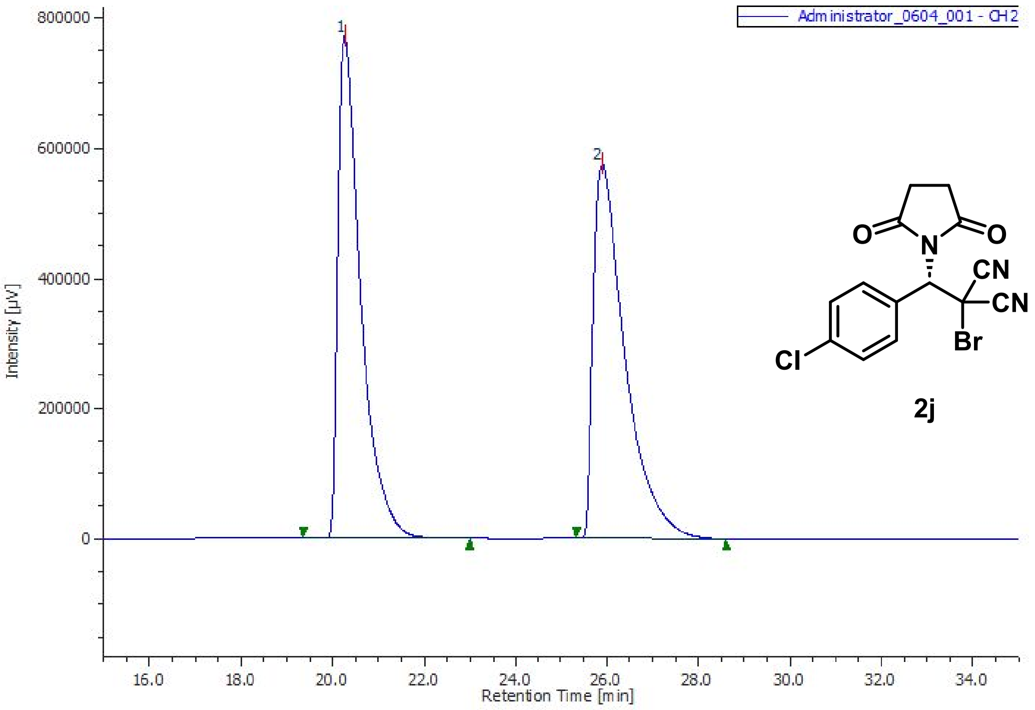


| | tR | Area (µV ・min) | Area (%) |
|---|---|---|---|
| 1 | 20.267 | 26754519 | 49.200 |
| 2 | 25.917 | 27624471 | 50.800 |

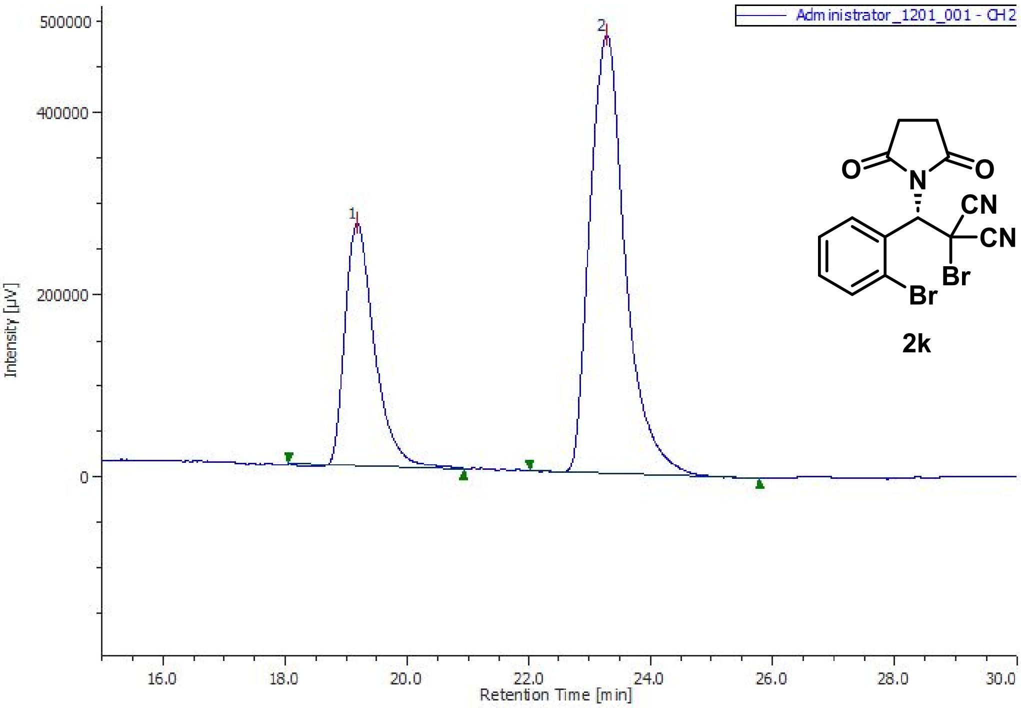


| | tR | Area (μV・min) | Area (%) |
|---|---|---|---|
| 1 | 19.175 | 8931418 | 31.490 |
| 2 | 23.292 | 19431709 | 68.510 |

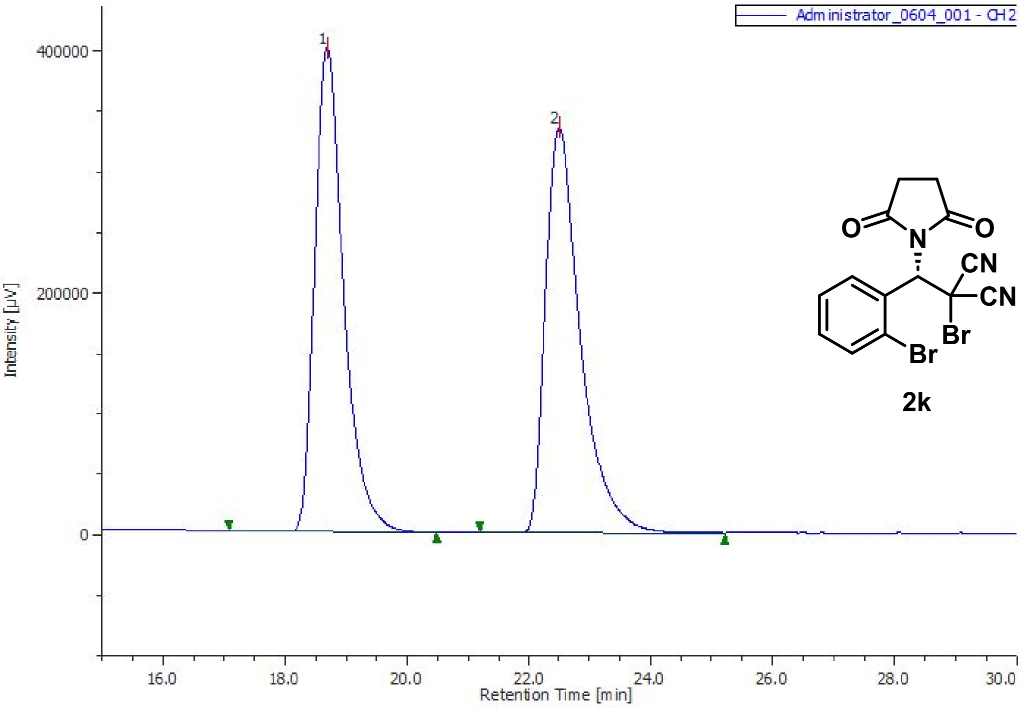


| | tR | Area (μV ・min) | Area (%) |
|---|---|---|---|
| 1 | 18.692 | 132114041 | 49.808 |
| 2 | 22.508 | 13315684 | 50.192 |

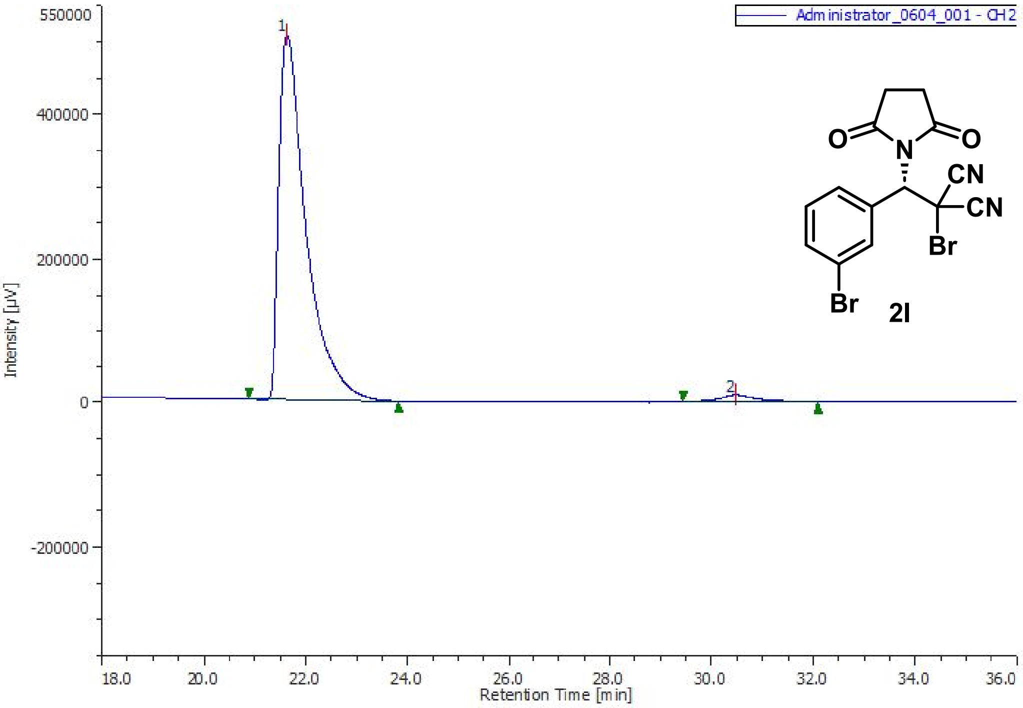


| | tR | Area (μV・min) | Area (%) |
|---|---|---|---|
| 1 | 21.633 | 18826286 | 97.472 |
| 2 | 30.475 | 488285 | 2.528 |

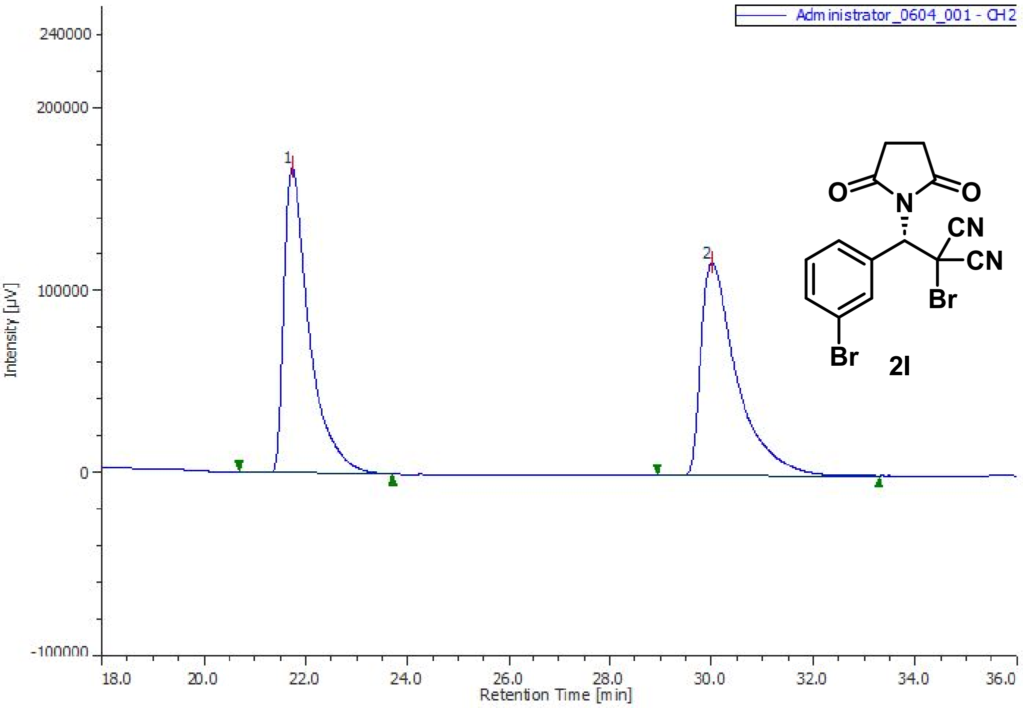


| | tR | Area (μV ・min) | Area (%) |
|---|---|---|---|
| 1 | 21.733 | 5736291 | 50.128 |
| 2 | 30.008 | 5706919 | 49.872 |

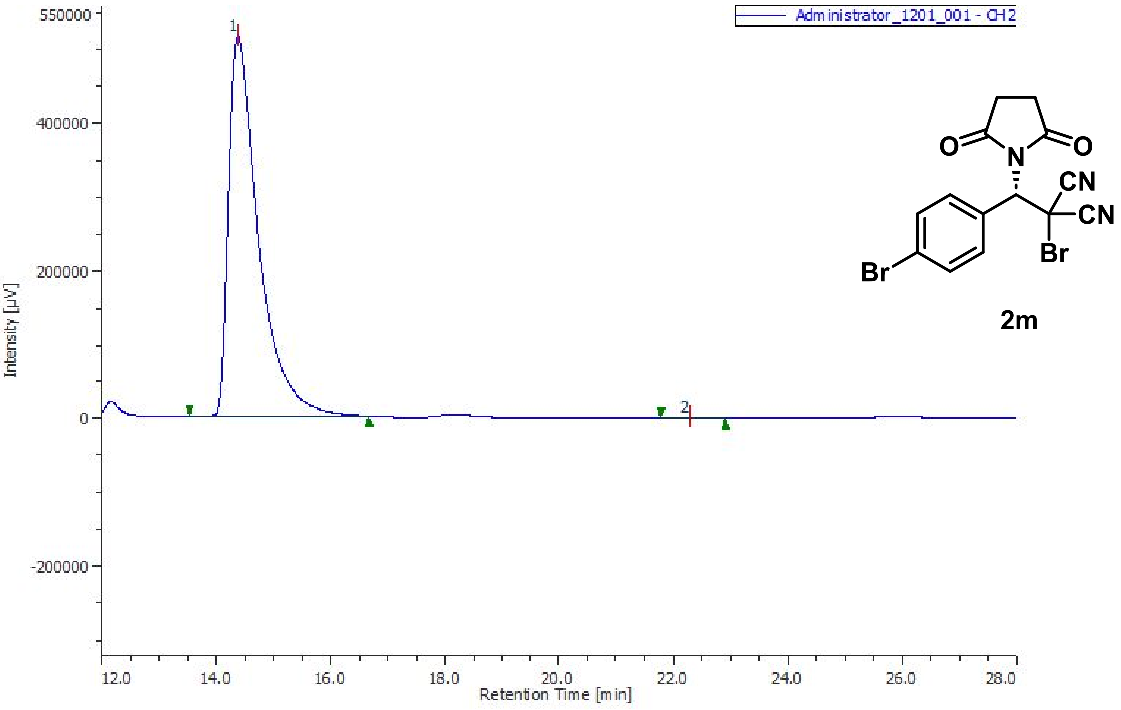


| | tR | Area (µV・min) | Area (%) |
|---|---|---|---|
| 1 | 14.383 | 18910732 | 99.959 |
| 2 | 22.300 | 7804 | 0.041 |

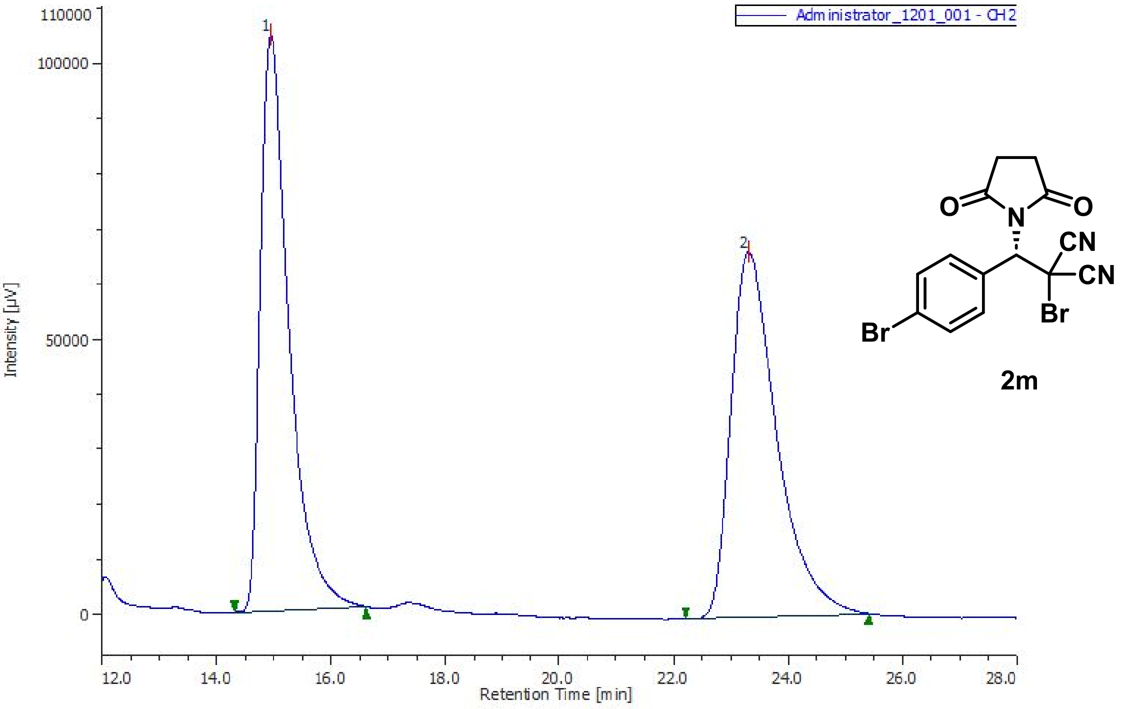


| | tR | Area (µV ・min) | Area (%) |
|---|---|---|---|
| 1 | 14.950 | 3631387 | 49.640 |
| 2 | 23.317 | 3684070 | 50.360 |

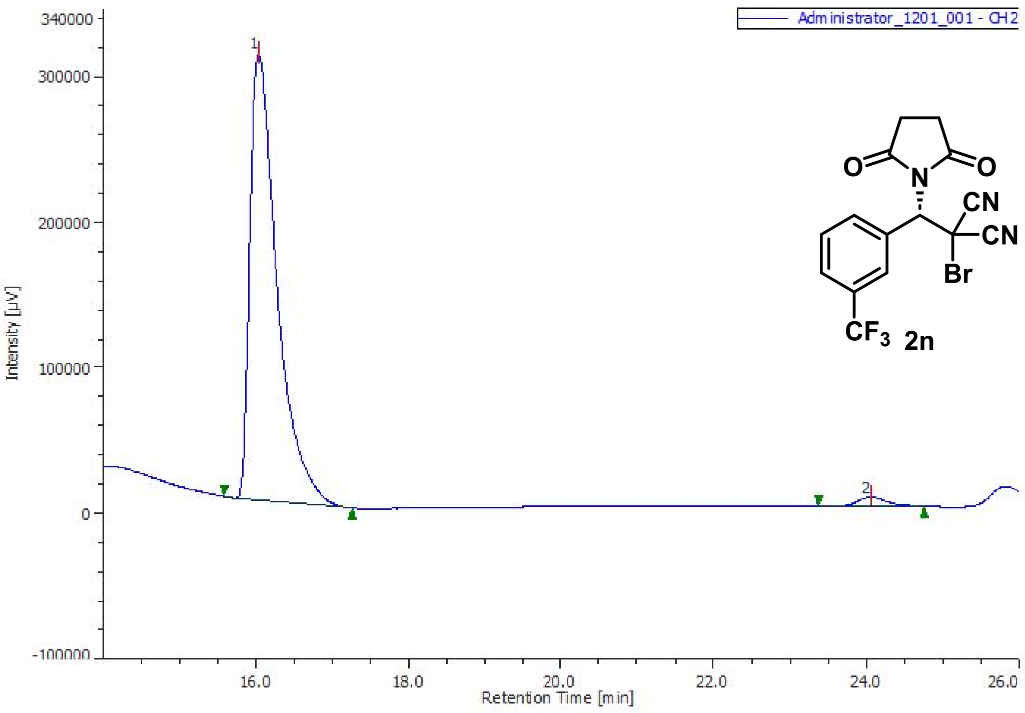


| | tR | Area (μV・min) | Area (%) |
|---|---|---|---|
| 1 | 16.033 | 7568482 | 97.908 |
| 2 | 24.058 | 161699 | 2.092 |

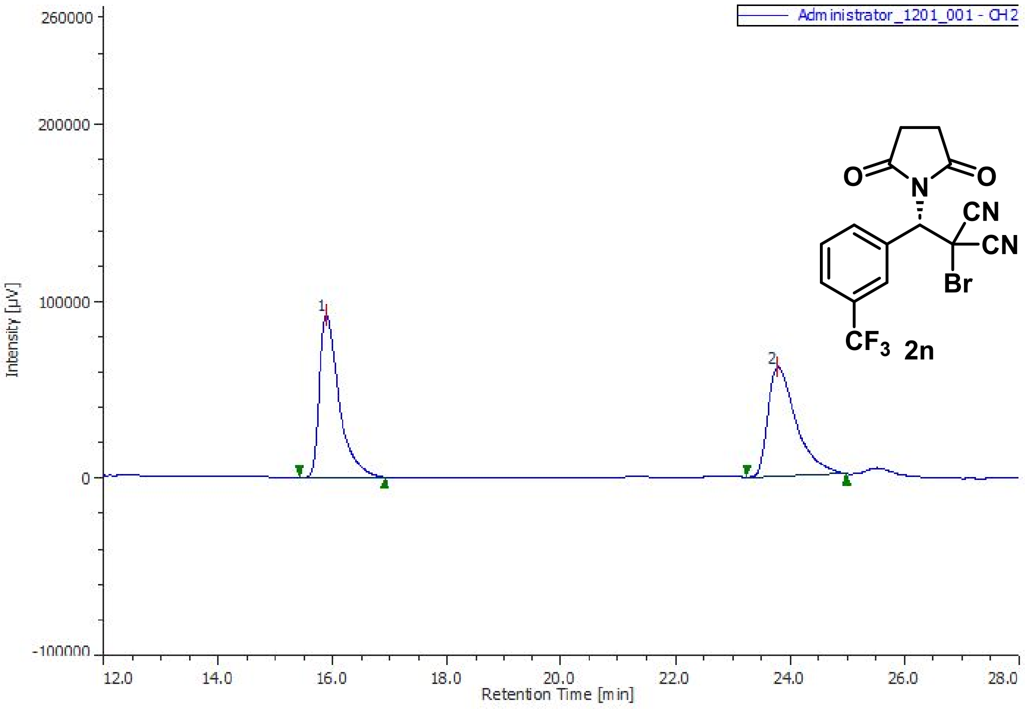


| | tR | Area (μV ・min) | Area (%) |
|---|---|---|---|
| 1 | 15.892 | 2179695 | 50.950 |
| 2 | 23.792 | 2098410 | 49.050 |

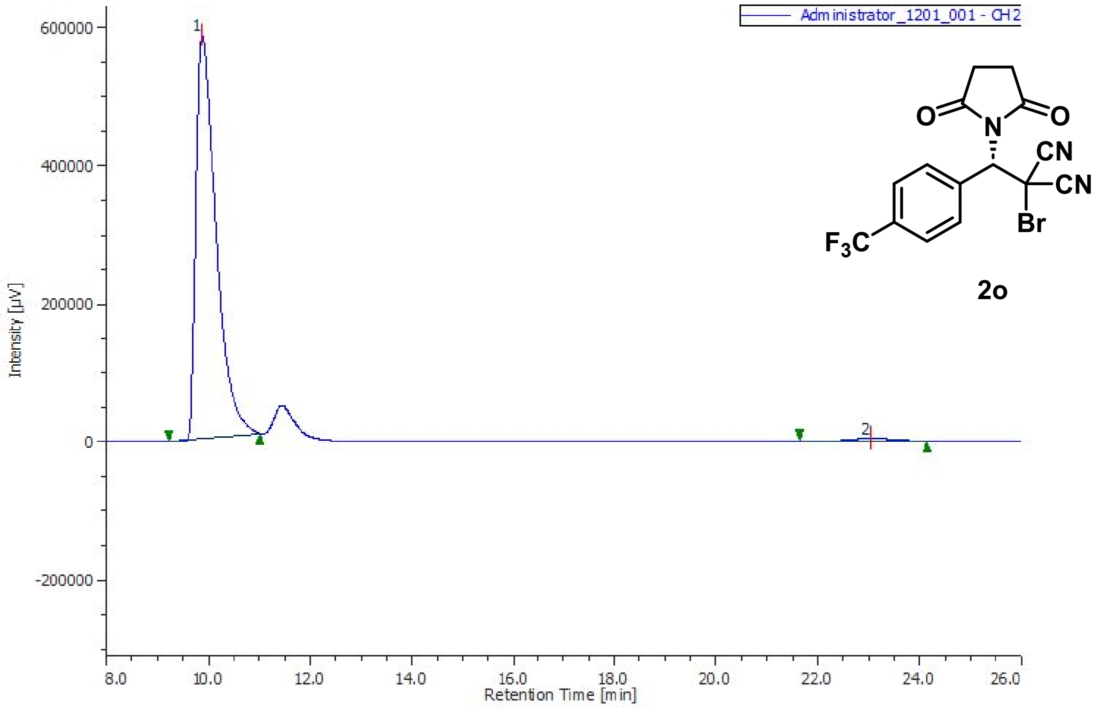


| | tR | Area (μV・min) | Area (%) |
|---|---|---|---|
| 1 | 9.883 | 15929963 | 98.875 |
| 2 | 23.033 | 181249 | 1.125 |

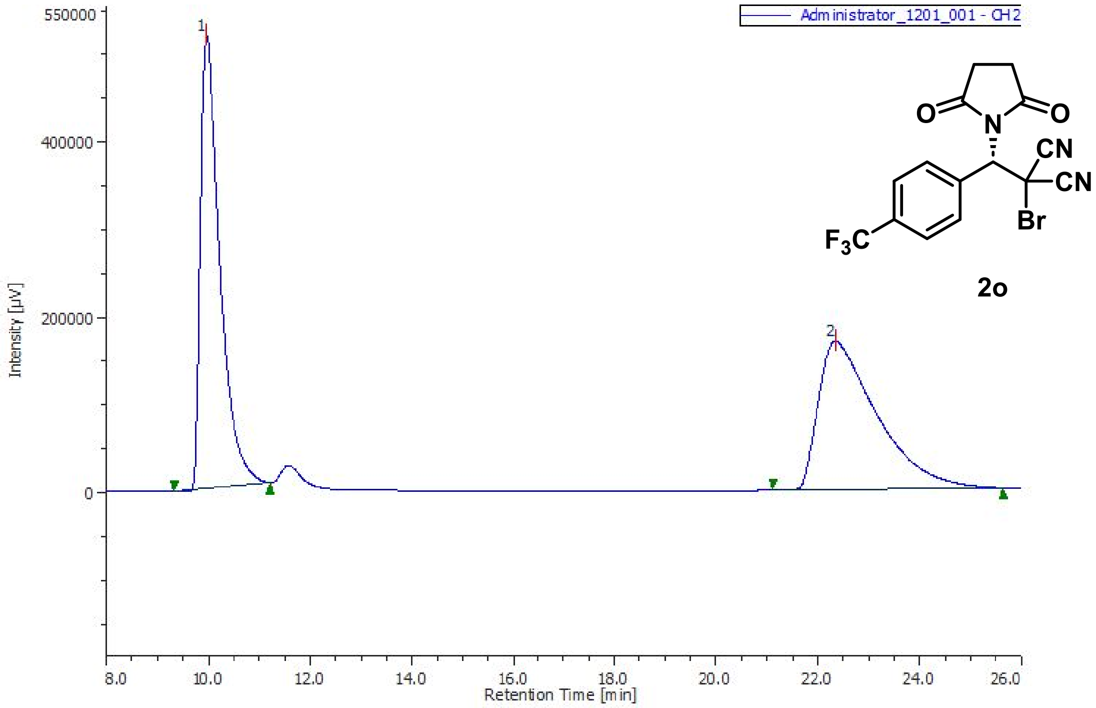


| | tR | Area (μV ・min) | Area (%) |
|---|---|---|---|
| 1 | 9.967 | 13719445 | 50.475 |
| 2 | 22.342 | 13461041 | 49.525 |

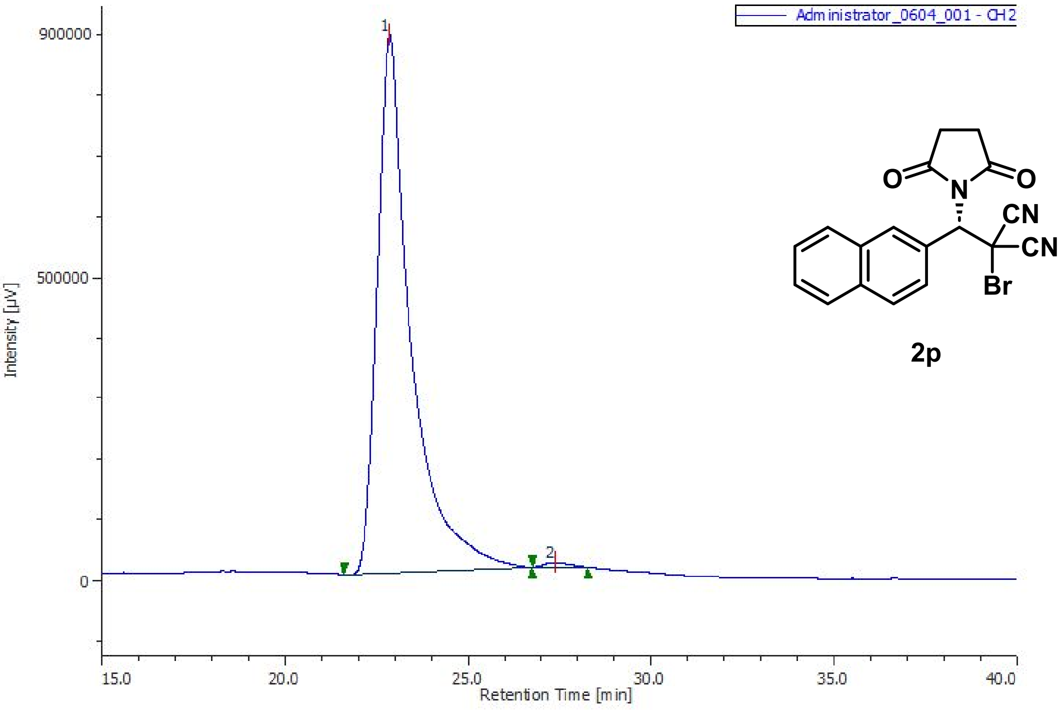


| | tR | Area (µV・min) | Area (%) |
|---|---|---|---|
| 1 | 22.858 | 56555129 | 99.362 |
| 2 | 27.392 | 363349 | 0.638 |

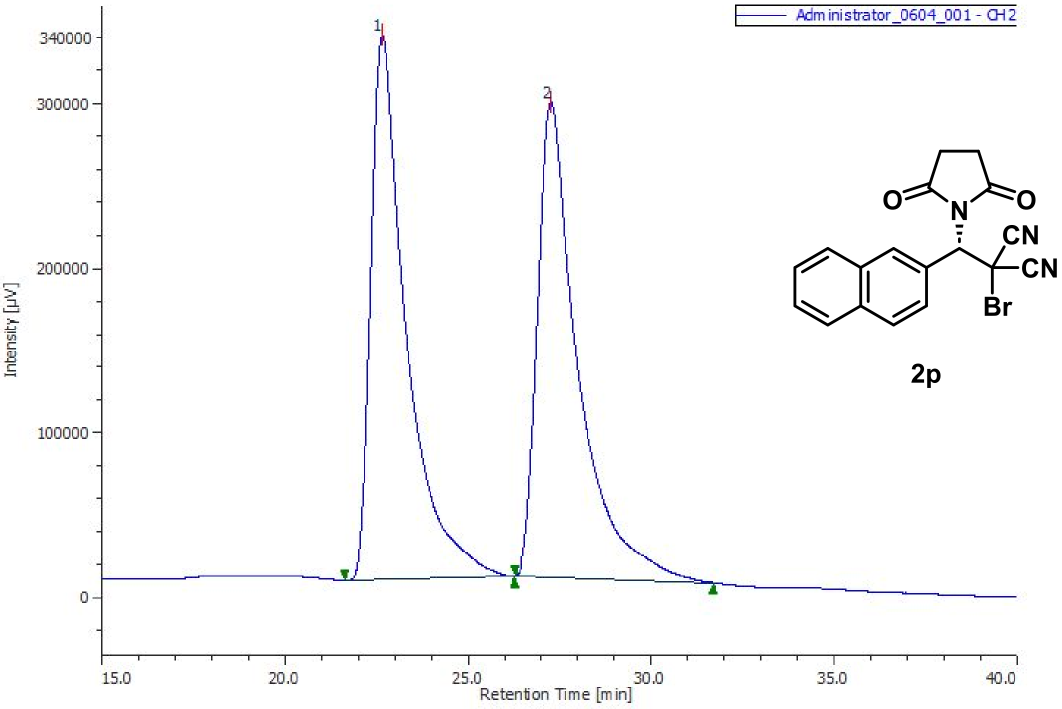


| | tR | Area (µV ・min) | Area (%) |
|---|---|---|---|
| 1 | 22.642 | 22200661 | 50.052 |
| 2 | 27.292 | 22154397 | 49.948 |

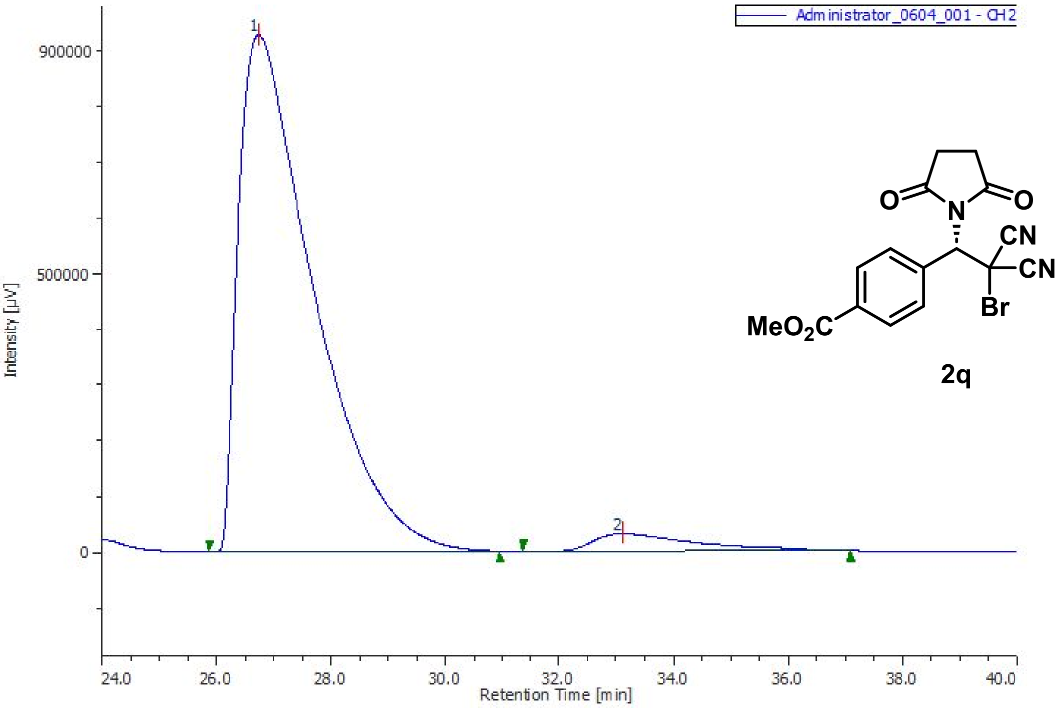


| | tR | Area (µV・min) | Area (%) |
|---|---|---|---|
| 1 | 26.733 | 84499246 | 95.659 |
| 2 | 33.117 | 3834942 | 4.341 |

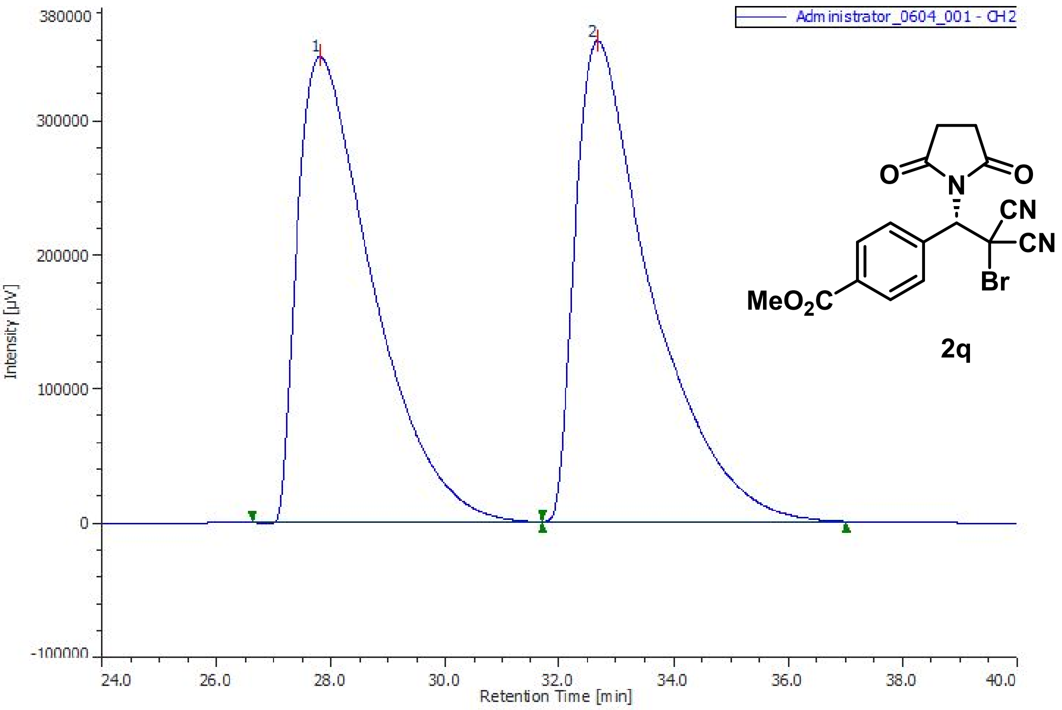


| | tR | Area (µV ・min) | Area (%) |
|---|---|---|---|
| 1 | 27.808 | 22200661 | 50.052 |
| 2 | 32.675 | 22154397 | 49.948 |

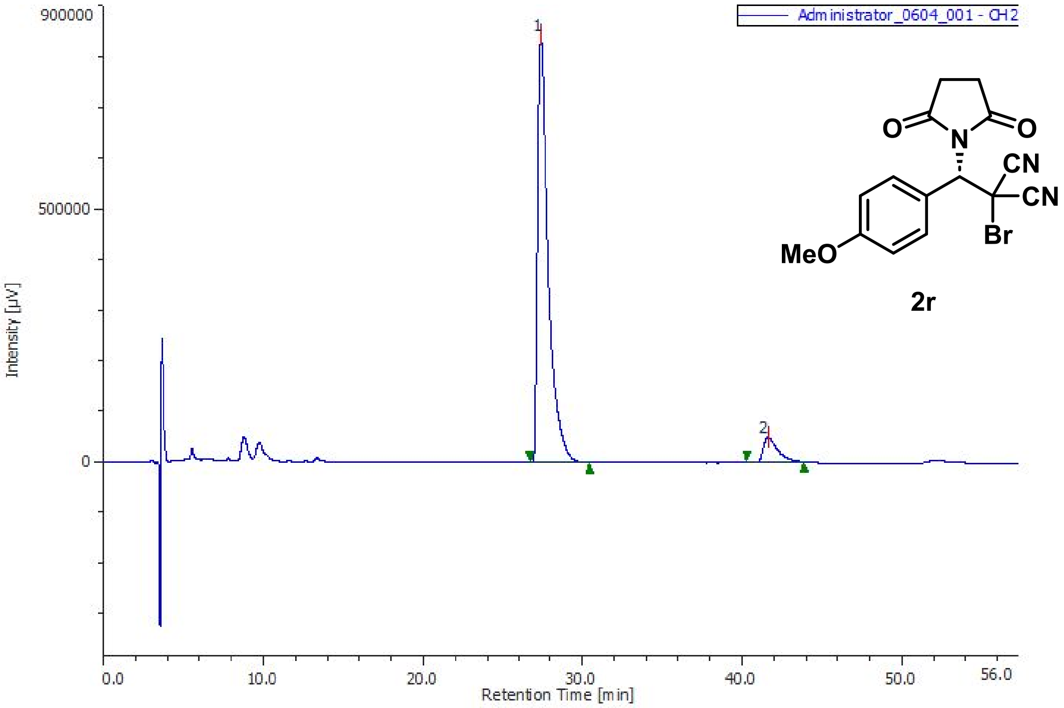


| | tR | Area (μV・min) | Area (%) |
|---|---|---|---|
| 1 | 27.433 | 39081644 | 92.702 |
| 2 | 41.642 | 3076739 | 7.298 |

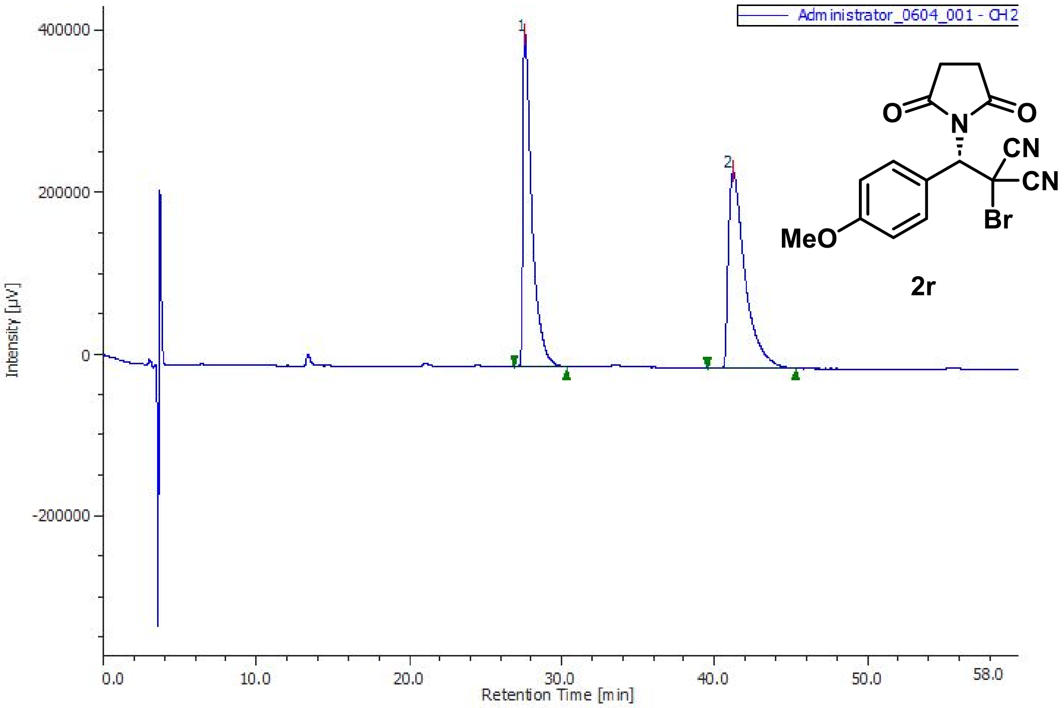


| | tR | Area (μV ・min) | Area (%) |
|---|---|---|---|
| 1 | 27.650 | 18324501 | 50.003 |
| 2 | 41.233 | 18322052 | 49.997 |

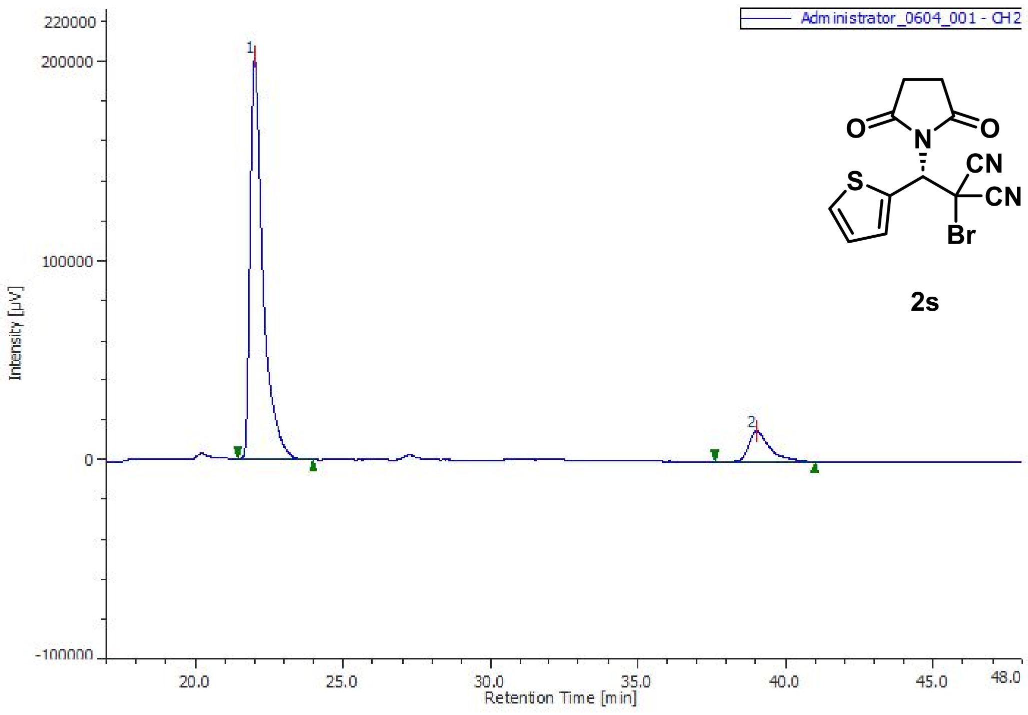


| | tR | Area (µV・min) | Area (%) |
|---|---|---|---|
| 1 | 22.008 | 6080170 | 92.974 |
| 2 | 39.033 | 15206 | 7.026 |

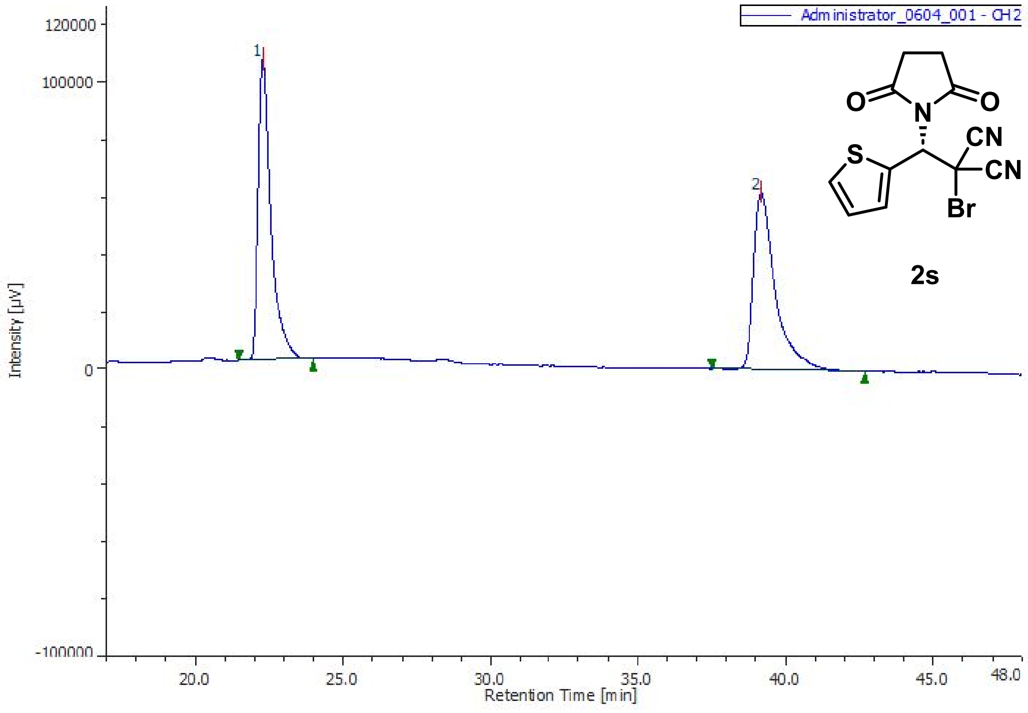


| | tR | Area (µV ・min) | Area (%) |
|---|---|---|---|
| 1 | 22.283 | 3171428 | 49.894 |
| 2 | 39.183 | 3184937 | 50.106 |

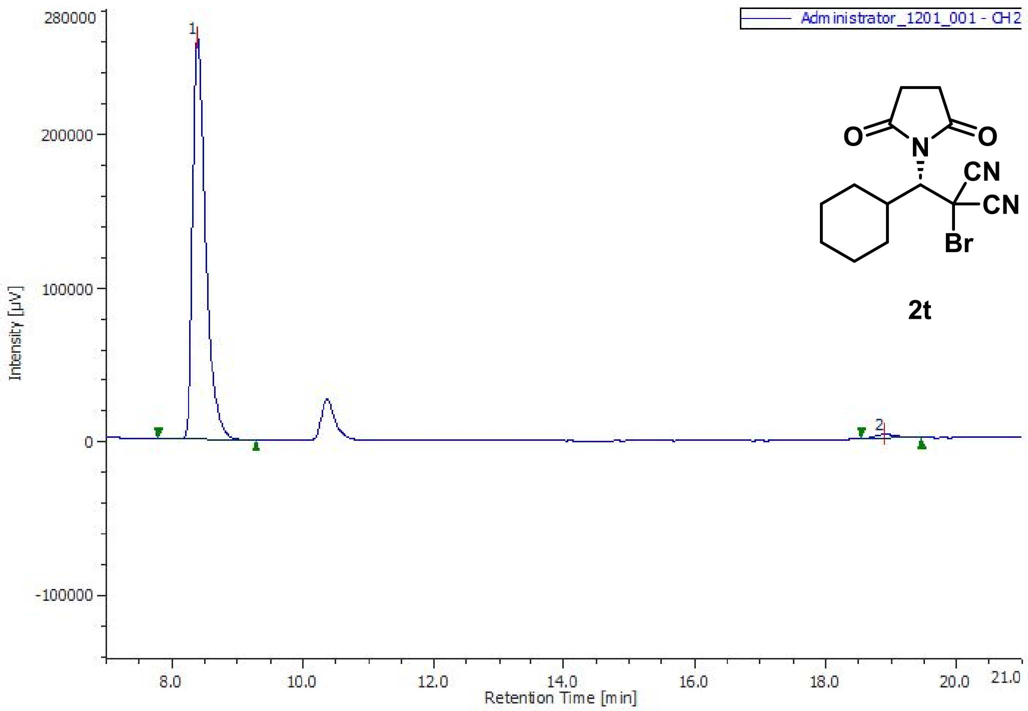


| | tR | Area (μV・min) | Area (%) |
|---|---|---|---|
| 1 | 8.392 | 3575541 | 98.327 |
| 2 | 18.900 | 60830 | 1.673 |

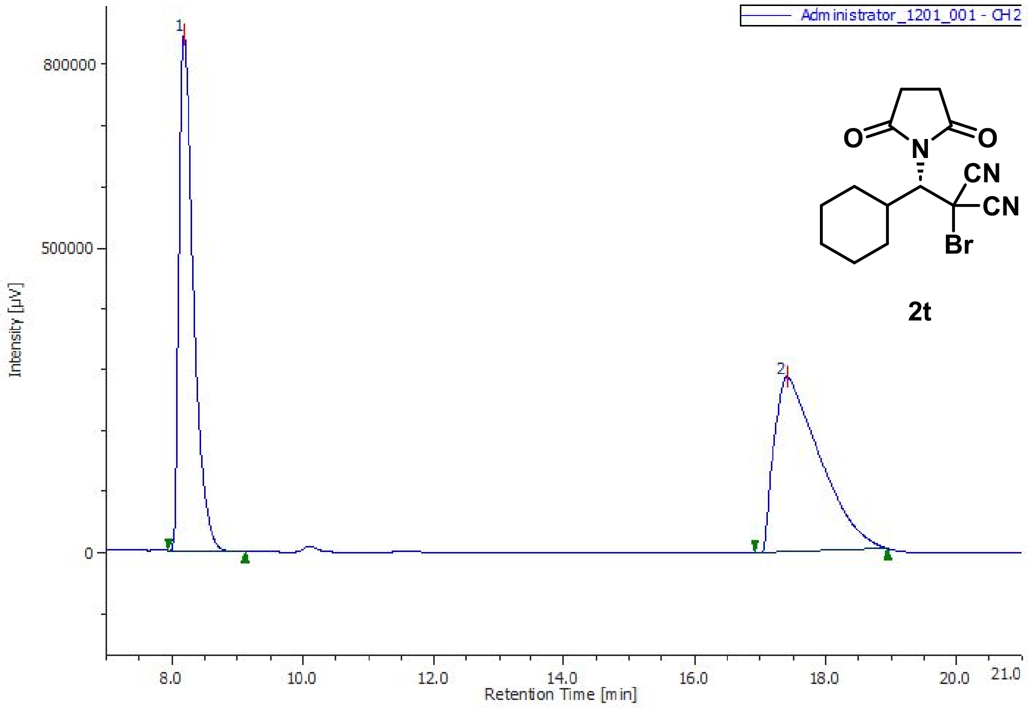


| | tR | Area (μV ・min) | Area (%) |
|---|---|---|---|
| 1 | 8.183 | 13075494 | 48.551 |
| 2 | 17.408 | 13855788 | 51.449 |

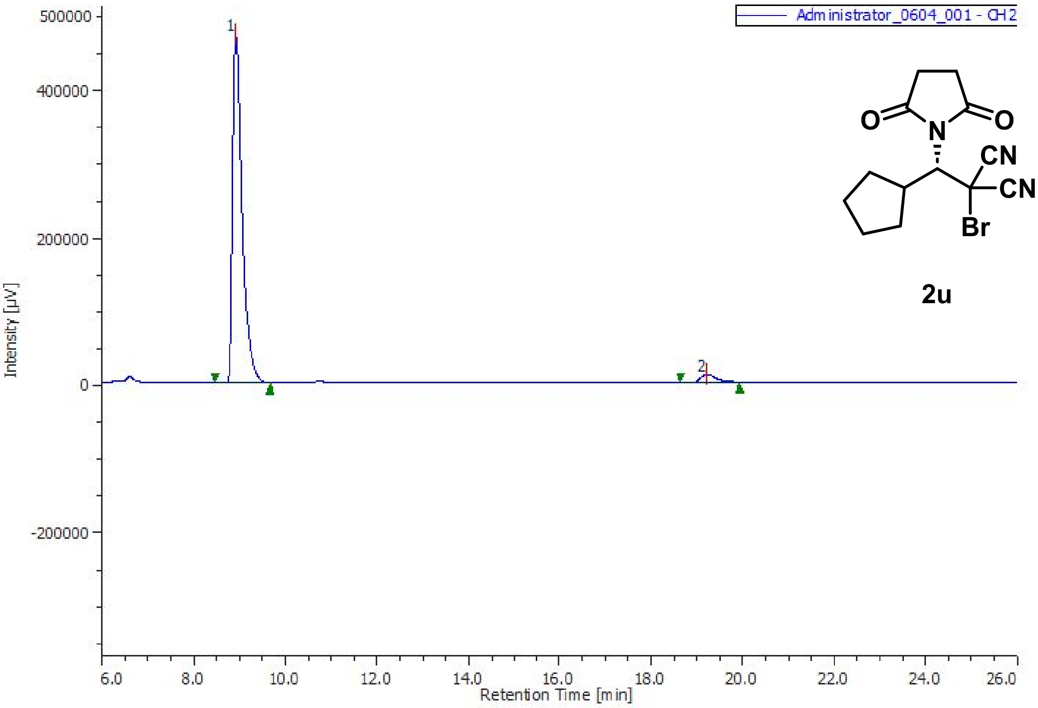


| | tR | Area (µV・min) | Area (%) |
|---|---|---|---|
| 1 | 8.925 | 6830701 | 96.267 |
| 2 | 19.233 | 264889 | 3.733 |

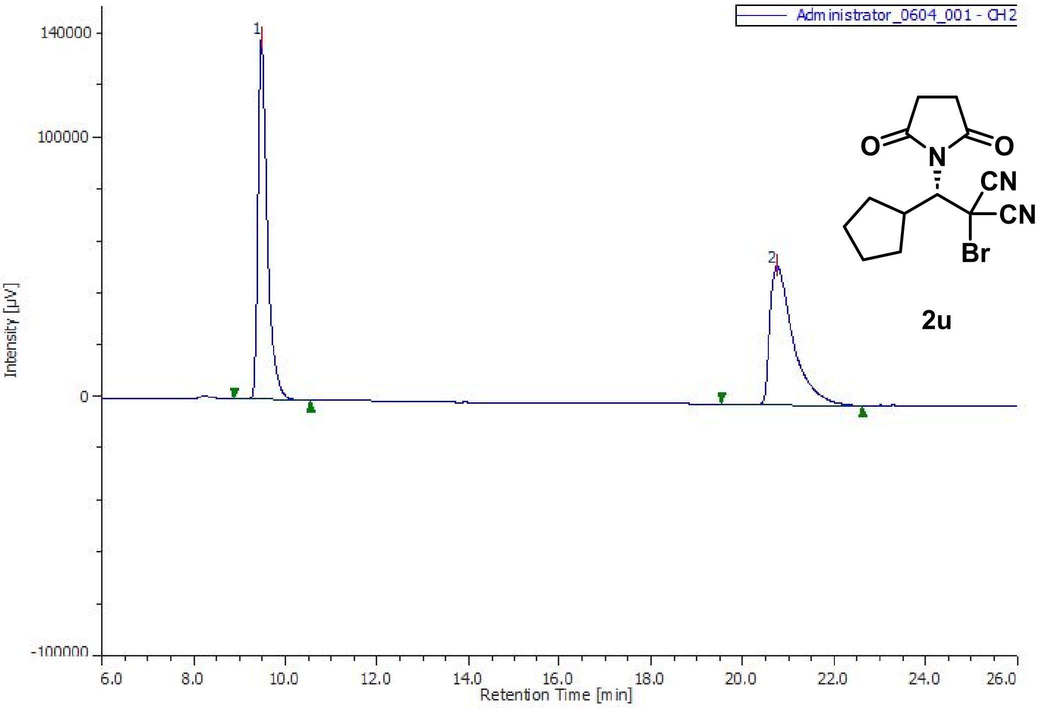


| | tR | Area (µV ・min) | Area (%) |
|---|---|---|---|
| 1 | 9.475 | 1945635 | 50.665 |
| 2 | 20.758 | 1894576 | 49.335 |

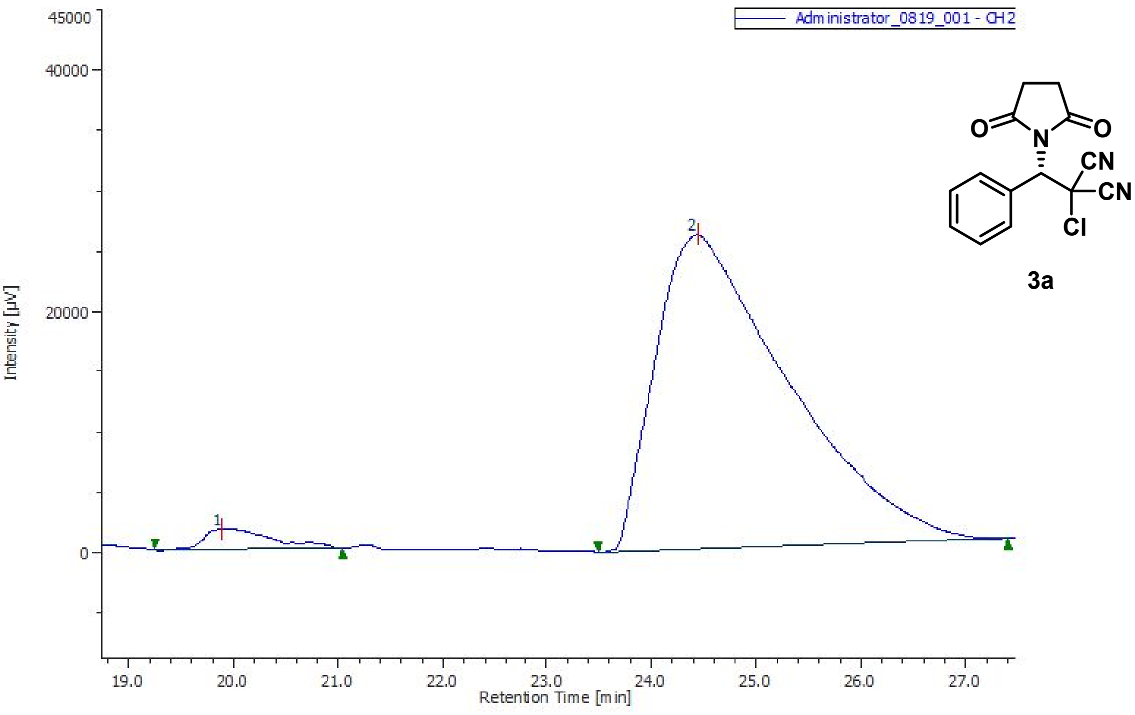


| | tR | Area (µV・min) | Area (%) |
|---|---|---|---|
| 1 | 19.883 | 74924 | 3.163 |
| 2 | 24.442 | 2293643 | 96.837 |

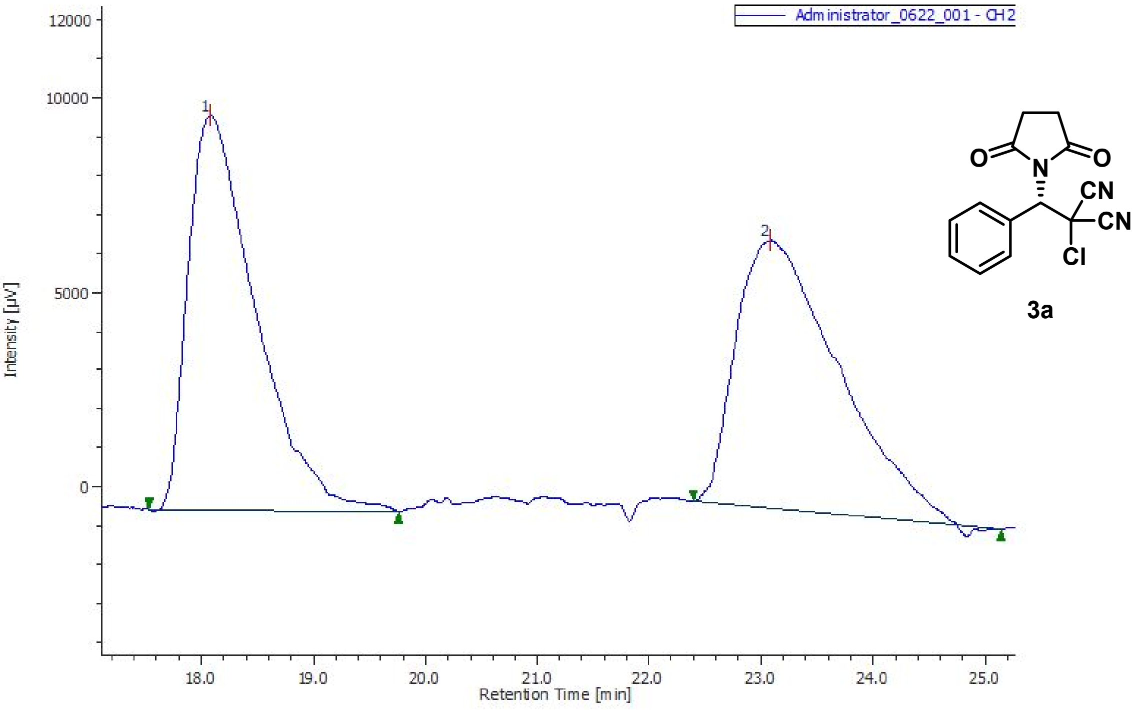


| | tR | Area (µV ・min) | Area (%) |
|---|---|---|---|
| 1 | 18.083 | 425411 | 49.17 |
| 2 | 23.083 | 439767 | 50.83 |

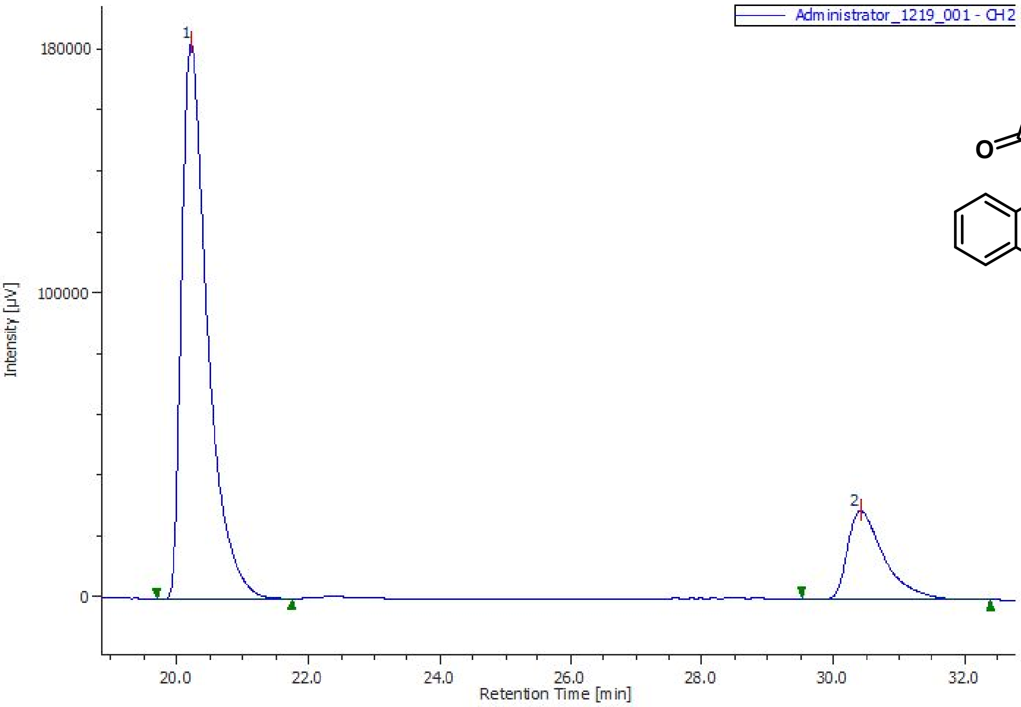


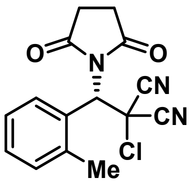


**3b**

| | tR | Area (μV ・min) | Area (%) |
|---|---|---|---|
| 1 | 20.217 | 5002799 | 81.924 |
| 2 | 30.408 | 1103821 | 18.076 |

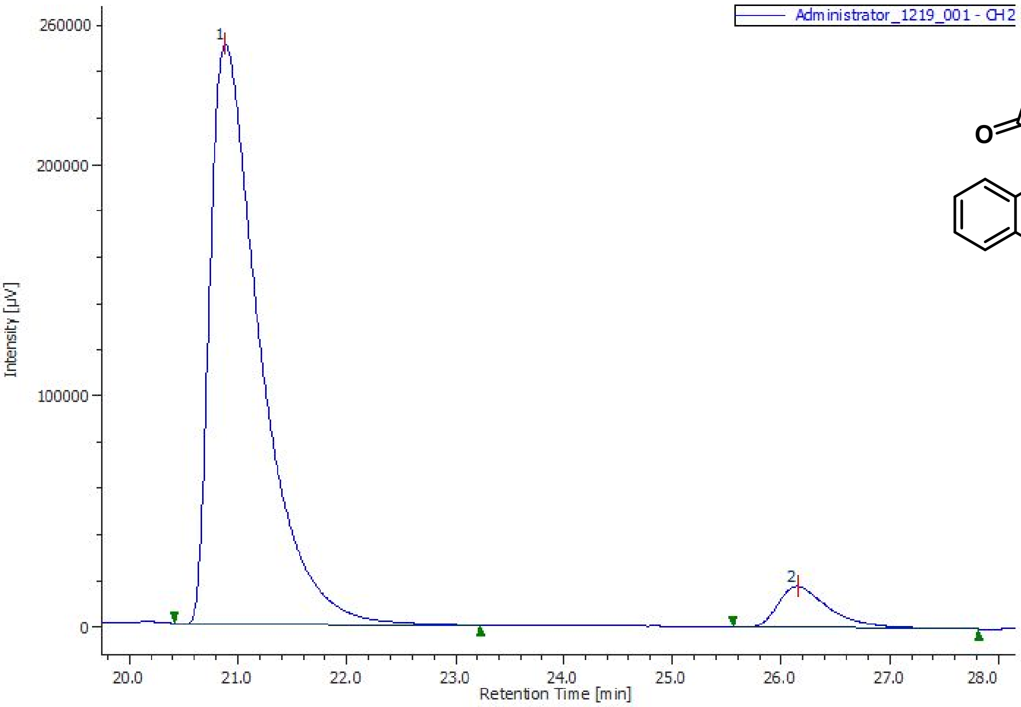


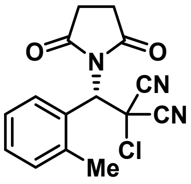


**3b**

| | tR | Area (μV ・min) | Area (%) |
|---|---|---|---|
| 1 | 19.925 | 8398685 | 50.029 |
| 2 | 29.55 | 8388844 | 49.971 |

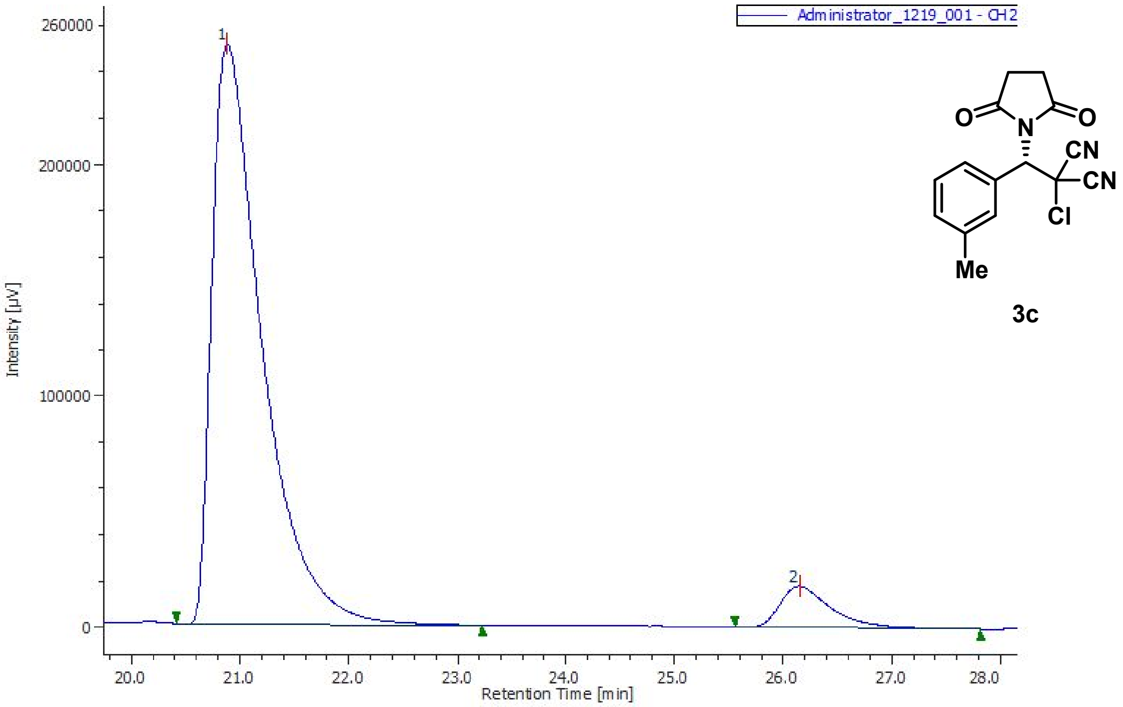


| | tR | Area (μV ・min) | Area (%) |
|---|---|---|---|
| 1 | 20.883 | 7989301 | 93.189 |
| 2 | 26.15 | 583917 | 6.811 |

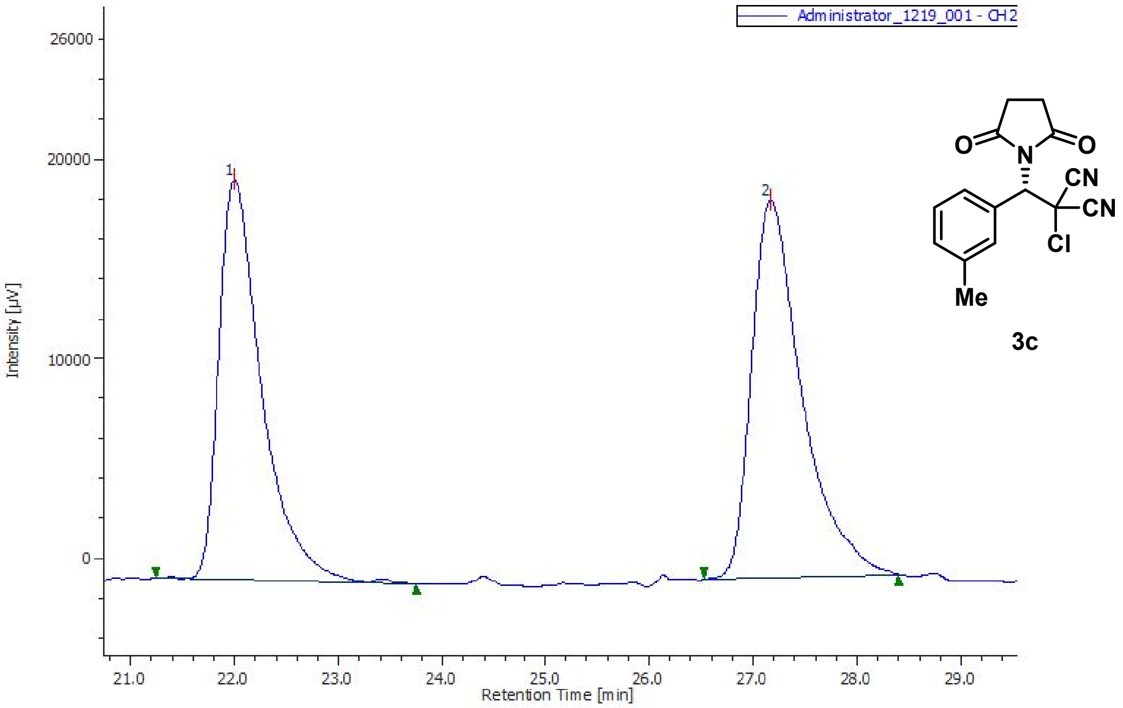


| | tR | Area (μV ・min) | Area (%) |
|---|---|---|---|
| 1 | 22.000 | 575083 | 47.533 |
| 2 | 27.167 | 634774 | 52.467 |

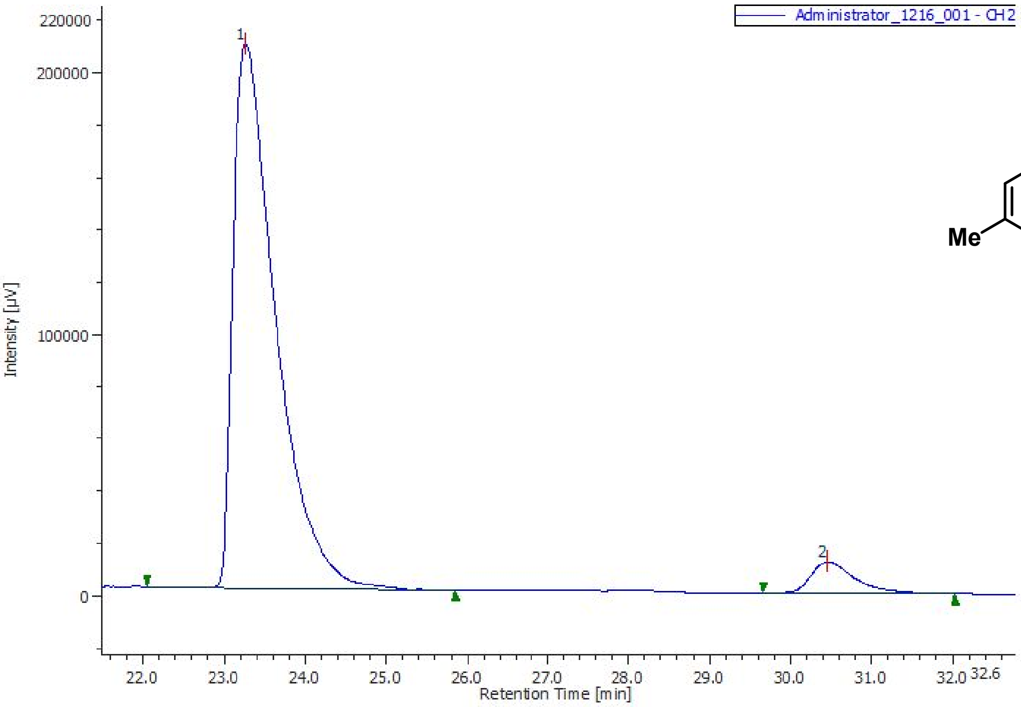


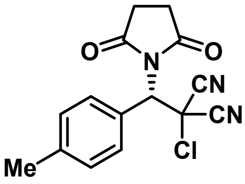


3d

| | tR | Area (μV ・min) | Area (%) |
|---|---|---|---|
| 1 | 23.267 | 7586989 | 94.39 |
| 2 | 30.458 | 450934 | 5.61 |

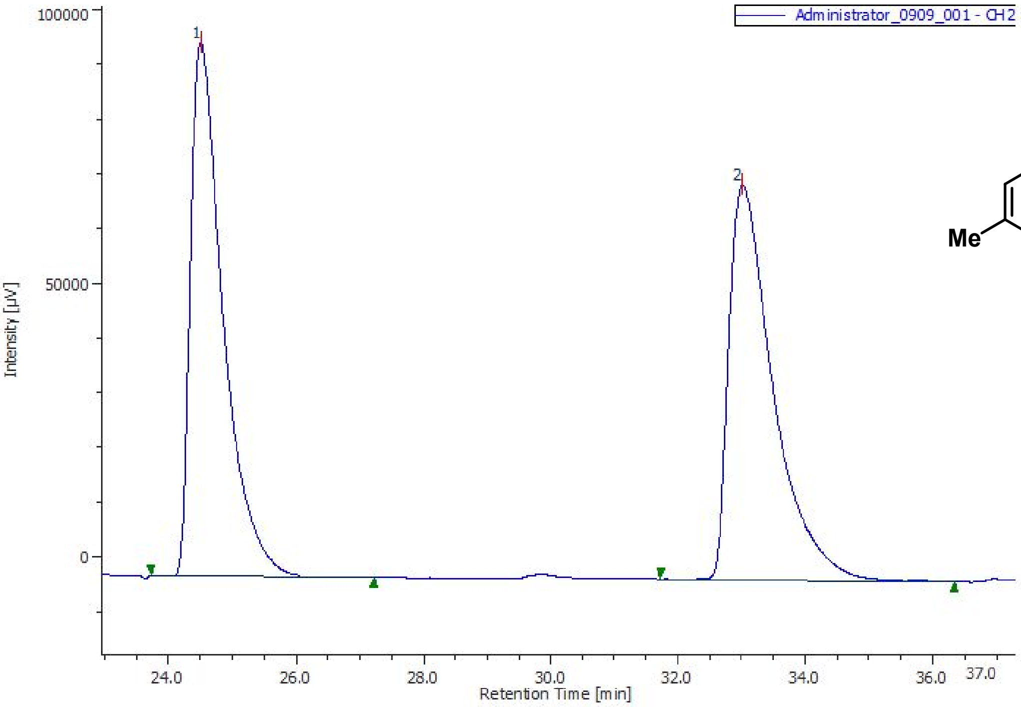


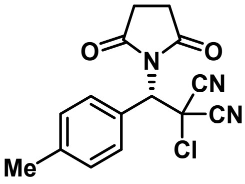


3d

| | tR | Area (μV ・min) | Area (%) |
|---|---|---|---|
| 1 | 24.517 | 3453550 | 49.891 |
| 2 | 33.017 | 3468577 | 50.109 |

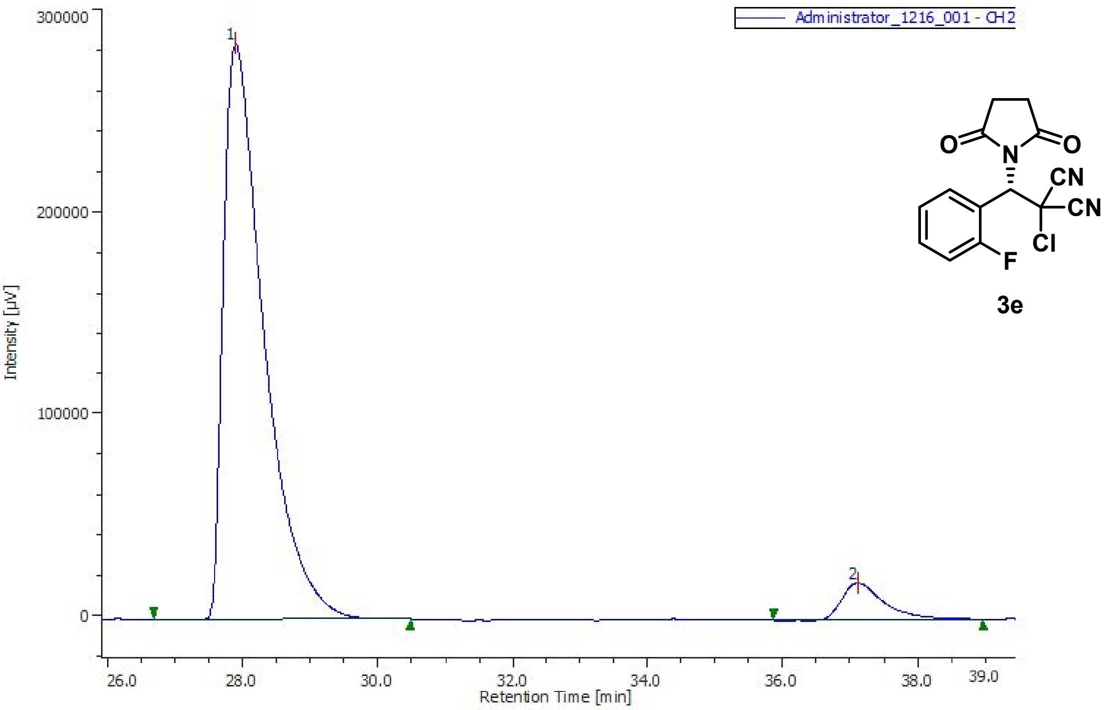


| | tR | Area (µV ・min) | Area (%) |
|---|---|---|---|
| 1 | 27.900 | 11872975 | 93.873 |
| 2 | 37.117 | 774904 | 6.127 |

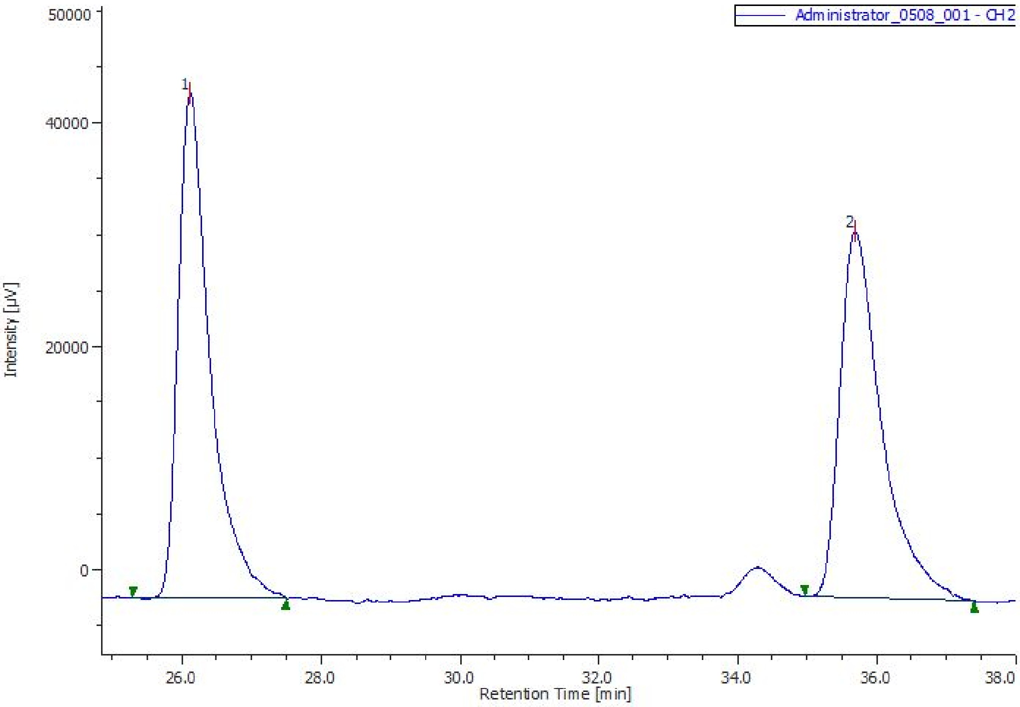


| | tR | Area (µV ・min) | Area (%) |
|---|---|---|---|
| 1 | 26.117 | 1440600 | 51.344 |
| 2 | 35.692 | 1365158 | 48.656 |

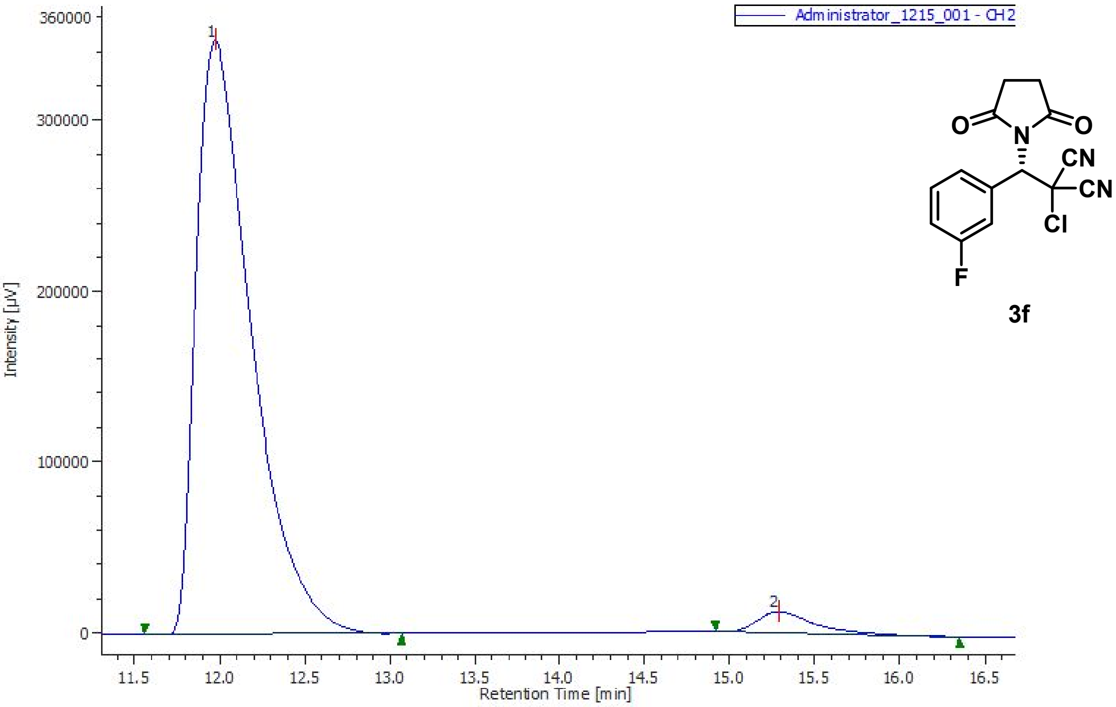


| | tR | Area (µV ・min) | Area (%) |
|---|---|---|---|
| 1 | 11.975 | 7814677 | 96.379 |
| 2 | 15.292 | 293581 | 3.621 |

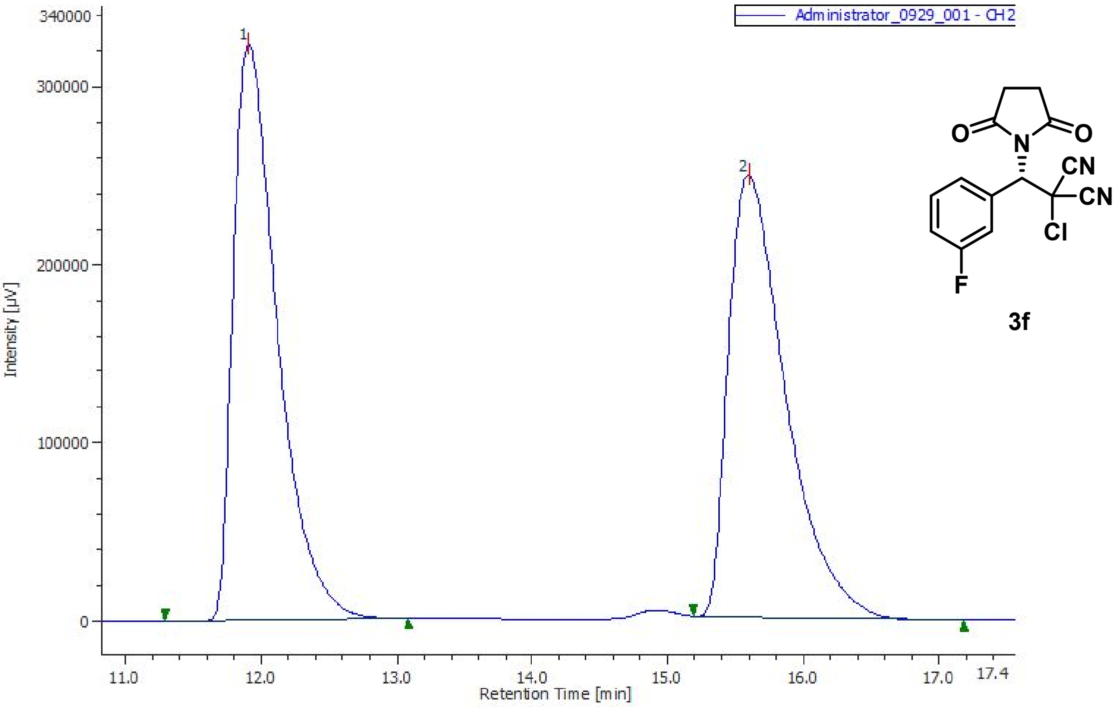


| | tR | Area (µV ・min) | Area (%) |
|---|---|---|---|
| 1 | 11.908 | 7288964 | 50.19 |
| 2 | 15.6 | 7233881 | 49.81 |

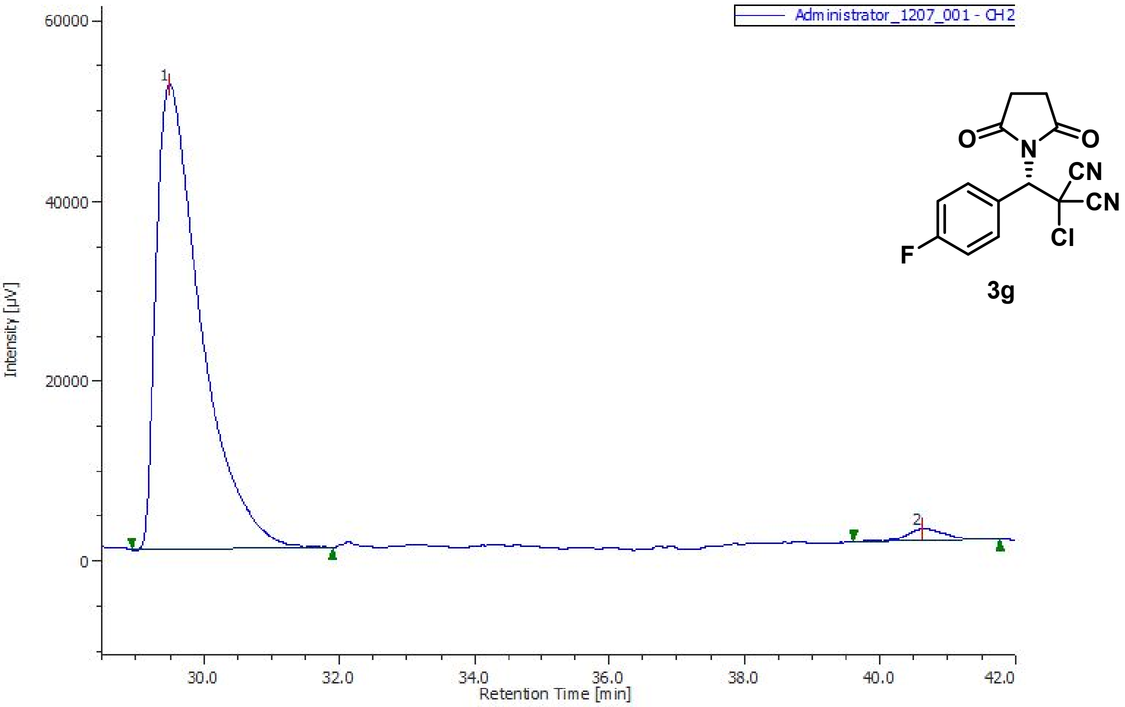


| | tR | Area (µV ・min) | Area (%) |
|---|---|---|---|
| 1 | 29.492 | 2363715 | 97.919 |
| 2 | 40.625 | 1279 | 2.081 |

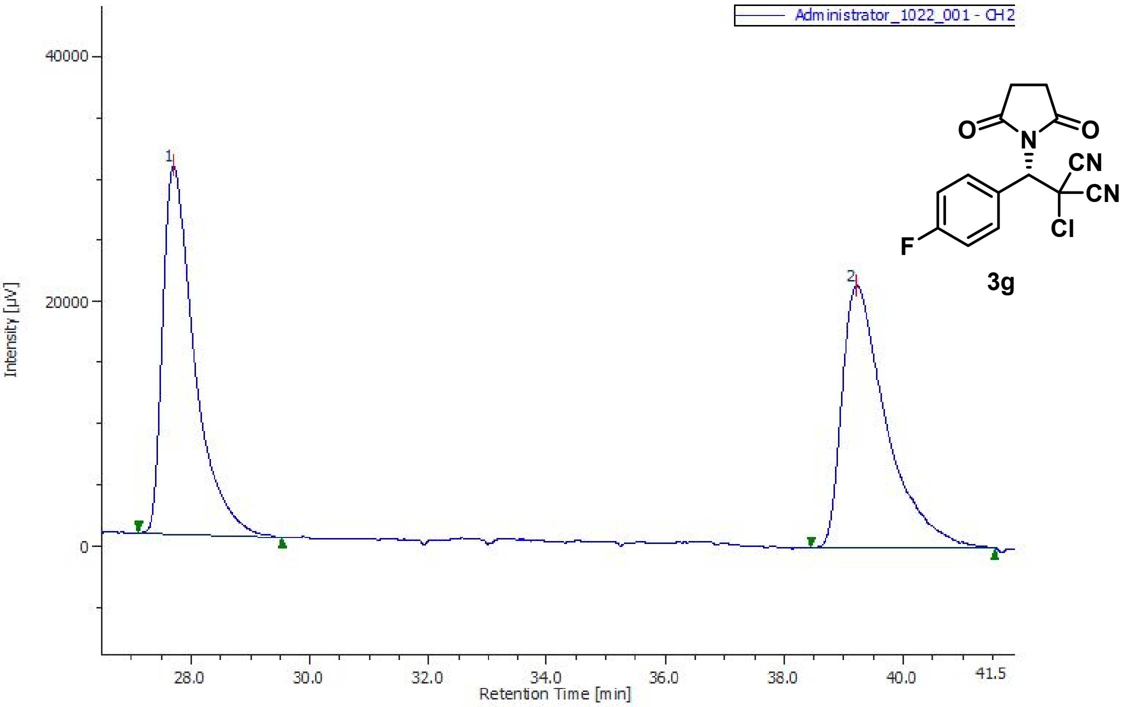


| | tR | Area (µV ・min) | Area (%) |
|---|---|---|---|
| 1 | 27.708 | 1113362 | 50.036 |
| 2 | 39.217 | 1111741 | 49.964 |

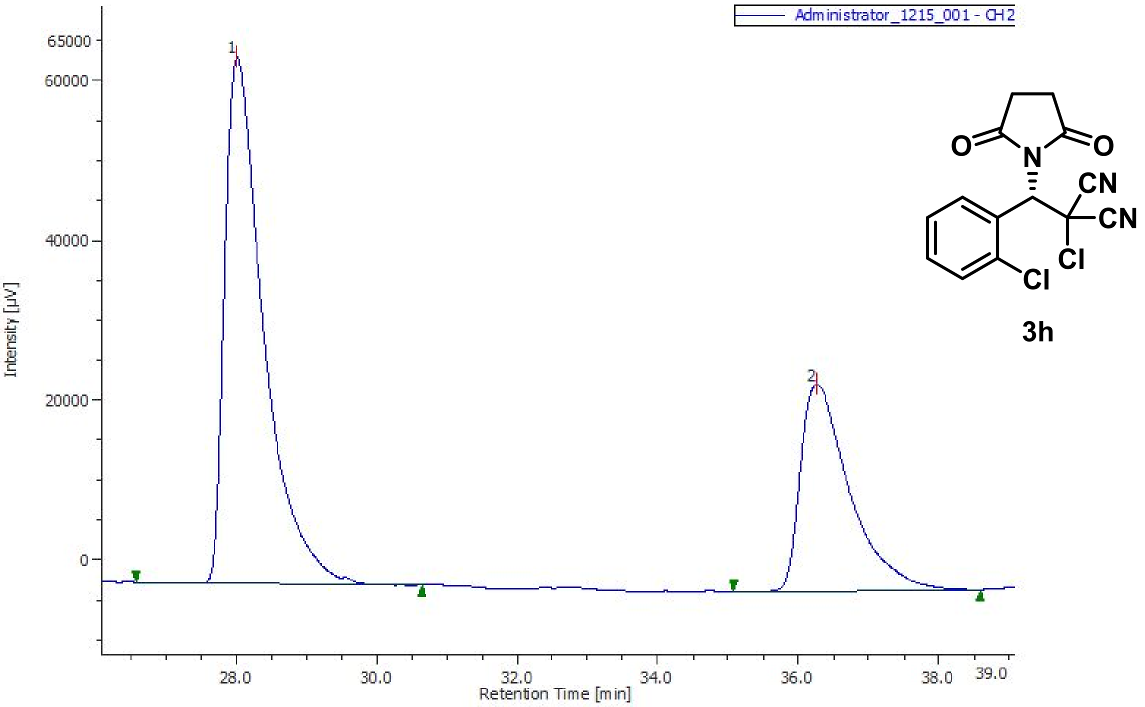


3h

| | tR | Area (μV ・min) | Area (%) |
|---|---|---|---|
| 1 | 28.000 | 2535079 | 66.993 |
| 2 | 36.258 | 1249043 | 33.007 |

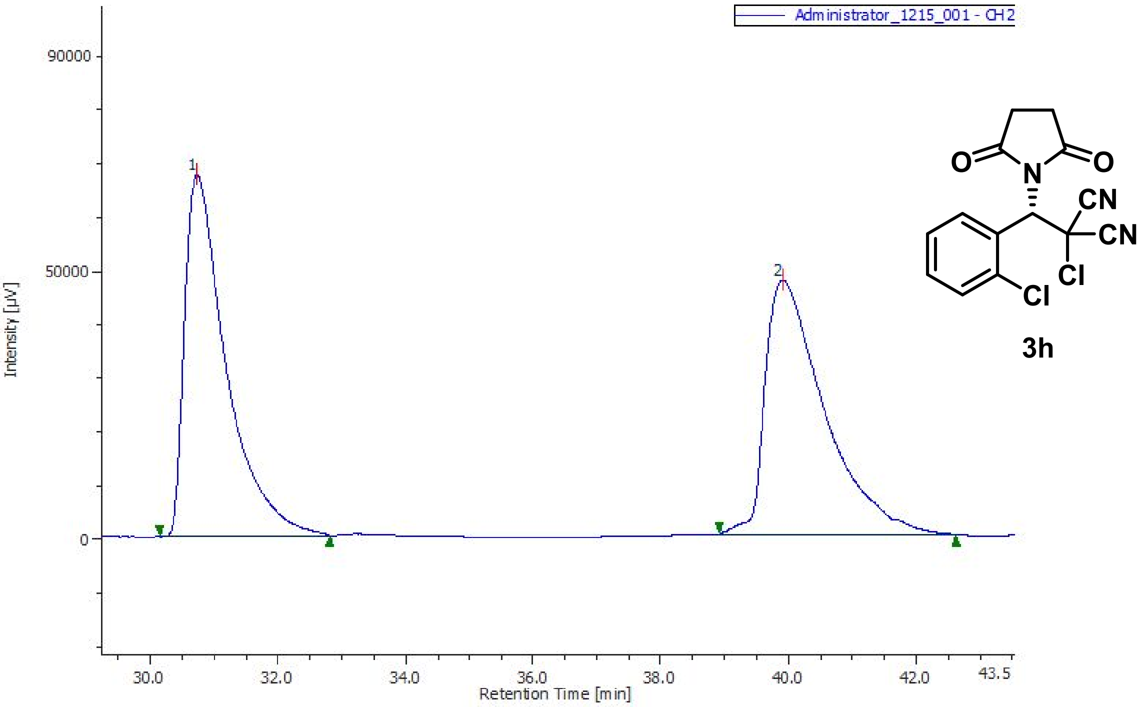


3h

| | tR | Area (μV ・min) | Area (%) |
|---|---|---|---|
| 1 | 30.733 | 3031136 | 49.775 |
| 2 | 39.917 | 3058545 | 50.225 |

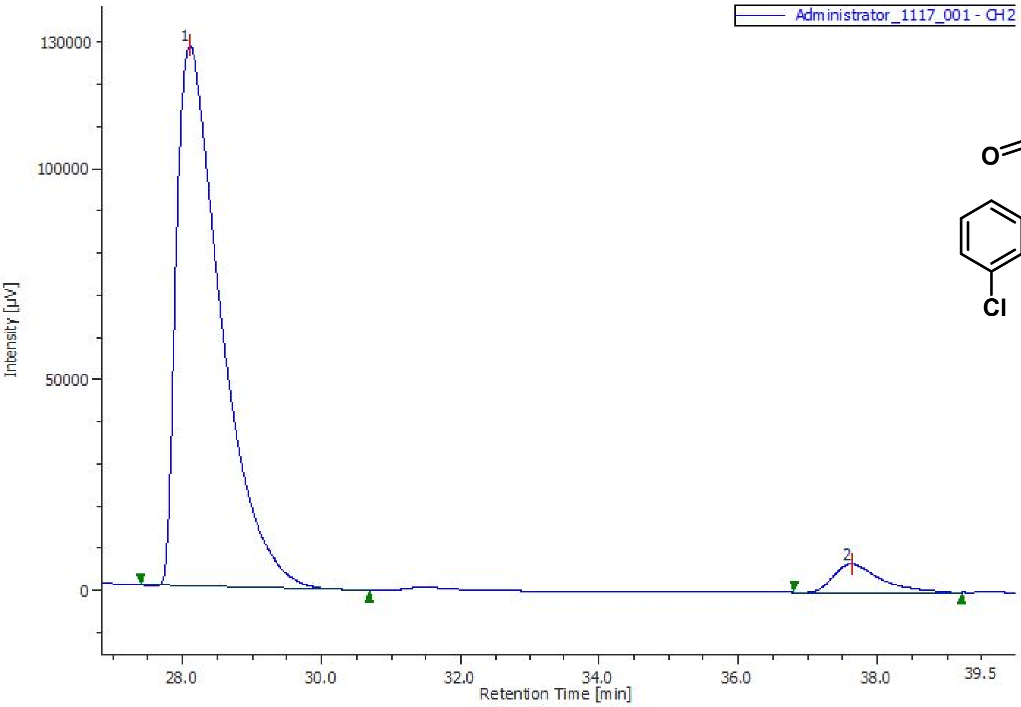


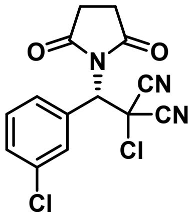


3i

| | tR | Area (μV ・min) | Area (%) |
|---|---|---|---|
| 1 | 28.100 | 5698533 | 94.791 |
| 2 | 37.633 | 313122 | 5.209 |

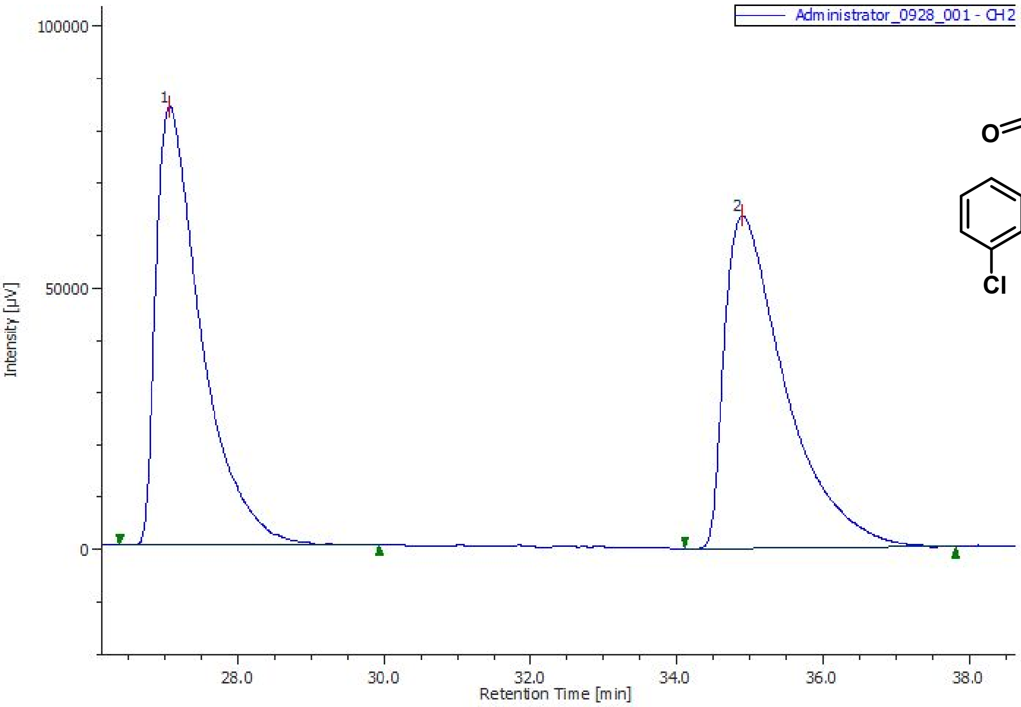


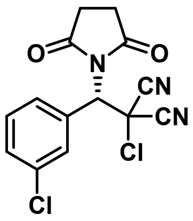


3i

| | tR | Area (μV ・min) | Area (%) |
|---|---|---|---|
| 1 | 27.058 | 3590676 | 49.418 |
| 2 | 34.900 | 3675319 | 50.582 |

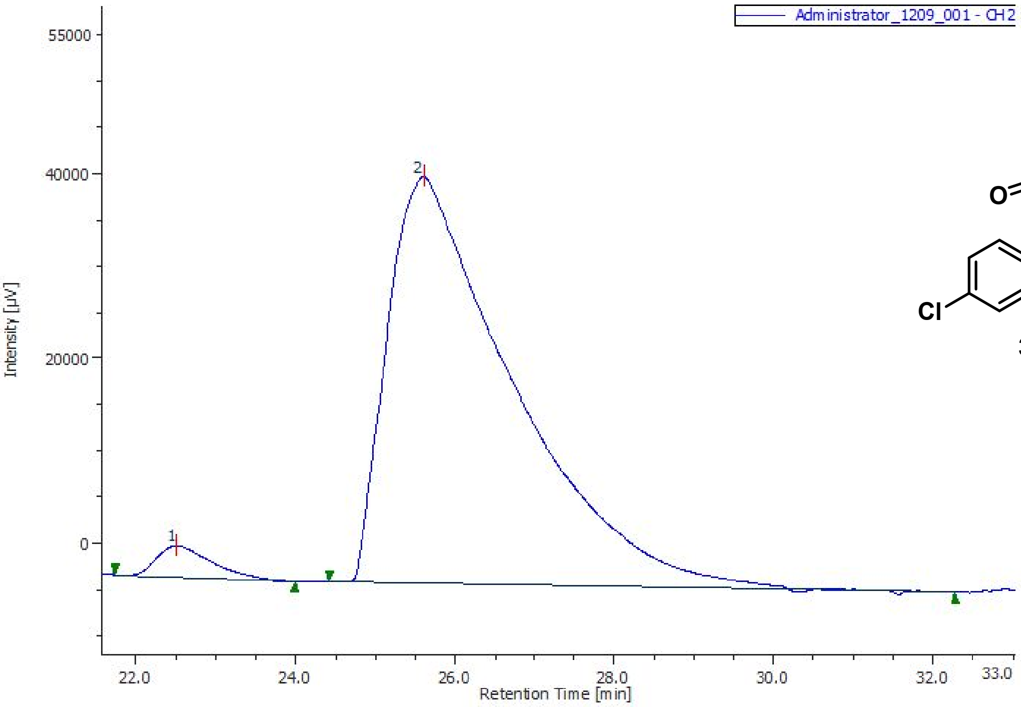


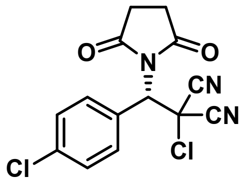


3j

| | tR | Area (µV ・min) | Area (%) |
|---|---|---|---|
| 1 | 22.508 | 160683 | 3.215 |
| 2 | 25.600 | 4836733 | 96.785 |

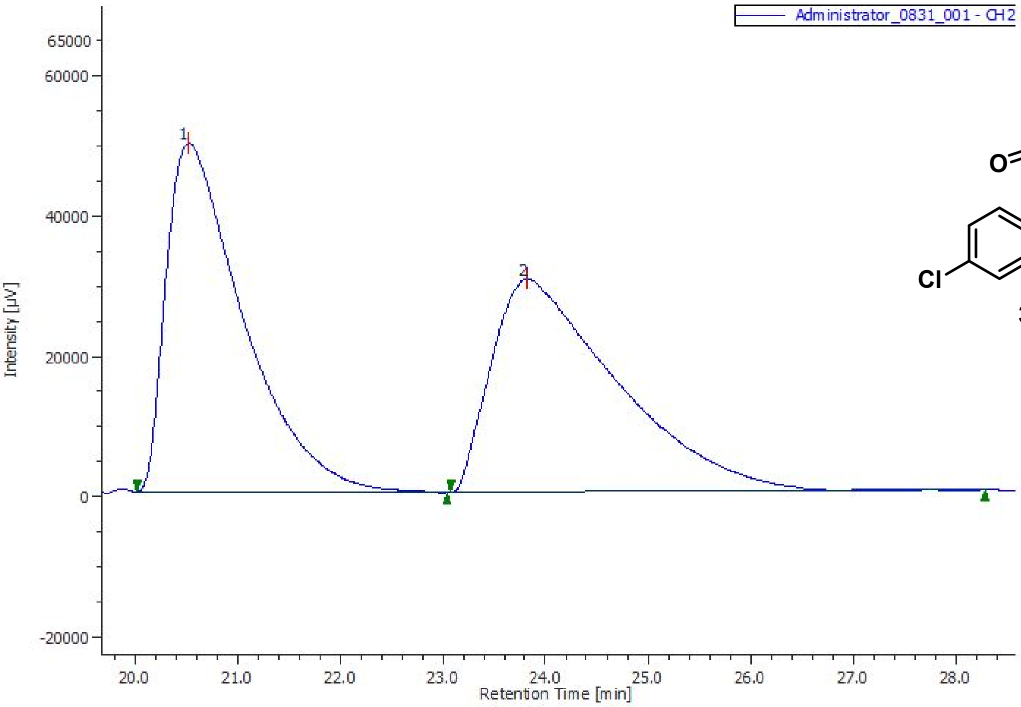


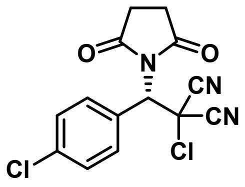


3j

| | tR | Area (µV ・min) | Area (%) |
|---|---|---|---|
| 1 | 20.517 | 2596582 | 50.207 |
| 2 | 23.825 | 2575166 | 49.793 |

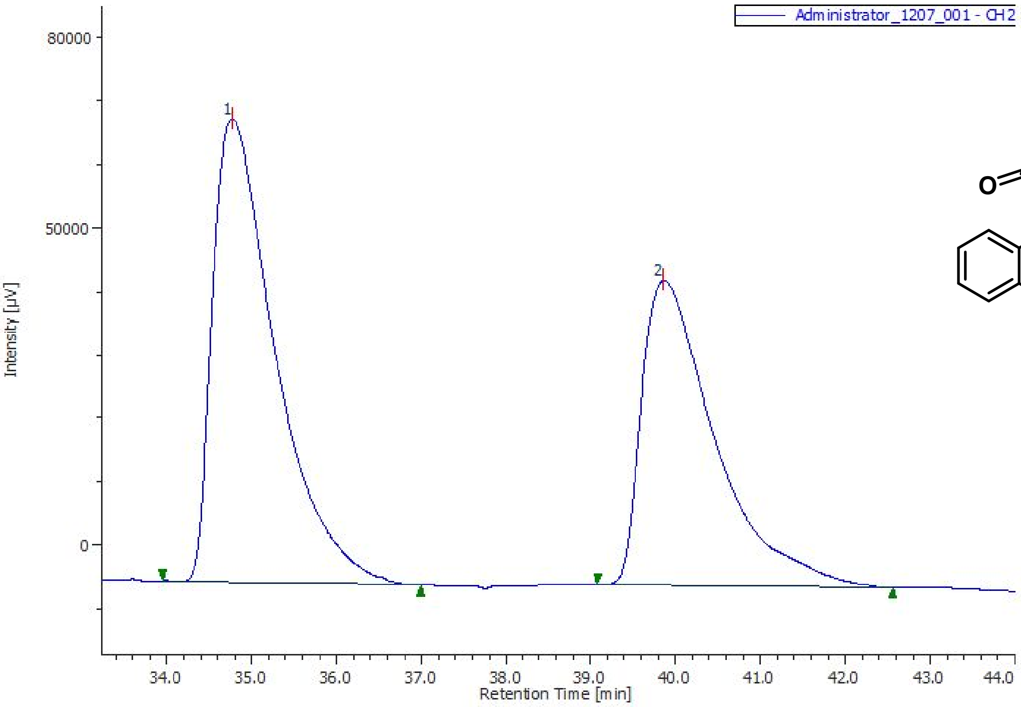


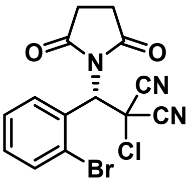

**3k**

| | tR | Area (µV ・min) | Area (%) |
|---|---|---|---|
| 1 | 22.508 | 160683 | 3.215 |
| 2 | 25.600 | 4836733 | 96.785 |

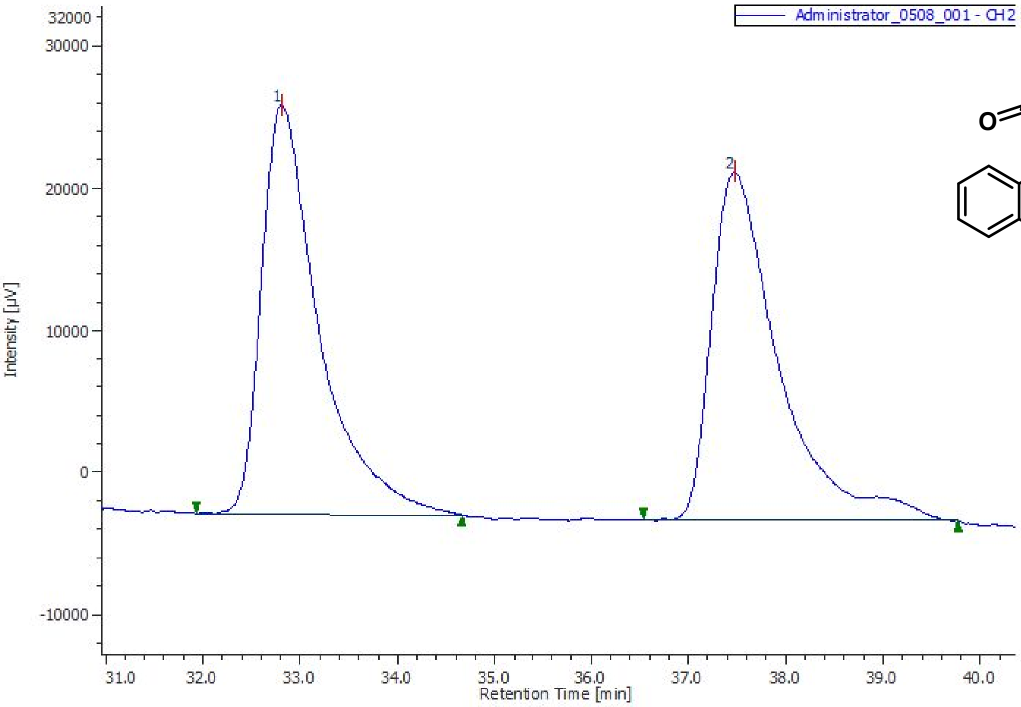


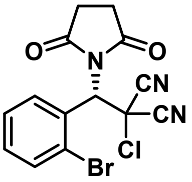

**3k**

| | tR | Area (µV ・min) | Area (%) |
|---|---|---|---|
| 1 | 20.517 | 2596582 | 50.207 |
| 2 | 23.825 | 2575166 | 49.793 |

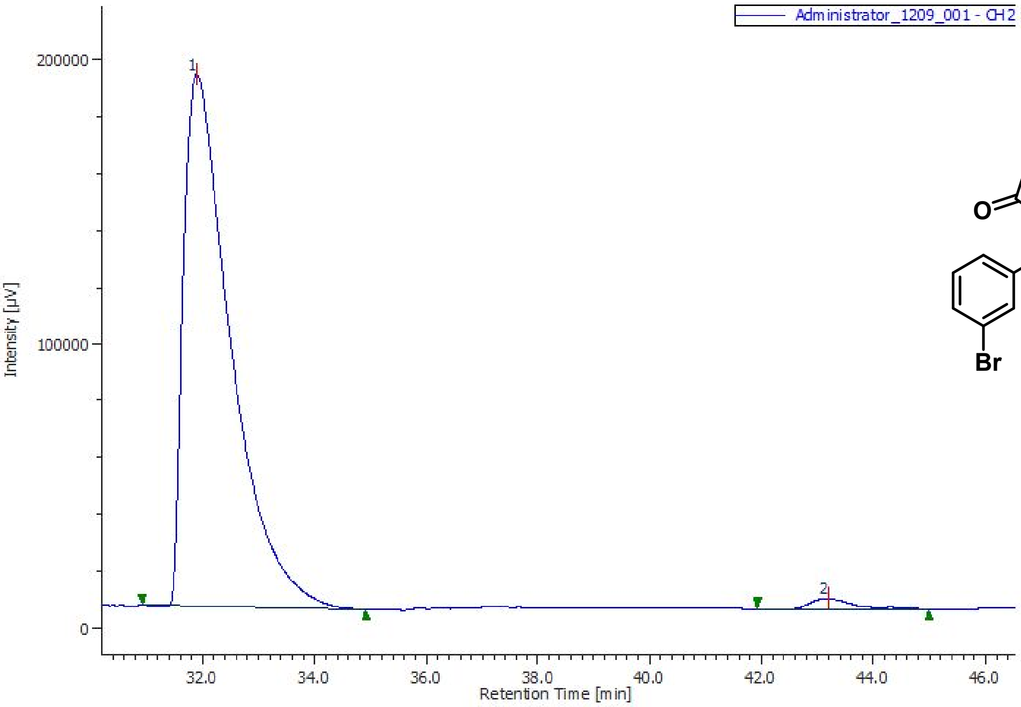


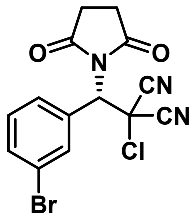


3l

| | tR | Area (μV ・min) | Area (%) |
|---|---|---|---|
| 1 | 31.900 | 10936123 | 98.229 |
| 2 | 43.208 | 189285 | 1.913 |

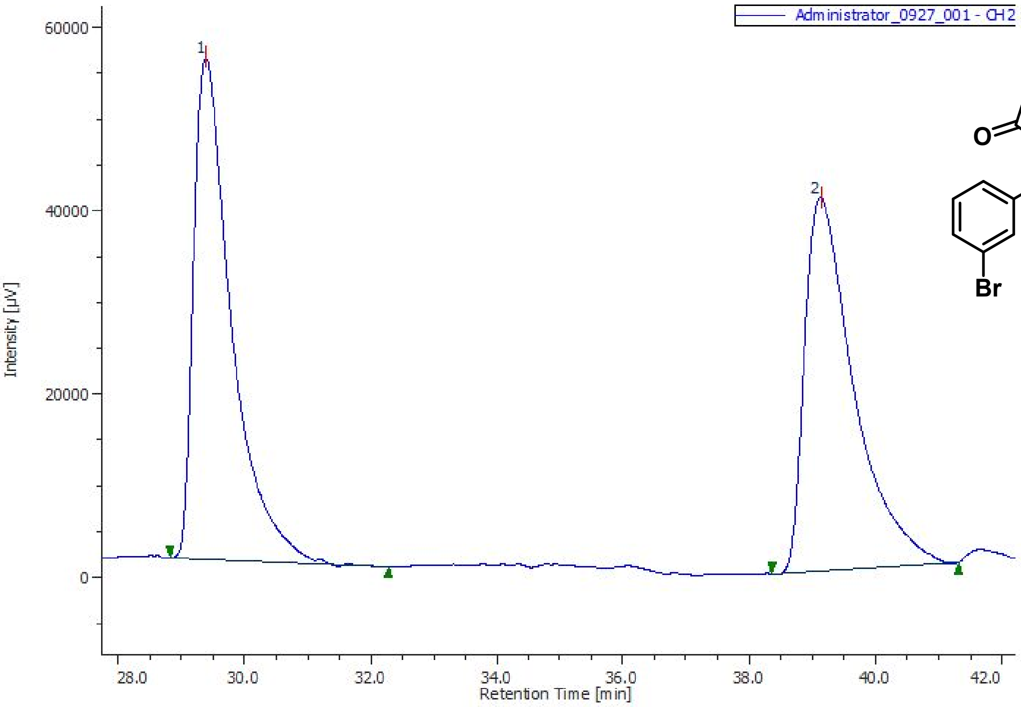


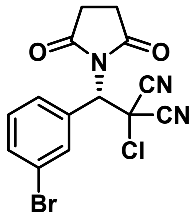


3l

| | tR | Area (μV ・min) | Area (%) |
|---|---|---|---|
| 1 | 29.392 | 2275341 | 50.771 |
| 2 | 39.133 | 2206223 | 49.229 |

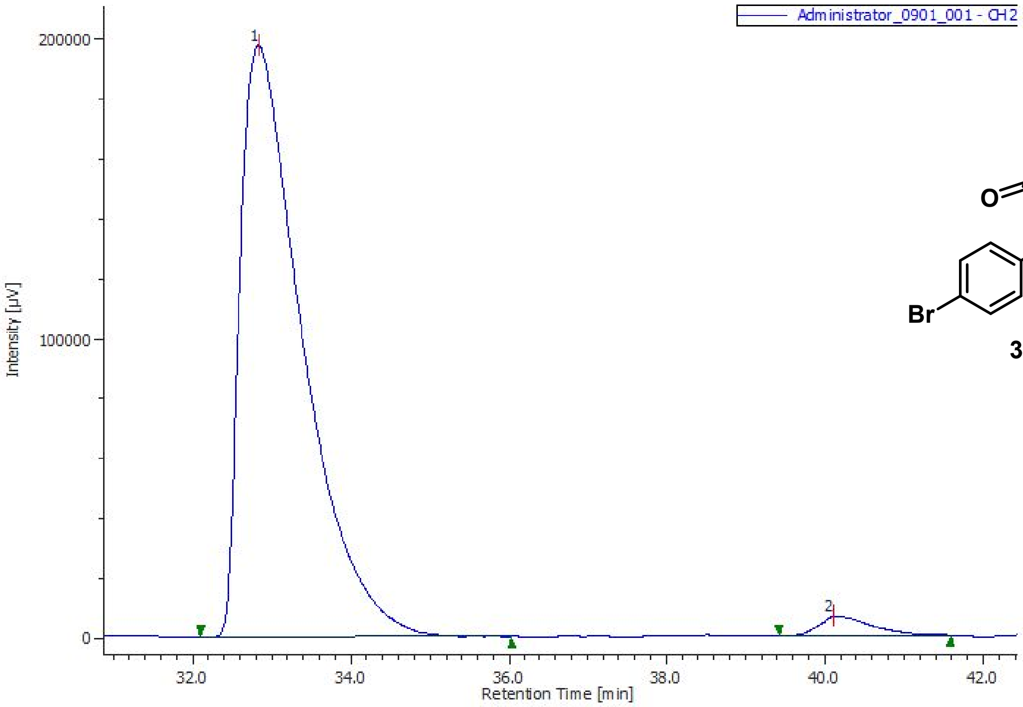


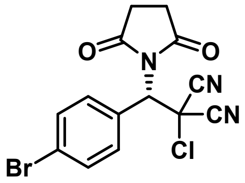


3m

| | tR | Area (μV ・min) | Area (%) |
|---|---|---|---|
| 1 | 32.833 | 10780508 | 97.235 |
| 2 | 40.108 | 306501 | 2.765 |

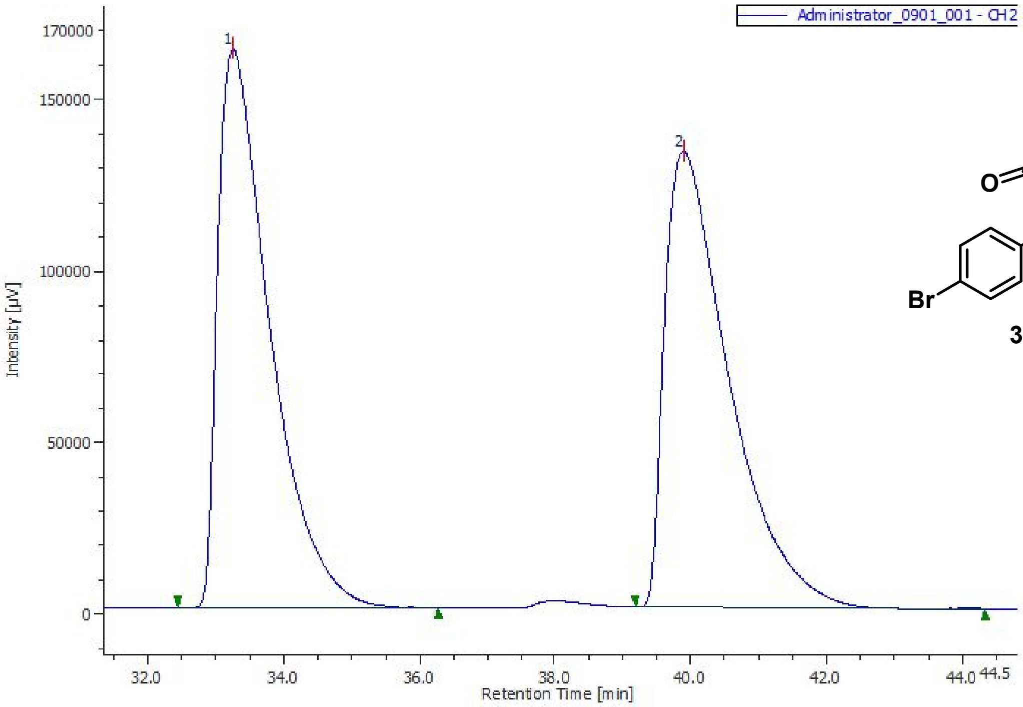


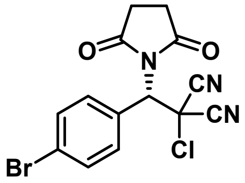


3m

| | tR | Area (μV ・min) | Area (%) |
|---|---|---|---|
| 1 | 33.258 | 8677165 | 49.8 |
| 2 | 39.900 | 8746844 | 50.2 |

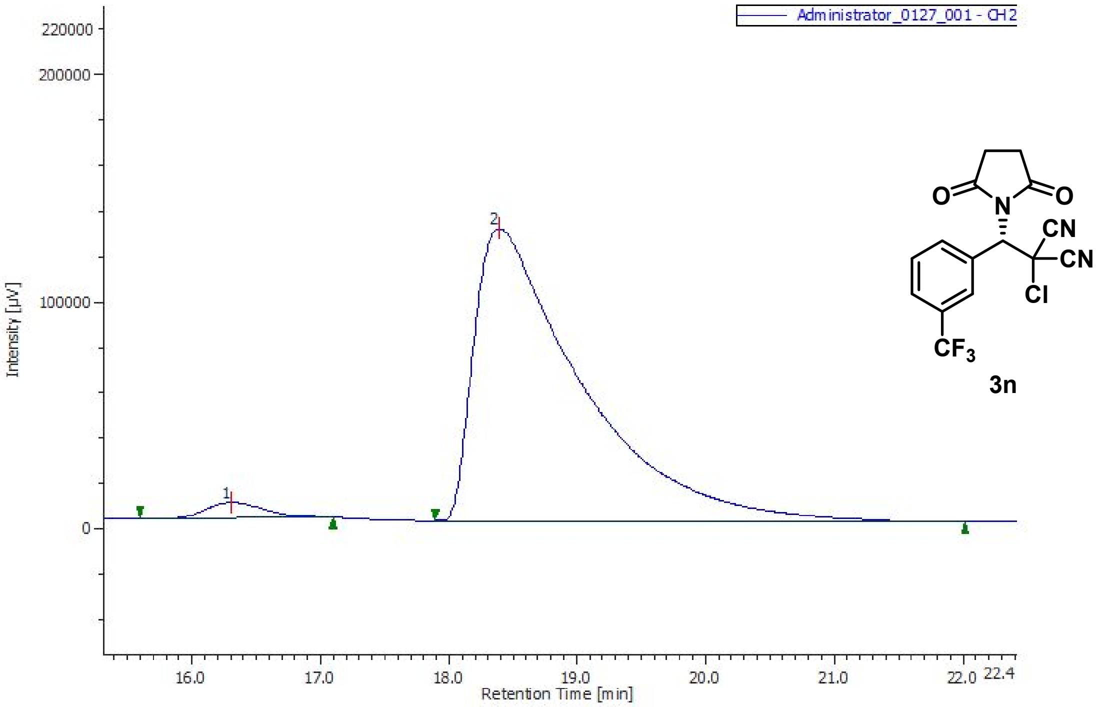


| | tR | Area (μV ・min) | Area (%) |
|---|---|---|---|
| 1 | 16.308 | 199523 | 2.58 |
| 2 | 18.383 | 7534481 | 97.42 |

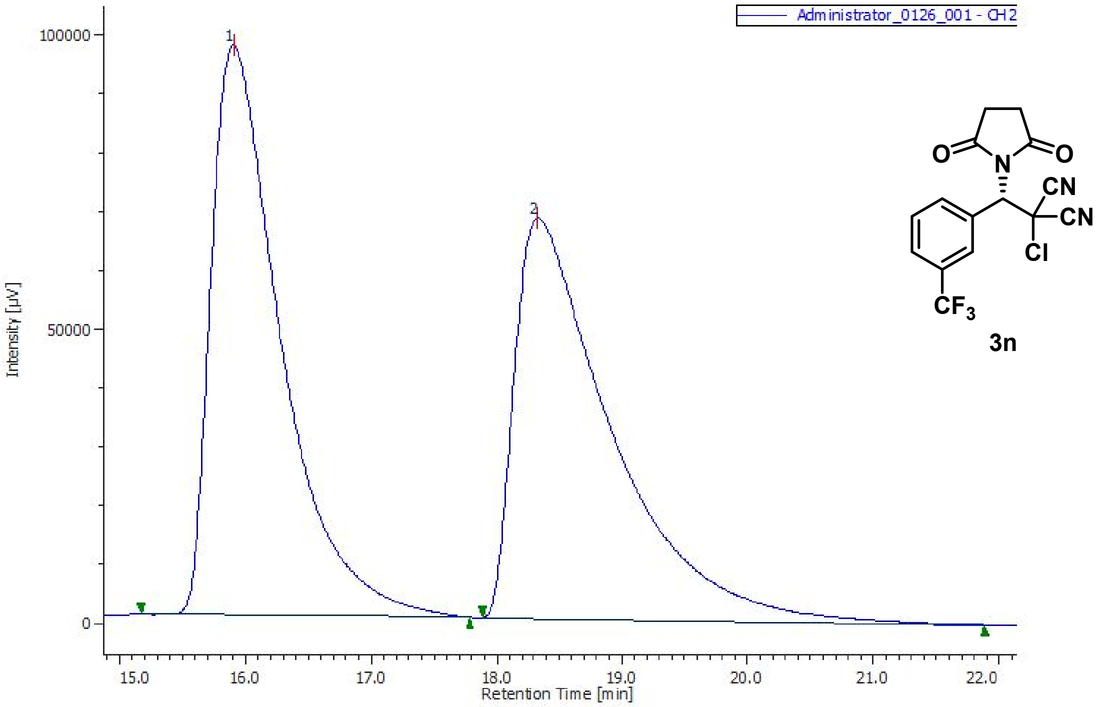


| | tR | Area (μV ・min) | Area (%) |
|---|---|---|---|
| 1 | 15.908 | 3722670 | 50.229 |
| 2 | 18.333 | 3688752 | 49.771 |

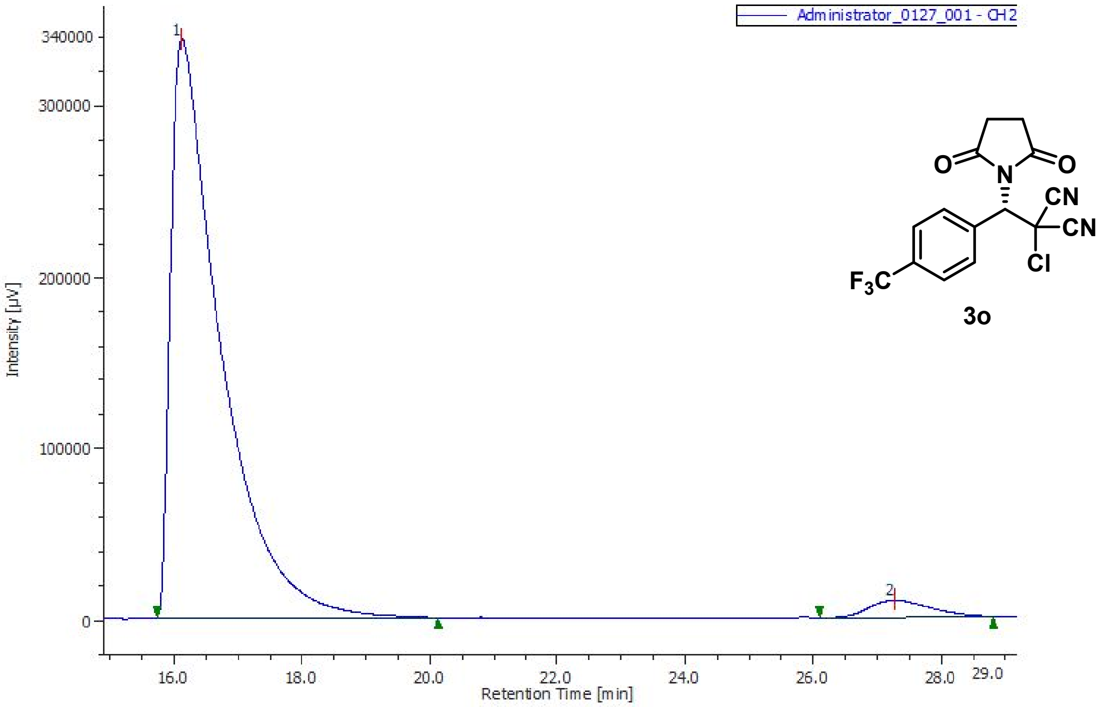


| | tR | Area (µV ・min) | Area (%) |
|---|---|---|---|
| 1 | 16.125 | 18291542 | 96.582 |
| 2 | 27.283 | 647244 | 3.418 |

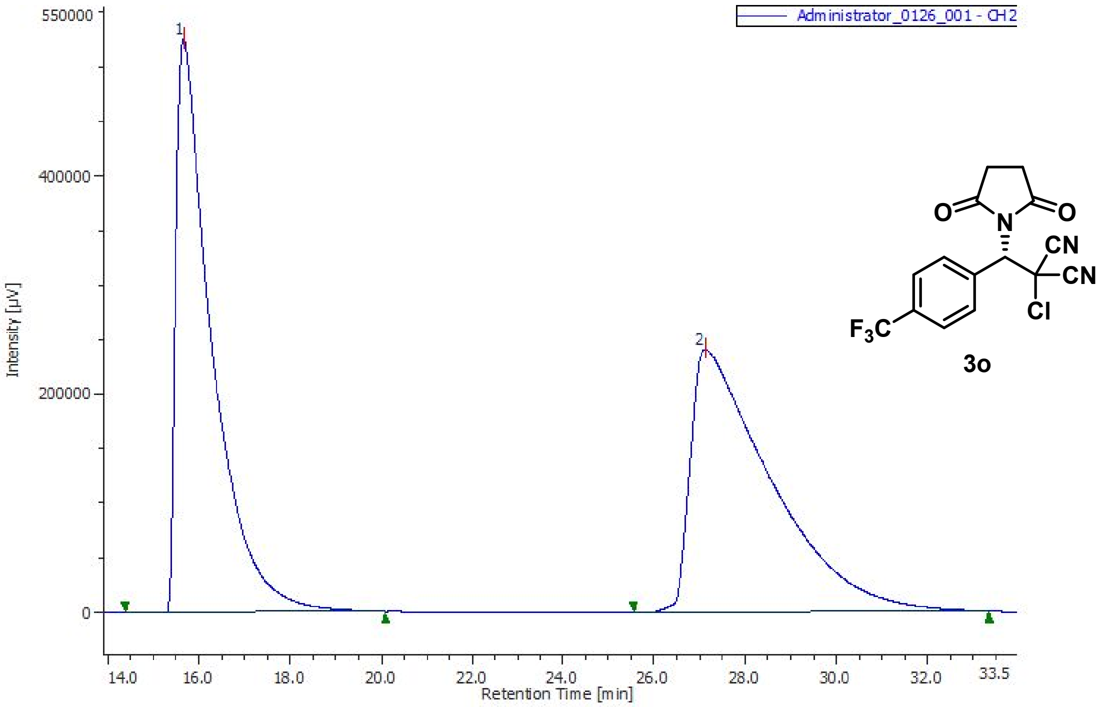


| | tR | Area (µV ・min) | Area (%) |
|---|---|---|---|
| 1 | 15.667 | 28936154 | 49.496 |
| 2 | 27.125 | 29525294 | 50.504 |

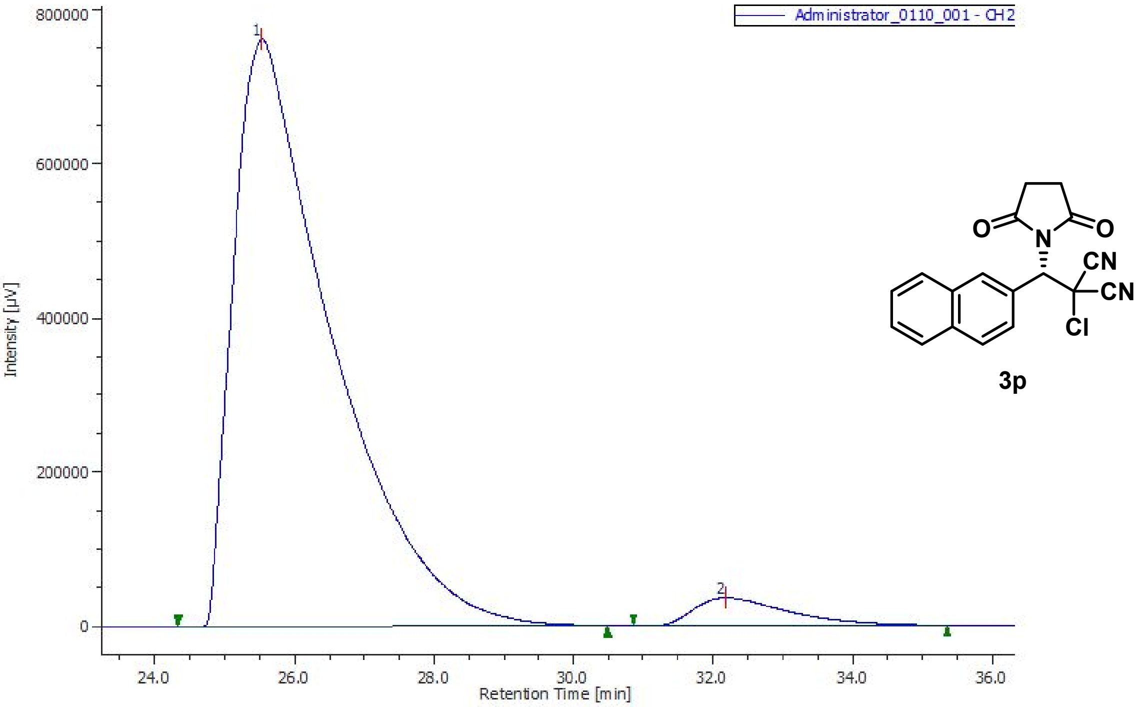


**3p**

| | tR | Area (μV ・min) | Area (%) |
|---|---|---|---|
| 1 | 25.533 | 74319968 | 95.654 |
| 2 | 32.183 | 3376740 | 4.346 |

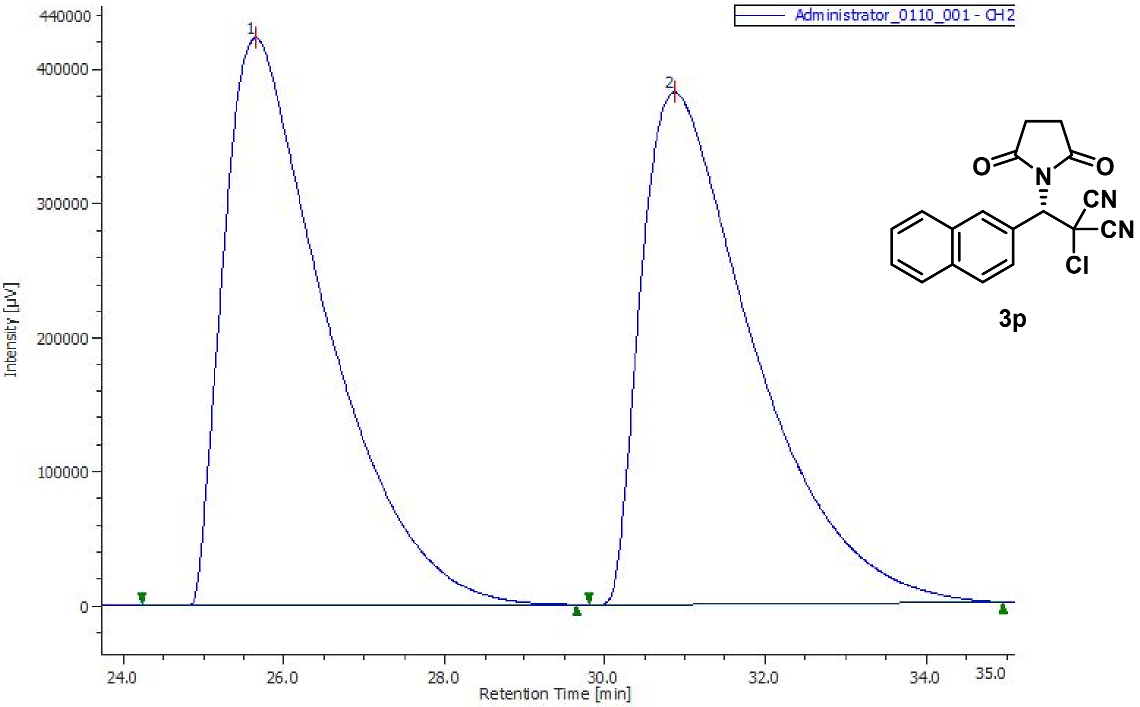


**3p**

| | tR | Area (μV ・min) | Area (%) |
|---|---|---|---|
| 1 | 25.650 | 37724940 | 50.313 |
| 2 | 30.875 | 37255241 | 47.472 |

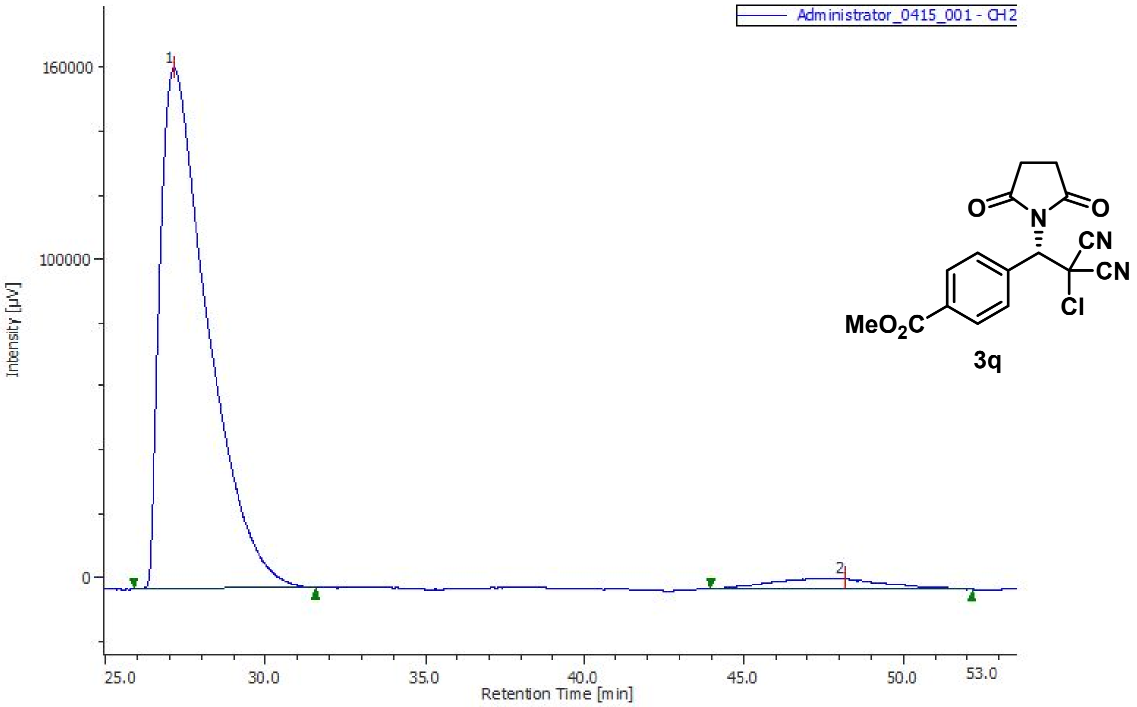


| | tR | Area (μV ・min) | Area (%) |
|---|---|---|---|
| 1 | 27.142 | 16921673 | 95.369 |
| 2 | 48.183 | 821627 | 4.631 |

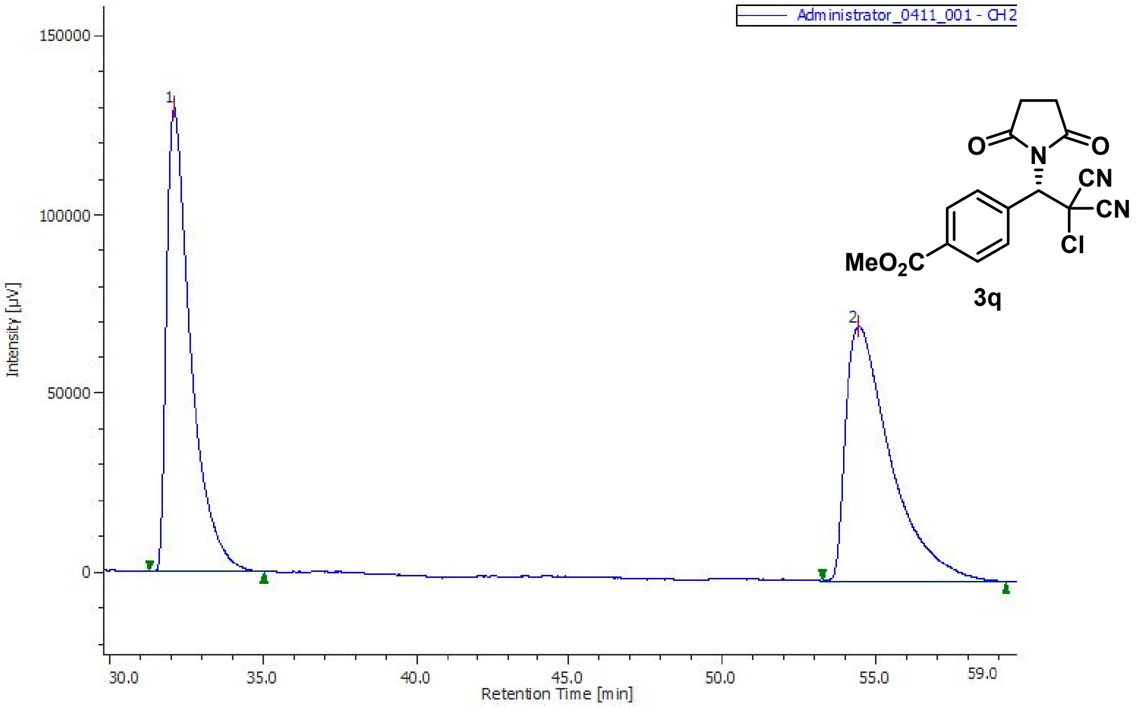


| | tR | Area (μV ・min) | Area (%) |
|---|---|---|---|
| 1 | 32.108 | 7085634 | 49.418 |
| 2 | 54.45 | 7252607 | 50.582 |

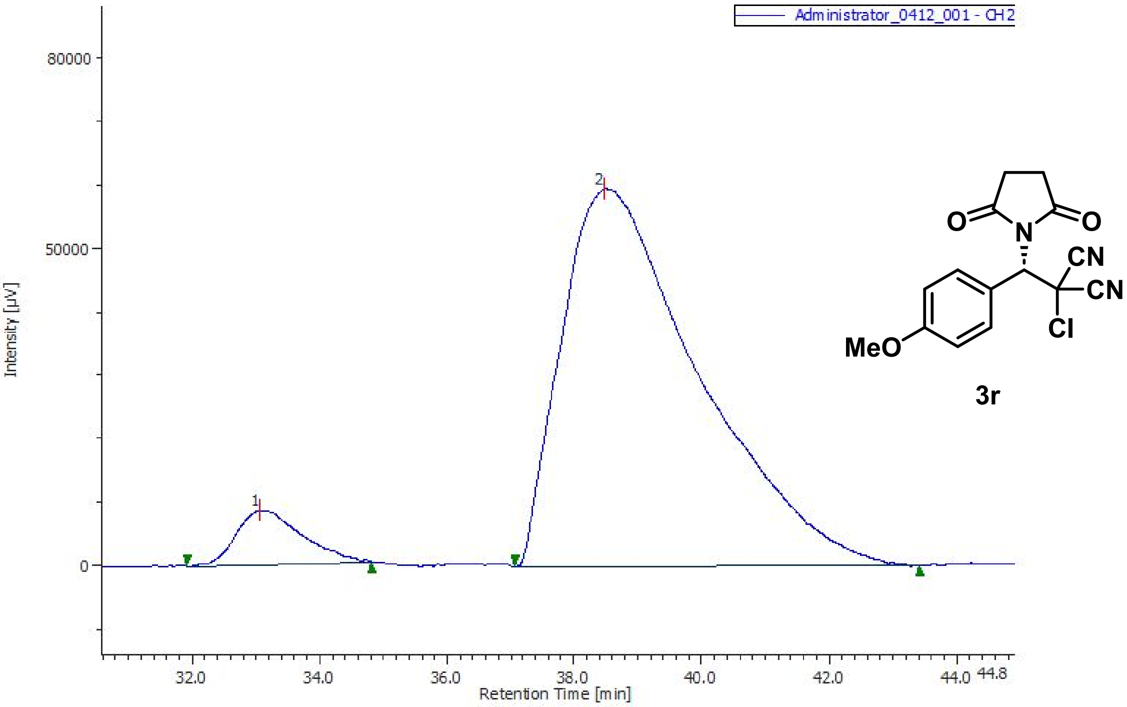


| | tR | Area (μV ・min) | Area (%) |
|---|---|---|---|
| 1 | 33.058 | 598880 | 6.342 |
| 2 | 38.483 | 8843664 | 93.658 |

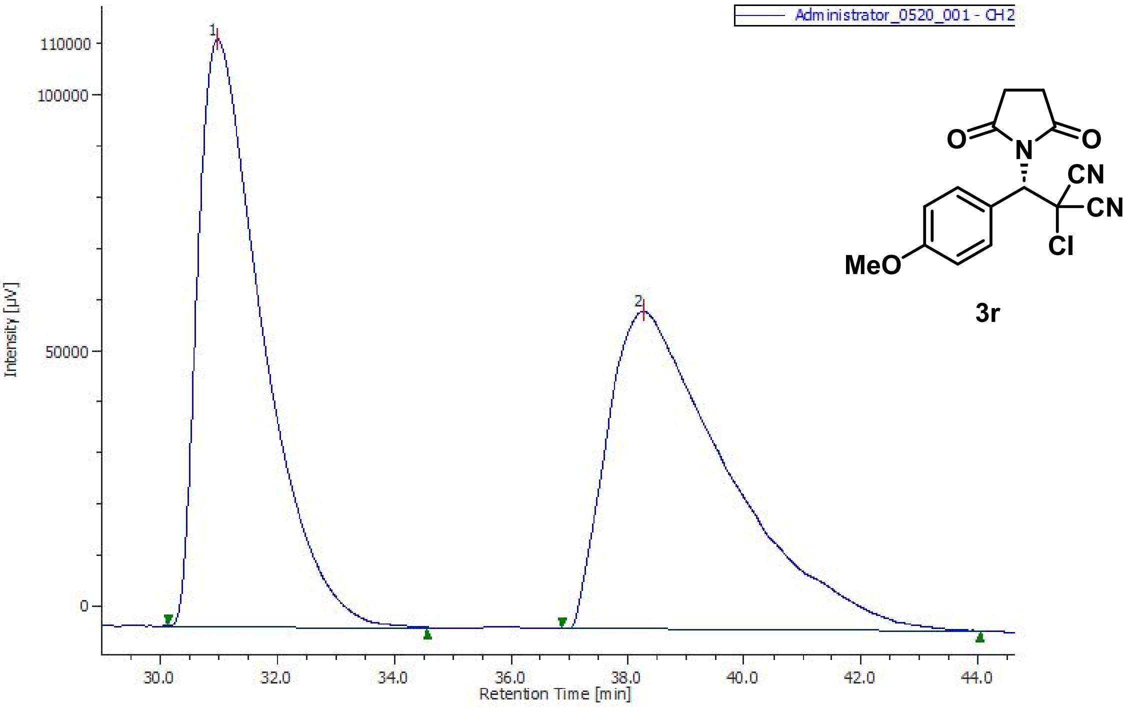


| | tR | Area (μV ・min) | Area (%) |
|---|---|---|---|
| 1 | 30.992 | 8831182 | 49.769 |
| 2 | 38.275 | 8913331 | 50.231 |

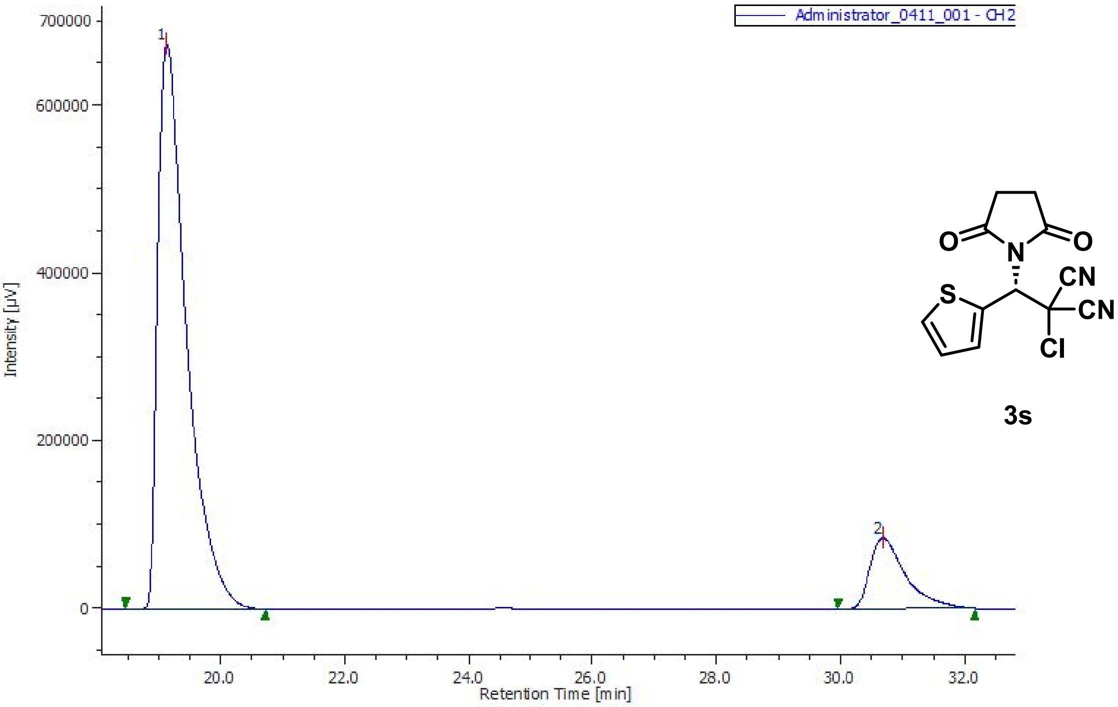


| | tR | Area (μV ・min) | Area (%) |
|---|---|---|---|
| 1 | 19.133 | 21499334 | 86.345 |
| 2 | 30.683 | 3400076 | 13.655 |

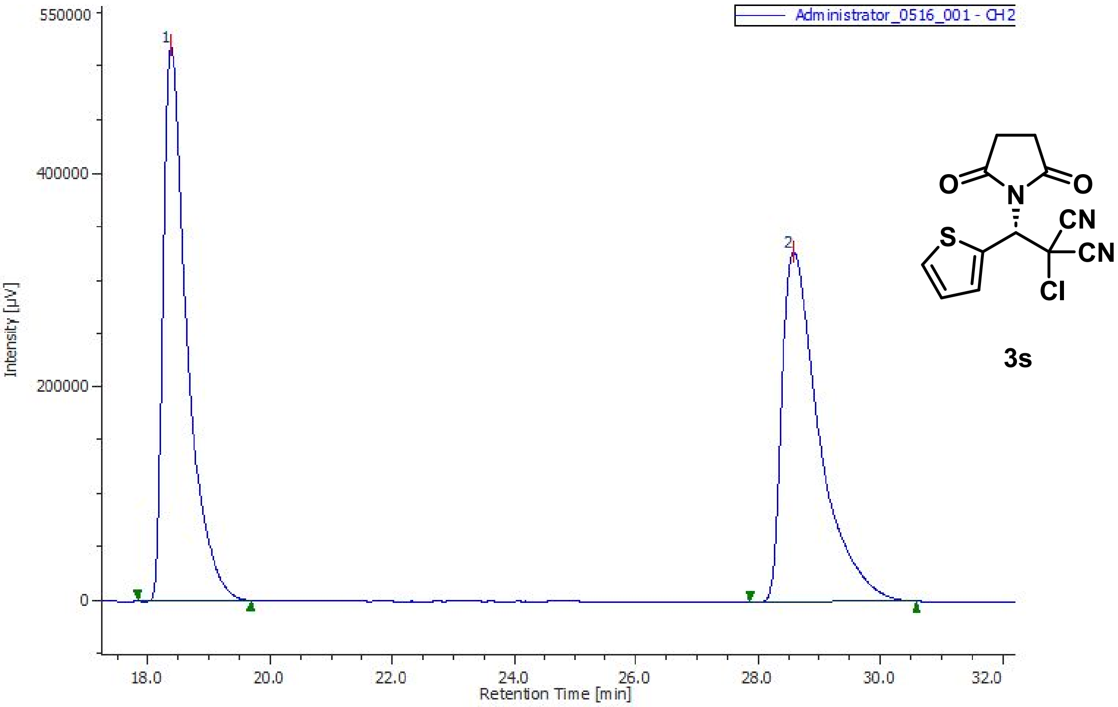


| | tR | Area (μV ・min) | Area (%) |
|---|---|---|---|
| 1 | 18.383 | 14101963 | 50.332 |
| 2 | 28.583 | 13916172 | 49.668 |

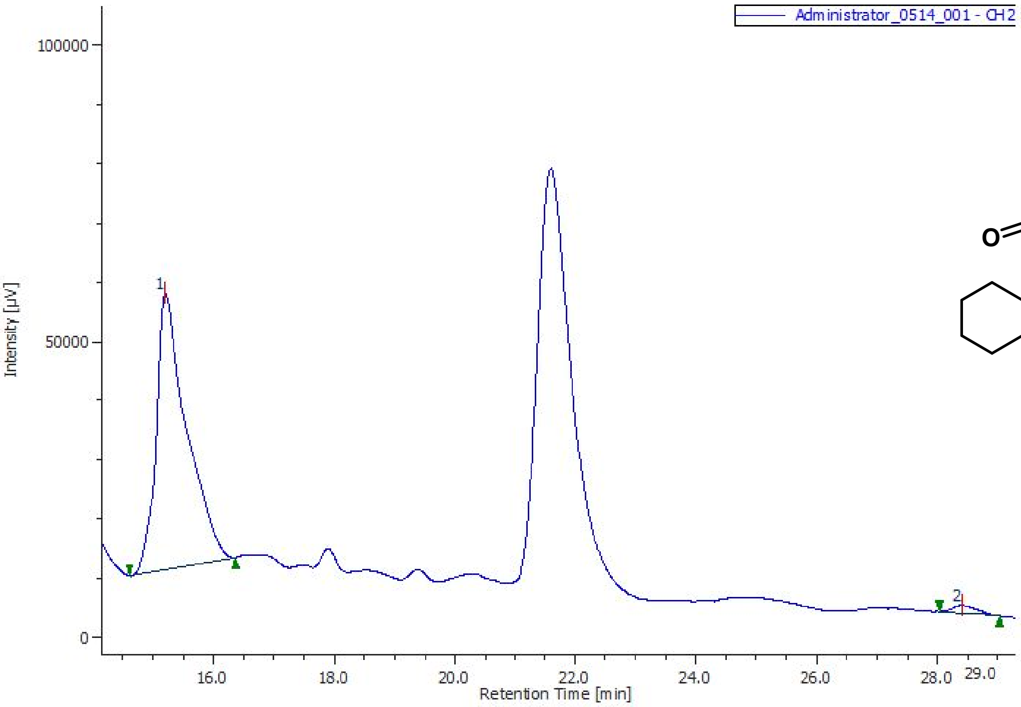


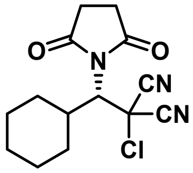


**3t**

| | tR | Area (μV ・min) | Area (%) |
|---|---|---|---|
| 1 | 15.208 | 1621043 | 97.839 |
| 2 | 28.400 | 35807 | 2.161 |

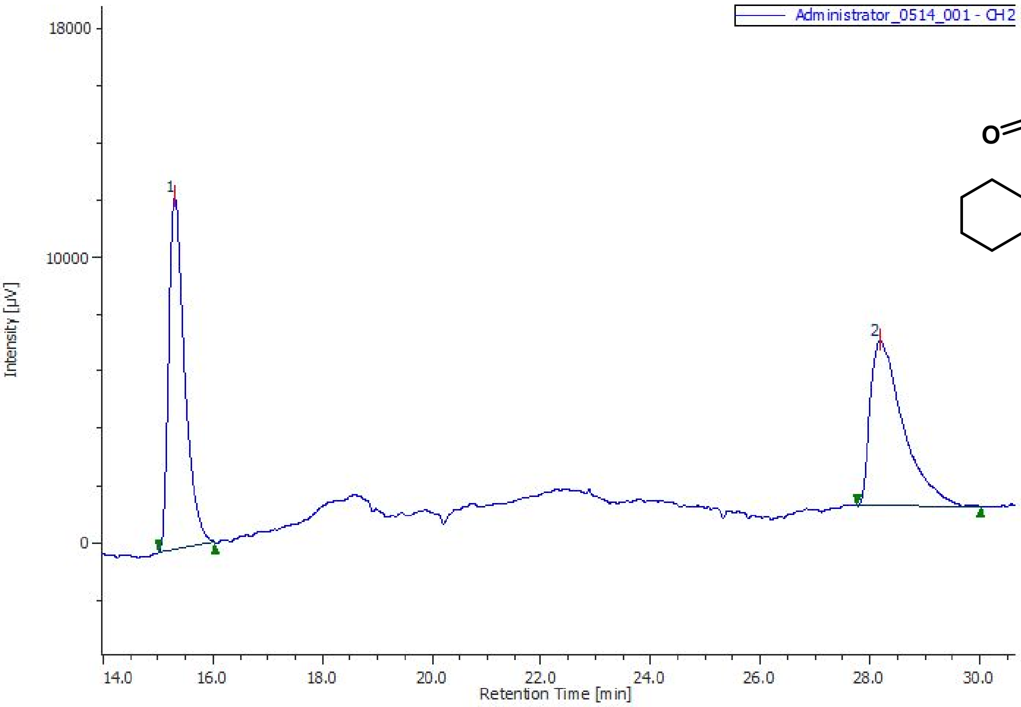


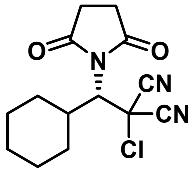


**3t**

| | tR | Area (μV ・min) | Area (%) |
|---|---|---|---|
| 1 | 15.308 | 250208 | 50.888 |
| 2 | 28.183 | 241476 | 49.112 |

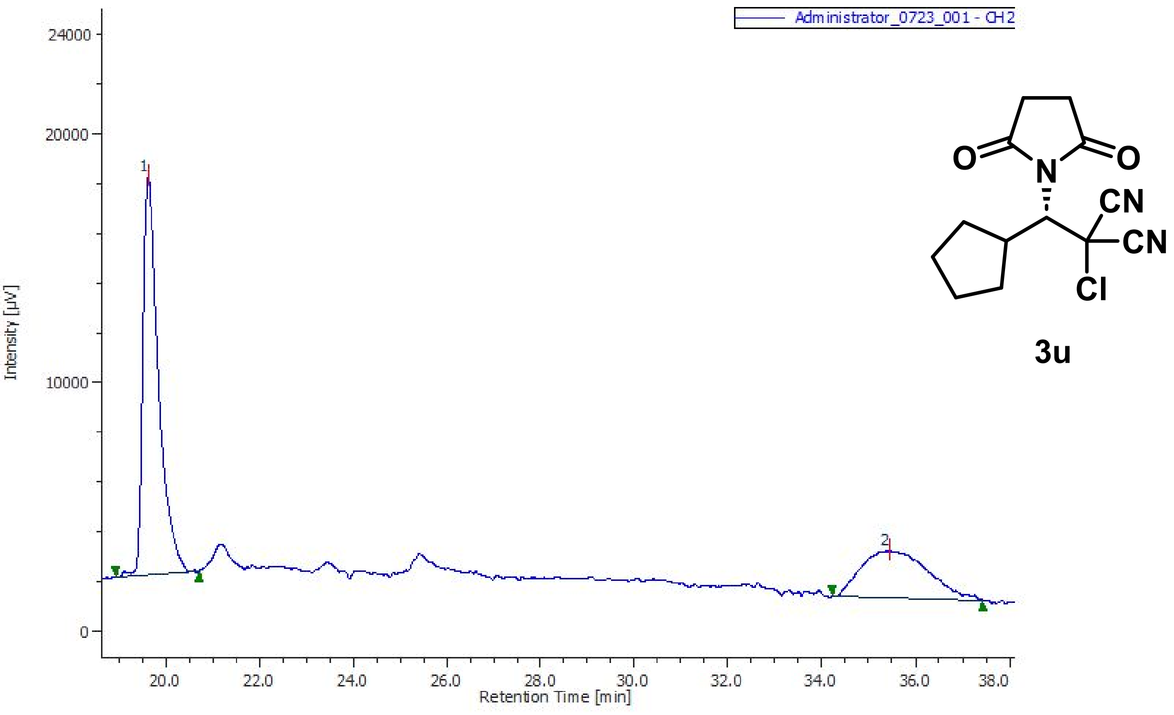


**3u**

| | tR | Area (μV ・min) | Area (%) |
|---|---|---|---|
| 1 | 19.625 | 371083 | 66.469 |
| 2 | 35.45 | 187200 | 33.531 |

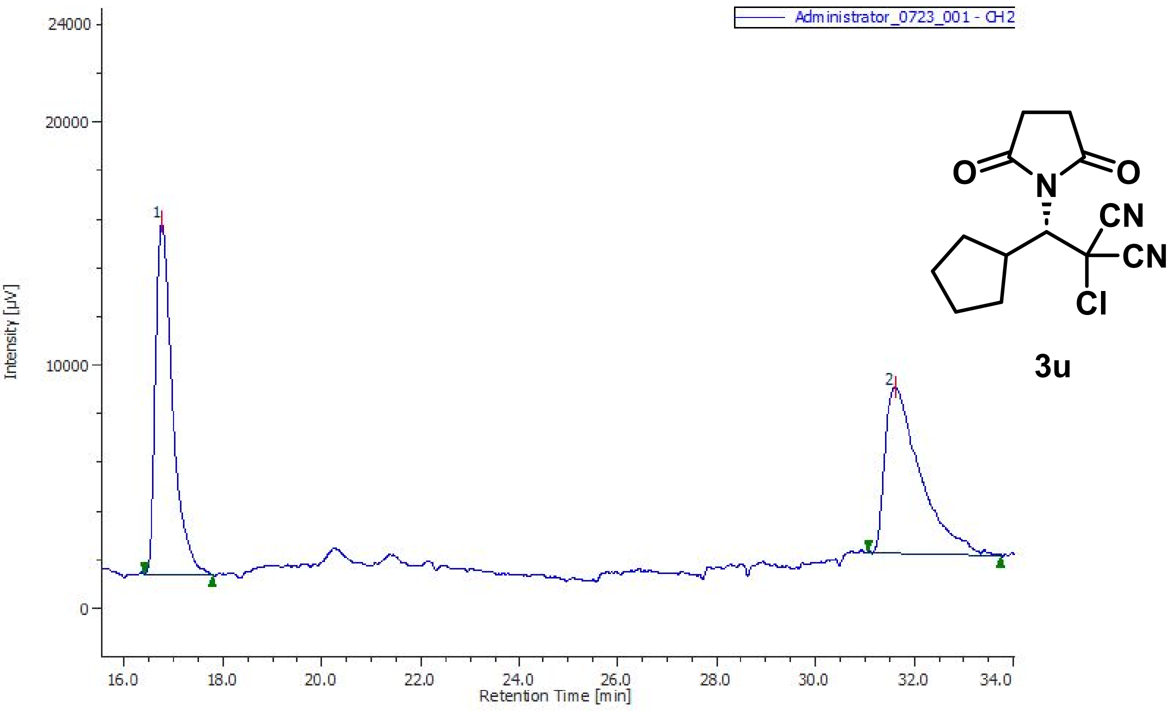


**3u**

| | tR | Area (μV ・min) | Area (%) |
|---|---|---|---|
| 1 | 16.767 | 345801 | 50.593 |
| 2 | 31.608 | 337696 | 49.407 |

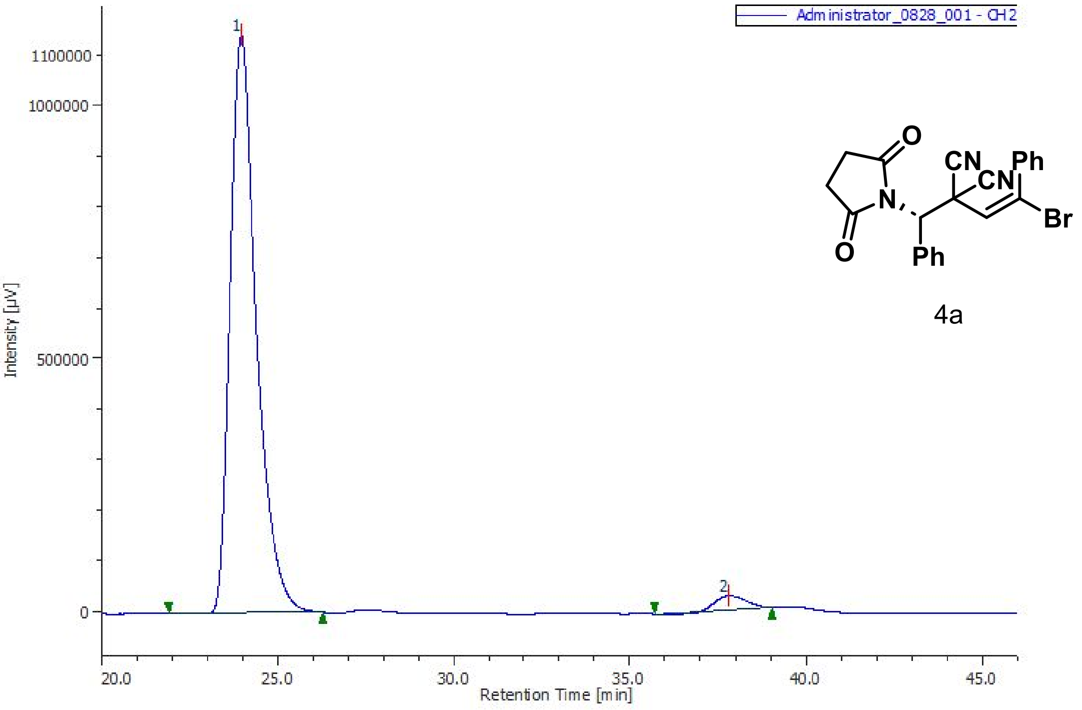


| | tR | Area (μV・min) | Area (%) |
|---|---|---|---|
| 1 | 23.950 | 59051442 | 97.206 |
| 2 | 37.817 | 1697560 | 2.794 |

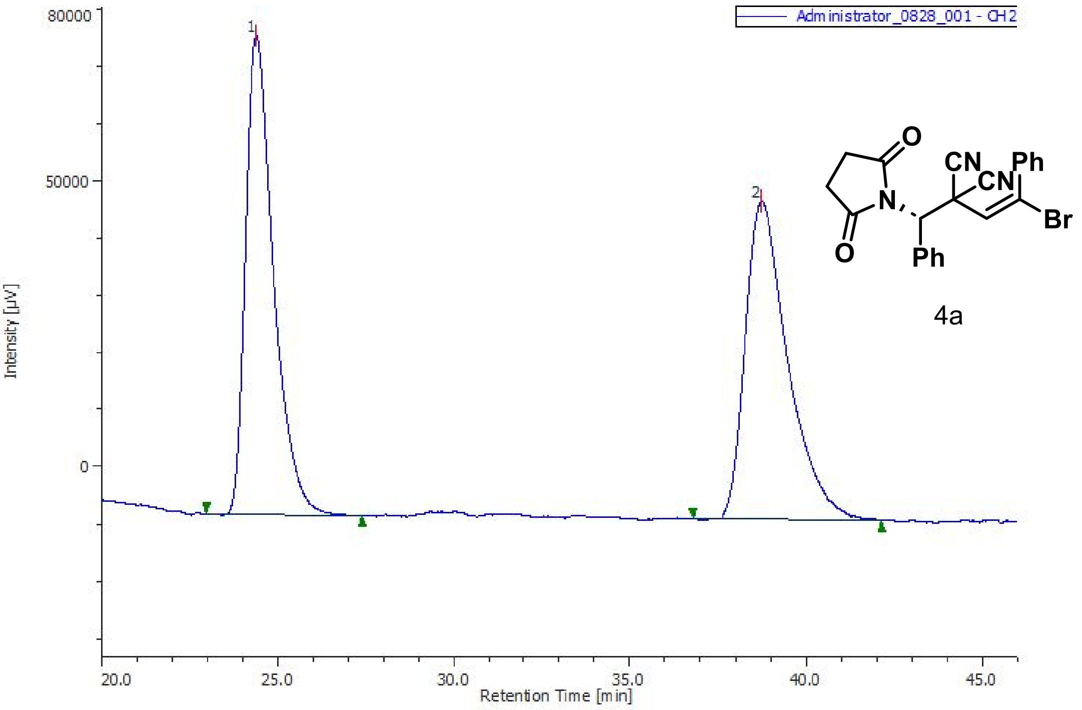


| | tR | Area (μV ・min) | Area (%) |
|---|---|---|---|
| 1 | 24.375 | 4605844 | 49.637 |
| 2 | 38.742 | 4673224 | 50.363 |

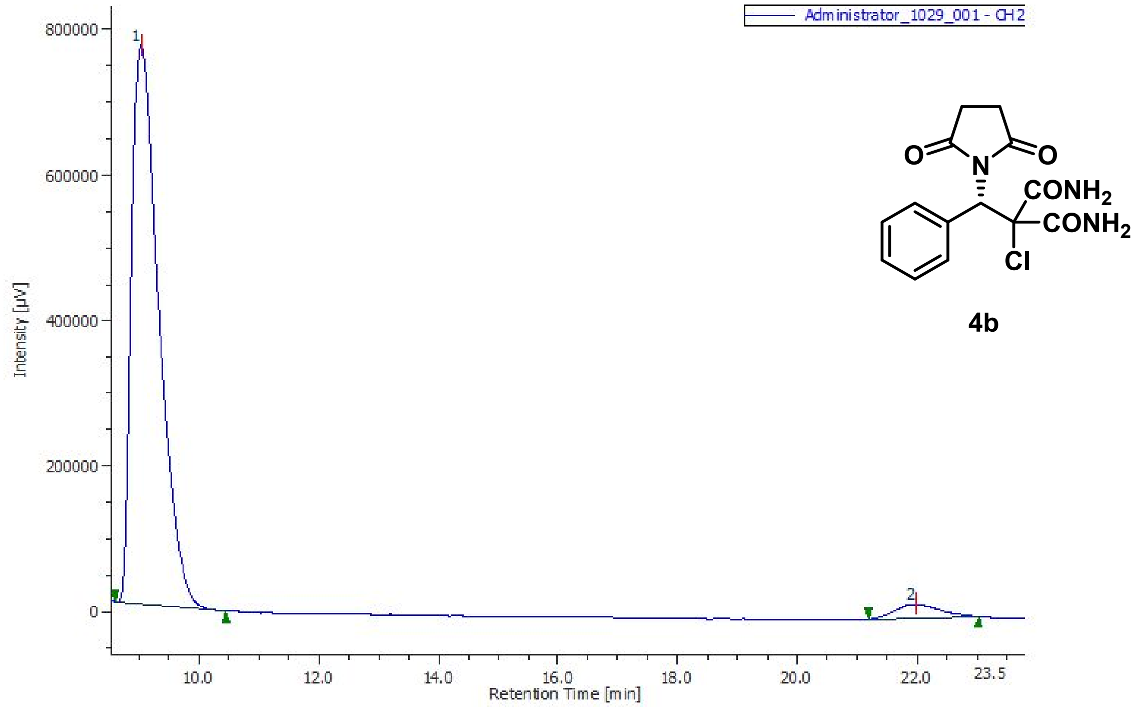


| | tR | Area (μV ・min) | Area (%) |
|---|---|---|---|
| 1 | 9.042 | 24327635 | 96.155 |
| 2 | 21.983 | 972917 | 3.845 |

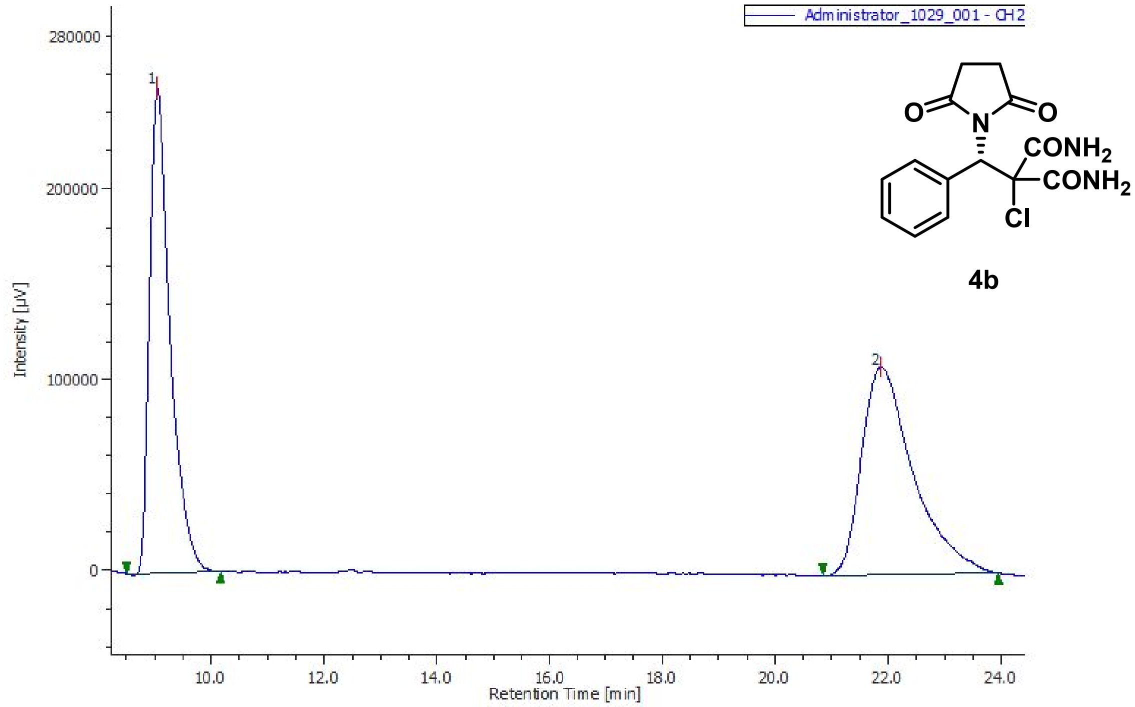


| | tR | Area (μV ・min) | Area (%) |
|---|---|---|---|
| 1 | 9.050 | 6397548 | 48.306 |
| 2 | 21.867 | 6846358 | 51.694 |

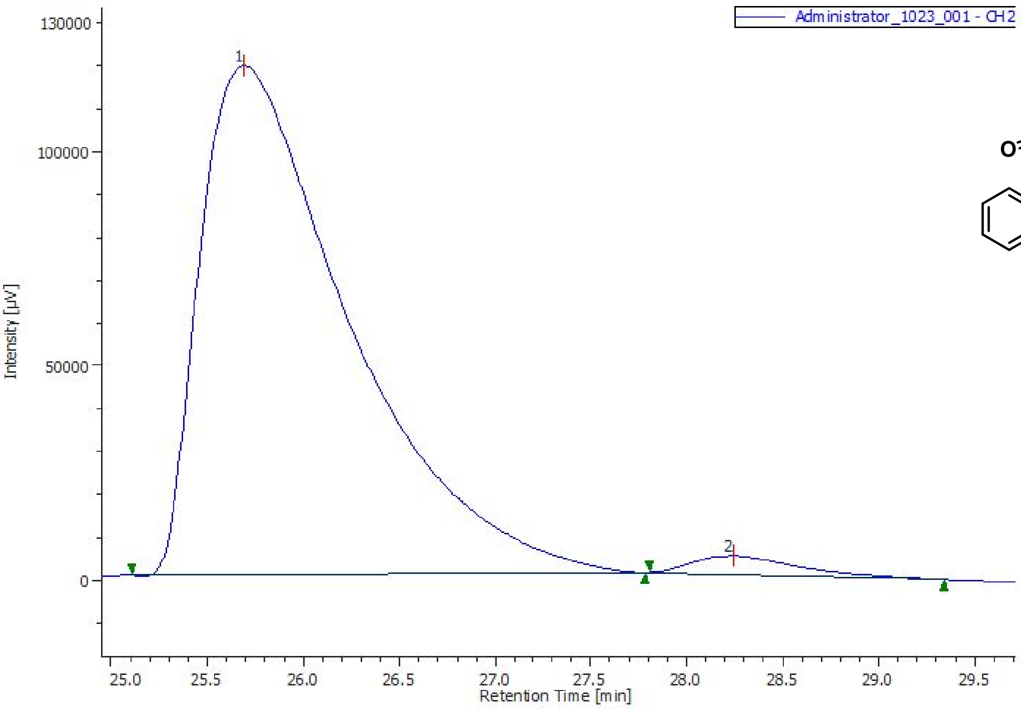


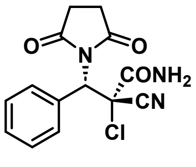


**4c**

| | tR | Area (µV ・min) | Area (%) |
|---|---|---|---|
| 1 | 25.692 | 6403175 | 97.571 |
| 2 | 28.242 | 159389 | 2.429 |

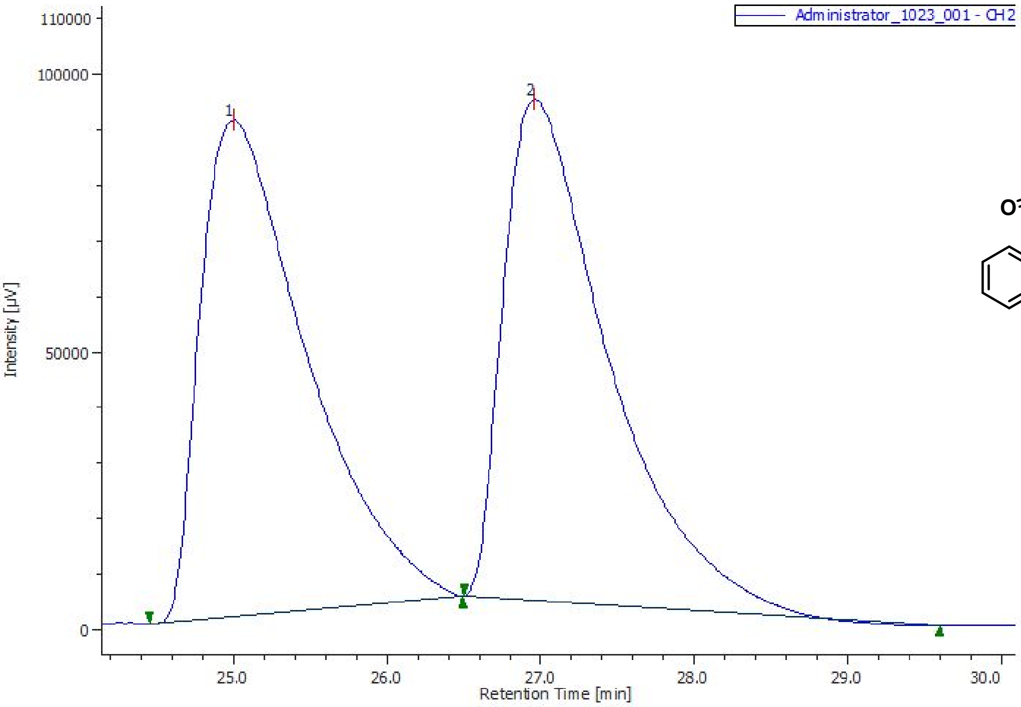


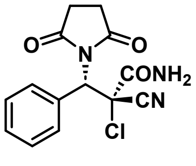


**4c**

| | tR | Area (µV ・min) | Area (%) |
|---|---|---|---|
| 1 | 25.000 | 4287522 | 49.710 |
| 2 | 26.967 | 4337526 | 50.290 |
